# Gold‐Catalyzed Cyclization of Yndiamides with Isoxazoles via α‐Imino Gold Fischer Carbenes

**DOI:** 10.1002/chem.202302821

**Published:** 2023-10-25

**Authors:** Zixuan Tong, Philip J. Smith, Helena D. Pickford, Kirsten E. Christensen, Edward A. Anderson

**Affiliations:** ^1^ Chemistry Research Laboratory Department of Chemistry University of Oxford 12 Mansfield Road Oxford OX1 3TA UK

**Keywords:** carbene, cyclization, gold catalysis, pyrrole, yndiamide

## Abstract

Gold catalysis is an important method for alkyne functionalization. Here we report the gold‐catalyzed formal [3+2] aminative cyclization of yndiamides and isoxazoles in a direct synthesis of polysubstituted diaminopyrroles, which are important motifs in drug discovery. Key to this process is the formation, and subsequent cyclization, of an α‐imino gold Fischer carbene, which represents a new type of gold carbene intermediate. The reaction proceeds rapidly under mild conditions, with high regioselectivity being achieved by introducing a subtle steric bias between the nitrogen substituents on the yndiamide. DFT calculations revealed that the key to this regioselectivity was the interconversion of isomeric gold keteniminiun ions via a low‐barrier π‐complex transition state, which establishes a Curtin‐Hammett scenario for isoxazole addition. By using benzisoxazoles as substrates, the reaction outcome could be switched to a formal [5+2] cyclization, leading to 1,4‐oxazepines.

## Graphical abstract

Gold‐catalyzed [3+2] aminative cyclization of yndiamides and isoxazoles affords polysubstituted diaminopyrroles via the intermediacy of an α‐imino gold Fischer carbene. The reaction proceeds rapidly under mild conditions; high regioselectivity was achieved via a subtle steric bias between the nitrogen substituents, as supported by DFT calculations. The reaction outcome could be switched to 1,4‐oxazepines by using benzisoxazoles as substrates.

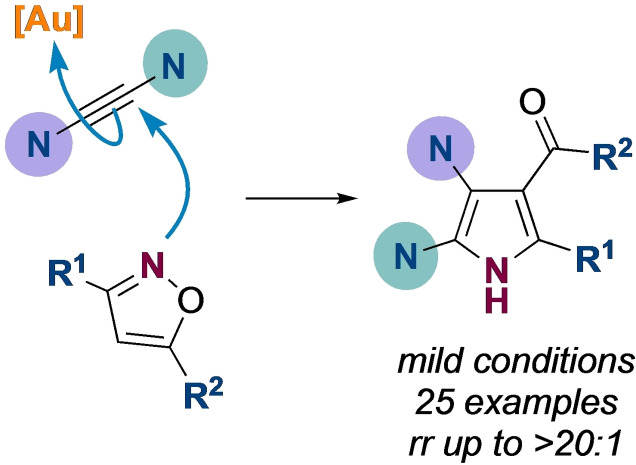

## Introduction

**Scheme 1:**
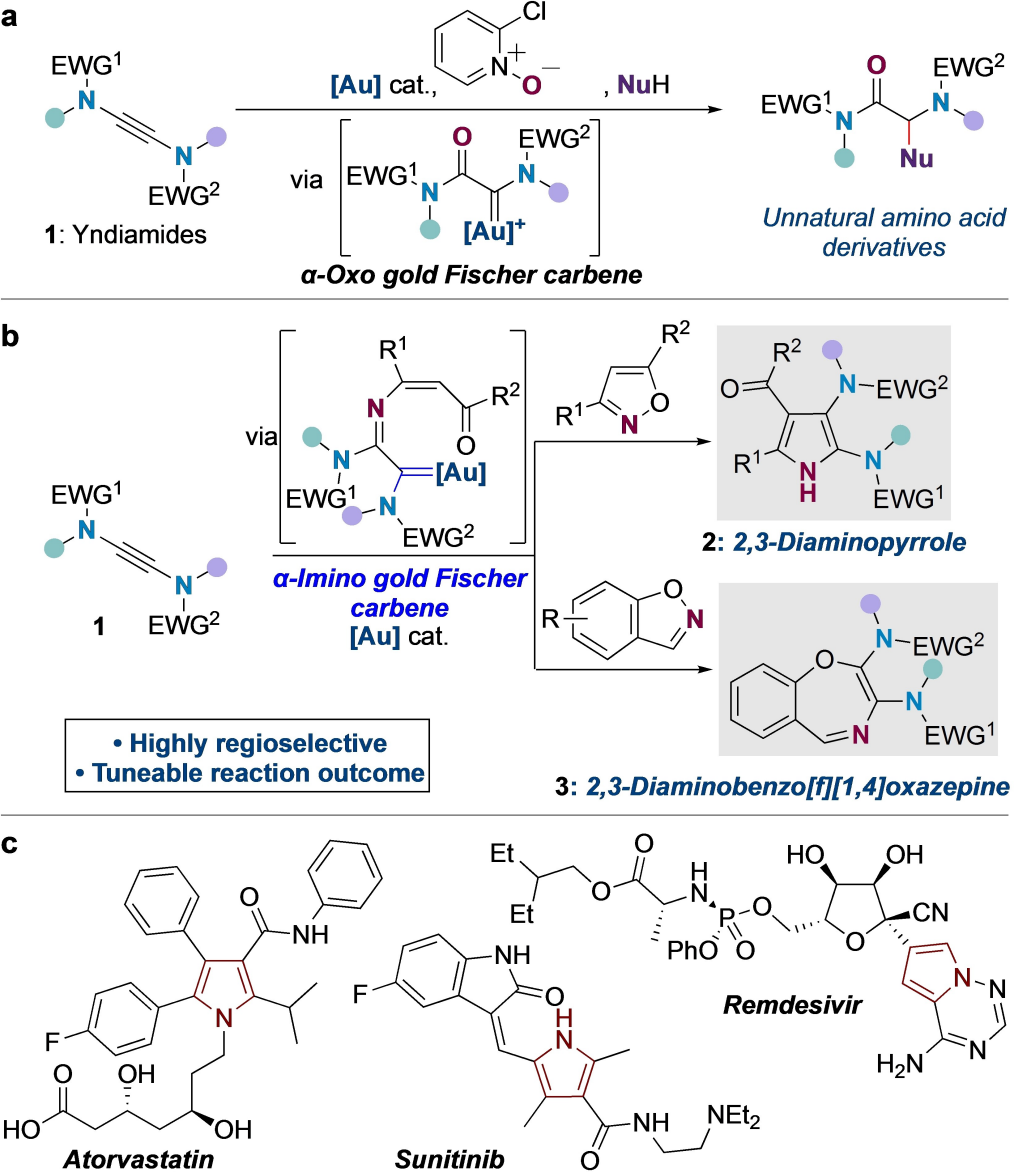
**a** Previous work: gold‐catalyzed oxidative functionalization of yndiamides with pyridine *N*‐oxides; **b This work**: Yndiamide activation via α‐imino gold Fischer carbenes; **c** Polysubstituted pyrroles in medicinal chemistry.

Au‐catalyzed functionalizations of alkynes have gained enormous importance over the past decade.^[1]^ Among those reactivities, generation of α‐oxo gold carbenes^[2]^ from unactivated alkynes^[3]^ as well as ynamides^[4]^ has received significant attention. Yndiamides (**1**, Scheme 1a),^[5]^ alkynes that feature electron‐donating nitrogen atoms activating the central alkyne at each end, offer unique reactivity profiles; these doubly‐nitrogenated motifs could serve as versatile precursors to 1,2‐diaminated products.^[6]^ In this context, our group recently reported the first gold‐catalyzed oxidative functionalization of yndiamides using pyridine *N*‐oxides[^4a^, ^4b^, ^4e^, ^4f^, ^7^] to produce amino acid derivatives via *α*‐oxo gold Fischer carbene intermediates (Scheme 1a).^[8]^

Nitrogen‐based nucleophiles such as isoxazoles,^[9]^ azides,^[10]^ sulfilimines,^[11]^ anthranils and benzisoxazoles^[12]^ have been widely applied in the gold‐catalyzed functionalization of ynamides, which proceed by regiospecific formation of a gold keteniminium ion.[^1e^, ^13^] Applications of yndiamides in such reactions are unknown, and would be additionally challenged by the *pseudo*‐symmetry of the yndiamide compared to the polarized ynamide motif. Here we describe the gold‐catalyzed formal [3+2] cyclization between yndiamides and isoxazoles to form 2,3‐diaminopyrroles (**2**, Scheme 1b) which proceeds via unprecedented *α*‐imino gold Fischer carbenes.^[14]^

Pyrroles are fundamental building blocks in medicinal chemistry, as evidenced by blockbuster drugs such as atorvastatin which feature fully‐substituted pyrrole cores (Scheme 1c);^[15]^ methods that directly synthesize highly decorated pyrroles are therefore of great interest, but access is typically challenging for pyrroles featuring heteroatom substituents.^[16]^ In addition to demonstrating regioselective cyclizations of nonsymmetric yndiamides, we also disclose a switch in reaction outcome to a formal [5+2] cyclization to form 2,3‐diaminobenzo[*f*][*1,4*]oxazepines (**3**) using benzisoxazole nucleophiles. We further describe computational investigations of the reaction mechanism and source of regioselectivity, which reveal a dynamic equilibrium of gold‐alkyne complexes prior to nucleophilic attack.

## Results and Discussion

Our studies began with a screen of gold(I) catalysts using yndiamide **1 a** and isoxazole (Table 1, entries 1–5). Successful reactions were observed in all cases, with optimal yields of product **2 a** obtained using the readily available catalysts IPrAuNTf_2_[^4f^, ^8^, ^10a^] and (ArO)_3_PAuNTf_2_[^9a^, ^9b^] (60 % and 78 % respectively, entries 1 and 2). The latter displayed heightened reactivity, reaching completion in 2 h. For some catalysts (entries 3–5), varying amounts of isomeric side product **4 a** were observed, the structure of which was determined by X‐ray crystallographic analysis.^[18]^ This isomer presumably arises from a 1,5‐formyl shift in the final aromatization step^[19]^ in the catalytic cycle (see below).^[20]^ No reaction was observed when no/non‐activated gold catalyst or AgNTf_2_ were used (entries 6–8). Elevation of the reaction temperature led to no detriment of the yield or selectivity (Entries 9–11), but proved beneficial for several cases in our study of reaction scope (see below).^[20]^ Application of the optimal conditions of entry 2 on 1.0 mmol scale afforded the desired product **2 a** in 75 % isolated yield (entry 12), albeit with a higher proportion of (separable) **4 a**.

**Table 1:** Optimization of Reaction Conditions.^[a]^

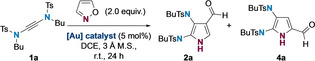				
Entry	Catalyst	T/°C	Yield (%)^[b]^	**2 a : 4 a** ^[c]^
1	IPrAuNTf_2_^[d]^	r.t.	51 (60)	>20 : 1
2^[e]^	(**Ar**O)_3_PAuNTf_2_	r.t.	77 (78)	>20 : 1
3	PPh_3_AuNTf_2_	r.t.	54	6.8 : 1
4	KAuBr_4_ ⋅ 2H_2_O^[12f]^	r.t.	55	3.6 : 1
5	PicAuCl_2_ ^[17]^	r.t.	28	7.8 : 1
6	–	r.t.	0	‐
7	(**Ar**O)_3_PAuCl	r.t.	0	‐
8	AgNTf_2_	r.t.	0	‐
9^[e]^	(**Ar**O)_3_PAuNTf_2_	40	71	>20 : 1
10^[e]^	(**Ar**O)_3_PAuNTf_2_	60	71	>20 : 1
11^[e]^	(**Ar**O)_3_PAuNTf_2_	80	72	>20 : 1
**12** ^[f]^	(**Ar**O)_3_PAuNTf_2_	r.t.	(75)	4.8 : 1

[a] Reactions conducted with **1 a** (0.05 mmol), 0.5 M. Catalysts LAuNTf_2_ were prepared *in situ* by premixing the corresponding LAuCl salt with AgNTf_2_; **Ar**=2,4‐di‐*tert*‐butylphenyl; [b] Yields determined by ^1^H NMR spectroscopy using dimethyl sulfone as internal standard; yields in parentheses are isolated yields on 0.1 mmol scale. [c] Ratio determined by ^1^H NMR spectroscopic analysis of the crude reaction mixture. [d] IPrAuNTf_2_ obtained commercially. [e] Reaction complete after 2 h. [f] Reaction conducted with **1 a** (1.0 mmol), complete after 3 h, **2 a** : **4 a** based on isolated products. DCE=1,2‐dichloroethane. IPr=1,3‐bis(2,6‐diisopropylphenyl)imidazol‐2‐ylidene. Pic=2‐pyridinecarboxylate. r.t.=room temperature. Tf=trifluoromethanesulfonyl. Ts=4‐toluenesulfonyl.

**Figure 1:**
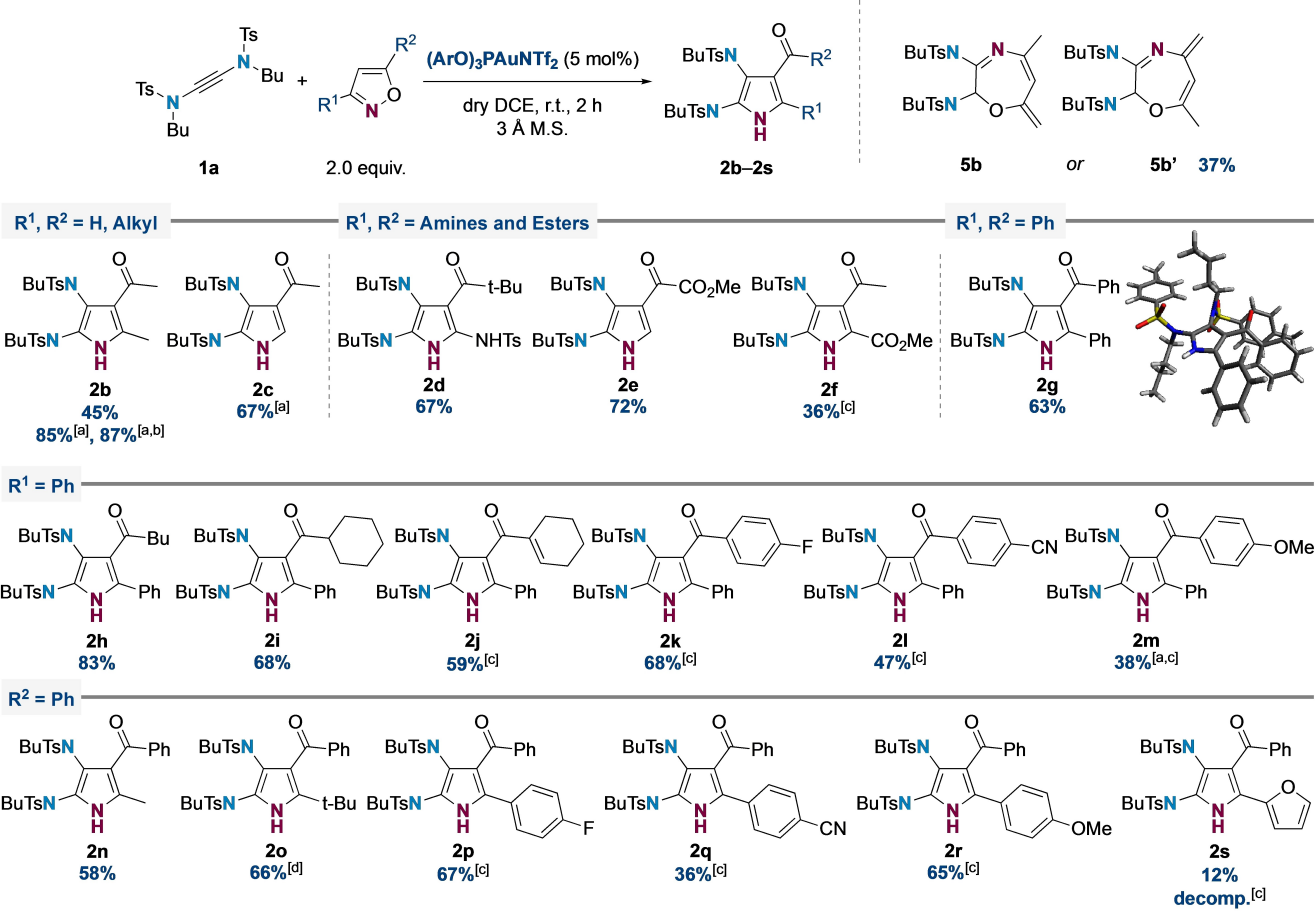
Isoxazole scope for Au(I)‐catalyzed yndiamide cyclization. Unless otherwise stated all reactions were performed on 0.1 mmol scale at room temperature with [**1 a**]=0.5 M; Yields refer to isolated yields. [a] Reaction treated with HCl (1.0 M in Et_2_O, 1.0 eq.) for 30 min after full conversion of **2 a** as indicated by TLC. [b] Reaction performed on 1.0 mmol scale and reached completion after 3 h. [c] Reaction performed at 80 °C. [d] Reaction reached completion after 4 h.

With optimized conditions in hand, the scope of the reaction was investigated using various 3,5‐disubstituted isoxazoles (Figure 1). Initial forays using 3,5‐dimethylisoxazole successfully afforded the desired product **2 b** (45 %), along with a significant amount of a side product assigned as 1,4‐oxazepine **5 b** or **5 b′** (37 %), which results from competitive 7‐membered ring formation of the proposed gold carbene intermediate (see mechanistic discussion below).^[20]^ Fortunately, we found that treatment of the crude reaction mixture with ethereal HCl triggered smooth conversion of **5 b** to **2 b**, giving an overall yield of **2 b** of 85 %. This protocol proved efficient on 1 mmol scale (0.48 g), delivering **2 b** in excellent yield (87 %). The reaction also worked well with monosubstituted 5‐methylisoxazole, affording trisubstituted pyrrole **2 c** in good yield (67 %). The use of a sulfonamidoisoxazole afforded a rarely‐seen *tris*‐aminopyrrole **2 d** in 67 % yield. Methyl esters were tolerated at either the 3‐ or 5‐position of the isoxazole, delivering **2 e** (72 %) and **2 f** (36 %), albeit the latter reaction required heating to 80 °C. This presumably reflects the reduced nucleophilicity of the nitrogen atom when adjacent to an electron‐withdrawing group.

Substituent effects were next examined when either R^1^ or R^2^ (or both) is an aryl group. **2 g** was first obtained in good yield (63 %) using 3,5‐diphenylisoxazole.^[18]^ A variety of 3‐phenyl isoxazoles were then used to examine the influence of the substituent at the 5 position. 5‐alkyl substituted isoxazoles performed well, affording **2 h** and **2 i** in good yields (83 % and 68 % respectively). The inclusion of sp^2^‐hybridized substituents at the 5‐position necessitated heating to effect conversion (80 °C), with a cyclohexenyl group delivering **2 j** in 59 % yield, and other aryl groups being moderately well tolerated (**2 k**–**2 m**). A complementary series of 5‐phenyl isoxazoles was used to examine the influence of the substituent at the 3‐position. Alkyl substitution was again well‐tolerated (**2 n**–**2 o**), with heating required for 3‐aryl isoxazoles (**2 p**–**2 r**); for these substrates, electron‐rich aryl isoxazoles proceeded more efficiently than electron‐poor. Finally, a 5‐furyl group resulted in a low yield of product **2 s** (12 %) with only decomposition observed on heating.

**Scheme 2:**
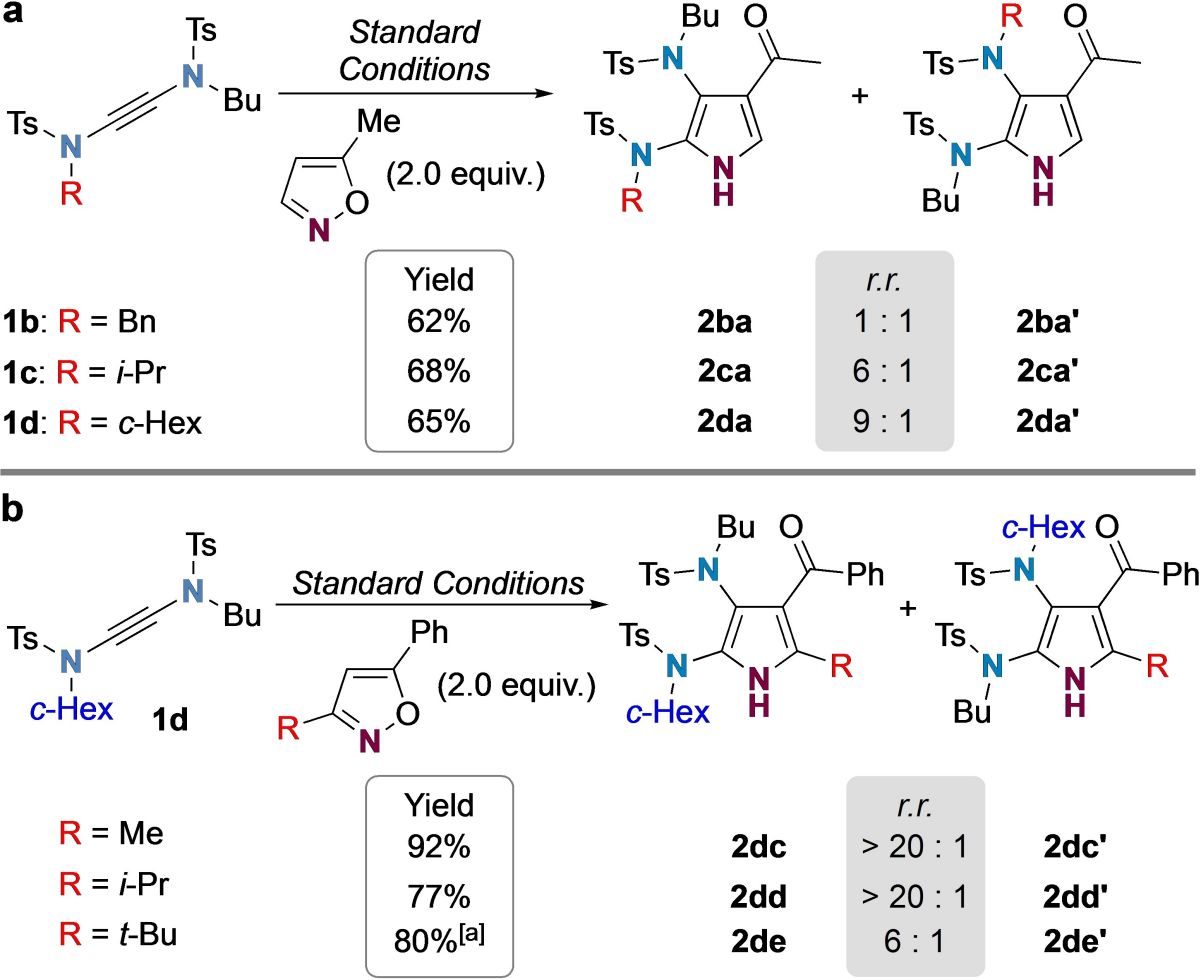
Regioselectivity in the gold‐catalyzed functionalization of unsymmetrical yndiamides with isoxazoles. Reactions were carried out using (ArO)_3_PAuNTf_2_ (5 mol%) in DCE (0.5 M) with 3 Å M.S. at room temperature for 2 h on 0.1 mmol scale. Yields are isolated yields of both regioisomers; *r.r*. determined by ^1^H NMR spectroscopic analysis of the isolated mixture of regioisomers. The identity of the major regioisomer was assigned by NOESY experiments. [a] Reaction reached completion after 24 h.

The pseudosymmetry of yndiamides presents a challenge and opportunity for regioselective functionalizations, which could enable differentiation of the pyrrole nitrogen substituents. In the oxidative functionalization of yndiamides via α‐*oxo* gold carbenes,^[8]^ we had found that steric effects afforded superior regiocontrol than electronic differentiation.^[6b]^ Pleasingly, exploration of steric effects in the regioselective functionalization of non‐symmetric yndiamides with isoxazoles revealed a similar trend (Scheme 2). A series of yndiamides (**1 b**–**1 d**) bearing varying levels of steric difference between the two nitrogen atoms were subjected to functionalization with 5‐methylisoxazole (Scheme 2a); as the steric bulk of one of the alkyl groups increased relative to the other *n‐*Bu substituent, regioselectivity increased up to 9 : 1 for yndiamide **1 d** (*n*‐Bu vs. *c‐*Hex, **2 da**/**2 da’**). This trend suggests that, as with α‐oxo‐gold carbenes, there is a preference for coordination of the less‐hindered alkyne carbon of the yndiamide to the bulky gold complex.

The ability of **1 d** to undergo regioselective cyclization with isoxazoles featuring increasing steric hindrance adjacent to the nucleophilic nitrogen atom was next studied, as the isoxazole is required to attack the yndiamide at the ′bulkier′ end (i. e. at the cyclohexyl‐substituted terminus). 5‐phenyl substituted isoxazoles with differently sized 3‐alkyl substituents were tested for regioselectivity (Scheme 2b). To our delight, excellent selectivity was observed for a methyl‐ and *iso‐*propyl substituent (R=Me, **2 dc**/**2 dc’**, *r.r*.>20 : 1; R=*i*‐Pr, **2 dd**/**2 dd’**, *r.r*.>20 : 1). Even the introduction of a *t*‐Bu group (which greatly reduced the reaction rate) maintained respectable regioselectivity (**2 de**/**2 de’**, *r.r*. 6 : 1). This underlines the dominance of the steric demands of the gold phosphite complex, reliably leading to the pyrrole product featuring the bulkier nitrogen substituent at the 2‐position.

**Scheme 3:**
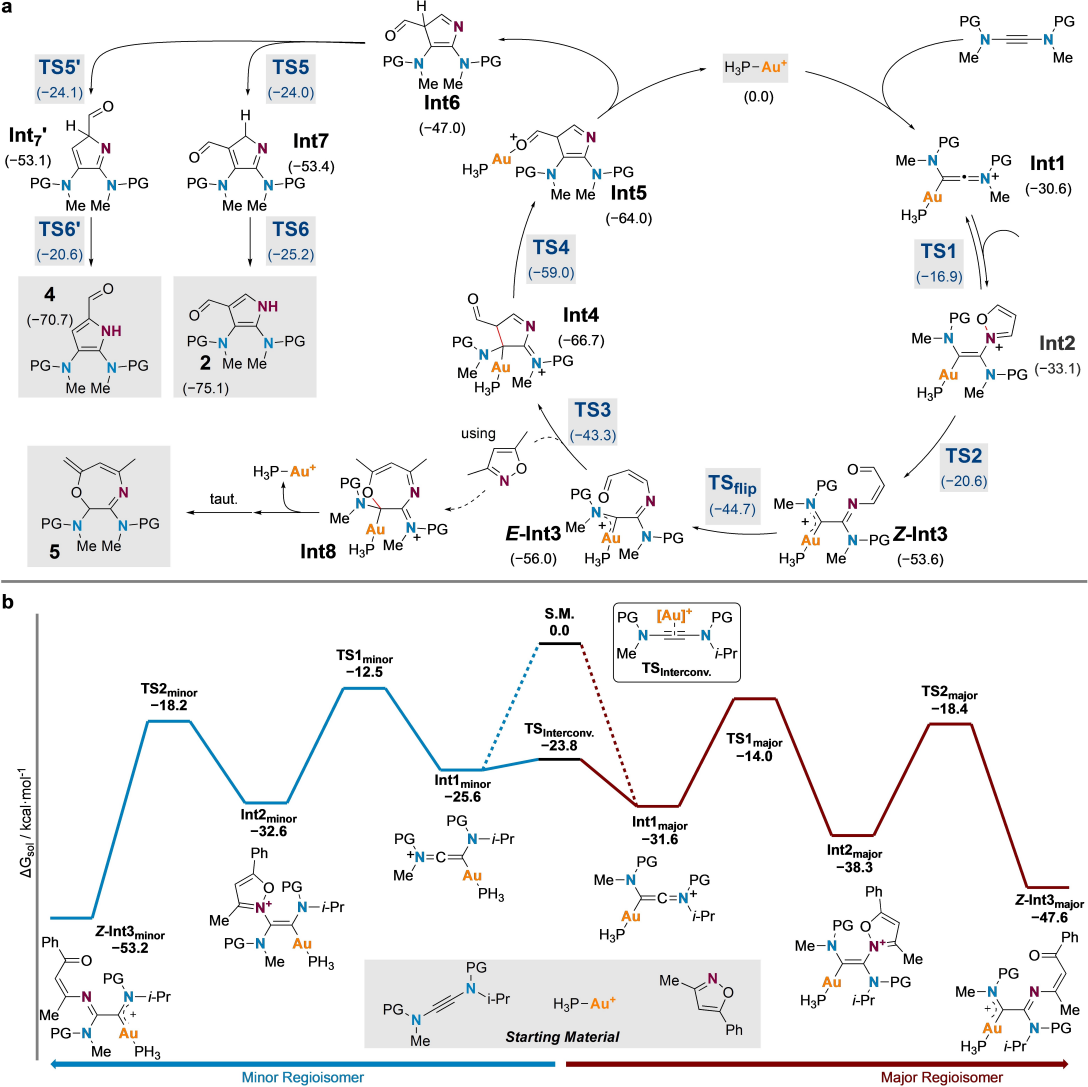
**a** Proposed catalytic cycle for symmetric yndiamide. PG=Benzenesulfonyl. **b** Reaction energy profiles of regioselectivity determining steps for unsymmetric yndiamide. PG=Benzenesulfonyl. Relative Gibbs free energies (ΔGsol, in kcal mol‐1) of key intermediates and transition states were computed at CPCM(1,2‐dichloroethane)‐DLPNO‐CCSD(T)/def2‐TZVPP//IEFPCM(1,2‐dichloroethane)‐M06/6‐31+g(d)+SDD(Au) level of theory at 298 K.

DFT calculations were next employed to explore the mechanism of this yndiamide oxidative functionalization (Scheme 3a).[^9a^, ^12g^, ^12k^, ^21^] The reaction was modelled using [AuPH_3_]^+^ as catalyst, and benzenesulfonyl groups instead of toluenesulfonyl groups on the yndiamide. The former approximation has been used for related calculations using ynamides;^[9a]^ attempts to use P(OMe)_3_ or P(OPh)_3_ as ligands were unsuccessful due to the higher complexity of these systems. Initial reaction of the yndiamide with the active gold species forms the keteniminium‐gold complex **Int1**. Similar to the findings of Ye *et al*.,^[9a]^ this process is energetically favored (ΔG_sol_=−30.6 kcal mol^−1^), although a transition state could not be located. **Int1** then undergoes nucleophilic attack by the isoxazole (here we present the most energetically favorable attack, see the Supporting Information for an alternative isoxazole attack from a different relative orientation), generating a triply nitrogen‐substituted vinyl gold complex **Int2**, with an activation barrier of 13.7 kcal mol^−1^ (**TS1**). **Int2** is close in energy to **Int1** (ΔG_sol_=−2.5 kcal mol^−1^), suggesting that these species should be in equilibrium. Subsequent isoxazole ring opening gives the α*‐*imino gold Fischer carbene intermediate ***Z*** **‐Int3** with a moderate activation energy (**TS2**, ΔG^≠^ **=**12.5 kcal mol^−1^). This step is significantly exergonic (ΔG_sol_=−20.6 kcal mol^−1^) due to the additional stabilization afforded by the Fischer carbene nitrogen atom, rendering the transformation irreversible. The imine geometry of ***Z*** **‐Int3** can readily invert to the slightly more stable ***E*** **‐Int3** (**TS_flip_**, ΔG^≠^ **=**9.0 kcal mol^−1^), which then undergoes a 4π Nazarov‐type cyclization^[22]^ to give the cyclic intermediate **Int4** (**TS3**, ΔG^≠^ **=**12.7 kcal mol^−1^). The formyl group in **Int4** appears to be beneficial for the subsequent deauration, delivering the acyl‐gold complex **Int5**, which releases the gold complex for the next cycle. While aromatization of **Int6** may be mediated by trace Brønsted acid, it could also undergo either two consecutive 1,5‐hydride shifts (through **TS5**, ΔG^≠^ **=**23.0 kcal mol^−1^ and **TS6**, ΔG^≠^ **=**28.2 kcal mol^−1^) to give the major product **2**, or a 1,5‐formyl shift/hydride shift sequence (through **TS5’**, ΔG^≠^ **=**22.9 kcal mol^−1^ and **TS6’**, ΔG^≠^ **=**32.5 kcal mol^−1^) to give the minor product **4**. The identification of a 1,4‐oxazepine side product in certain cases (e. g. **5**, using 3,5‐dimethylisoxaozle) may be explained by an alternative 7‐membered ring formation pathway which diverts from the main catalytic cycle at ***E*** **‐Int3**: Instead of undergoing Nazarov cyclization, a 6π electrocyclization[^12b^, ^12c^] could take place to afford **Int8**, which can undergo deauration and tautomerization to give oxazepine **5**.

Having gained insight into the catalytic cycle for symmetrical yndiamides, we next aimed to understand the regioselectivity observed using non‐symmetric substrates. As mentioned above, regioselectivity is likely controlled at or before the rate‐determining step ***Z*** **‐Int3**, and as such additional reaction energy profiles were calculated up to this point for an yndiamide featuring methyl and isopropyl substituents (Scheme 3b). This unsymmetrical yndiamide could initially react with the active gold catalyst to form two regioisomeric keteniminium‐gold complexes (**Int1_major_** and **Int1_minor_**). The formation of these complexes is highly exergonic and barrierless, and hence this process is likely irreversible. However, we were able to identify a low barrier π‐complex transition state **TS_interconv_**_._ that enables the interconversion of **Int1_major_** and **Int1_minor_** (ΔG^≠^=1.8 kcal mol^−1^, ΔG_sol_=−6.0 kcal mol^−1^), which suggests the subsequent regioselectivity‐determining step operates under a Curtin‐Hammett scenario. With sterically undemanding isoxazole nucleophiles (e. g. 3‐methyl‐5‐phenylisoxazole as shown in Scheme 3b), attack on **Int1_major_** via **TS1_major_** (ΔG^≠^=17.6 kcal mol^−1^) has a lower barrier than that via **TS1_minor_** (ΔΔG^≠^ (**TS1_major_**/**TS1_minor_**)=1.5 kcal mol^−1^). **Int2_major_** is hence formed predominantly and eventually produces the major product regioisomer. As the bulkiness of the isoxazole nucleophile increases, the energy difference between **TS1_major_** and **TS1_minor_** would be expected to diminish due to increased steric repulsion between the isoxazole and the bulkier nitrogen substituent, leading to the lower regioselectivity observed experimentally.

**Scheme 4:**
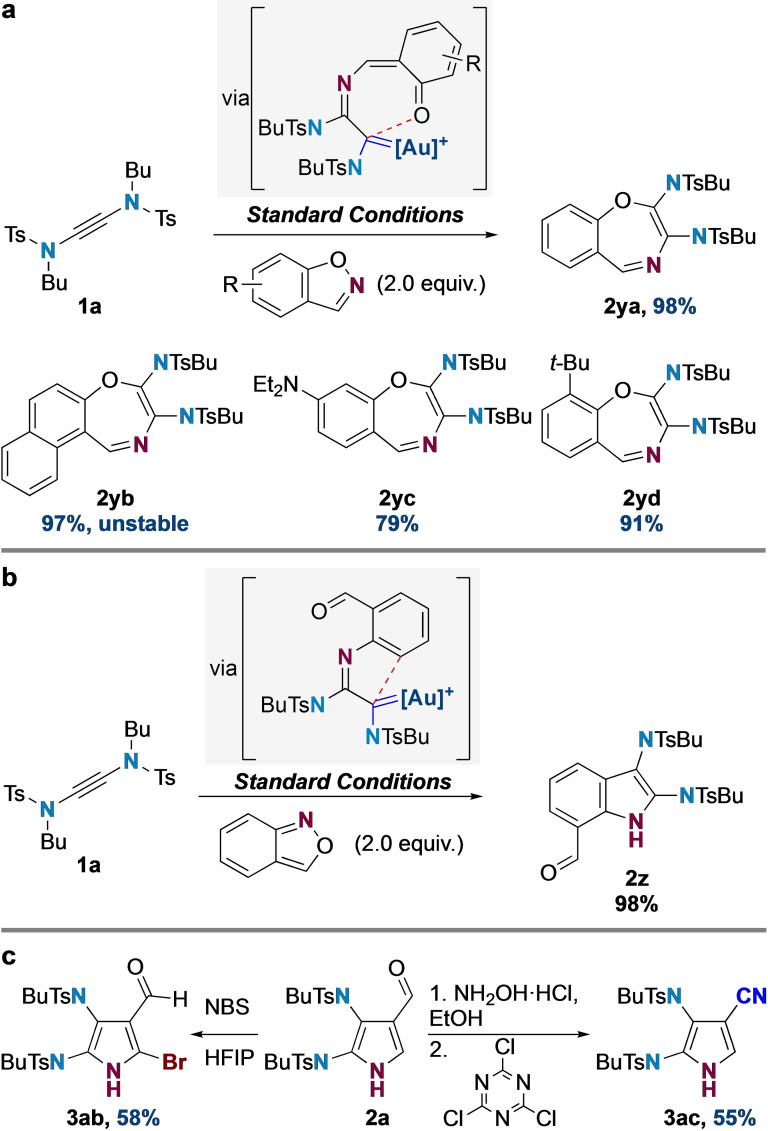
Reactivity of yndiamides with **a** benzisoxazoles and **b** anthranil. Standard conditions: (ArO)_3_PAuNTf_2_ (5 mmol%), DCE (0.5 M), 3 Å M.S., room temperature, 2 h, 0.10 mmol scale; yields refer to isolated yields. **c** 2,3‐Diaminopyrrole derivatizations.

Finally, we questioned whether the choice between 4π‐ and 6π‐electrocyclization as discussed above could be controlled by the use of a benzisoxazole nucleophile[^12b^, ^12c^] rather than isoxazole (Scheme 4a). In this case, 6π‐electrocyclization would enable rearomatization of the benzene ring from the intermediate gold carbene, whereas 4π‐electrocyclization would lead to a spirocyclic product that cannot easily aromatize. In the event, reactions with benzisoxazoles indeed led to the formation of a series of diamino‐1,4‐benzoxazepine 6π products **2 ya**–**2 yd** in excellent yields (79–98 %). In a complementary fashion, the reaction with anthranil produced exclusively the 2,3‐diamino substituted formyl indole **2 z** in nearly quantitative yield (98 %, Scheme 4b). Finally, to illustrate the potential of the 2,3‐diaminopyrrole products to undergo functionalizations, we briefly explored the derivatization of pyrrole **2 a** (Scheme 4c). Electrophilic bromination^[23]^ of **2 a** was successful, affording **3 ab** in good yield, introducing a useful halogen handle for further transformations. Additionally, the formyl group in **2 a** could be transformed into an oxime, subsequent elimination^[24]^ of which produced cyanopyrrole 3ac in 55 % yield.

## Conclusions

In conclusion, we have developed a gold‐catalyzed formal [3+2] cycloaddition between yndiamides and isoxazoles to synthesize valuable but typically hard‐to‐access 2,3‐diaminopyrroles. Excellent regioselectivity was established for non‐symmetric yndiamides by steric differentiation between the two yndiamide alkyl substituents, favoring complexation of the gold(I) catalyst at the less‐hindered terminus of the yndiamide alkyne. DFT calculations suggest a Curtin‐Hammett scenario where a gold π‐complex‐like transition state was crucial for interconversion of alkenyl‐gold complexes, and hence in determining the regiochemical outcome. In addition to the observation of an unusual 1,2‐formyl migration product, the formation of an oxazepine product enabled the implementation of a switch in ring‐size selectivity using benzisoxazole nucleophiles, while anthranil provided access to a 2,3‐diaminoindole. Given the rare nature of these di‐aminated heterocycles, this chemistry could be of significant utility for the synthesis of medicinally relevant scaffolds, and adds to the growing body of chemo‐ and regioselective transformations of yndiamides.

## Experimental Section

**General Procedure for Au‐Catalyzed Cyclization between Yndiamides and Isoxazoles**: To a heat gun‐dried vial containing a stirrer bar and 3 Å molecular sieves was added the yndiamide (0.1 mmol. 1.0 equiv.). The vial was purged under high vacuum for 30 s and backfilled with argon; this cycle was repeated twice more. The vial was then taken into glovebox where chloro[tris(2,4‐di‐*tert*‐butylphenyl)phosphite]gold(I) (5 mol%) and silver bis(trifluoromethanesulfonyl)imide (5 mol%) were added. After the vial was removed from the glovebox, anhydrous 1,2‐dichloroethane (0.2 mL, 0.5 M) and the isoxazole (0.2 mmol, 2.0 equiv.) were added (the isoxazole was added to the vial together with yndiamide before moving into the glovebox if it is non‐volatile). The reaction mixture was stirred at room temperature (or the stated temperature, see the Supporting Information) under an argon atmosphere in the dark until the reaction reached completion as judged by TLC. The reaction mixture was concentrated *in vacuo*, and the residue was purified by column chromatography (EtOAc/pentane eluant) to give the pyrrole product.

## Supporting Information

Experimental procedures, additional discussion, copies of ^1^H and ^13^C NMR spectra (.pdf), crystallographic data (.cif). The authors have cited additional references within the Supporting Information.[^25^, ^26^, ^27^, ^28^, ^29^, ^30^, ^31^, ^32^, ^33^, ^34^, ^35^, ^36^, ^37^, ^38^, ^39^, ^40^, ^41^, ^42^, ^43^, ^44^, ^45^, ^46^, ^47^, ^48^, ^49^, ^50^, ^51^, ^52^, ^53^, ^54^, ^55^, ^56^, ^57^, ^58^, ^59^, ^60^]

## Conflict of interest

The authors declare no conflict of interest.

1

## Supporting information

As a service to our authors and readers, this journal provides supporting information supplied by the authors. Such materials are peer reviewed and may be re‐organized for online delivery, but are not copy‐edited or typeset. Technical support issues arising from supporting information (other than missing files) should be addressed to the authors.

Supporting Information

## Data Availability

The data that support the findings of this study are available in the supplementary material of this article. Deposition Numbers 2213976 (for **2 g**), 2213977 (for **4 a**) contain the supplementary crystallographic data for this paper. These data are provided free of charge by the joint Cambridge Crystallographic Data Centre and Fachinformationszentrum Karlsruhe Access Structures service.
