# Supplementary material for: Gold‐Catalyzed Cyclization of Yndiamides with Isoxazoles via α‐Imino Gold Fischer Carbenes
Source: Chemistry. 2023 Oct 25;29(70):e202302821. doi: 10.1002/chem.202302821 (PMC10947298; doi:10.1002/chem.202302821)
Supplement: Supplementary file 1 — Supporting Information [file CHEM-29-0-s001.pdf]

Chemistry–A European Journal

Supporting Information

Gold-Catalyzed Cyclization of Yndiamides with Isoxazoles via α-Imino Gold Fischer Carbenes

Zixuan Tong, Philip J. Smith, Helena D. Pickford, Kirsten E. Christensen, and
Edward A. Anderson*

1. Materials and Physical Measurements……………………………………………………..… S2

2. General Procedures……………………………………………………...…………….….. S3

3. Additional Experimental Results……………………………………….………………... S5

4. Characterization of Synthesized Compounds…………………………………………….. S16

5. Density Functional Theory (DFT) Calculations.……………………………………...…... S50

6. X-Ray Crystallography ………………………….……………………………………..….. S79

7. NMR Spectra of Novel Compounds ……………………………………...…....…..……S84

8. References for Supporting Information …………………...……………………..……...……... S154

1. Materials and Physical Measurements

Reagents and Solvents: All reagents were purchased from commercial suppliers and used as received unless stated otherwise. Anhydrous dichloromethane (DCM) and THF were obtained from solvent dispenser units having been passed through an activated alumina column under argon. Anhydrous 1,2-dichloroethane (DCE) was prepared by refluxing commercially purchased non-anhydrous DCE over CaH_2 followed by distillation onto 3 Å molecular sieves under argon.

NMR Spectroscopy: ^{1}H, ^{13}C, ^{19}F, and ^{31}P NMR spectra were obtained by a Bruker AVIII HD 400, a Bruker NEO 400, a Bruker AVIII HD 500, or a Bruker AVIII HD 600 instrument at room temperature using TOPSPIN software. Assignments were determined either on the basis of unambiguous chemical shift/coupling patterns, or from COSY, HSQC, HMBC and/or NOESY experiments. Peak multiplicities are defined as: s = singlet, d = doublet, t = triplet, q = quartet, quin = quintet, sext = sextet, hept=heptet, m = multiplet, br s = broad singlet. Coupling constants (*J*) are reported to the nearest 0.1 Hz.

Infrared Spectroscopy: All infrared spectra were recorded by a Bruker Tensor 27 FT-IR spectrometer on a sample thin film prepared on a diamond ATR module. Maximum absorptions (ν_{max}) are reported in wavenumbers (cm^{-1}).

Mass Spectrometry: High resolution mass spectra (HRMS) were measured by a Bruker Microflex TOF spectrometer using electrospray ionization (ESI). Mass-to-charge ratios are calculated to 4 decimal places from the molecular formula and all reported experimental results are within 5 ppm difference compared to calculated values.

Melting Points: Melting points were measured on a Griffin melting point apparatus and are reported without correction.

2 General Procedures

2.1 General Procedures for Synthesis of Yndiamides

All yndiamides and related coupling partners were synthesized according to a protocol reported by our group previously (Scheme S1).[1]

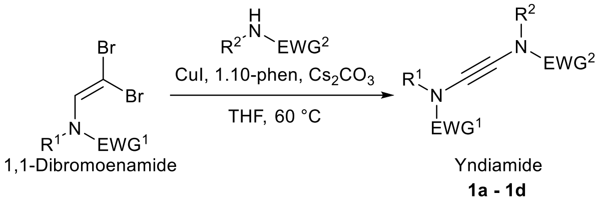

Scheme S1. Synthesis of yndiamides.

To a heat gun-dried round-bottom flask equipped with a stirrer bar was added the corresponding sulfonamide (1.0 eq.), 1,1-dibromoenamide (1.1 eq.)[1], and 1,10-phenanthroline (40 mol%). The flask was evacuated under high vacuum for 10 min and backfilled with argon (the vacuum cycle was repeated twice more). Then the flask was placed inside the glovebox where CuI (20 mol%) and Cs_2CO_3 (3.0 eq.) were added. The flask was removed from the glovebox and, again, purged under high vacuum for 30 s and then backfilled with argon. Anhydrous THF (3.3 mL $mmol^{-1}$ of sulfonamide substrate) was added and the mixture was stirred at 60 °C until completion by TLC. The reaction mixture was cooled to room temperature and filtered through a Celite® pad (eluted with EtOAc). The filtrate was concentrated *in vacuo* and purified by chromatography (column conditions described per compound) to afford the yndiamide. Yndiamides **1a** and **1c** were prepared using the same method and fully characterized in our previous work.[2]

2.2 General Procedure for Synthesis of 3,5-Substituted Isoxazoles

According to a modified literature procedure.[3] To a solution of hydroxylamine hydrochloride (1.05 eq.) in 1:1 *t*-BuOH/H_2O (0.25 M) was added the corresponding aldehyde (1.00 eq.) and NaOH (1.05 eq.). The reaction was stirred until completion by TLC. Chloramine T trihydrate (1.05 eq.) was then added in small portions, followed by copper sulfate pentahydrate (3 mol%), copper turning (1 pc, *ca.* 50 mg) and the corresponding alkyne (1.05 eq.). The reaction mixture was adjusted to ~ pH 6 by adding 1 M NaOH solution, and then it was stirred at room temperature until completion. Upon completion, the reaction was poured onto ice, and dilute aqueous ammonia was added to remove all the copper salts. The crude product was collected by filtration and further purified by recrystallization or column chromatography (described per compound) to give the final product.

2.3 General Procedures for Au-Catalyzed Cyclization between Yndiamides and Isoxazoles

To a heat gun dried vial containing a stirrer bar and 3 Å molecular sieves were added the corresponding yndiamide (1.0 eq.). The vial was purged under high vacuum for 30 s and backfilled with argon (this vacuum cycle was repeated twice more). The vial was then moved into glovebox where chloro[tris(2,4-di-tert-butylphenyl)phosphite]gold(I) (5 mol%) and silver bis(trifluoromethanesulfonyl)imide (5 mol%) were added. DCE (anhy., 0.5 M) and the corresponding isoxazole (2.0 eq.) were added after the vial was removed from the glovebox (the isoxazole reagent was added to the vial together with yndiamide before moving into the glovebox if it is non-volatile). The reaction mixture was stirred at the stated temperature under argon atmosphere in dark until completion by TLC. Upon completion, unless otherwise stated, the reaction mixture was concentrated *in vacuo* and purified by column chromatography (column conditions described per compound) to give the final product.

2.4 General Procedure for Synthesis of Benzo[d]isoxazoles

Step 1**:** In a round-bottom flask was mixed the corresponding salicylaldehyde (1.0 eq.), hydroxylamine hydrochloride (1.5 eq.) and $NaHCO_3$ (2.0 eq.) in EtOH (0.5 M). The reaction mixture was stirred at room temperature until completion by TLC. Upon completion, the reaction mixture was diluted with water and extracted three times with DCM. The organic layer was combined, dried over Na_2SO_4, and concentrated *in vacuo* to give the crude salicylaldoxime which was used in the next step without further purification.

Step 2: *Modified from a literature procedure.*[4] To a heat gun dried flask was added the crude salicylaldoxime (1.00 eq.) prepared in the previous step and PPh_3 (1.25 eq.). The flask was purged under high vacuum for 30 s and backfilled with argon (this cycle was repeated twice more). Anhydrous THF (0.05 M) was added to dissolve the starting material, and the solution was cooled to 0 °C on an ice bath. DIAD (1.20 eq.) was added slowly to the solution over 1 h. The reaction mixture was then warmed to room temperature, concentrated *in vacuo* and purified by column chromatography (column conditions described per reaction) to afford the final product.

3 Additional Experimental Results

3.1 Optimization of Conditions

Table S1. Optimization of conditions.[a]

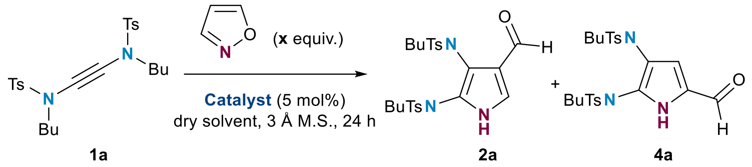

Entry	Catalyst[b]	x	Solvent[c]	[1a]	Temp.	Yield[d]	2a:4a[e]
1	$IPrAuNTf_2$	2.0	DCE	0.50 M	r.t.	51% (60%)	> 20:1
2	$IPrAuNTf_2$	2.0	DCM	0.50 M	r.t.	51%	14:1
3	$IPrAuNTf_2$	2.0	MeCN	0.50 M	r.t.	trace	-
4	$IPrAuNTf_2$	2.0	DMSO	0.50 M	r.t.	0%	-
5	$IPrAuNTf_2$	2.0	DMF	0.50 M	r.t.	0%	-
6	$IPrAuNTf_2$	2.0	PhMe	0.50 M	r.t.	19%	> 20:1
7	$IPrAuNTf_2$	2.0	THF	0.50 M	r.t.	6%	> 20:1
8	$IPrAuNTf_2$	2.0	EDB	0.50 M	r.t.	47%	> 20:1
9	$IPrAuNTf_2$	2.0	$CHCl_3$	0.50 M	r.t.	47%	> 20:1
10	$IPrAuNTf_2$	2.0	MeOH	0.50 M	r.t.	0%	-
11	$IPrAuNTf_2$	2.0	Neat[f]	0.50 M	r.t.	28%	> 20:1
12	$IPrAuNTf_2$	1.1	DCE	0.50 M	r.t.	41%	17:1
13	$IPrAuNTf_2$	1.5	DCE	0.50 M	r.t.	44%	12:1
14	$IPrAuNTf_2$	10	DCE	0.50 M	r.t.	60%	12:1
15	$IPrAuNTf_2$	2.0	DCE	0.25 M	r.t.	47%	13:1
16	$IPrAuNTf_2$	2.0	DCE	0.125 M	r.t.	44%	11:1
17	$IPrAuNTf_2$	2.0	DCE	0.05 M	r.t.	41%	10:1
18	$AuCl_3$[g]	2.0	DCE	0.50 M	r.t.	27%	4:1
19	$dppe(AuNTf_2)_2$[h]	2.0	DCE	0.50 M	r.t.	51%	5:1
20	(**Ar**O)$_3$PAuNTf$_2$[g]	2.0	DCE	0.50 M	r.t.	77% (78%)	> 20:1
21	PPh_3AuNTf_2	2.0	DCE	0.50 M	r.t.	54%	6.8:1
22	$KAuBr_4 \cdot 2H_2O$	2.0	DCE	0.50 M	r.t.	55%	3.6:1
23	$PicAuCl_2$	2.0	DCE	0.50 M	r.t.	28%	7.8:1
24	-	2.0	DCE	0.50 M	r.t.	0%	-
25	(**Ar**O)$_3$PAuCl	2.0	DCE	0.50 M	r.t.	0%	-
26	$AgNTf_2$	2.0	DCE	0.50 M	r.t.	0%	-
27	(**Ar**O)$_3$PAuNTf$_2$[g]	2.0	DCE	0.50 M	40 °C	71%	> 20:1
28	(**Ar**O)$_3$PAuNTf$_2$[g]	2.0	DCE	0.50 M	60 °C	71%	> 20:1
29	(**Ar**O)$_3$PAuNTf$_2$[g]	2.0	DCE	0.50 M	80 °C	72%	> 20:1

[a] Reactions conducted with **1a** (0.05 mmol); [b] Catalysts $LAuNTf_2$ were prepared *in situ* by premixing the corresponding LAuCl pre-catalyst and $AgNTf_2$ unless stated otherwise. dppe = 1,2-bis(diphenylphosphino)ethane. $IPrAuNTf_2$ was obtained commercially. **Ar** = 2,4-di-tert-butylphenyl. Pic = 2-pyridinecarboxylate; [c] DCE = 1,2-dichloroethane. EDB = 1,2-dibromoethane. [d] Yields determined by ^{1}H NMR spectroscopy using dimethyl sulfone as internal standard; yields in parentheses are isolated yields on 0.1 mmol scale. [e] Ratio determined by ^{1}H NMR spectroscopic analysis of the

crude reaction mixture; [f] 0.1 mL of isoxazole was added as solvent. [g] Reaction completed after 2 h; [h] 2.5 mol% of the catalyst was used.

The above-tabled is a comprehensive list of reaction conditions we screened for this transformation. Given that our first attempt with $IPrAuNTf_2$ catalyst revealed successful (Table S1, entry 1), we carrier on using this catalyst to test different solvents (Table S1, entries 2 – 11), isoxazole loadings (Table S1, entries 12 – 14) and concentrations (Table S1, entries 15 – 17). Halogenated solvents are generally good for this reaction, with DCE and DCM being the two best performing ones. Considering the potential to conducting the reaction at higher temperatures, we chose to carry out our studies using DCE as solvent. Increasing isoxazole loading turned out to be marginally beneficial to the overall yield, while dilution appears to be detrimental to the formation of **2a**, as evidenced by decreased yields and **2a**-to-**4a** ratio. A subsequent testing of various commonly used gold catalysts (Table S1, entries 18 – 26) showed that $(ArO_3)PAuNTf_2$ is the optimal catalyst for this transformation and there's no significant improvement in yield with increased temperatures (Table S1, entries 27 – 29).

3.2 Unsuccessful Isoxazole Examples

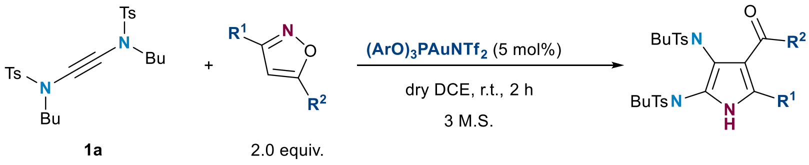

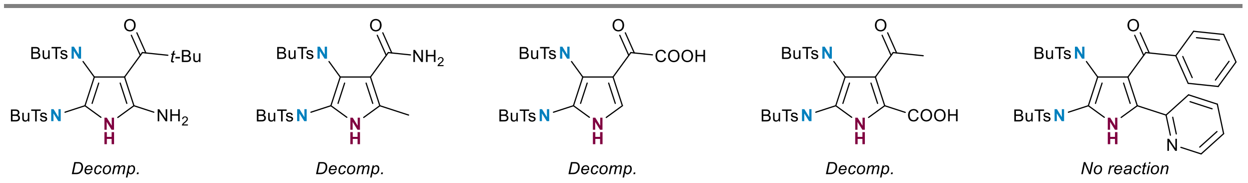

*Figure S1. Unsuccessful examples in our scope study. All reactions performed on 0.1 mmol scale with [**1a**] = 0.50 M.*

In addition to the isoxazole scope we presented in Figure 1, the above-listed are unsuccessful isoxazole examples in our scope study. Functional groups with active hydrogen atoms (*e.g.*, -COOH and -NH_2) would cause complete decomposition of **1a**, presumably due to the presence of the competitive Brønsted acid addition across the yndiamide triple bond followed by subsequent decomposition of the adduct. The presence of a strong coordinating group (*e.g.*, pyridine ring) would also shut down the reactivity due to pyridine coordinate to the active gold complex.

3.3 1,4-Oxazepine Side-product Formation

Based on the precedented [5+2] cyclization pathways in gold-catalyzed functionalization of ynamides with isoxazoles[5], we proposed the structure of the isolable side-product **5b**, its formation pathway and the acid-mediated isomerization pathway to form the desired pyrrole product **2b** (Scheme S2).

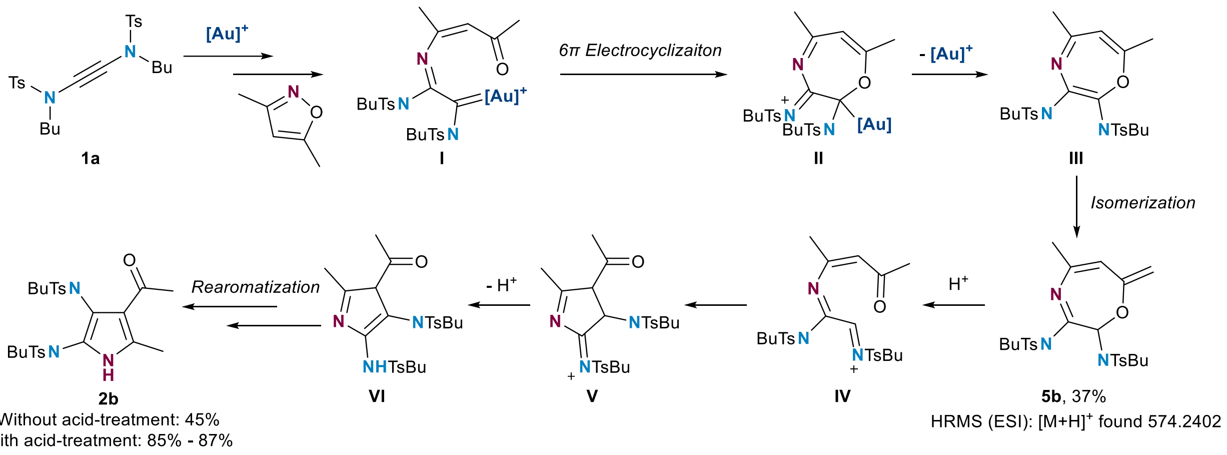

*Scheme S2. Proposed structure of **5b**, its formation pathway and the acid-mediated isomerization pathway to form **2b**.*

Treatment of yndiamide **1a** with gold catalyst and 3,5-dimethylisoxazole generated the α-imino carbene intermediate **I.** A 6π-electrocyclization then formed intermediate **II** which then undergoes deauration to give a 1,4-oxazepine product **III**. However, as suggested by ^{1}H NMR (see later full characterization of **5b**), it is its isomer **5b** that was isolated. HRMS also confirmed its chemical formula. Treatment with ethereal HCl triggered a ring-opening which generated an iminium ion intermediate **IV**. **IV** could undergo a 4π-electrocyclization again to afford **V**. Subsequent elimination of H^+ lead to intermediate **VI** which rearomatized to the final isolated product **2b**.

3.4 Regioselectivity Studies

a)

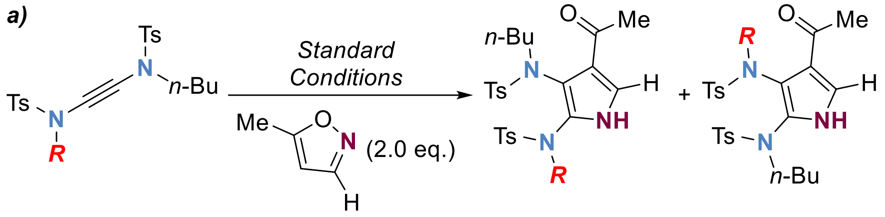

	Yield		r.r.	
**1b**: ***R*** = Bn	62%	**2ba**	1 : 1	**2ba'**
**1c**: ***R*** = *i*-Pr	68%	**2ca**	6 : 1	**2ca'**
**1d**: ***R*** = *c*-Hex[a]	65%	**2da**	10 : 1	**2da'**

b)

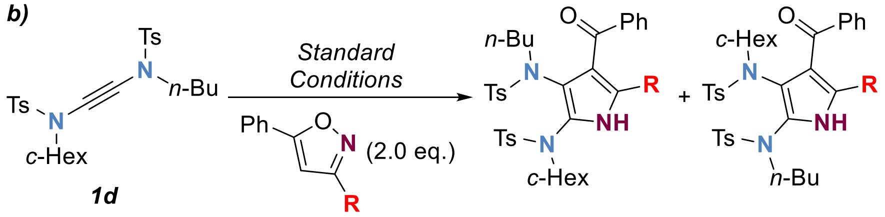

	Yield		r.r.	
R = H	Decomp.	**2db**	-	**2db'**
R = Me	92%	**2dc**	> 20 : 1	**2dc'**
R = *i*-Pr	77%	**2dd**	> 20 : 1	**2dd'**
R = *t*-Bu[a]	80%	**2de**	6 : 1	**2de'**

Scheme S3. Regioselectivity in gold-catalyzed functionalization of unsymmetrical yndiamides with isoxazoles. Standard conditions: $(ArO)_3PAuNTf_2$ (5 mmol%), DCE (anhy., 0.5 M), 3 Å M.S., r.t., 2 h. All reactions are done at 0.1 mmol scale; yields correspond to isolated yield of both regioisomers together; r.r. determined by ^{1}H NMR experiment; the identity of the major regioisomer was assigned based on NOESY experiment. Decomp. denotes decomposition of the yndiamide and no products were isolated. [a] Reactions complete after 24 h.

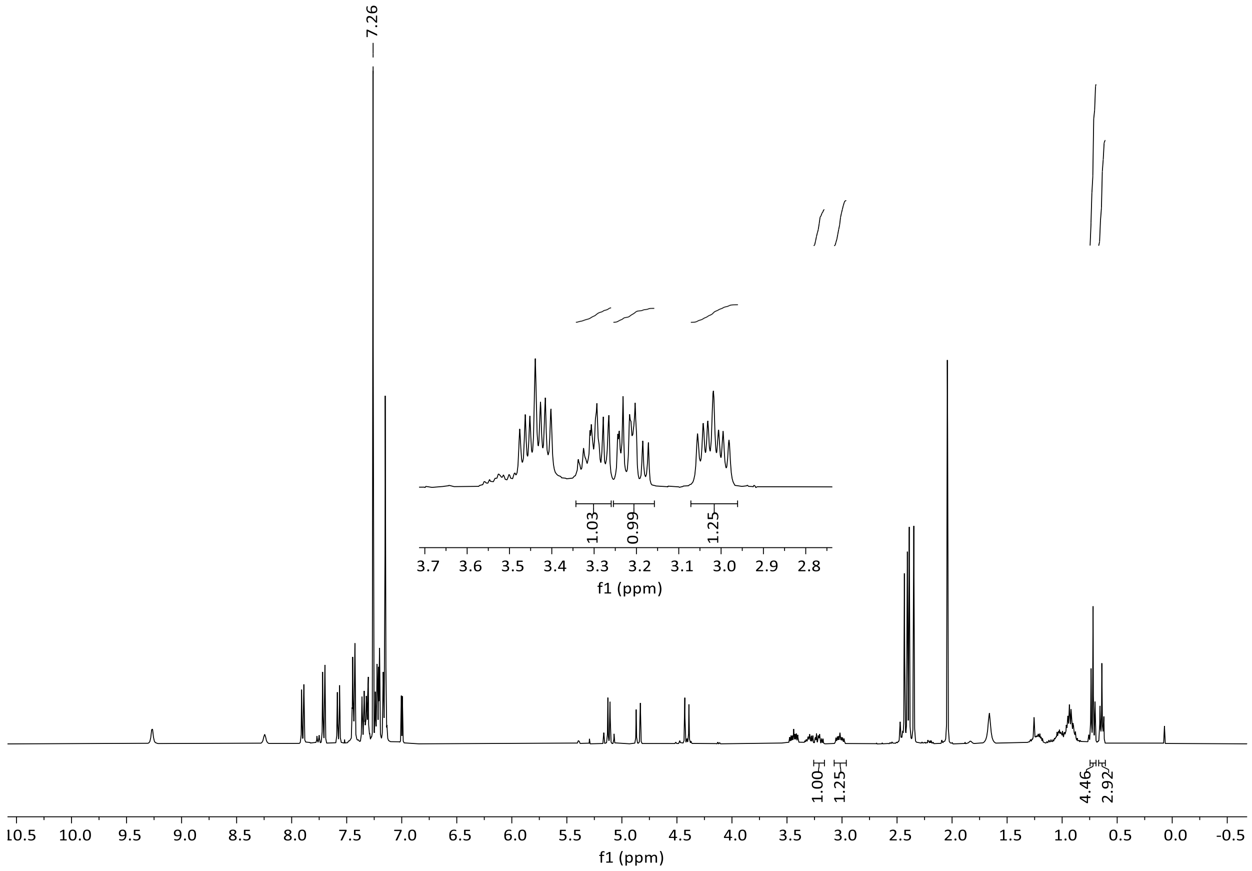

Figure S2. *^{1}H NMR (400 MHz, $CDCl_3$) of* ***2ba/2ba'*** *mixture. Integrated peak: Bu*CH_2*, showing that* ***2ba*****:*****2ba'*** *≈ 1:1.* **HRMS** (ESI) m/z: [M + H]+ Calcd for $C_{31}H_{36}N_3O_5S_2$ 594.2091.; Found 594.2084.

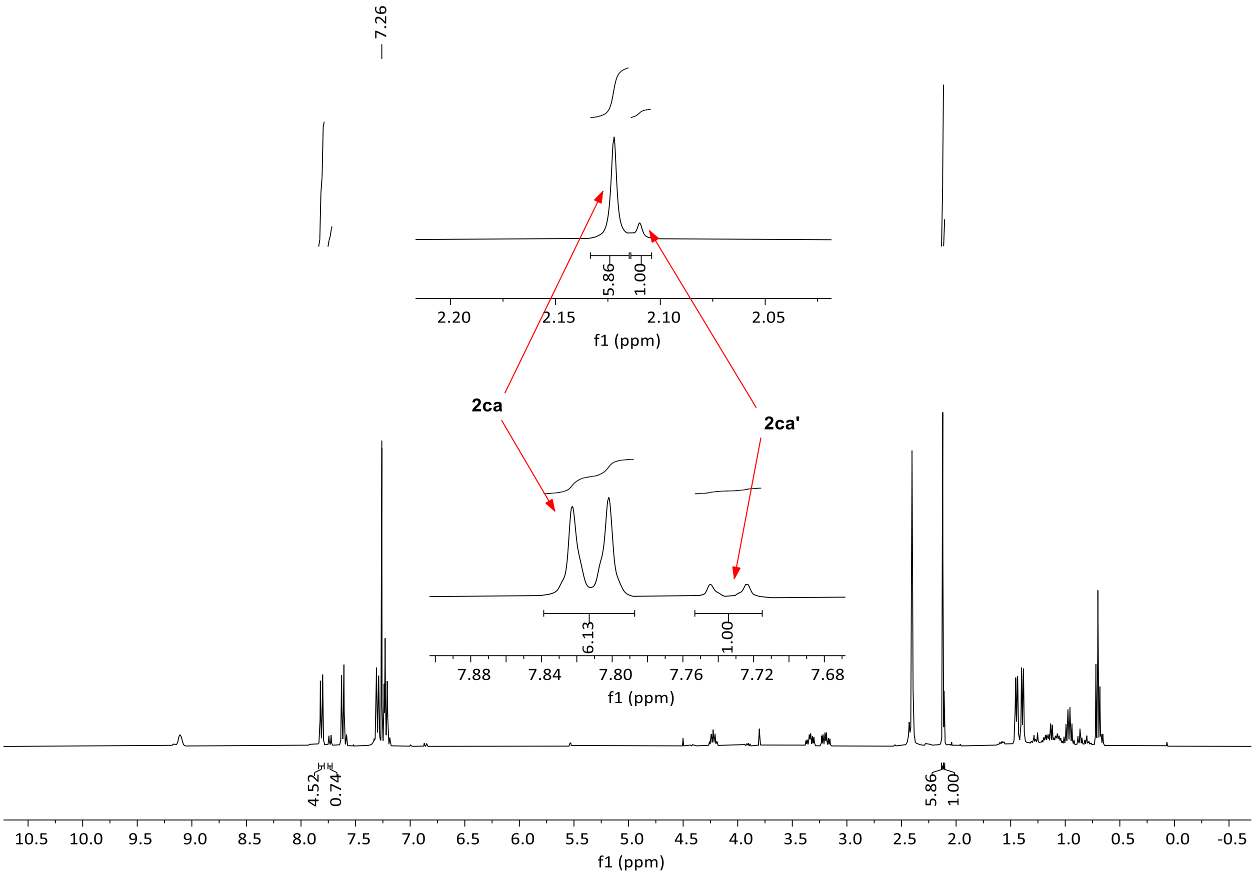

Figure S3. [1]*H NMR (400 MHz, $CDCl_3$) of* ***2ca/2ca'*** *mixture. Integrated peak: TsAr*H *and C(=O)*Me, *showing that* ***2ca:2ca'*** *≈ 6:1. The structure of* **2ca** *was confirmed in latter NOESY experiment (Figure S4).*

HRMS (ESI) m/z: [M + H]+ Calcd for $C_{27}H_{36}N_3O_5S_2$ 546.2091.; Found 546.2081.

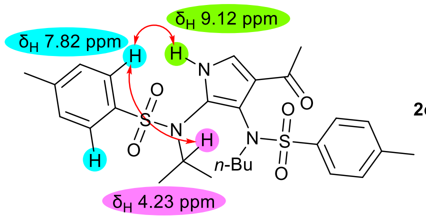

2ca

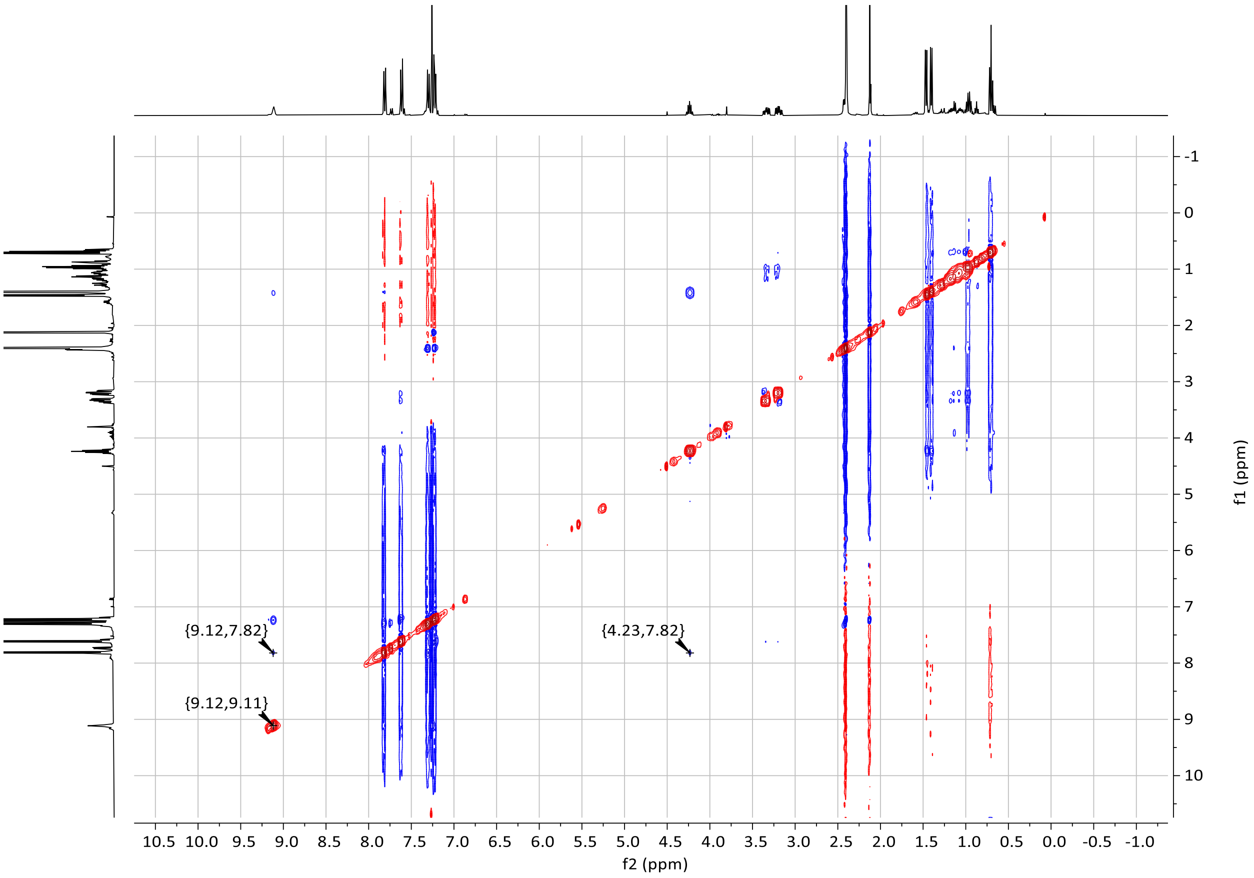

*Figure S4. ^{1}H – ^{1}H NOESY (400 MHz, $CDCl_3$) of **2ca/2ca'** mixture. The picked peaks show the marked correlation between protons in **2ca**, confirming its structure is as proposed and it is the major regioisomer in the mixture.*

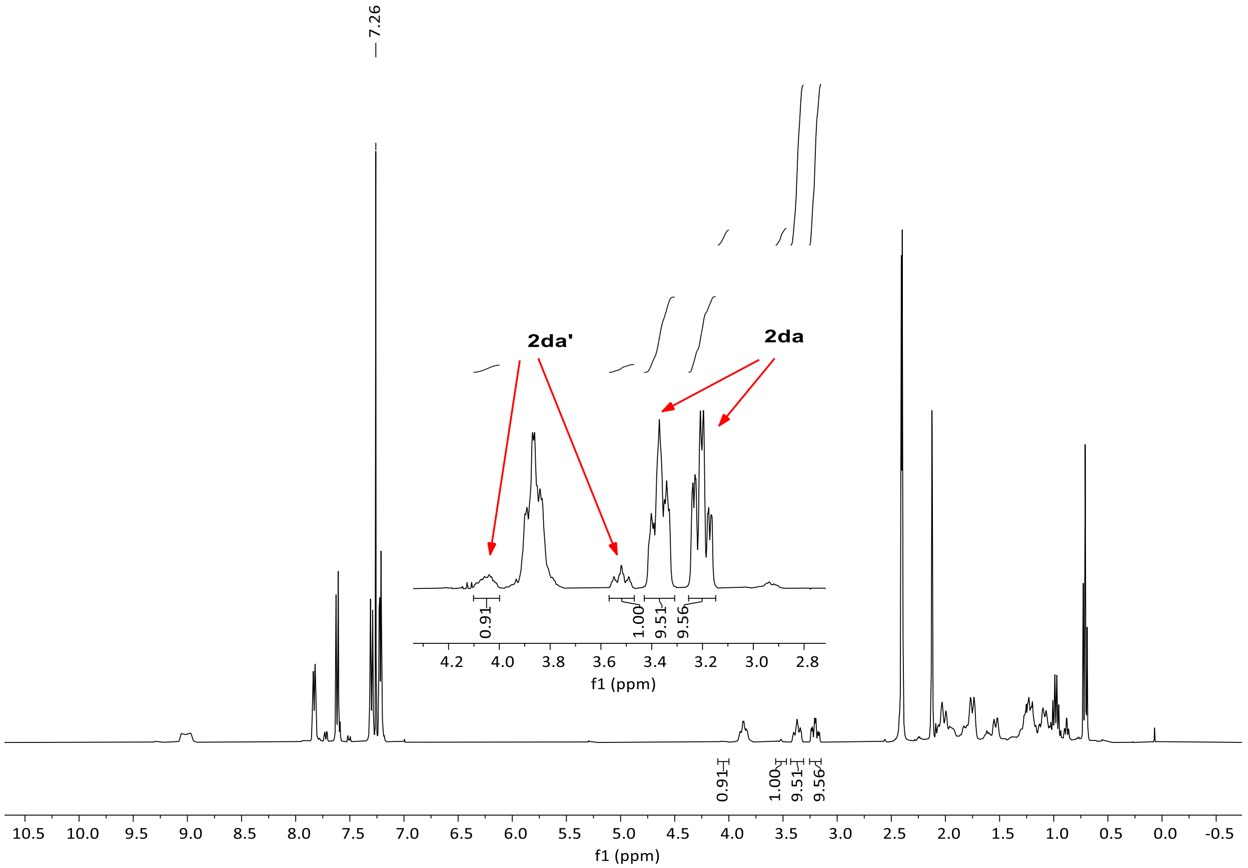

Figure S5. ^{1}*H NMR (400 MHz,* $CDCl_3$*) of* ***2da/2da'*** *mixture. Integrated peak: Cy*CH *and one of Bu*CH_2*, showing that* ***2da*****:*****2da'*** *≈ 10:1. The structure of* ***2da*** *was confirmed in latter NOESY experiment (Figure S6).*

HRMS (ESI) m/z: [M + H]+ Calcd for $C_{30}H_{40}N_3O_5S_2$ 586.2404.; Found 586.2401.

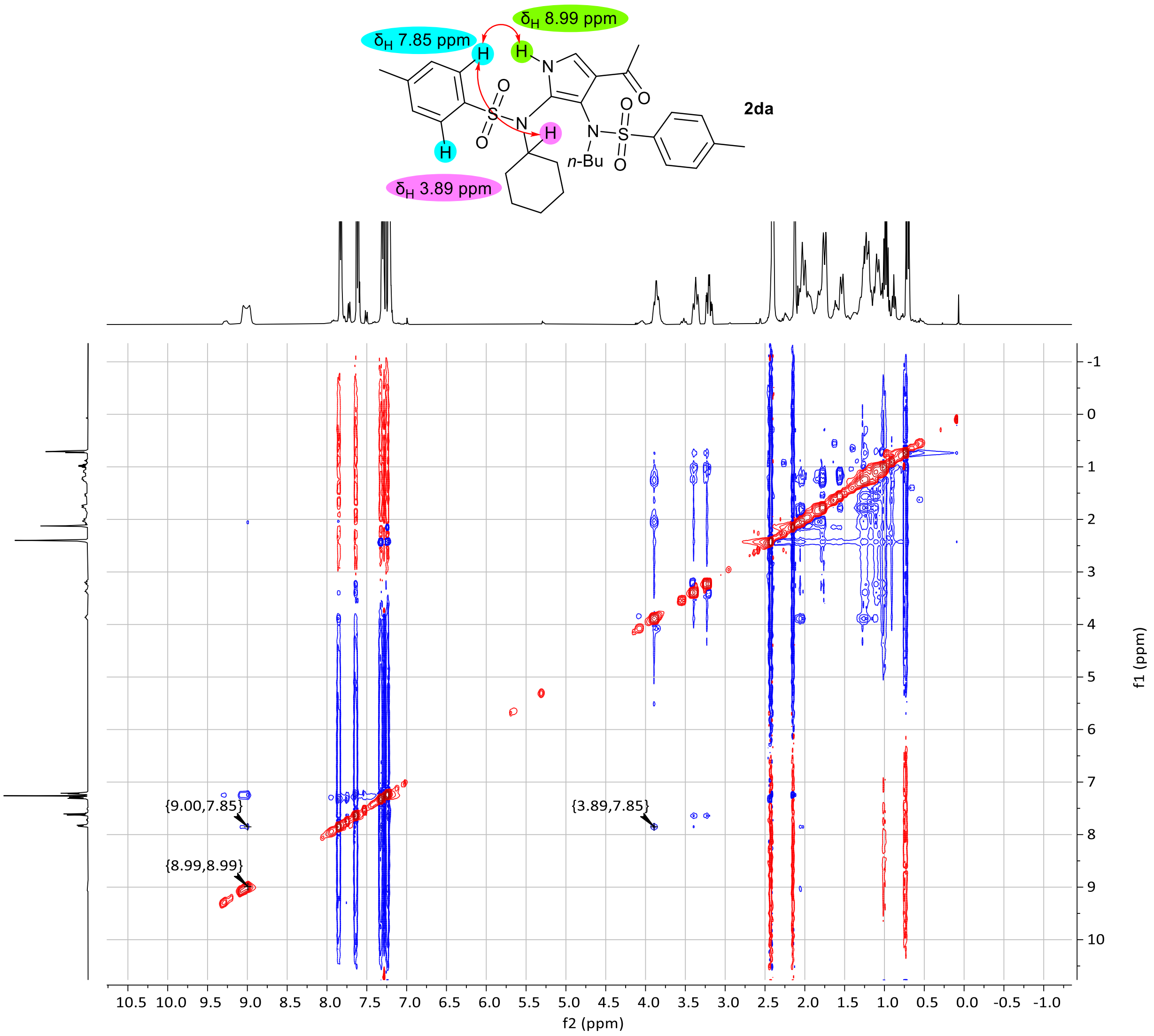

Figure S6. [1]*H – *[1]*H NOESY (400 MHz, $CDCl_3$) of* ***2da/2da'*** *mixture. The picked peaks show the marked correlation between protons in* ***2da****, confirming its structure is as proposed and it is the major regioisomer in the mixture.*

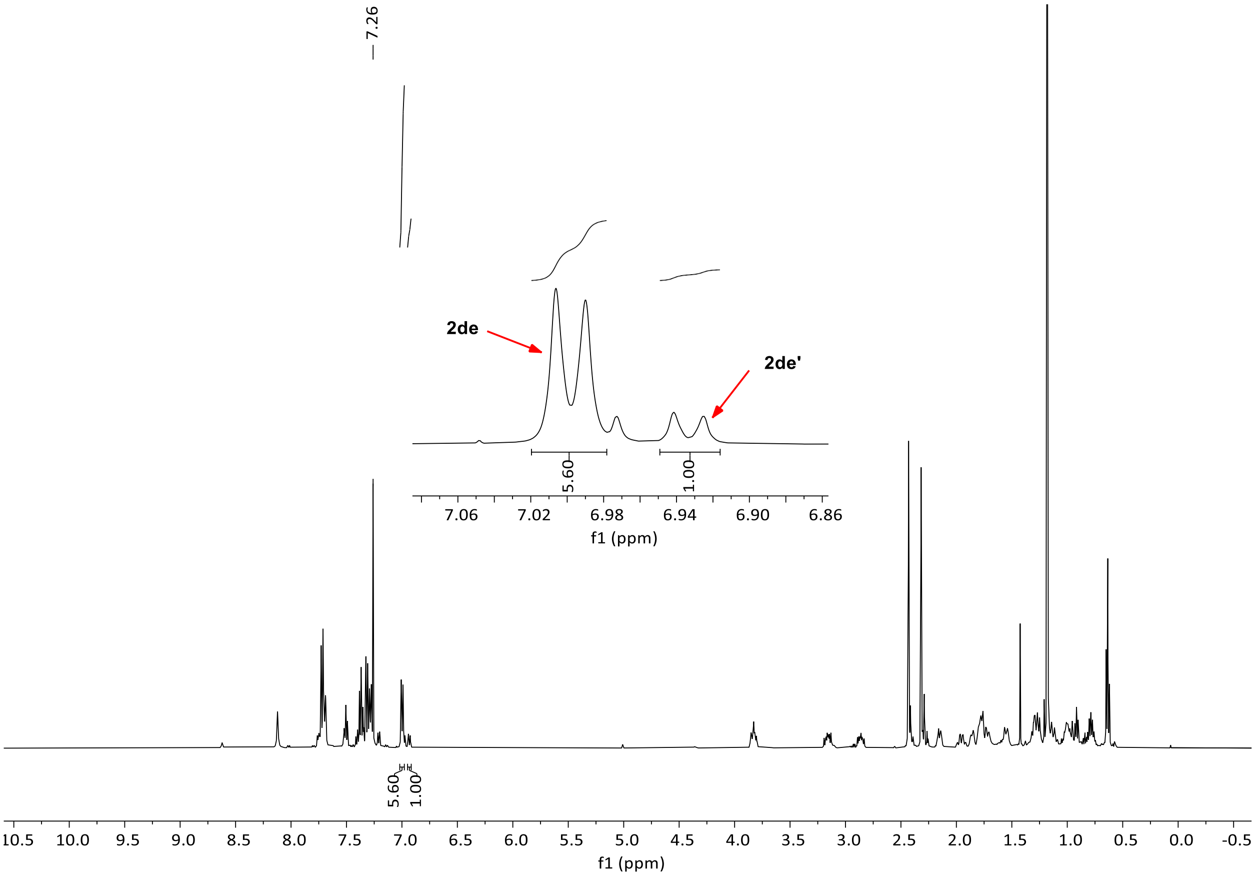

Figure S7. [1]*H NMR (400 MHz, $CDCl_3$) of **2de/2de'** mixture. Integrated peak: Ts*ArH, *showing that **2de**:**2de'** ≈ 6:1. The structure of **2de** was confirmed in latter NOESY experiment (Figure S8).* **HRMS** (ESI) m/z: [M + H]+ Calcd for $C_{39}H_{50}N_3O_5S_2$ 704.3187.; Found 704.3174.

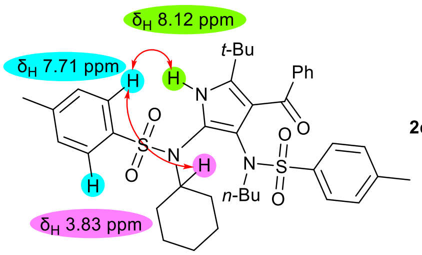

2de

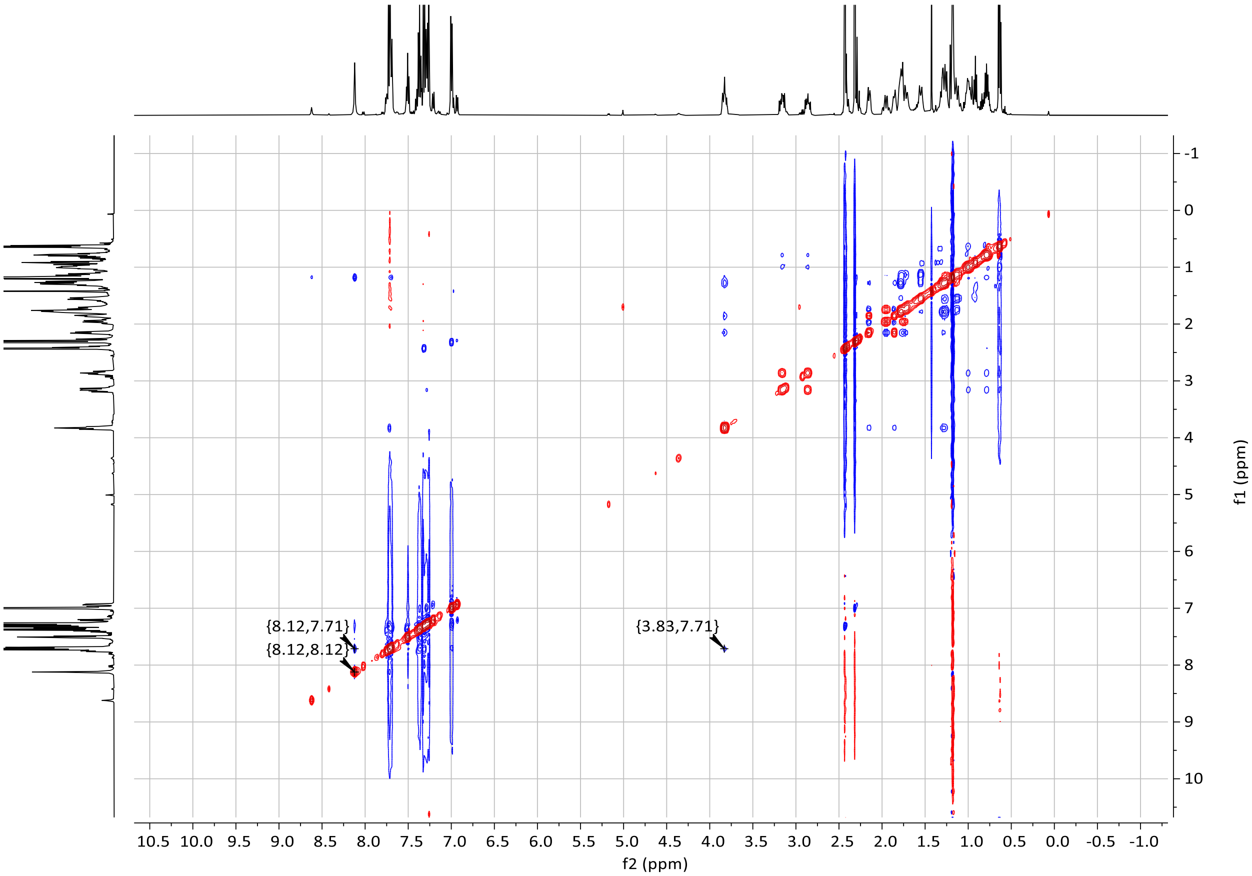

Figure S8. ${}^{1}H - {}^{1}H$ *NOESY (400 MHz, $CDCl_3$) of* ***2de/2de'*** *mixture. The picked peaks show the marked correlation between protons in* ***2de****, confirming its structure is as proposed and it is the major regioisomer in the mixture.*

4 Characterization of Synthesized Compounds

4.1 Yndiamides

***N*-Benzyl-*N*-(((*N*-butyl-4-methylphenyl)sulfonamido)ethynyl)-4-methylbenzenesulfonamide, 1b**

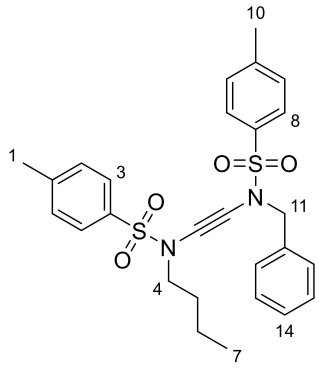

Synthesized from *N*-butyl-4-methylbenzenesulfonamide (455 mg, 2.00 mmol, 1.0 eq.), *N*-benzyl-*N*-(2,2-dibromovinyl)-4-methylbenzenesulfonamide[2] (979 mg, 2.20 mmol, 1.1 eq.), CuI (76 mg, 0.40 mmol, 0.20 eq.), 1,10-phenanthroline (144 mg, 0.80 mmol, 0.40 eq.) and Cs_2CO_3 (1.95 g, 6.00 mmol, 3.0 eq.) with general procedure **2.1**. Purification by flash chromatography (silica gel, 20% Et_2O + 10% DCM/pentane) afforded the title compound (689 mg, 1.35 mmol, 67%) as a white solid.

R_f 0.48 (20% Et_2O + 10% DCM/pentane)

IR (thin film, ν_{max} / cm^{-1}) 2959, 2933, 1363, 1169, 1089, 814, 729;

^{1}H NMR (400 MHz, $CDCl_3$) δ_H 7.67 (2H, d, *J* = 8.3 Hz, TsC*H*), 7.60 (2H, d, *J* = 8.3 Hz, TsC*H*), 7.29 – 7.24 (7H, m, TsC*H*, H13, H14), 7.19 – 7.15 (2H, m, H12), 4.49 (2H, s, H11), 3.17 (2H, t, *J* = 7.2 Hz, H4), 2.44 (6H, s, H1, H10), 1.29 (2H, quin, *J* = 7.2 Hz, H5), 1.13 (2H, sext, *J* = 7.3 Hz, H6), 0.77 (3H, t, *J* = 7.3 Hz, H7);

^{13}C NMR (101 MHz, $CDCl_3$) δ_C 144.6, 144.5, 135.1, 134.8, 129.77, 129.75, 128.9, 128.5, 128.3, 127.8, 127.7, 69.9, 69.2, 56.1, 51.5, 29.8, 21.8, 19.4, 13.7. *Two peaks are obscured due to signal overlap.*

Data consistent with literature values.[1]

***N*-Butyl-*N*-(((*N*-cyclohexyl-4-methylphenyl)sulfonamido)ethynyl)-4-methylbenzenesulfonamide, 1d**

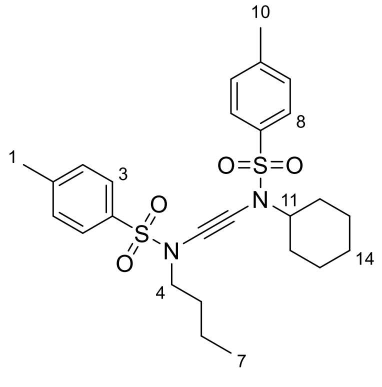

Synthesized from *N*-cyclohexyl-4-methylbenzenesulfonamide (507 mg, 2.00 mmol, 1.0 eq.), *N*-butyl-*N*-(2,2-dibromovinyl)-4-methylbenzenesulfonamide[2] (905 mg, 2.20 mmol, 1.1 eq.), CuI (76 mg, 0.40 mmol, 0.20 eq.), 1,10-phenanthroline (144 mg, 0.80 mmol, 0.40 eq.) and Cs_2CO_3 (1.95 g, 6.00 mmol, 3.0 eq.) with general procedure **2.1**. Purification by flash chromatography (silica gel, 5% → 10% EtOAc/pentane) afforded the title compound (380 mg, 0.76 mmol, 38%) as a white solid.

m.p. 99 °C;

R_f 0.27 (10% EtOAc/pentane);

IR (thin film, ν_{max} / cm^{-1}) 2934, 2859, 1686, 1356, 1162, 1088, 814, 667;

^{1}**H NMR** (400 MHz, $CDCl_3$) δ_H 7.74 – 7.69 (4H, m, H3, H8), 7.32 – 7.27 (4H, m, H2, H9), 3.81 – 3.69 (1H, m, H11), 3.35 (2H, t, *J* = 7.2 Hz, H4), 2.44 (6H, s, H1, H10), 1.75 – 1.64 (2H, m, H12), 1.59 – 1.49 (5H, m, H5, H13, H14), 1.35 – 1.22 (6H, m, H6, H12, H13), 1.00 – 0.91 (1H, m, H14), 0.88 (3H, t, *J* = 7.4 Hz, H7);

^{13}**C NMR** (101 MHz, $CDCl_3$) δ_C 144.5, 144.3, 136.5, 135.2, 129.8, 127.8, 127.7, 127.6, 70.4, 66.8, 59.3, 51.5, 31.2, 30.0, 25.5, 25.0, 21.77, 21.76, 19.5, 13.7.

HRMS (ESI) m/z: $[M + Na]^+$ Calcd for $C_{26}H_{34}N_2N_aO_4S_2$ 525.1853, found 525.1853;

4.2 Isoxazoles

***N*-(5-(*tert*-Butyl)isoxazol-3-yl)-4-methylbenzenesulfonamide, S2d**

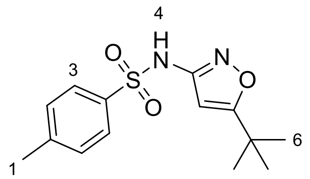

In a round-bottom flask containing a stirrer bar, was dissolved 5-(*tert*-butyl)isoxazole-3-amine (463 mg, 3.30 mmol, 1.1 eq.) and pyridine (0.27 mL, 3.35 mmol, 1.1 eq.) in DCM (4 mL). The solution was cooled to 0 °C on an ice bath before a solution of 4-methylbenzenesulfonyl chloride (573 mg, 3.00 mmol, 1.0 eq.) in DCM (2 mL) was added dropwise. The reaction was slowly warmed to room temperature and stirred until completion by TLC. Upon completion, the reaction mixture was diluted with DCM and washed with 1 M HCl. The organic phase was collected, washed with saturated $NaHCO_3$ solution, then with brine, and finally dried over Na_2SO_4. The solvent was removed *in vacuo* and recrystallization from DCM/pentane afforded the title compound (578 mg, 1.96 mmol, 65%) as a pink solid;

m.p. 122 °C;

$\mathbf{R_f}$ 0.14 (DCM);

IR (thin film, ν_{max} / cm^{-1}) 3140, 2971, 1599, 1459, 1168, 1091, 908, 814, 732, 670;

^{1}**H NMR** (400 MHz, DMSO) δ_H 11.33 (1H, s, H4), 7.75 (2H, d, *J* = 8.2 Hz, H3), 7.40 (2H, d, *J* = 8.2 Hz, H2), 6.04 (1H, s, H5), 2.37 (3H, s, H1), 1.22 (9H, s, H6);

^{13}**C NMR** (101 MHz, DMSO) δ_C 181.1, 157.2, 143.8, 136.7, 129.7, 126.8, 92.3, 32.4, 28.2, 21.0;

HRMS (ESI) m/z: $[M + H]^+$ Calcd for $C_{14}H_{19}N_2O_3S$ 295.1111; Found 295.1111.

Methyl 5-methylisoxazole-3-carboxylate, S2f

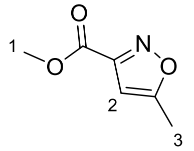

In a round-bottom flask containing a stirrer bar, was dissolved 5-methylisoxazole-3-carboxylic acid (256 mg, 2.01 mmol, 1.0 eq.). in MeOH (4 mL). Concentrated sulfuric acid (97 wt%, 55 μL, 1.01 mmol, 0.5 eq.) was added to the solution. The reaction mixture was stirred under reflux until completion by TLC. Upon completion, the reaction mixture was concentrated in vacuo, diluted with DCM, and washed with saturated $NaHCO_3$ solution. The organic phase was then

collected, washed with brine, and dried over Na_2SO_4. The solvent was removed *in vacuo* to give the title compound (243 mg, 1.72 mmol, 85%) as a white solid;

IR (thin film, ν_{max} / cm^{-1}) 3137, 2965, 1729, 1458, 1275, 1213, 1004, 918, 785, 729;

^{1}H NMR (400 MHz, $CDCl_3$) δ_H 6.40 (1H, s, H2), 3.95 (3H, s, H1), 2.48 (3H, s, H3);

^{13}C NMR (101 MHz, $CDCl_3$) δ_C 171.5, 160.7, 156.4, 102.4, 52.9, 12.4;

Data consistent with literature values.[6]

3,5-Diphenylisoxazole, S2g

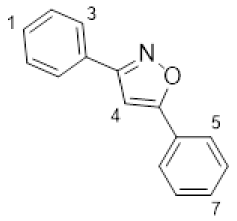

Synthesized from hydroxylamine hydrochloride (215 mg, 3.09 mmol, 1.05 eq.), benzaldehyde (0.30 mL, 2.94 mmol, 1.00 eq.), NaOH (123 mg, 3.09 mmol, 1.05 eq.), Chloramine T trihydrate (870 mg, 3.09 mmol, 1.05 eq.), copper sulfate pentahydrate (22 mg, 88 μmol, 3 mol%), copper turning (1 pc, *ca.* 50 mg), and phenyl acetylene (0.34 mL, 3.09 mmol, 1.05 eq.) with general procedure **2.2**. Column chromatography (10% → 20% EtOAc/pentane) afforded the title compound (225 mg, 2.94 mmol, 35%) as a white solid.

R_f 0.10 (20% EtOAc/pentane);

IR (thin film, ν_{max} / cm^{-1}) 3114, 3050, 1573, 1463, 1451, 1402, 950, 909, 763, 732, 691;

^{1}H NMR (400 MHz, $CDCl_3$) δ_H 7.93 – 7.81 (4H, m, H3, H5), 7.56 – 7.40 (6H, m, H1, H2, H6, H7), 6.84 (1H, s, H4);

^{13}C NMR (101 MHz, $CDCl_3$) δ_C 170.6, 163.1, 130.4, 130.2, 129.3, 129.2, 129.1, 127.7, 127.0, 126.0, 97.6;

Spectral data consistent with literature values.[7]

5-Butyl-3-phenylisoxazole, S2h

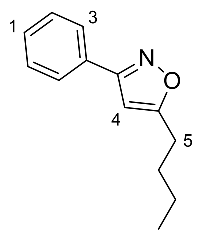

Synthesized from hydroxylamine hydrochloride (143 mg, 2.06 mmol, 1.05 eq.), benzaldehyde (0.20 mL, 2.06 mmol, 1.00 eq.), NaOH (82 mg, 2.06 mmol, 1.05 eq.), Chloramine T trihydrate (580 mg, 2.06 mmol, 1.05 eq.), copper sulfate pentahydrate (15 mg, 60 μmol, 3 mol%), copper turning (1 pc, *ca.* 50 mg), and hex-1-yne (0.24 mL, 2.09 mmol, 1.05 eq.) with general procedure **2.2**. Column chromatography (5% EtOAc/pentane) afforded the title compound (209 mg, 1.04 mmol, 53%) as a pale yellow oil;

R_f 0.58 (5% EtOAc/pentane);

IR (thin film, ν_{max} / cm^{-1}) 2959, 2873, 1602, 1580, 1471, 1408, 908, 768, 731, 693;

^{1}H NMR (400 MHz, $CDCl_3$) δ_H 7.83 – 7.74 (2H, m, H3), 7.49 – 7.39 (3H, m, H1, H2), 6.28 (1H, s, H4), 2.80 (2H, t, *J* = 7.6 Hz, H5), 1.74 (2H, quin, *J* = 7.5 Hz, H6), 1.44 (2H, sext, *J* = 7.4 Hz, H7), 0.97 (3H, t, *J* = 7.4 Hz, H8);

^{13}C NMR (101 MHz, $CDCl_3$) δ_C 174.4, 162.5, 129.9, 129.6, 129.0, 126.9, 98.9, 29.8, 26.7, 22.3, 13.8;

Data consistent with literature values.[8]

5-Cyclohexyl-3-phenylisoxazole, S2i

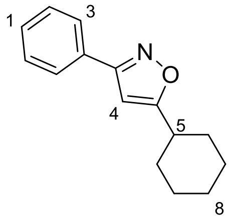

Synthesized from hydroxylamine hydrochloride (286 mg, 4.12 mmol, 1.05 eq.), benzaldehyde (0.40 mL, 3.92 mmol, 1.00 eq.), NaOH (165 mg, 4.12 mmol, 1.05 eq.), Chloramine T trihydrate (1.16 g, 4.12 mmol, 1.05 eq.), copper sulfate pentahydrate (29 mg, 0.12 mmol, 3 mol%), copper turning (1 pc, *ca.* 50 mg), and ethynylcyclohexane (0.54 mL, 4.12 mmol, 1.05 eq.) with general procedure **2.2**. Column chromatography (5% → 10% EtOAc/pentane) afforded the title compound (391 mg, 1.72 mmol, 44%) as a white solid;

R_f 0.78 (10% EtOAc/pentane);

IR (thin film, ν_{max} / cm^{-1}) 2934, 2859, 1597, 1580, 1406, 924, 765, 690;

^{1}H NMR (400 MHz, $CDCl_3$) δH 7.83 – 7.76 (2H, m, H3), 7.48 – 7.39 (3H, m, H1, H2), 6.25 (1H, s, H4), 2.83 (1H, tt, *J* = 11.3, 3.6 Hz, H5), 2.15 – 2.07 (2H, m, H6), 1.88 – 1.80 (2H, m, H7), 1.78 – 1.70 (1H, m, H8), 1.56 – 1.36 (4H, m, H6, H7), 1.35 – 1.23 (1H, m, H8);

^{13}C NMR (101 MHz, $CDCl_3$) δ_C 178.5, 162.3, 129.9, 129.7, 129.0, 126.9, 97.2, 36.6, 31.4, 26.0, 25.8.

Data consistent with literature values.[9]

5-(Cyclohex-1-en-1-yl)-3-phenylisoxazole, S2j

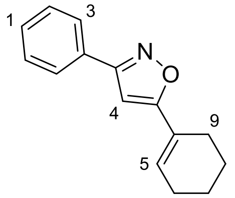

Synthesized from hydroxylamine hydrochloride (286 mg, 4.12 mmol, 1.05 eq.), benzaldehyde (0.40 mL, 3.92 mmol, 1.00 eq.), NaOH (165 mg, 4.12 mmol, 1.05 eq.), Chloramine T trihydrate (1.16 g, 4.12 mmol, 1.05 eq.), copper sulfate pentahydrate (29 mg, 0.12 mmol, 3 mol%), copper turning (1 pc, *ca.* 50 mg), and 1-ethynylcyclohex-1-ene (0.48 mL, 4.12 mmol, 1.05 eq.) with general procedure **2.2**. Column chromatography (5% → 10% EtOAc/pentane) afforded the title compound (467 mg, 2.07 mmol, 53%) as a white solid;

R_f 0.72 (10% EtOAc/pentane);

IR (thin film, ν_{max} / cm^{-1}) 2934, 1564, 1470, 1437, 1406, 920, 801, 766, 687;

^{1}H NMR (400 MHz, $CDCl_3$) δ_H 7.85 – 7.77 (2H, m, H3), 7.49 – 7.39 (3H, m, H1, H2), 6.66 (1H, tt, *J* = 4.0, 1.7 Hz, H5), 6.38 (1H, s, H4), 2.42 – 2.36 (2H, m, H9), 2.29 – 2.23 (2H, m, H6), 1.83 – 1.75 (2H, m, H8), 1.73 – 1.65 (2H, m, H7);

^{13}C NMR (101 MHz, $CDCl_3$) δ_C 171.8, 162.6, 130.3, 129.9, 129.6, 129.0, 126.9, 125.5, 96.3, 25.6, 25.4, 22.2, 21.9;

Data consistent with literature values.[10]

5-(4-Fluorophenyl)-3-phenylisoxazole, S2k

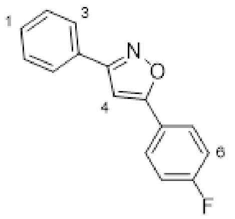

Synthesized from hydroxylamine hydrochloride (151 mg, 2.16 mmol, 1.05 eq.), benzaldehyde (0.21 mL, 2.06 mmol, 1.00 eq.), NaOH (86 mg, 2.16 mmol, 1.05 eq.), Chloramine T trihydrate (609 mg, 2.16 mmol, 1.05 eq.), copper sulfate pentahydrate (15 mg, 62 μmol, 3 mol%), copper turning (1 pc, *ca.* 50 mg), and 1-ethynyl-4-fluorobenzene (260 mg, 2.16 mmol, 1.05 eq.) with general procedure **2.2**. Recrystallization from DCM/pentane afforded the title compound (133 mg, 0.56 mmol, 27%) as a yellow solid;

IR (thin film, ν_{max} / cm^{-1}) 3115, 1617, 1501, 1462, 1418, 1237, 951, 844, 814, 768, 697;

^{1}H NMR (400 MHz, $CDCl_3$) δ_C 7.90 – 7.79 (4H, m, H3, H5), 7.54 – 7.43 (3H, m, H1, H2), 7.23 – 7.15 (2H, m, H6), 6.78 (1H, s, H4);

^{13}C NMR (101 MHz, $CDCl_3$) δ_C 169.6, 164.0 (d, $^1J_{C-F}$ = 250.7 Hz), 163.2, 130.2, 129.2, 129.1, 128.1 (d, $^3J_{C-F}$ = 8.6 Hz), 127.0, 124.03 (d, $^4J_{C-F}$ = 3.2 Hz), 116.4 (d, $^2J_{C-F}$ = 22.2 Hz), 97.4;

^{19}F NMR (377 MHz, $CDCl_3$) δ_F -109.45;

Data consistent with literature values.[11]

4-(3-Phenylisoxazol-5-yl)benzonitrile, S2l

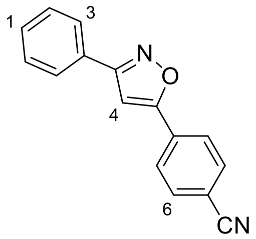

Synthesized from hydroxylamine hydrochloride (293 mg, 4.22 mmol, 1.05 eq.), benzaldehyde (0.41 mL, 4.02 mmol, 1.00 eq.), NaOH (169 mg, 4.22 mmol, 1.05 eq.), Chloramine T trihydrate (1.19 g, 4.22 mmol, 1.05 eq.), copper sulfate pentahydrate (30 mg, 0.12 mmol, 3 mol%), copper turning (1 pc, *ca.* 50 mg), and 1-ethynylbenzonitrile (536 mg, 4.22 mmol, 1.05 eq.) with general procedure **2.2**. Column chromatography (10% → 20% EtOAc/pentane) afforded the title compound (335 mg, 1.36 mmol, 34%) as a white solid.

R_f 0.27 (10% EtOAc/pentane);

IR (thin film, ν_{max} / cm^{-1}) 3111, 2981, 2234, 1597, 1462, 1440, 819, 772, 698;

^{1}H NMR (600 MHz, $CDCl_3$) δ_H 7.95 (2H, d, J = 8.6 Hz, H6), 7.89 – 7.84 (2H, m, H3), 7.79 (2H, d, J = 8.6 Hz, H5), 7.55 – 7.46 (3H, m, H1, H2), 6.96 (1H, s, H4);

^{13}C NMR (151 MHz, $CDCl_3$) δ_C 168.3, 163.4, 133.0, 131.3, 130.5, 129.2, 128.7, 127.0, 126.4, 118.3, 113.8, 99.8.

Data consistent with literature values.[12]

5-(4-Methoxyphenyl)-3-phenylisoxazole, S2m

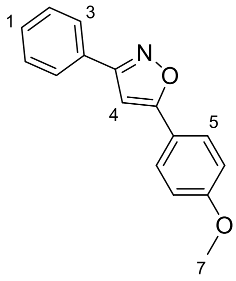

Synthesized from hydroxylamine hydrochloride (143 mg, 2.06 mmol, 1.05 eq.), benzaldehyde (0.20 mL, 2.06 mmol, 1.00 eq.), NaOH (82 mg, 2.06 mmol, 1.05 eq.), Chloramine T trihydrate (580 mg, 2.06 mmol, 1.05 eq.), copper sulfate pentahydrate (15 mg, 60 μmol, 3 mol%), copper turning (1 pc, *ca.* 50 mg), and 1-ethynyl-4-methoxybenzene (272 mg, 2.06 mmol, 1.05 eq.) with general procedure **2.2**. Column chromatography (10% EtOAc/pentane) afforded the title compound (135 mg, 0.54 mmol, 27%) as a white solid;

R_f 0.31 (10% EtOAc/pentane);

IR (thin film, ν_{max} / cm^{-1}) 2964, 2839, 1615, 1504, 1466, 1262, 1033, 767, 690;

^{1}H NMR (400 MHz, $CDCl_3$) δ_H 7.89 – 7.84 (2H, m, H3), 7.78 (2H, d, *J* = 9.0 Hz, H5), 7.51 – 7.44 (3H, m, H1, H2), 7.00 (2H, d, *J* = 9.0 Hz, H6), 6.71 (1H, s, H4), 3.87 (3H, s, H7);

^{13}C NMR (101 MHz, $CDCl_3$) δ_C 170.5, 163.1, 161.3, 130.1, 129.5, 129.0, 127.6, 127.0, 120.5, 114.6, 96.3, 55.6;

Data consistent with literature values.[7]

3-(*tert*-Butyl)-5-phenylisoxazole, S2o

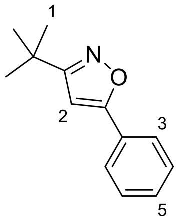

Synthesized from hydroxylamine hydrochloride (202 mg, 2.91 mmol, 1.05 eq.), pivalaldehyde (0.30 mL, 2.76 mmol, 1.00 eq.), NaOH (116 mg, 2.90 mmol, 1.05 eq.), Chloramine T trihydrate (817 mg, 2.90 mmol, 1.05 eq.), copper sulfate pentahydrate (21 mg, 83 μmol, 3 mol%), copper turning (1 pc, *ca.* 50 mg), and phenylacetylene (0.32 mL, 2.91 mmol, 1.05 eq.) with general procedure **2.2**. Column chromatography (5% EtOAc/pentane) afforded the title compound (223 mg, 1.11 mmol, 40%) as a yellow oil;

R_f 0.42 (5% EtOAc/pentane);

IR (thin film, ν_{max} / cm^{-1}) 2966, 1575, 1448, 1406, 1243, 906, 764, 690;

^{1}H NMR (400 MHz, $CDCl_3$) δ_H 7.80 – 7.73 (2H, m, H3), 7.48 – 7.38 (3H, m, H4, H5), 6.43 (1H, s, H2), 1.39 (9H, s, H1);

^{13}C NMR (101 MHz, $CDCl_3$) δ_C 172.8, 169.5, 130.0, 129.0, 128.0, 125.9, 97.5, 32.2, 29.7;

Data consistent with literature values.[13]

3-(4-Fluorophenyl)-5-phenylisoxazole, S2p

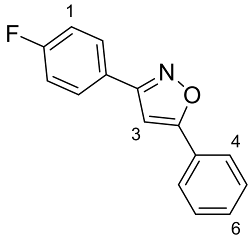

Synthesized from hydroxylamine hydrochloride (367 mg, 5.29 mmol, 1.05 eq.), 4-fluorobenzaldehyde (0.54 mL, 5.03 mmol, 1.00 eq.), NaOH (211 mg, 5.29 mmol, 1.05 eq.), Chloramine T trihydrate (1.49 g, 5.29 mmol, 1.05 eq.), copper sulfate pentahydrate (38 mg, 0.15 mmol, 3 mol%), copper turning (1 pc, *ca.* 50 mg), and phenylacetylene (0.58 mL, 5.29 mmol, 1.05 eq.) with general procedure **2.2**. Column chromatography (10% EtOAc/pentane) afforded the title compound (484 mg, 2.02 mmol, 40%) as a white solid;

R_f 0.76 (10% EtOAc/pentane);

IR (thin film, ν_{max} / cm^{-1}) 3112, 1606, 1527, 1493, 1448, 1231, 845, 816, 765, 693;

^{1}H NMR (400 MHz, $CDCl_3$) δ_H 7.90 – 7.81 (4H, m, H4, H2), 7.52 – 7.42 (3H, m, H1, H6), 7.21 – 7.13 (2H, m, H5), 6.79 (1H, s, H3);

^{13}C NMR (101 MHz, $CDCl_3$) δ_C 170.8, 164.0 (d, $^1J_{C\text{-}F}$ = 249.9 Hz), 162.2, 130.5, 129.2, 128.9 (d, $^3J_{C\text{-}F}$ = 8.4 Hz), 127.5, 126.0, 125.5 (d, $^4J_{C\text{-}F}$ = 3.4 Hz), 116.2 (d, $^2J_{C\text{-}F}$ = 21.9 Hz), 97.5;

^{19}F NMR (377 MHz, $CDCl_3$) δ_F -110.57;

Data consistent with literature values.[14]

4-(5-Phenylisoxazol-3-yl)benzonitrile, S2q

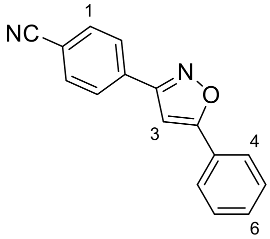

Synthesized from hydroxylamine hydrochloride (292 mg, 4.19 mmol, 1.05 eq.), 4-cyanobenzaldehyde (525 mg, 4.00 mmol, 1.00 eq.), NaOH (168 mg, 4.19 mmol, 1.05 eq.), Chloramine T trihydrate (1.18 g, 4.19 mmol, 1.05 eq.), copper sulfate pentahydrate (30 mg, 0.12 mmol, 3 mol%), copper turning (1 pc, *ca.* 50 mg), and phenylacetylene (0.46 mL, 4.19 mmol, 1.05 eq.) with general procedure **2.2**. Column chromatography (20% EtOAc/pentane) afforded the title compound (324 mg, 1.32 mmol, 33%) as a yellow solid;

R_f 0.42 (20% EtOAc/pentane);

IR (thin film, ν_{max} / cm^{-1}) 3113, 1447, 908, 768, 732, 695;

^{1}H NMR (400 MHz, $CDCl_3$) δ_H 7.99 (2H, d, *J* = 8.6 Hz, H1), 7.87 – 7.83 (2H, m, H4), 7.78 (2H, d, *J* = 8.6 Hz, H2), 7.55 – 7.47 (3H, m, H5, H6), 6.86 (1H, s, H3);

^{13}C NMR (101 MHz, $CDCl_3$) δ_C 171.5, 161.6, 133.6, 132.9, 130.8, 129.3, 127.5, 127.1, 126.0, 118.5, 113.8, 97.5;

^{1}H NMR data constituent with literature values, while we suspect the literature has misidentified their ^{13}C NMR peaks.[15]

3-(4-Methoxyphenyl)-5-phenylisoxazole, S2r

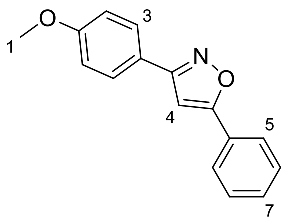

Synthesized from hydroxylamine hydrochloride (300 mg, 4.32 mmol, 1.05 eq.), 4-methoxybenzaldehyde (0.50 mL, 4.11 mmol, 1.00 eq.), NaOH (173 mg, 4.33 mmol, 1.05 eq.), Chloramine T trihydrate (1.22 g, 4.32 mmol, 1.05 eq.), copper sulfate pentahydrate (31 mg, 0.12 mmol, 3 mol%), copper turning (1 pc, *ca.* 50 mg), and phenylacetylene (0.48 mL, 4.37 mmol, 1.06 eq.) with general procedure **2.2**. Column chromatography (5% → 10% EtOAc/pentane) afforded the title compound (424 mg, 1.69 mmol, 41%) as a white solid;

R$_f$ 0.42 (10% EtOAc/pentane);

IR (thin film, ν_{max} / cm^{-1}) 3003, 2837, 1612, 1493, 1448, 1428, 1250, 1177, 1031, 837, 763, 686;

^{1}H NMR (400 MHz, $CDCl_3$) δ_H 7.86 – 7.78 (4H, m, H3, H5), 7.52 – 7.41 (3H, m, H6, H7), 7.00 (2H, d, *J* = 8.8 Hz, H2), 6.78 (1H, s, H4), 3.87 (3H, s, H1);

^{13}C NMR (101 MHz, $CDCl_3$) δ_C 170.3, 162.7, 161.2, 130.3, 129.1, 128.4, 127.8, 126.0, 121.9, 114.5, 97.4, 55.5;

Data consistent with literature values.[7]

3-(Furan-2-yl)-5-phenylisoxazole, S2s

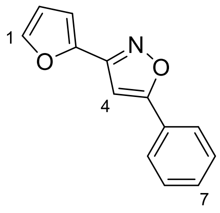

Synthesized from hydroxylamine hydrochloride (176 mg, 2.54 mmol, 1.05 eq.), furan-2-carbaldehyde (0.20 mL, 2.41 mmol, 1.00 eq.), NaOH (101 mg, 2.54 mmol, 1.05 eq.), Chloramine T trihydrate (714 mg, 2.54 mmol, 1.05 eq.), copper sulfate pentahydrate (18 mg, 72 μmol, 3 mol%), copper turning (1 pc, *ca.* 50 mg), and phenylacetylene (0.28 mL, 2.54 mmol, 1.05 eq.) with general procedure **2.2**. Column chromatography (5% → 10% → 20% EtOAc/pentane) afforded the title compound (124 mg, 0.59 mmol, 24%) as a white solid;

R$_f$ 0.67 (20% EtOAc/pentane);

IR (thin film, ν_{max} / cm^{-1}) 3269, 2961, 1691, 1453, 1346, 1162, 1090, 669;

^{1}H NMR (400 MHz, $CDCl_3$) δ_H 7.85 – 7.80 (2H, m, H5), 7.58 (1H, dd, *J* = 1.8, 0.7 Hz, H1), 7.52 – 7.43 (3H, m, H6, H7), 6.96 (1H, dd, *J* = 3.4, 0.7 Hz, H3), 6.78 (1H, s, H4), 6.55 (1H, dd, *J* = 3.4, 1.8 Hz, H2);

^{13}C NMR (101 MHz, $CDCl_3$) δ_C 170.3, 155.5, 144.5, 144.0, 130.5, 129.2, 127.3, 126.0, 111.9, 110.3, 97.1;

Data consistent with literature values.[16]

5-Phenyl-3-(pyridin-2-yl)isoxazole

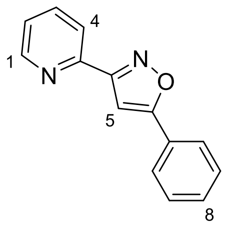

Synthesized from hydroxylamine hydrochloride (730 mg, 10.50 mmol, 1.05 eq.), picolinaldehyde (0.95 mL, 10.00 mmol, 1.00 eq.), NaOH (420 mg, 10.05 mmol, 1.05 eq.), Chloramine T trihydrate (2.96 g, 10.05 mmol, 1.05 eq.), copper sulfate pentahydrate (75 mg, 0.30 mmol, 3 mol%), copper turning (1 pc, *ca*. 50 mg), and phenylacetylene (1.2 mL, 10.93 mmol, 1.09 eq.) with general procedure **2.2**. Column chromatography (10% → 20% EtOAc/pentane) afforded the title compound (242 mg, 1.09 mmol, 11%) as a beige solid;

R_f 0.22 (10% EtOAc/pentane);

^{1}H NMR (400 MHz, $CDCl_3$) δ_H 8.72 (1H, ddd, *J* = 4.9, 1.8, 1.0 Hz, H1), 8.14 (1H, dt, *J* = 7.9, 1.1 Hz, H4), 7.88 – 7.85 (2H, m, H6), 7.82 (1H, td, *J* = 7.8, 1.8 Hz, H3), 7.52 – 7.45 (3H, m, H7, H8), 7.37 (1H, ddd, *J* = 7.6, 4.9, 1.2 Hz, H2), 7.20 (1H, s, H5);

^{13}C NMR (101 MHz, $CDCl_3$) δ_C 170.8, 164.0, 149.9, 148.8, 137.1, 130.4, 129.2, 127.6, 126.0, 124.7, 121.8, 98.5.

Data consistent with literature values.[9]

3-Isopropyl-5-phenylisoxazole, S2dd

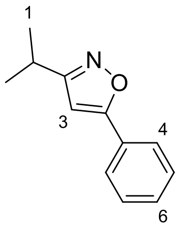

Synthesized from hydroxylamine hydrochloride (292 mg, 4.19 mmol, 1.05 eq.), isobutyraldehyde (0.37 mL, 4.00 mmol, 1.00 eq.), NaOH (168 mg, 4.19 mmol, 1.05 eq.), Chloramine T trihydrate (1.18 mg, 4.19 mmol, 1.05 eq.), copper sulfate pentahydrate (30 mg, 0.12 mmol, 3 mol%), copper turning (1 pc, *ca*. 50 mg), and phenylacetylene (0.46 mL, 4.19 mmol, 1.05 eq.) with general procedure **2.2**. Column chromatography (5% EtOAc/pentane) afforded the title compound (343 mg, 1.83 mmol, 46%) as a yellow oil;

R_f 0.48 (5% EtOAc/pentane);

IR (thin film, ν_{max} / cm^{-1}) 2971, 2883, 1575, 1451, 1066, 948, 908, 764, 731, 690;

^{1}H NMR (500 MHz, $CDCl_3$) δ_H 7.79 – 7.74 (2H, m, H4), 7.46 – 7.38 (3H, m, H5, H6), 6.39 (1H, s, H3), 3.11 (1H, hept, *J* = 7.0 Hz, H2), 1.34 (6H, d, *J* = 7.0 Hz, H1);

^{13}C NMR (126 MHz, $CDCl_3$) δ_C 170.1, 169.6, 130.0, 129.0, 127.9, 125.8, 97.7, 26.7, 21.9;

Data consistent with literature values.[17]

Naphtho[1,2-*d*]isoxazole, S2yb

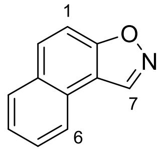

Synthesized from 2-hydroxy-1-naphthaldehyde (1.72 g, 10.0 mmol, 1.0 eq.), hydroxylamine hydrochloride (1.04 g, 15.0 mmol. 1.5 eq.), $NaHCO_3$ (1.68 g, 20.0 mmol, 2.0 eq.), PPh_3 (3.28 g, 12.5 mmol, 1.25 eq.) and DIAD (2.4 mL, 12.2 mmol, 1.22 eq.) with general procedure **2.4.** Column chromatography (silica gel, 0% → 10% → 20% Et_2O/pentane) afforded the title compound (738 mg, 4.4 mmol, 44%) as a white solid;

R_f 0.47 (20% Et_2O/pentane);

IR (thin film, ν_{max} / cm^{-1}) 3069, 1633, 1582, 1532, 1253, 1169, 930, 810, 753;

^{1}H NMR (400 MHz, $CDCl_3$) δ_H 9.10 (1H, d, *J* = 0.8 Hz, H7), 8.14 (1H, d, *J* = 8.1 Hz, H3), 8.02 – 7.92 (2H, m, H2, H6), 7.76 – 7.65 (2H, m, H1, H4), 7.57 (1H, ddd, *J* = 8.2, 7.2, 1.1 Hz, H5);

^{13}C NMR (101 MHz, $CDCl_3$) δ_C 162.4, 145.0, 131.8, 130.6, 129.2, 128.3, 126.9, 125.7, 123.3, 116.5, 110.4.

Data consistent with literature values.[18]

***N,N*-Diethylbenzo[*d*]isoxazol-6-amine, S2yc**

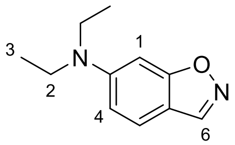

Synthesized from 4-(diethylamino)-2-hydroxybenzaldehyde (1.93 g, 10.0 mmol, 1.0 eq.), hydroxylamine hydrochloride (1.04 g, 15.0 mmol. 1.5 eq.), $NaHCO_3$ (1.68 g, 20.0 mmol, 2.0 eq.), PPh_3 (3.28 g, 12.5 mmol, 1.25 eq.) and DIAD (2.4 mL, 12.2 mmol, 1.22 eq.) with general procedure **2.4.** Column chromatography (silica gel, DCM) afforded the title compound (1.28 g, 6.7 mmol, 67%) as a pink oil;

R_f 0.50 (DCM);

IR (thin film, ν_{max} / cm^{-1}) 2974, 1623, 1518, 1497, 1356, 1124, 803, 730;

^{1}H NMR (400 MHz, $CDCl_3$) δ_H 8.44 (1H, d, *J* = 1.0 Hz, H6), 7.45 (1H, d, *J* = 8.8 Hz, H5), 6.71 (1H, dd, *J* = 8.8, 2.2 Hz, H4), 6.69 – 6.67 (1H, m, H1), 3.43 (4H, q, *J* = 7.1 Hz, H2), 1.21 (6H, t, *J* = 7.1 Hz, H3);

^{13}C NMR (101 MHz, $CDCl_3$) δ_C 165.2, 150.2, 145.7, 122.1, 110.8, 110.6, 89.6, 45.1, 12.5;

HRMS (ESI) m/z: $[M + H]^+$ Calcd for $C_{11}H_{15}N_2O$ 191.1179.; Found 191.1179;

^{1}H NMR data consistent with literature values.[4]

7-(*tert*-Butyl)benzo[*d*]isoxazole, S2yd

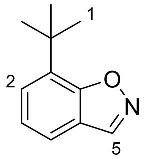

Synthesized from 3-(*tert*-butyl)-2-hydroxybenzaldehyde (1.71 mL, 10.0 mmol, 1.0 eq.), hydroxylamine hydrochloride (1.04 g, 15.0 mmol. 1.5 eq.), $NaHCO_3$ (1.68 g, 20.0 mmol, 2.0 eq.), PPh_3 (3.28 g, 12.5 mmol, 1.25 eq.) and DIAD (2.4 mL, 12.2 mmol, 1.22 eq.) with

general procedure **2.4.** Column chromatography (silica gel, DCM) followed by recrystallization from DCM/pentane afforded the title compound (0.48 g, 2.74 mmol, 27%) as a white solid;

m.p. 76 °C;

R_f 0.61 (10% EtOAc/pentane);

IR (thin film, ν_{max} / cm^{-1}) 2966, 2873, 1597, 1469, 1365, 1175, 908, 860, 751, 731;

^{1}H NMR (400 MHz, $CDCl_3$) δ_H 8.69 (1H, s, H5), 7.58 (1H, dd, *J* = 7.8, 1.1 Hz, H4), 7.43 (1H, dd, *J* = 7.4, 1.1 Hz, H2), 7.26 (1H, t, *J* = 7.6 Hz, H3), 1.53 (9H, s, H1);

^{13}C NMR (101 MHz, $CDCl_3$) δ 161.2, 146.3, 134.6, 126.2, 124.1, 122.0, 119.8, 34.6, 29.8;

HRMS (ESI) m/z: $[M + H]^+$ Calcd for $C_{11}H_{14}NO$ 176.1070; Found 176.1071;

4.3 Au(I)-Catalyzed Reactions of Yndiamides

***N,N'*-(4-Formyl-1*H*-pyrrole-2,3-diyl)bis(*N*-butyl-4-methylbenzenesulfonamide), 2a**

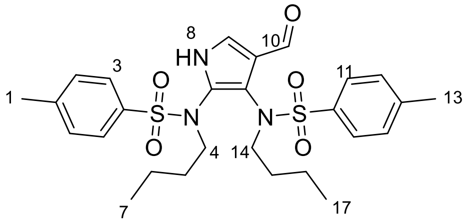

0.10 mmol scale: Synthesized from *N,N'*-(ethyne-1,2-diyl)bis(*N*-butyl-4-methylbenzenesulfonamide) (48 mg, 0.10 mmol, 1.0 eq.), isoxazole (13 μL, 0.20 mmol, 2.0 eq.), chloro[tris(2,4-di-tert-butylphenyl)phosphite]gold(I) (4.4 mg, 5.0 μmol, 5 mol%) and silver bis(trifluoromethanesulfonyl)imide (2.0 mg, 5.2 μmol, 5 mol%) in DCE (anhy., 0.2 mL) with general procedure **2.3** at room temperature for 2 h. Column chromatography (silica gel, 20% → 40% EtOAc/pentane) afforded the title compound (43 mg, 79 μmol, 78%) as a brown solid.

1 mmol scale: Synthesized from *N,N'*-(ethyne-1,2-diyl)bis(*N*-butyl-4-methylbenzenesulfonamide) (477 mg, 1.00 mmol, 1.0 eq.), isoxazole (0.13 mL, 2.03 mmol, 2.0 eq.), chloro[tris(2,4-di-tert-butylphenyl)phosphite]gold(I) (44 mg, 5.0 μmol, 5 mol%) and silver bis(trifluoromethanesulfonyl)imide (20 mg, 0.05 mmol, 5 mol%) in DCE (anhy., 2.0 mL) with general procedure **2.3** at room temperature for 2 h. Column chromatography (silica gel, 20% → 40% EtOAc/pentane) afforded the title compound (407 mg, 0.75 mmol, 75%) as a brown solid.

m.p. 138 °C (decomp.);

R_f 0.37 (40% EtOAc/pentane);

IR (thin film, ν_{max} / cm^{-1}) 3295, 2960, 2873, 1673, 1519, 1457, 1345, 1163, 1090, 814, 666;

^{1}H NMR (500 MHz, C_6D_6) δ_H 9.18 (1H, s, H10), 8.97 (1H, s, H8), 7.98 (2H, d, *J* = 8.2 Hz, H3), 7.72 (2H, d, *J* = 8.2 Hz, H11), 6.88 (2H, d, *J* = 8.2 Hz, H2), 6.80 (2H, d, *J* = 8.2 Hz, H12), 6.76 (1H, d, *J* = 3.7 Hz, H9), 4.25 – 4.14 (1H, m, H4), 4.01 – 3.92 (1H, m, H4), 3.61 – 3.48 (2H, m, H14), 1.88 (6H, s, H1, H13), 1.83 – 1.68 (2H, m, H5), 1.43 – 1.34 (1H, m, H15), 1.33 – 1.22 (3H, m, H6, H15), 0.94 (2H, sext, *J* = 7.4 Hz, H16), 0.85 (3H, t, *J* = 7.4 Hz, H7), 0.64 (3H, t, *J* = 7.4 Hz, H17);

^{13}C NMR (126 MHz, C_6D_6) δ_C 183.3, 143.9, 143.5, 137.7, 136.7, 130.0, 129.8, 128.6, 128.5, 127.4, 123.5, 122.9, 118.0, 52.1, 51.6, 32.2, 30.9, 21.19, 21.15, 20.3, 20.1, 14.0, 13.8;

HRMS (ESI) m/z: $[M + Na]^+$ Calcd for $C_{27}H_{35}O_5N_3NaS_2$ 568.1910; Found 568.1908.

_N,N'_-(5-Formyl-1*H*-pyrrole-2,3-diyl)bis(_N_-butyl-4-methylbenzenesulfonamide), 4a

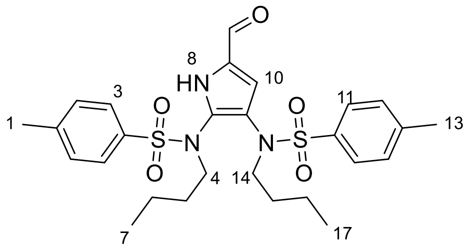

Synthesized from *N,N'*-(ethyne-1,2-diyl)bis(*N*-butyl-4-methylbenzenesulfonamide) (477 mg, 1.00 mmol, 1.0 eq.), isoxazole (0.13 mL, 2.03 mmol, 2.0 eq.), chloro[tris(2,4-di-tert-butylphenyl)phosphite]gold(I) (44 mg, 5.0 μmol, 5 mol%) and silver bis(trifluoromethanesulfonyl)imide (20 mg, 0.05 mmol, 5 mol%) in DCE (anhy., 2.0 mL) with general procedure **2.3** at room temperature for 2 h. Column chromatography (silica gel, 20% EtOAc/pentane) afforded the title compound (85 mg, 0.16 mmol, 16%) as a yellow solid. Crystals suitable for X-ray diffraction were grown by vapor diffusion of pentane into a concentrated solution of the title compound in benzene.

m.p. 156 °C;

R_f 0.24 (20% EtOAc/pentane);

IR (thin film, ν_{max} / cm^{-1}) 3232, 2961, 2873, 1659, 1087, 1350, 1163, 1090, 814, 665;

^{1}H NMR (400 MHz, C_6D_6) δ_H 10.61 (1H, s, H8), 9.06 (1H, s, H9), 7.80 (2H, d, *J* = 8.3 Hz, H3), 7.66 (2H, d, *J* = 8.3 Hz, H11), 6.84 – 6.74 (4H, m, H2, H12), 6.29 (1H, d, *J* = 2.8 Hz, H10), 4.05 – 3.96 (2H, m, H4), 3.38 – 3.29 (2H, m, H14), 1.93 (3H, s, H1), 1.86 (3H, s, H13), 1.67 (2H, quin, *J* = 7.7 Hz, H5), 1.37 – 1.28 (2H, m, H15), 1.28 – 1.19 (2H, m, H6), 1.01 (2H, sext, *J* = 7.4 Hz, H16), 0.79 (3H, t, *J* = 7.4 Hz, H7), 0.73 (3H, t, *J* = 7.4 Hz, H17);

^{13}C NMR (101 MHz, C_6D_6) δ 178.5, 144.0, 143.5, 137.4, 137.1, 132.3, 129.9, 129.7, 128.5, 128.1, 127.7, 120.5, 118.2, 51.9, 50.7, 32.0, 30.4, 21.18, 21.16, 20.2, 20.0, 13.91, 13.85;

HRMS (ESI) m/z: $[M + H]^+$ Calcd for $C_{27}H_{36}O_5N_3S_2$ 546.2091; Found 546.2086.

_N,N'_-(4-Acetyl-5-methyl-1*H*-pyrrole-2,3-diyl)bis(_N_-butyl-4-methylbenzenesulfonamide), 2b

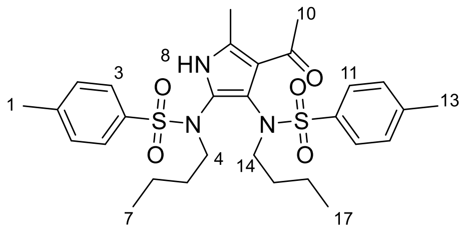

0.10 mmol scale: Synthesized from *N,N'*-(ethyne-1,2-diyl)bis(*N*-butyl-4-methylbenzenesulfonamide) (48 mg, 0.10 mmol, 1.0 eq.), 3,5-dimethylisoxazole (20 μL, 0.20 mmol, 2.0 eq.), chloro[tris(2,4-di-tert-butylphenyl)phosphite]gold(I) (4.4 mg, 5.0 μmol, 5 mol%) and silver bis(trifluoromethanesulfonyl)imide (2.0 mg, 5.2 μmol, 5 mol%) in DCE (anhy., 0.2 mL) with general procedure **2.3** at room temperature for 2 h. Upon completion, the reaction mixture was treated with HCl (1 M in Et_2O, 0.1 mL) for 30 min before it was concentrated *in vacuo* and purified by column chromatography (silica gel, 20% → 40% EtOAc/pentane) to give the title compound (49 mg, 85 μmol, 85%) as a beige solid.

1 mmol scale: Synthesized from *N,N'*-(ethyne-1,2-diyl)bis(*N*-butyl-4-methylbenzenesulfonamide) (477 mg, 1.00 mmol, 1.0 eq.), 3,5-dimethylisoxazole (0.20 mL, 2.00 mmol, 2.0 eq.), chloro[tris(2,4-di-tert-

butylphenyl)phosphite]gold(I) (44 mg, 5.0 μmol, 5 mol%) and silver bis(trifluoromethanesulfonyl)imide (20 mg, 0.05 mmol, 5 mol%) in DCE (anhy., 2.0 mL) with general procedure **2.3** at room temperature for 3 h. Upon completion, the reaction mixture was treated with HCl (1 M in Et_2O, 1.0 mL) 30 min before it was concentrated *in vacuo* and purified by column chromatography (silica gel, 20% → 40% EtOAc/pentane) afforded the title compound (497 mg, 0.87 mmol, 87%) as a brown solid.

m.p. 130 °C (decomp.);

R$_f$ 0.27 (40% EtOAc/pentane);

IR (thin film, ν_{max} / cm^{-1}) 3308, 2960, 2931, 2874, 1655, 1444, 1339, 1158, 1089, 910, 814, 731, 674;

^{1}H NMR (400 MHz, $CDCl_3$) δ_H 8.55 (1H, s, H8), 7.74 (2H, d, *J* = 8.2 Hz, H3), 7.60 (2H, d, *J* = 8.2 Hz, H11), 7.31 (2H, d, *J* = 8.2 Hz, H2), 7.23 (2H, d, *J* = 8.2 Hz, H12), 3.68 – 3.57 (1H, m, H4), 3.54 – 3.43 (1H, m, H4), 3.36 – 3.20 (2H, m, H14), 2.45 (3H, s, H10), 2.42 (3H, s, H1), 2.40 (3H, s, H13), 2.17 (3H, s, H9), 1.58 – 1.46 (2H, m, H5), 1.26 – 1.09 (4H, m, H6, H15), 1.05 – 0.93 (2H, m, H16), 0.86 (3H, t, *J* = 7.3 Hz, H7), 0.73 (3H, t, *J* = 7.3 Hz, H17);

^{13}C NMR (101 MHz, $CDCl_3$) δ_C 193.6, 144.3, 143.2, 137.0, 136.8, 130.5, 129.9, 129.4, 128.2, 127.8, 123.3, 120.2, 118.0, 52.0, 51.7, 31.5, 30.7, 30.6, 21.70, 21.67, 20.2, 19.8, 15.6, 13.9, 13.8.

HRMS (ESI) m/z: [M - H]$^-$ Calcd for $C_{29}H_{38}O_5N_3S_2$ 572.2258; Found 572.2263.

***N,N'*-(5-Methyl-7-methylene-2,7-dihydro-1,4-oxazepine-2,3-diyl)bis(*N*-butyl-4-methylbenzenesulfonamide), 5b**

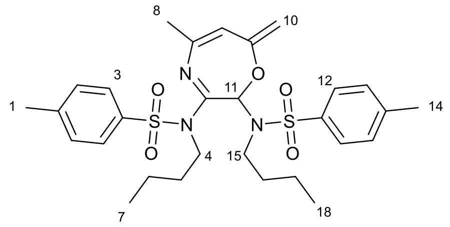

Synthesized from *N,N'*-(ethyne-1,2-diyl)bis(*N*-butyl-4-methylbenzenesulfonamide) (48 mg, 0.10 mmol, 1.0 eq.), 3,5-dimethylisoxazole (20 μL, 0.20 mmol, 2.0 eq.), chloro[tris(2,4-di-tert-butylphenyl)phosphite]gold(I) (4.4 mg, 5.0 μmol, 5 mol%) and silver bis(trifluoromethanesulfonyl)imide (2.0 mg, 5.2 μmol, 5 mol%) in DCE (anhy., 0.2 mL) with general procedure **2.4** at room temperature for 2 h. Column chromatography (silica gel, 20% → 40% EtOAc/pentane) to give the title compound (21 mg, 0.37 mmol, 37%) as a white solid. *N,N'*-(4-acetyl-5-methyl-1*H*-pyrrole-2,3-diyl)bis(*N*-butyl-4-methylbenzenesulfonamide) (26 mg, 0.45 mmol, 45%) was also collected as a brown solid.

m.p. 145 °C (decomp.);

R$_f$ 0.22 (20% EtOAc/pentane);

IR (thin film, ν_{max} / cm^{-1}) 2961, 2932, 2874, 2280, 1713, 1586, 1353, 1166, 1088, 812, 667;

^{1}H NMR (400 MHz, C_6D_6) δ_H 8.13 (2H, d, *J* = 8.3 Hz, H12), 7.79 (2H, d, *J* = 7.7 Hz, H3), 6.78 – 6.71 (4H, m, H2, H13), 6.06 – 6.01 (1H, m, H9), 5.28 – 5.23 (1H, s, H10), 4.40 (1H, s, H10), 4.21 – 4.01 (2H, m,

H15), 3.46 (1H, brs., H11), 3.26 (1H, ddd, *J* = 14.8, 11.0, 5.4 Hz, H4), 2.89 (1H, brs., H4), 1.94 – 1.69 (13H, m, H1, H5, H8, H14, H16), 1.33 – 1.12 (4H, m, H6, H17), 0.87 (3H, t, *J* = 7.4 Hz, H18), 0.82 (3H, t, *J* = 7.4 Hz, H7);

^{13}C NMR (101 MHz, C_6D_6) δ_C 201.9, 165.3, 155.7, 144.2, 143.6, 137.2, 137.1, 129.9, 129.4, 129.3, 128.17, 103.5, 65.2, 59.5, 48.4, 47.0, 32.7, 32.3, 28.3, 21.14, 21.09, 20.5, 20.2, 13.9, 13.8.

HRMS (ESI) m/z: $[M + H]^+$ Calcd for $C_{29}H_{40}O_5N_3S_2$ 574.2404; Found 574.2402.

_N,N'_-(4-Acetyl-1_H_-pyrrole-2,3-diyl)bis(_N_-butyl-4-methylbenzenesulfonamide), 2c

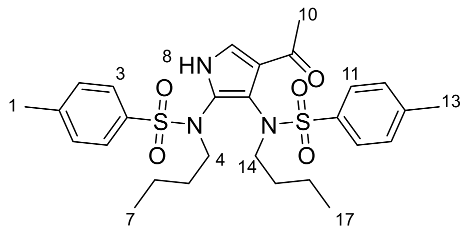

Synthesized from *N,N'*-(ethyne-1,2-diyl)bis(*N*-butyl-4-methylbenzenesulfonamide) (48 mg, 0.10 mmol, 1.0 eq.), 5-methylisoxazole (16.5 µL, 0.20 mmol, 2.0 eq.), chloro[tris(2,4-di-tert-butyl-phenyl)phosphite]gold(I) (4.4 mg, 5.0 µmol, 5 mol%) and silver bis(trifluoromethane-sulfonyl)imide (2.0 mg, 5.2 µmol, 5 mol%) in DCE (anhy., 0.2 mL) with general procedure **2.3** at room temperature for 2 h. Upon completion, the reaction was treated with HCl (1.0 M in Et_2O, 0.1 mL) for 30 min at room temperature, after which column chromatography (silica gel, 20% → 40% EtOAc/pentane) afforded the title compound (38 mg, 68 µmol, 67%) as a beige solid.

m.p. 103 °C;

R_f 0.29 (40% EtOAc/pentane);

IR (thin film, ν_{max} / cm^{-1}) 3315, 2960, 2874, 1669, 1341, 1159, 1121, 910, 814, 731, 670;

^{1}H NMR (400 MHz, $CDCl_3$) δ_H 9.30 (1H, s, H8), 7.81 (2H, d, *J* = 8.2 Hz, H3), 7.51 (2H, d, *J* = 8.3 Hz, H11), 7.31 (2H, d, *J* = 8.2 Hz, H2), 7.23 – 7.16 (3H, m, H9, H12), 4.00 – 3.89 (1H, m, H4), 3.84 – 3.74 (1H, m, H4), 3.42 – 3.31 (1H, m, H14), 3.26 – 3.16 (1H, m, H14), 2.40 (3H, s, H1), 2.39 (3H, s, H13), 2.08 (3H, s, H10), 1.63 (2H, quin, *J* = 7.6 Hz, H5), 1.35 – 1.24 (2H, m, H6), 1.22 – 1.12 (1H, m, H15), 1.05 – 0.94 (3H, m, H15, H16), 0.88 (3H, t, *J* = 7.4 Hz, H7), 0.70 (3H, t, *J* = 7.1 Hz, H17);

^{13}C NMR (101 MHz, $CDCl_3$) δ_C 190.1, 144.3, 143.1, 136.6, 136.1, 129.9, 129.1, 128.4, 127.8, 127.4, 122.1, 121.6, 116.7, 51.8, 51.3, 31.8, 30.5, 27.3, 21.7 (2C), 20.1, 19.8, 13.9, 13.7;

HRMS (ESI) m/z: $[M + Na]^+$ Calcd for $C_{28}H_{37}N_3NaO_5S_2$ 582.2067; Found 582.2068.

***N,N'*-(5-((4-Methylphenyl)sulfonamido)-4-pivaloyl-1*H*-pyrrole-2,3-diyl)bis(*N*-butyl-4-methylbenzenesulfonamide), 2d**

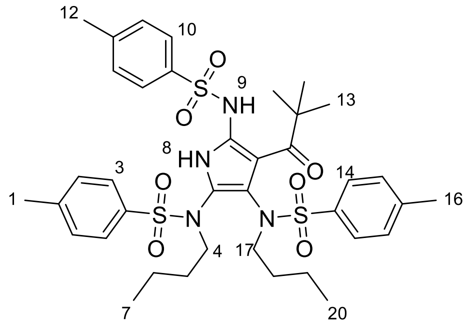

Synthesized from *N,N'*-(ethyne-1,2-diyl)bis(*N*-butyl-4-methylbenzenesulfonamide) (48 mg, 0.10 mmol, 1.0 eq.), *N*-(5-(*tert*-butyl)isoxazol-3-yl)-4-methylbenzenesulfonamide (60 mg, 0.20 mmol, 2.0 eq.), chloro[tris(2,4-di-tert-butyl-phenyl)phosphite]gold(I) (4.4 mg, 5.0 µmol, 5 mol%) and silver bis(trifluoromethane-sulfonyl)imide (2.0 mg, 5.2 µmol, 5 mol%) in DCE (anhy., 0.2 mL) with general procedure **2.3** at room temperature for 2 h. Column chromatography (silica gel, 5% → 10% → 20% EtOAc/pentane) afforded the title compound (52 mg, 0.67 mmol, 67%) as a white solid;

m.p. 93 °C (decomp.);

R$_f$ 0.13 (20% EtOAc/pentane);

IR (thin film, ν_{max} / cm^{-1}) 3662, 2980, 2884, 1598, 1461, 1346, 1163, 1089, 910, 732, 668;

^{1}H NMR (400 MHz, $CDCl_3$) δ_H 8.67 (1H, s, H8), 7.86 (1H, s, H9), 7.71 (2H, d, *J* = 8.4 Hz, H3), 7.64 (2H, d, *J* = 8.4 Hz, H14), 7.46 (2H, d, *J* = 8.1 Hz, H10), 7.38 – 7.29 (4H, m, H2, H15), 7.18 (2H, d, *J* = 8.1 Hz, H11), 3.78 – 3.65 (1H, m, H4), 3.62 – 3.51 (1H, m, H4), 3.30 – 3.19 (1H, m, H17), 2.45 (3H, s, H1), 2.42 (3H, s, H16), 2.35 (3H, s, H12), 1.85 – 1.69 (3H, m, H17, H5), 1.33 – 1.25 (2H, m, H6), 0.98 – 0.84 (13H, m, H7, H13, H18), 0.80 – 0.68 (5H, m, H19, H20), 0.66 – 0.51 (1H, m, H18);

^{13}C NMR (101 MHz, $CDCl_3$) δ 208.7, 145.1, 144.8, 143.7, 137.1, 136.2, 135.3, 130.6, 130.2, 129.7, 127.9, 127.4, 127.2, 123.7, 117.6, 117.2, 114.6, 51.4, 50.8, 44.7, 31.4, 30.4, 27.9, 21.8, 21.7, 21.6, 20.4, 19.4, 14.0, 13.8;

HRMS (ESI) m/z: $[M + H]^+$ Calcd for $C_{38}H_{51}N_4O_7S_3$ 771.2914; Found 771.2911;

Methyl 2-(4,5-bis((*N*-butyl-4-methylphenyl)sulfonamido)-1*H*-pyrrol-3-yl)-2-oxoacetate, 2e

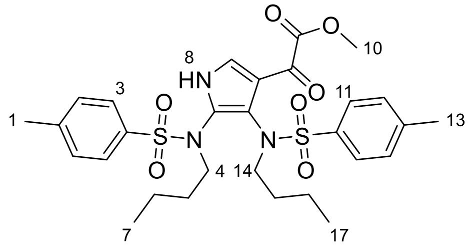

Synthesized from *N,N'*-(ethyne-1,2-diyl)bis(*N*-butyl-4-methylbenzenesulfonamide) (48 mg, 0.10 mmol, 1.0 eq.), methyl isoxazole-5-carboxylate (26 mg, 0.20 mmol, 2.0 eq.), chloro[tris(2,4-di-tert-butyl-phenyl)phosphite]gold(I) (4.4 mg, 5.0 µmol, 5 mol%) and silver bis(trifluoromethane-sulfonyl)imide (2.0 mg, 5.2 µmol, 5 mol%) in DCE (anhy., 0.2 mL) with general procedure **2.3** at 80 °C for 2 h. Column chromatography (silica gel, 20% → 40% EtOAc/pentane) afforded the title compound (44 mg, 73 µmol, 72%) as a brown oil;

R$_f$ 0.38 (40% EtOAc/pentane);

IR (thin film, ν_{max} / cm^{-1}) 3290, 2959, 1736, 1670, 1339, 1161, 1090, 910, 732, 666;

^{1}H NMR (400 MHz, $CDCl_3$) δ_H 9.56 (1H, s, H8), 7.78 (2H, d, *J* = 8.2 Hz, H3), 7.67 (1H, d, *J* = 3.9 Hz, H9), 7.46 (2H, d, *J* = 8.2 Hz, H11), 7.31 (2H, d, *J* = 8.2 Hz, H2), 7.17 (2H, d, *J* = 8.2 Hz, H12), 4.05 – 3.96 (1H, m, H4), 3.92 – 3.83 (1H, m, H4), 3.79 (3H, s, H10), 3.45 – 3.35 (1H, m, H14), 3.20 – 3.10 (1H, m, H14), 2.40 (3H, s, H1), 2.37 (3H, s, H13), 1.66 (2H, quin, *J* = 7.7 Hz, H5), 1.38 – 1.28 (2H, m, H6), 1.23 – 1.14 (1H, m, H15), 1.04 – 0.95 (3H, m, H15, H16), 0.90 (3H, t, *J* = 7.4 Hz, H7), 0.70 (3H, t, *J* = 7.1 Hz, H17);

^{13}C NMR (101 MHz, $CDCl_3$) δ_C 176.2, 162.8, 144.4, 143.4, 136.3, 135.6, 129.9, 129.2, 128.4, 128.2, 127.9, 126.7, 117.3, 116.8, 52.8, 51.9, 51.1, 32.0, 30.5, 21.70, 21.66, 20.1, 19.8, 13.9, 13.7;

HRMS (ESI) m/z: $[M + H]^+$ Calcd for $C_{29}H_{38}N_3O_7S_2$ 604.2146; Found 604.2144;

Methyl 3-acetyl-4,5-bis((N-butyl-4-methylphenyl)sulfonamido)-1*H*-pyrrole-2-carboxylate, 2f

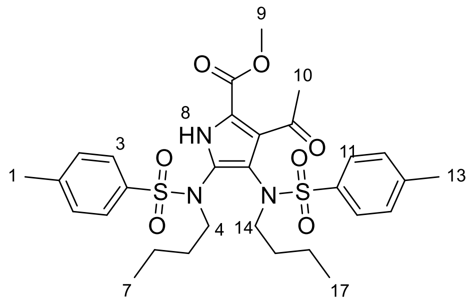

Synthesized from *N*,*N'*-(ethyne-1,2-diyl)bis(*N*-butyl-4-methylbenzenesulfonamide) (48 mg, 0.10 mmol, 1.0 eq.), methyl 5-methylisoxazole-3-carboxylate (29 mg, 0.20 mmol, 2.0 eq.), chloro[tris(2,4-di-tert-butyl-phenyl)phosphite]gold(I) (4.4 mg, 5.0 μmol, 5 mol%) and silver bis(trifluoromethane-sulfonyl)imide (2.0 mg, 5.2 μmol, 5 mol%) in DCE (anhy., 0.2 mL) with general procedure **2.3** at 80 °C for 2 h. Column chromatography (silica gel, 10% → 20% EtOAc/pentane) afforded the title compound (22 mg, 36 μmol, 36%) as a yellow oil;

R_f 0.11 (20% EtOAc/pentane);

IR (thin film, ν_{max} / cm^{-1}) 3268, 2960, 1691, 1499, 1453, 1345, 1285, 1162, 1090, 859, 708;

^{1}H NMR (400 MHz, C_6D_6) δ_H 9.93 (1H, s, H8), 7.85 (2H, d, *J* = 8.2 Hz, H3), 7.73 (2H, d, *J* = 8.1 Hz, H11), 6.79 (2H, d, *J* = 8.2 Hz, H2), 6.68 (2H, d, *J* = 8.1 Hz, H12), 4.26 – 4.16 (1H, m, H4), 4.14 – 4.01 (1H, m, H4), 3.52 – 3.43 (1H, m, H14), 3.42 – 3.33 (1H, m, H14), 3.25 (3H, s, H9), 2.56 (3H, s, H10), 1.85 (3H, s, H1), 1.82 – 1.70 (5H, m, H5, H13), 1.41 – 1.28 (4H, m, H6, H15), 0.98 – 0.85 (2H, m, H16), 0.82 (3H, t, *J* = 7.4 Hz, H7), 0.64 (3H, t, *J* = 7.3 Hz, H17);

^{13}C NMR (101 MHz, C_6D_6) δ_C 198.2, 159.7, 144.2, 143.6, 137.5, 137.3, 131.0, 130.1, 129.7, 128.7, 128.62, 128.59, 117.2, 115.6, 51.6, 51.5, 51.3, 32.4, 31.9, 30.4, 21.1 (2C), 20.1, 20.0, 13.9, 13.8;

HRMS (ESI) m/z: $[M + H]^+$ Calcd for $C_{30}H_{40}N_3O_7S_2$ 618.2302; Found 618.2299;

N,N'-(4-Benzoyl-5-phenyl-1H-pyrrole-2,3-diyl)bis(N-butyl-4-methylbenzenesulfonamide), 2g

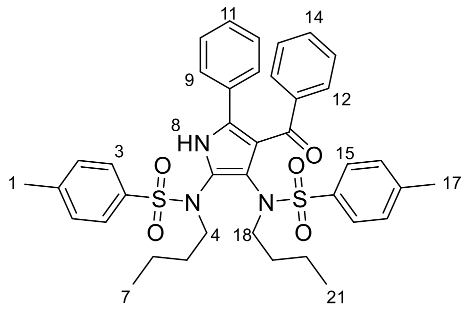

Synthesized from N,N'-(ethyne-1,2-diyl)bis(N-butyl-4-methylbenzenesulfonamide) (48 mg, 0.10 mmol, 1.0 eq.), 3,5-diphenylisoxazole (45 mg, 0.20 mmol, 2.0 eq.), chloro[tris(2,4-di-tert-butyl-phenyl)phosphite]gold(I) (4.4 mg, 5.0 μmol, 5 mol%) and silver bis(trifluoromethane-sulfonyl)imide (2.0 mg, 5.2 μmol, 5 mol%) in DCE (anhy., 0.2 mL) with general procedure **2.3** at room temperature for 2 h. Column chromatography (silica gel, 20% EtOAc/pentane) afforded the title compound (44 mg, 63 μmol, 63%) as a beige solid. Single crystals suitable for X-ray crystallography were grown by vapor diffusion of pentane into a concentrated solution of the title compound in DCM.

m.p. 218 °C (decomp.);

R_f 0.39 (20% EtOAc/pentane);

IR (thin film, ν_{max} / cm^{-1}) 3298, 2961, 2874, 1647, 1456, 1338, 1158, 1090, 908, 730, 696, 662;

^{1}H NMR (400 MHz, $CDCl_3$) δ_H 8.99 (1H, s, H8), 7.74 (2H, d, J = 8.2 Hz, H15), 7.46 (2H, d, J = 7.1 Hz, H12), 7.39 (2H, d, J = 8.2 Hz, H3), 7.31 (2H, d, J = 8.2 Hz, H16), 7.28 – 7.23 (1H, m, H14), 7.13 – 7.05 (7H, m, H9, H10, H11, H13), 6.76 (2H, d, J = 8.2 Hz, H2), 4.14 – 4.02 (1H, m, H18), 3.99 – 3.88 (1H, m, H18), 3.38 (1H, td, J = 12.4, 5.2 Hz, H4), 3.28 (1H, td, J = 12.4, 5.2 Hz, H4), 2.42 (3H, s, H17), 2.10 (3H, s, H1), 1.81 (2H, quin, J = 7.9 Hz, H19), 1.42 (2H, sext, J = 7.3 Hz, H20), 1.32 – 1.21 (1H, m, H5), 1.17 – 1.05 (1H, m, H5), 1.02 – 0.91 (5H, m, H6, H21), 0.73 (3H, t, J = 7.3 Hz, H7);

^{13}C NMR (101 MHz, $CDCl_3$) δ_C 191.6, 144.3, 143.0, 138.2, 136.7, 136.3, 132.2, 131.5, 131.1, 130.2, 129.9, 129.2, 128.5, 128.4, 128.2, 128.1, 127.7, 127.6, 126.4, 119.0, 118.7, 53.1, 51.7, 32.2, 30.7, 21.7, 21.4, 20.1, 19.9, 14.1, 13.7;

HRMS (ESI) m/z: $[M - H]^-$ Calcd for $C_{39}H_{42}N_3O_5S_2$ 696.2571; Found 696.2571.

N,N'-(4-Pentanoyl-5-phenyl-1H-pyrrole-2,3-diyl)bis(N-butyl-4-methylbenzenesulfonamide), 2h

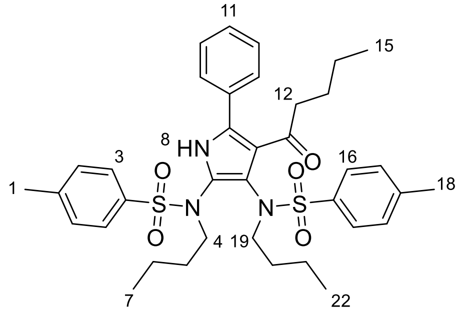

Synthesized from *N*,*N'*-(ethyne-1,2-diyl)bis(*N*-butyl-4-methylbenzenesulfonamide) (48 mg, 0.10 mmol, 1.0 eq.), 5-butyl-3-phenylisoxazole (41 mg, 0.20 mmol, 2.0 eq.), chloro[tris(2,4-di-tert-butyl-phenyl)phosphite]gold(I) (4.4 mg, 5.0 μmol, 5 mol%) and silver bis(trifluoromethane-sulfonyl)imide (2.0 mg, 5.2 μmol, 5 mol%) in DCE (anhy., 0.2 mL) with general procedure **2.3** at room temperature for 2 h. Column chromatography (silica gel, 10% → 20% EtOAc/pentane) afforded the title compound (57 mg, 84 μmol, 83%) as a white solid;

m.p. 164 °C;

R_f 0.53 (20% EtOAc/pentane);

IR (thin film, ν_{max} / cm^{-1}) 3292, 2959, 1667, 1455, 1337, 1159, 1090, 909, 730, 665;

^{1}H NMR (400 MHz, $CDCl_3$) δ_H 8.79 (1H, s, H8), 7.73 (2H, d, *J* = 8.2 Hz, H16), 7.59 (2H, d, *J* = 8.2 Hz, H3), 7.46 – 7.40 (3H, m, H10, H11), 7.39 – 7.35 (2H, m, H12), 7.30 (2H, d, *J* = 8.2 Hz, H17), 7.20 (2H, d, *J* = 8.2 Hz, H2), 3.82 (2H, td, *J* = 7.2, 2.8 Hz, H19), 3.38 – 3.23 (2H, m, H4), 2.41 (3H, s, H18), 2.38 (3H, s, H1), 2.30 – 2.20 (1H, m, H12), 2.06 – 1.94 (1H, m, H12), 1.66 (2H, quin, *J* = 7.9 Hz, H20), 1.35 – 1.17 (6H, m, H5, H13, H21), 1.05 – 0.95 (4H, m, H6, H14), 0.92 (3H, t, *J* = 7.4 Hz, H22), 0.76 – 0.67 (6H, m, H7, H15);

^{13}C NMR (101 MHz, $CDCl_3$) δ_C 198.6, 144.3, 143.2, 137.0, 136.7, 132.1, 131.6, 129.9, 129.3, 129.1, 128.9 (2C), 128.2, 127.8, 125.5, 120.9, 117.5, 52.4, 51.5, 42.4, 31.8, 30.6, 25.9, 22.1, 21.7, 21.6, 20.1, 19.9, 13.9, 13.9, 13.8;

HRMS (ESI) m/z: $[M + H]^+$ Calcd for $C_{37}H_{48}N_3O_5S_2$ 678.3030; Found 638.3027;

N,N'-(4-(Cyclohexanecarbonyl)-5-phenyl-1H-pyrrole-2,3-diyl)bis(N-butyl-4-methylbenzenesulfonamide), 2i

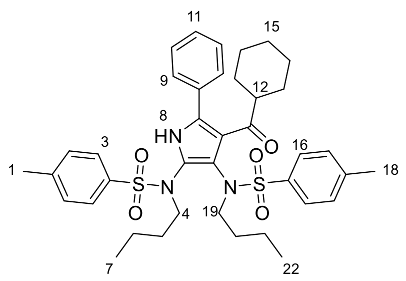

Synthesized from *N*,*N'*-(ethyne-1,2-diyl)bis(*N*-butyl-4-methylbenzenesulfonamide) (48 mg, 0.10 mmol, 1.0 eq.), 5-cyclohexyl-3-phenylisoxazole (46 mg, 0.20 mmol, 2.0 eq.), chloro[tris(2,4-di-tert-butyl-phenyl)phosphite]gold(I) (4.4 mg, 5.0 μmol, 5 mol%) and silver bis(trifluoromethane-sulfonyl)imide (2.0 mg, 5.2 μmol, 5 mol%) in DCE (anhy., 0.2 mL) with general procedure **2.2** at room temperature for 2 h. Column chromatography (silica gel, 5% → 10% → 20% EtOAc/pentane) afforded the title compound (48 mg, 68 μmol, 68%) as a white solid;

m.p. 173 °C (decomp.);

R$_f$ 0.41 (20% EtOAc/pentane);

IR (thin film, ν_{max} / cm^{-1}) 3290, 2931, 1660, 1335, 1158, 1089, 908, 730;

^{1}H NMR (400 MHz, $CDCl_3$) δ_H 8.69 (1H, s, H8), 7.73 (2H, d, *J* = 8.2 Hz, H16), 7.66 (2H, d, *J* = 8.2 Hz, H3), 7.47 – 7.40 (5H, m, H9, H10, H11), 7.29 (2H, d, *J* = 8.2 Hz, H17), 7.23 (2H, d, *J* = 8.2 Hz, H2), 3.79 – 3.67 (1H, m, H19), 3.64 – 3.52 (1H, m, H19), 3.35 – 3.25 (1H, m, H4), 3.24 – 3.14 (1H, m, H4)., 2.47 – 2.36 (7H, m, H1, H12, H18), 1.66 – 1.44 (m, 7H, H13, H14, H15, H20), 1.32 – 1.16 (5H, m, H5, H15, H21), 1.11 – 0.92 (4H, m, H6, H13), 0.88 (3H, t, *J* = 7.3 Hz, H22), 0.85 – 0.70 (5H, m, H14, H7);

^{13}C NMR (101 MHz, $CDCl_3$) δ_C 201.3, 144.4, 143.2, 137.2, 136.7, 132.4, 132.2, 129.9, 129.3, 129.2, 129.1, 128.8, 128.4, 127.8, 124.9, 119.8, 118.3, 51.6, 51.5, 49.1, 31.6, 30.6, 30.0, 28.5, 26.1, 25.9, 25.6, 21.7, 21.6, 20.1, 19.8, 13.9, 13.8; *Extra peaks are observed due to diastereotopic cyclohexyl group.*

HRMS (ESI) m/z: [M + H]$^+$ Calcd for $C_{39}H_{50}N_3O_5S_2$ 704.3186; Found 704.3182;

***N,N'*-(4-(Cyclohex-1-ene-1-carbonyl)-5-phenyl-1*H*-pyrrole-2,3-diyl)bis(*N*-butyl-4-methylbenzenesulfonamide), 2j**

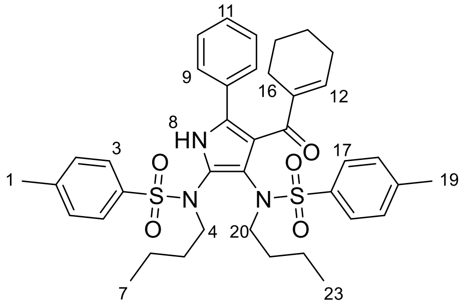

Synthesized from *N,N'*-(ethyne-1,2-diyl)bis(*N*-butyl-4-methylbenzenesulfonamide) (48 mg, 0.10 mmol, 1.0 eq.), 5-(cyclohex-1-en-1-yl)-3-phenylisoxazole (46 mg, 0.20 mmol, 2.0 eq.), chloro[tris(2,4-di-tert-butyl-phenyl)phosphite]gold(I) (4.4 mg, 5.0 μmol, 5 mol%) and silver bis(trifluoromethane-sulfonyl)imide (2.0 mg, 5.2 μmol, 5 mol%) in DCE (anhy., 0.2 mL) with general procedure **2.3** at 80 °C for 2 h. Column chromatography (silica gel, 5% → 10% → 20% EtOAc/pentane) afforded the title compound (42 mg, 60 μmol, 59%) as a white solid;

m.p. 218 °C (decomp.);

R$_f$ 0.22 (10% EtOAc/pentane);

IR (thin film, ν_{max} / cm^{-1}) 3297, 2959, 2932, 1630, 1336, 1155, 1090, 910, 731, 664;

^{1}H NMR (400 MHz, $CDCl_3$) δ_H 8.91 (1H, s, H8), 7.71 (2H, d, *J* = 8.3 Hz, H17), 7.54 (2H, d, *J* = 8.2 Hz, H3), 7.37 – 7.27 (5H, m, H10, H11, H18), 7.20 – 7.13 (2H, m, H9), 7.11 (2H, d, *J* = 8.2 Hz, H2), 6.36 (1H, t, *J* = 3.8 Hz, H12), 4.06 – 3.96 (1H, m, H20), 3.95 – 3.83 (1H, m, H20), 3.31 (1H, td, *J* = 12.5, 5.2 Hz, H4), 3.16 (1H, td, *J* = 12.5, 5.2 Hz, H4), 2.40 (3H, s, H19), 2.35 (3H, s, H1), 2.11 – 1.98 (1H, m, H10), 1.97 – 1.82 (2H, m, H13, H16), 1.76 (2H, quin, *J* = 7.7 Hz, H21), 1.59 – 1.48 (1H, m, H16), 1.47 – 1.30 (5H, m, H14, H15, H22), 1.29 – 1.15 (2H, m, H5, H14), 1.16 – 1.00 (1H, m, H5), 1.00 – 0.86 (5H, m, H6, H23), 0.71 (3H, t, *J* = 7.3 Hz, H7);

^{13}C NMR (101 MHz, $CDCl_3$) δ_C 193.6, 144.5, 144.2, 143.0, 139.3, 136.9, 136.8, 132.2, 130.9, 129.9, 129.3, 128.7, 128.3, 128.2, 128.0, 127.7, 125.8, 119.4, 118.6, 52.7, 51.6, 32.0, 30.5, 26.1, 23.6, 21.9, 21.7 (2C), 21.6, 20.1, 19.9, 14.0, 13.7;

HRMS (ESI) m/z: $[M + H]^+$ Calcd for $C_{39}H_{48}N_3O_5S_2$ 702.3030; Found 702.3027;

***N*,*N'*-(4-(4-Fluorobenzoyl)-5-phenyl-1*H*-pyrrole-2,3-diyl)bis(*N*-butyl-4-methylbenzenesulfonamide), 2k**

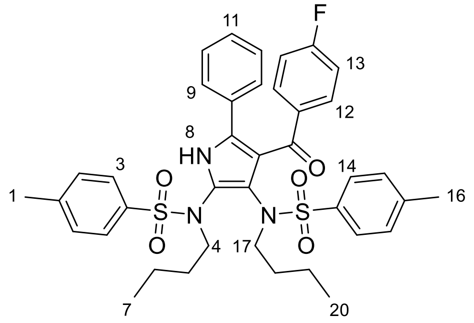

Synthesized from *N*,*N'*-(ethyne-1,2-diyl)bis(*N*-butyl-4-methylbenzenesulfonamide) (48 mg, 0.10 mmol, 1.0 eq.), 5-(4-fluorophenyl)-3-phenylisoxazole (49 mg, 0.20 mmol, 2.0 eq.), chloro[tris(2,4-di-tert-butyl-phenyl)phosphite]gold(I) (4.4 mg, 5.0 μmol, 5 mol%) and silver bis(trifluoromethane-sulfonyl)imide (2.0 mg, 5.2 μmol, 5 mol%) in DCE (anhy., 0.2 mL) with general procedure **2.3** at 80 °C for 2 h. Column chromatography (silica gel, 10% EtOAc/pentane) afforded the title compound (48 mg, 67 μmol, 68%) as a white solid.

m.p. 185 °C (decomp);

R_f 0.13 (10% EtOAc/pentane);

IR (thin film, ν_{max} / cm^{-1}) 3293, 2963, 1650, 1597, 1338, 1151, 1090, 772, 732, 667;

^{1}H NMR (500 MHz, $CDCl_3$) δ_H 9.03 (1H, s, H8), 7.73 (2H, d, *J* = 8.2 Hz, H3), 7.48 (2H, dd, *J* = 8.7, 5.5 Hz, H12), 7.40 (2H, d, *J* = 8.2 Hz, H14), 7.31 (2H, d, *J* = 8.2 Hz, H2), 7.15 – 7.11 (3H, m, H9, H11), 7.10 – 7.06 (2H, m, H10), 6.81 (2H, d, *J* = 8.2 Hz, H15), 6.79 – 6.73 (2H, m, H13), 4.09 – 4.00 (1H, m, H17), 3.96 – 3.86 (1H, m, H17), 3.38 (1H, td, *J* = 12.5, 5.2 Hz, H4), 3.27 (1H, td, *J* = 12.5, 5.2 Hz, H4), 2.42 (3H, s, H1), 2.14 (3H, s, H16), 1.80 (2H, quin, *J* = 7.8 Hz, H18), 1.41 (2H, sext, *J* = 7.1 Hz, H19), 1.31 – 1.22 (1H, m, H5), 1.16 – 1.07 (1H, m, H5), 1.01 – 0.92 (5H, m, H6, H20), 0.73 (3H, t, *J* = 7.3 Hz, H7);

^{13}C NMR {^{19}F} (126 MHz, $CDCl_3$) δ_C 190.0, 165.2, 144.4, 143.1, 136.6, 136.3, 134.6, 132.6, 131.4, 130.9, 130.0, 129.2, 128.6, 128.40, 128.36, 128.1, 127.7, 126.5, 118.9, 118.5, 114.7, 53.0, 51.6, 32.1, 30.6, 21.7, 21.5, 20.1, 19.9, 14.0, 13.7;

^{19}F NMR (471 MHz, $CDCl_3$) δ_F -106.55.

HRMS (ESI) m/z: $[M - H]^-$ Calcd for $C_{39}H_{41}FN_3O_5S_2$ 714.2477; Found 714.2472;

***N,N'*-(4-(4-Cyanobenzoyl)-5-phenyl-1*H*-pyrrole-2,3-diyl)bis(*N*-butyl-4-methylbenzenesulfonamide), 2l**

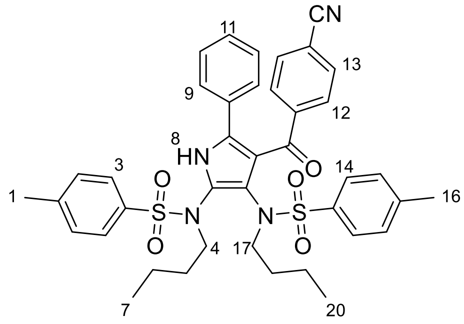

Synthesized from *N,N'*-(ethyne-1,2-diyl)bis(*N*-butyl-4-methylbenzenesulfonamide) (48 mg, 0.10 mmol, 1.0 eq.), 4-(3-phenylisoxazol-5-yl)benzonitrile (50 mg, 0.20 mmol, 2.0 eq.), chloro[tris(2,4-di-tert-butyl-phenyl)phosphite]gold(I) (4.4 mg, 5.0 μmol, 5 mol%) and silver bis(trifluoromethane-sulfonyl)imide (2.0 mg, 5.2 μmol, 5 mol%) in DCE (anhy., 0.2 mL) with general procedure **2.3** at 80 °C for 2 h. Column chromatography twice (silica gel, 10% → 20% EtOAc/pentane) afforded the title compound (34 mg, 47 μmol, 47%) as a yellow oil.

R_f 0.22 (20% EtOAc/pentane);

IR (thin film, ν_{max} / cm^{-1}) 3273, 2980, 2881, 2230, 1651, 1342, 1160, 1090, 910, 733;

^{1}H NMR (500 MHz, $CDCl_3$) δ_H 9.00 (1H, s, H8), 7.72 (2H, d, *J* = 8.2 Hz, H13), 7.53 (2H, d, *J* = 8.4 Hz, H14), 7.43 (2H, d, *J* = 8.1 Hz, H3), 7.37 (2H, d, *J* = 8.4 Hz, H15), 7.32 (2H, d, *J* = 8.2 Hz, H12), 7.17 – 7.10 (3H, m, H10, H11), 7.06 – 7.03 (2H, m, H9), 6.85 (2H, d, *J* = 8.1 Hz, H2), 4.04 – 3.86 (2H, m, H17), 3.37 (1H, td, *J* = 12.3, 5.3 Hz, H4), 3.28 (1H, td, *J* = 12.3, 5.3 Hz, H4), 2.42 (3H, s, H16), 2.15 (3H, s, H1), 1.77 (2H, quin, *J* = 7.8 Hz, H18), 1.46 – 1.35 (2H, m, H19), 1.30 – 1.20 (1H, m, H5), 1.17 – 1.07 (1H, m, H5), 1.01 – 0.92 (5H, m, H6, H20), 0.73 (3H, t, *J* = 7.3 Hz, H7);

^{13}C NMR (126 MHz, $CDCl_3$) δ_C 189.8, 144.5, 143.3, 141.8, 136.6, 136.4, 132.5, 131.5, 130.6, 130.3, 130.0, 129.3, 128.9, 128.8, 128.6, 128.1, 127.7, 126.8, 118.8, 118.3, 118.1, 115.1, 52.8, 51.6, 32.1, 30.7, 21.7, 21.5, 20.1, 19.9, 14.0, 13.8;

HRMS (ESI) m/z: $[M + Na]^+$ Calcd for $C_{40}H_{42}N_4O_5NaS_2$ 745.2489; Found 745.2482;

***N,N'*-(4-(4-Methoxybenzoyl)-5-phenyl-1*H*-pyrrole-2,3-diyl)bis(*N*-butyl-4-methylbenzenesulfonamide), 2m**

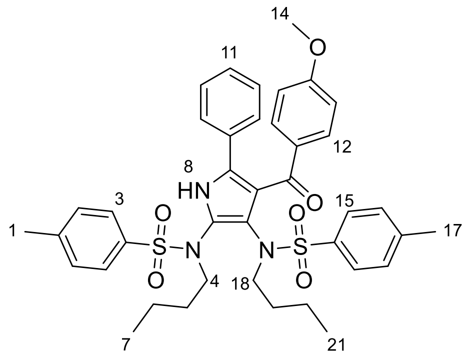

Synthesized from *N,N'*-(ethyne-1,2-diyl)bis(*N*-butyl-4-methylbenzenesulfonamide) (48 mg, 0.10 mmol, 1.0 eq.), 5-(4-methoxyphenyl)-3-phenylisoxazole (51 mg, 0.20 mmol, 2.0 eq.), chloro[tris(2,4-di-tert-butyl-phenyl)phosphite]gold(I) (4.4 mg, 5.0 μmol, 5 mol%) and silver bis(trifluoromethane-sulfonyl)imide (2.0 mg, 5.2 μmol, 5 mol%) in DCE (anhy., 0.2 mL) with general procedure **2.3** at 80 °C for 2 h. Upon completion, the reaction was treated with HCl (1.0 M in Et_2O, 0.1 mL) for 30 min at room temperature, after which

column chromatography (silica gel, 10% → 20% EtOAc/pentane) afforded the title compound (28 mg, 38 μmol, 38%) as a pink solid;

m.p. 156 °C (decomp.);

R$_f$ 0.26 (20% EtOAc/pentane);

IR (thin film, ν_{max} / cm^{-1}) 3293, 2961, 1644, 1599, 1338, 1257, 1158, 910, 732;

^{1}H NMR (400 MHz, $CDCl_3$) δ_H 8.96 (1H, s, H8), 7.73 (2H, d, *J* = 8.2 Hz, H15), 7.48 (2H, d, *J* = 8.9 Hz, H12), 7.37 (2H, d, *J* = 8.2 Hz, H3), 7.31 (2H, d, *J* = 8.2 Hz, H16), 7.16 – 7.10 (5H, m, H9, H10, H11), 6.80 (2H, d, *J* = 8.2 Hz, H2), 6.60 (2H, d, *J* = 8.9 Hz, H13), 4.12 – 4.02 (1H, m, H18), 4.00 – 3.88 (1H, m, H18) 3.75 (3H, s, H14), 3.41 – 3.30 (1H, m, H4), 3.25 – 3.14 (1H, m, H4), 2.41 (3H, s, H17), 2.15 (3H, s, H1), 1.81 (2H, quin, *J* = 7.8 Hz, H19), 1.42 (2H, sext, *J* = 7.3 Hz, H20), 1.29 – 1.19 (1H, m, H5), 1.15 – 1.04 (1H, m, H5), 0.98 (3H, t, *J* = 7.4 Hz, H21), 0.95 – 0.87 (2H, m, H6), 0.71 (3H, t, *J* = 7.3 Hz, H7);

^{13}C NMR (101 MHz, $CDCl_3$) δ_C 190.5, 163.0, 144.3, 143.0, 136.8, 136.4, 132.5, 131.3, 131.2, 130.4, 129.9, 129.2, 128.6, 128.09, 128.06 (2C), 127.7, 126.2, 119.1, 118.9, 113.0, 55.5, 52.9, 51.6, 32.1, 30.6, 21.7, 21.5, 20.0, 19.9, 14.1, 13.7;

HRMS (ESI) m/z: [M + H]$^+$ Calcd for $C_{40}H_{46}N_3O_6S_2$ 728.2823; Found 728.2819;

***N,N'*-(4-Benzoyl-5-methyl-1*H*-pyrrole-2,3-diyl)bis(*N*-butyl-4-methylbenzenesulfonamide), 2n**

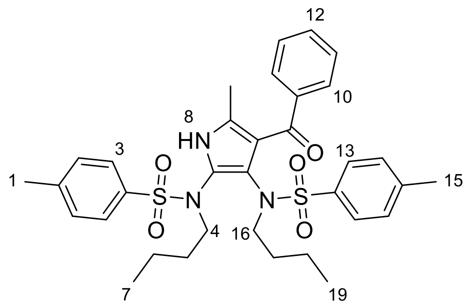

Synthesized from *N,N'*-(ethyne-1,2-diyl)bis(*N*-butyl-4-methylbenzenesulfonamide) (48 mg, 0.10 mmol, 1.0 eq.), 3-methyl-5-phenylisoxazole (32 mg, 0.20 mmol, 2.0 eq.), chloro[tris(2,4-di-tert-butyl-phenyl)phosphite]gold(I) (4.4 mg, 5.0 μmol, 5 mol%) and silver bis(trifluoromethane-sulfonyl)imide (2.0 mg, 5.2 μmol, 5 mol%) in DCE (anhy., 0.2 mL) with general procedure **2.3** at room temperature for 2 h. Column chromatography (silica gel, 10% → 15% → 20% EtOAc/pentane) afforded the title compound (37 mg, 58 μmol, 58%) as a colorless oil.

R$_f$ 0.26 (20% EtOAc/pentane);

IR (thin film, ν_{max} / cm^{-1}) 3325, 2960, 1643, 1450, 1339, 1158, 1090, 909, 731;

^{1}H NMR (400 MHz, $CDCl_3$) δ_H 8.80 (1H, s, H8), 7.74 (2H, d, *J* = 8.2 Hz, H13), 7.61 (2H, d, *J* = 7.0 Hz, H10), 7.52 (1H, t, *J* = 7.4 Hz, H12), 7.46 (2H, d, *J* = 8.2 Hz, H3), 7.43 – 7.37 (2H, m, H11), 7.31 (2H, d, *J* = 8.2 Hz, H14), 6.82 (2H, d, *J* = 8.2 Hz, H2), 4.03 – 3.91 (1H, m, H16), 3.88 – 3.77 (1H, m, H16), 3.33 – 3.18 (2H, m, H4), 2.41 (3H, s, H15), 2.12 (3H, s, H1), 1.91 (3H, s, H9), 1.75 – 1.67 (2H, m, H17), 1.40 – 1.30 (2H, m, H18), 1.25 – 1.15 (1H, m, H5), 1.10 – 1.00 (1H, m, H5), 0.97 – 0.88 (5H, m, H6, H19), 0.70 (3H, t, *J* = 7.3 Hz, H7);

^{13}C NMR (101 MHz, $CDCl_3$) δ_C 191.5, 144.2, 143.0, 139.8, 136.8, 136.4, 132.3, 129.9, 129.7, 129.3, 128.7, 128.3, 128.1, 127.7, 124.6, 119.1, 118.0, 52.8, 51.8, 32.0, 30.6, 21.7, 21.4, 20.1, 19.9, 14.5, 14.0, 13.7;
HRMS (ESI) m/z: $[M + H]^+$ Calcd for $C_{34}H_{42}N_3O_5S_2$ 636.2560; Found 636.2560;

***N*,*N'*-(4-Benzoyl-5-(tert-butyl)-1*H*-pyrrole-2,3-diyl)bis(*N*-butyl-4-methylbenzenesulfonamide), 2o**

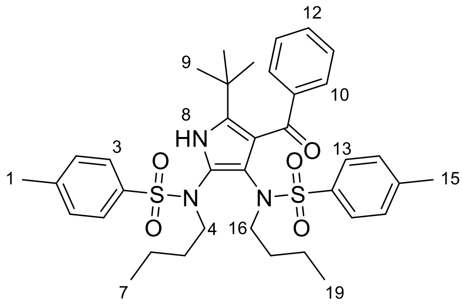

Synthesized from *N*,*N'*-(ethyne-1,2-diyl)bis(*N*-butyl-4-methylbenzenesulfonamide) (48 mg, 0.10 mmol, 1.0 eq.), 3-(tert-butyl)-5-phenylisoxazole (41 mg, 0.20 mmol, 2.0 eq.), chloro[tris(2,4-di-tert-butyl-phenyl)phosphite]gold(I) (4.4 mg, 5.0 μmol, 5 mol%) and silver bis(trifluoromethane-sulfonyl)imide (2.0 mg, 5.2 μmol, 5 mol%) in DCE (anhy., 0.2 mL) with general procedure **2.3** at room temperature for 4 h. Column chromatography (silica gel, 5% → 10% EtOAc/pentane) afforded the title compound (45 mg, 66 μmol, 66%) as a white solid;
m.p. 64 °C ($CHCl_3$);
R$_f$ 0.27 (10% EtOAc/pentane);
IR (thin film, ν_{max} / cm^{-1}) 2960, 1660, 1332, 1159, 1090, 1040, 910, 815, 732, 665;
^{1}H NMR (500 MHz, $CDCl_3$) δ_H 8.42 (1H, s, H8), 7.75 (2H, d, *J* = 6.9 Hz, H10), 7.69 (2H, d, *J* = 8.1 Hz, H13), 7.53 (1H, t, *J* = 7.4 Hz, H12), 7.43 – 7.37 (2H, m, H11), 7.31 (2H, d, *J* = 8.1 Hz, H14), 7.21 (2H, d, *J* = 8.2 Hz, H3), 6.98 (2H, d, *J* = 8.2 Hz, H2), 3.91 – 3.82 (1H, m, H16), 3.80 – 3.69 (1H, m, H16), 3.00 – 2.89 (1H, m, H4), 2.63 – 2.53 (1H, m, H4), 2.42 (3H, s, H15), 2.31 (3H, s, H1), 1.68 – 1.58 (2H, m, H17), 1.32 (2H, sext, *J* = 7.4 Hz, H18), 1.21 (9H, s, H9), 0.93 (3H, t, *J* = 7.4 Hz, H19), 0.90 – 0.83 (2H, m, H5), 0.73 – 0.63 (2H, m, H6), 0.58 (3H, t, *J* = 7.1 Hz, H7);
^{13}C NMR (126 MHz, $CDCl_3$) δ_C 195.8, 144.3, 143.1, 139.9, 137.1, 137.0, 135.4, 133.1, 130.4, 129.9, 129.2, 128.2, 128.1, 127.8, 121.4, 118.5, 117.9, 51.8, 51.5, 33.1, 31.6, 30.6, 30.1, 21.7, 21.6, 19.9, 19.8, 14.0, 13.6;
HRMS (ESI) m/z: $[M + H]^+$ Calcd for $C_{37}H_{48}N_3O_5S_2$ 678.3030; Found 678.3024;

***N*,*N'*-(4-Benzoyl-5-(4-fluorophenyl)-1*H*-pyrrole-2,3-diyl)bis(*N*-butyl-4-methylbenzenesulfonamide), 2p**

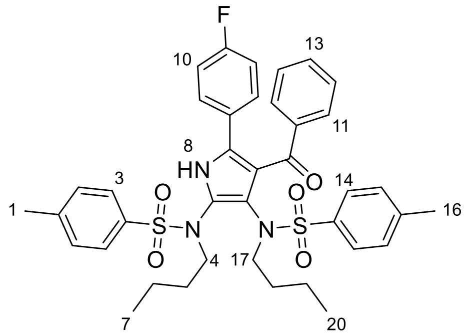

Synthesized from *N*,*N'*-(ethyne-1,2-diyl)bis(*N*-butyl-4-methylbenzenesulfonamide) (48 mg, 0.10 mmol, 1.0 eq.), 3-(4-fluorophenyl)-5-phenylisoxazole (49 mg, 0.20 mmol, 2.0 eq.), chloro[tris(2,4-di-tert-butyl-phenyl)phosphite]gold(I) (4.4 mg, 5.0 μmol, 5 mol%) and silver bis(trifluoromethane-sulfonyl)imide (2.0 mg, 5.2 μmol, 5 mol%) in DCE (anhy., 0.2 mL) with general procedure **2.3** at 80°C for 2 h. Column chromatography (silica gel, 10% → 20% EtOAc/pentane) afforded the title compound (48 mg, 67 μmol, 67%) as a white solid;

m.p. 187 °C (decomp.);

R_f 0.11 (10% EtOAc/pentane);

IR (thin film, ν_{max} / cm^{-1}) 3293, 2960, 1648, 1532, 1453, 1337, 1158, 1090, 732;

^{1}H NMR (400 MHz, $CDCl_3$) δ_H 9.09 (1H, s, H8), 7.73 (2H, d, J = 8.3 Hz, H14), 7.43 (2H, d, J = 7.1 Hz, H10), 7.39 (2H, d, J = 8.3 Hz, H3), 7.33 – 7.26 (3H, m, H13, H15), 7.14 – 7.02 (4H, m, H9, H11), 6.80 – 6.73 (4H, m, H2, H12), 4.12 – 4.00 (1H, m, H17), 3.95 – 3.84 (1H, m, H17), 3.37 (1H, td, J = 12.4, 5.2 Hz, H4), 3.26 (1H, td, J = 12.4, 5.2 Hz, H4), 2.42 (3H, s, H16), 2.10 (3H, s, H1), 1.81 (2H, quin, J = 7.7 Hz, H18), 1.41 (2H, sext, J = 7.2 Hz, H19), 1.33 – 1.18 (1H, m, H5), 1.19 – 1.03 (1H, m, H5), 1.02 – 0.90 (5H, m, H6, H20), 0.72 (3H, t, J = 7.3 Hz, H7);

^{13}C NMR (101 MHz, $CDCl_3$) δ_C 191.5, 162.5 (d, $^1J_{C-F}$ = 249.2 Hz), 144.4, 143.1, 138.1, 136.7, 136.2, 132.3, 130.6, 130.3, 130.2, 130.1, 129.9, 129.2, 128.0, 127.7 (2C), 127.4 (d, $^4J_{C-F}$ = 3.2 Hz), 126.5, 118.9 (d, $^2J_{C-F}$ = 22.2 Hz), 115.6 (d, $^3J_{C-F}$ = 21.9 Hz), 53.0, 51.7, 32.1, 30.6, 21.7, 21.4, 20.1, 19.9, 14.0, 13.7;

^{19}F NMR (377 MHz, $CDCl_3$) δ_F -112.68;

HRMS (ESI) m/z: $[M + H]^+$ Calcd for $C_{39}H_{43}FN_3O_5S_2$ 716.2623; Found 716.2618;

***N*,*N'*-(4-Benzoyl-5-(4-cyanophenyl)-1*H*-pyrrole-2,3-diyl)bis(*N*-butyl-4-methylbenzenesulfonamide), 2q**

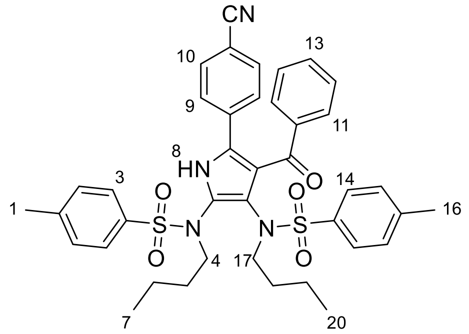

Synthesized from *N*,*N'*-(ethyne-1,2-diyl)bis(*N*-butyl-4-methylbenzenesulfonamide) (48 mg, 0.10 mmol, 1.0 eq.), 4-(5-phenylisoxazol-3-yl)benzonitrile (50 mg, 0.20 mmol, 2.0 eq.), chloro[tris(2,4-di-tert-butyl-phenyl)phosphite]gold(I) (4.4 mg, 5.0 μmol, 5 mol%) and silver bis(trifluoromethane-sulfonyl)imide (2.0 mg, 5.2 μmol, 5 mol%) in DCE (anhy., 0.2 mL) with general procedure **2.3** at 80 °C for 2 h. Column chromatography twice (silica gel, 10% → 20% EtOAc/pentane) afforded the title compound (26 mg, 36 μmol, 36%) as a yellow oil.

R_f 0.29 (20% EtOAc/pentane);

IR (thin film, ν_{max} / cm^{-1}) 3281, 2961, 2873, 2228, 1650, 1452, 1338, 1159, 1090, 911, 732;

^{1}H NMR (500 MHz, $CDCl_3$) δ_H 9.34 (1H, s, H8), 7.73 (2H, d, *J* = 8.3 Hz, H14), 7.46 (2H, d, *J* = 7.2 Hz, H11), 7.39 – 7.30 (7H, m, H3, H10, H13, H15), 7.19 (2H, d, *J* = 8.5 Hz, H9), 7.16 – 7.11 (2H, m, H12), 6.79 (2H, d, *J* = 8.0 Hz, H2), 4.09 – 3.99 (1H, m, H17), 3.96 – 3.86 (1H, m, H17), 3.35 (1H, td, *J* = 12.4, 5.1 Hz, H4), 3.21 (1H, td, *J* = 12.4, 5.1 Hz, H4), 2.43 (3H, s, H16), 2.13 (3H, s, H1), 1.81 (2H, quin, *J* = 7.7 Hz, H18), 1.47 – 1.34 (2H, m, H19), 1.26 – 1.17 (1H, m, H5), 1.14 – 1.05 (1H, m, H5), 1.01 – 0.89 (5H, m, H6, H20), 0.71 (3H, t, *J* = 7.3 Hz, H7);

^{13}C NMR (126 MHz, $CDCl_3$) δ_C 191.3, 144.6, 143.3, 137.8, 136.4, 136.1, 135.3, 132.9, 132.2, 130.1, 130.0, 129.3, 128.5, 128.5, 128.03, 127.98, 127.71, 127.69, 120.6, 119.4, 118.4, 111.4, 52.8, 51.5, 32.1, 30.6, 21.7, 21.5, 20.0, 19.9, 14.0, 13.7;

HRMS (ESI) m/z: $[M + H]^+$ Calcd for $C_{40}H_{43}N_4O_5S_2$ 723.2669; Found 723.2654;

***N*,*N'*-(4-Benzoyl-5-(4-methoxyphenyl)-1*H*-pyrrole-2,3-diyl)bis(*N*-butyl-4-methylbenzenesulfonamide), 2r**

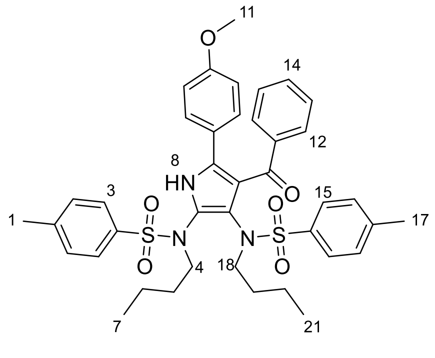

Synthesized from *N*,*N'*-(ethyne-1,2-diyl)bis(*N*-butyl-4-methylbenzenesulfonamide) (48 mg, 0.10 mmol, 1.0 eq.), 3-(4-methoxyphenyl)-5-phenylisoxazole (51 mg, 0.20 mmol, 2.0 eq.), chloro[tris(2,4-di-tert-butyl-phenyl)phosphite]gold(I) (4.4 mg, 5.0 μmol, 5 mol%) and silver bis(trifluoromethane-sulfonyl)imide (2.0 mg, 5.2 μmol, 5 mol%) in DCE (anhy., 0.2 mL) with general procedure **2.3** at 80°C for 2 h. Column chromatography (silica gel, 20% EtOAc/pentane) afforded the title compound (48 mg, 66 μmol, 65%) as a pale yellow solid;

m.p. 180 °C (decomp.);

R$_f$ 0.24 (20% EtOAc/pentane);

IR (thin film, ν_{max} / cm^{-1}) 3303, 2962, 1646, 1578, 1495, 1338, 1251, 1159, 1090, 732;

^{1}H NMR (400 MHz, $CDCl_3$) δ_H 8.93 (1H, s, H8), 7.74 (2H, d, *J* = 8.3 Hz, H15), 7.45 (2H, d, *J* = 7.1 Hz, H12), 7.39 (2H, d, *J* = 8.2 Hz, H3), 7.34 – 7.26 (3H, m, H14, H16), 7.13 – 7.07 (2H, m, H13), 7.00 (2H, d, *J* = 8.8 Hz, H9), 6.75 (2H, d, *J* = 8.2 Hz, H2), 6.62 (2H, d, *J* = 8.8 Hz, H10), 4.12 – 4.01 (1H, m, H18), 3.97 – 3.87 (1H, m, H18), 3.69 (3H, s, H11), 3.42 – 3.33 (1H, m, H4), 3.32 – 3.22 (1H, m, H4), 2.42 (3H, s, H17), 2.10 (3H, s, H1), 1.81 (2H, quin, *J* = 7.7 Hz, H19), 1.41 (2H, sext, *J* = 7.3 Hz, H20), 1.33 – 1.19 (1H, m, H5), 1.17 – 1.03 (1H, m, H5), 1.02 – 0.90 (5H, m, H6, H21), 0.72 (3H, t, *J* = 7.3 Hz, H7);

^{13}C NMR (101 MHz, $CDCl_3$) δ_C 191.6, 159.5, 144.2, 143.0, 138.2, 136.7, 136.3, 132.1, 131.8, 130.2, 129.9, 129.7, 129.2, 128.0, 127.7, 127.6, 126.0, 123.8, 119.0, 118.0, 114.0, 55.4, 53.1, 51.7, 32.1, 30.6, 21.7, 21.4, 20.1, 19.9, 14.0, 13.7;

HRMS (ESI) m/z: $[M + H]^+$ Calcd for $C_{40}H_{46}N_3O_6S_2$ 728.2823; Found 728.2820;

***N,N'*-(4-Benzoyl-5-(furan-2-yl)-1*H*-pyrrole-2,3-diyl)bis(*N*-butyl-4-methylbenzenesulfonamide), 2s**

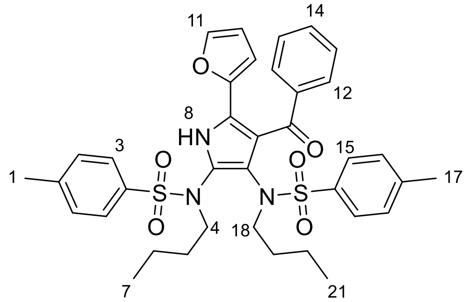

Synthesized from *N,N'*-(ethyne-1,2-diyl)bis(*N*-butyl-4-methylbenzenesulfonamide) (48 mg, 0.10 mmol, 1.0 eq.), 3-(furan-2-yl)-5-phenylisoxazole (43 mg, 0.20 mmol, 2.0 eq.), chloro[tris(2,4-di-tert-butyl-phenyl)phosphite]gold(I) (4.4 mg, 5.0 μmol, 5 mol%) and silver bis(trifluoromethane-sulfonyl)imide (2.0 mg, 5.2 μmol, 5 mol%) in DCE (anhy., 0.2 mL) with general procedure **2.3** at room temperature for 2 h. Column chromatography (silica gel, 5% → 10% → 20% EtOAc/pentane) afforded the title compound (8 mg, 12 μmol, 12%) as a yellow oil;

R$_f$ 0.33 (20% EtOAc/pentane);

IR (thin film, ν_{max} / cm^{-1}) 3297, 2960, 2873, 1651, 1339, 1162, 1090, 731, 665;

^{1}H NMR (400 MHz, $CDCl_3$) δ_H 9.18 (1H, s, H8), 7.74 (2H, d, *J* = 8.3 Hz, H15), 7.67 (2H, d, *J* = 7.1 Hz, H12), 7.47 (1H, t, *J* = 7.4 Hz, H14), 7.38 (2H, d, *J* = 8.2 Hz, H3), 7.34 – 7.29 (4H, m, H13, H16), 7.23 (1H, dd, *J* = 1.8, 0.6 Hz, H11), 6.83 (2H, d, *J* = 8.2 Hz, H2), 6.15 (1H, dd, *J* = 3.5, 1.8 Hz, H10), 5.67 (1H, dd, *J* = 3.5, 0.6 Hz, H9), 3.98 – 3.87 (2H, m, H18), 3.35 – 3.24 (1H, m, H4), 3.21 – 3.09 (1H, m, H4), 2.42 (3H, s, H17), 2.17 (3H, s, H1), 1.74 (2H, quin, *J* = 7.9 Hz, H19), 1.44 – 1.32 (2H, m, H20), 1.23 – 1.01 (2H, m, H5), 0.96 (3H, t, *J* = 7.4 Hz, H21), 0.93 – 0.85 (2H, m, H6), 0.70 (3H, t, *J* = 7.3 Hz, H7);

^{13}C NMR (101 MHz, $CDCl_3$) δ_C 191.3, 144.7, 144.4, 143.2, 141.9, 138.4, 136.7, 136.4, 132.8, 130.04, 130.00, 129.3, 128.2, 128.0, 127.7, 125.8, 121.1, 118.7, 118.0, 111.8, 109.3, 52.6, 51.6, 32.0, 30.5, 21.7, 21.5, 20.0, 19.9, 14.0, 13.7;

HRMS (ESI) m/z: $[M + H]^+$ Calcd for $C_{37}H_{42}N_3O_6S_2$ 688.2510; Found 688.2506;

_N_-(4-Benzoyl-2-((_N_-cyclohexyl-4-methylphenyl)sulfonamido)-5-methyl-1_H_-pyrrol-3-yl)-_N_-butyl-4-methylbenzenesulfonamide, 2dc

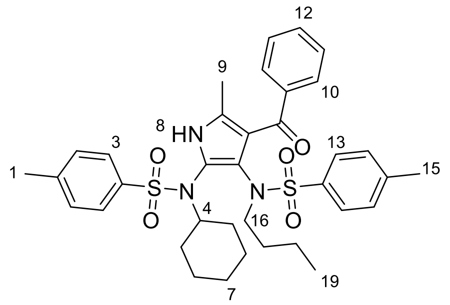

Synthesized from *N*-butyl-*N*-(((*N*-cyclohexyl-4-methylphenyl)sulfonamido)ethynyl)-4-methylbenzenesulfonamide (50 mg, 0.10 mmol, 1.0 eq.), 3-methyl-5-phenylisoxazole (32 mg, 0.20 mmol, 2.0 eq.), chloro[tris(2,4-di-tert-butyl-phenyl)phosphite]gold(I) (4.4 mg, 5.0 µmol, 5 mol%) and silver bis(trifluoromethane-sulfonyl)imide (2.0 mg, 5.2 µmol, 5 mol%) in DCE (anhy., 0.2 mL) with general procedure **2.3** at room temperature for 2 h. Upon completion, HCl (1 M in Et_2O, 0.1 mL) was added to the reaction. The reaction mixture was stirred for 30 min before it was concentrated *in vacuo* and purified by column chromatography (silica gel, 20% → 40% Et_2O/pentane) afforded the title compound (61 mg, 92 µmol, 92%) as a yellow solid;

m.p. 114 – 115 °C;

R_f 0.13 (40% Et_2O/pentane);

IR (thin film, ν_{max} / cm^{-1}) 3329, 2933, 1643, 1450, 1335, 1160, 1090, 909, 731;

^{1}H NMR (500 MHz, C_6D_6) δ_H 8.84 (1H, s, H8), 8.04 (2H, d, *J* = 8.3 Hz, H3), 7.83 (2H, d, *J* = 8.2 Hz, H13), 7.65 – 7.61 (2H, m, H10), 7.10 – 7.03 (1H, m, H12), 6.99 – 6.94 (2H, m, H11), 6.82 (2H, d, *J* = 8.3 Hz, H2), 6.54 (2H, d, *J* = 8.2 Hz, H14), 4.48 (1H, tt, *J* = 12.0, 3.7 Hz, H4), 3.77 – 3.61 (2H, m, H16), 2.72 – 2.36 (4H, m, H5), 1.90 – 1.79 (5H, m, H1, H6), 1.72 (3H, s, H15), 1.66 – 1.53 (3H, m, H6, H7), 1.51 – 1.45 (4H, m, H7, H9), 1.37 – 1.19 (2H, m, H17), 1.10 – 0.94 (2H, m, H18), 0.74 (3H, t, *J* = 7.4 Hz, H19);

^{13}C NMR (126 MHz, C_6D_6) δ_C 191.1, 143.7, 142.6, 140.7, 139.0, 137.7, 131.8, 129.9, 129.3, 128.8, 128.6, 128.5, 128.4, 126.0, 120.6, 119.9, 67.2, 53.0, 33.4, 32.2, 30.8, 26.9, 25.5, 21.1, 21.0, 20.6, 14.0, 13.9;

HRMS (ESI) m/z: $[M + H]^+$ Calcd for $C_{36}H_{44}N_3O_5S_2$ 662.2717; Found 662.2716;

***N*-(4-Benzoyl-2-((*N*-cyclohexyl-4-methylphenyl)sulfonamido)-5-isopropyl-1*H*-pyrrol-3-yl)-*N*-butyl-4-methylbenzenesulfonamide, 2dd**

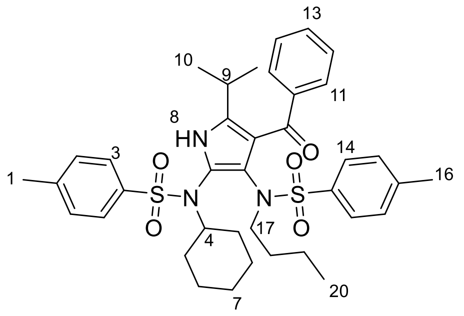

Synthesized from *N*-butyl-*N*-(((*N*-cyclohexyl-4-methylphenyl)sulfonamido)ethynyl)-4-methylbenzenesulfonamide (50 mg, 0.10 mmol, 1.0 eq.), 3-isopropyl-5-phenylisoxazole (38 mg, 0.20 mmol, 2.0 eq.), chloro[tris(2,4-di-tert-butyl-phenyl)phosphite]gold(I) (4.4 mg, 5.0 µmol, 5 mol%) and silver bis(trifluoromethane-sulfonyl)imide (2.0 mg, 5.2 µmol, 5 mol%) in DCE (anhy., 0.2 mL) with general procedure **2.3** at room temperature for 2 h. Upon completion, HCl (1 M in Et_2O, 0.1 mL) was added to the reaction. The reaction mixture was stirred for 12 h before it was concentrated *in vacuo* and purified by column chromatography (silica gel, 10% EtOAc/pentane) afforded the title compound (53 mg, 77 µmol, 77%) as a pink solid;

m.p. 183 °C (decomp.);

R_f 0.14 (10% EtOAc/pentane);

IR (thin film, ν_{max} / cm^{-1}) 2933, 2860, 1646, 1452, 1349, 1161, 909, 731;

^{1}H NMR (500 MHz, $CDCl_3$) δ_C 8.19 (1H, s, H8), 7.70 (2H, d, *J* = 7.1 Hz, H11), 7.66 (2H, d, *J* = 8.2 Hz, H3), 7.54 (1H, t, *J* = 7.4 Hz, H13), 7.47 – 7.38 (4H, m, H12, H14), 7.29 (2H, d, *J* = 8.2 Hz, H2), 6.83 (2H, d, *J* = 8.1 Hz, H15), 4.01 (1H, tt, *J* = 11.6, 4.2 Hz, H4), 3.35 – 3.23 (2H, m, H17), 2.63 (1H, hept, *J* = 6.9 Hz, H9), 2.42 (3H, s, H1), 2.20 – 2.03 (5H, m, H5, H16), 1.92 – 1.78 (4H, m, H5, H6), 1.60 – 1.54 (1H, m, H7), 1.46 – 1.15 (5H, m, H6, H7, H18), 1.11 (3H, d, *J* = 6.9 Hz, H10), 1.02 – 0.94 (5H, m, H10, H19), 0.77 (3H, t, *J* = 7.3 Hz, H20);

^{13}C NMR (126 MHz, $CDCl_3$) δ_C 191.9, 144.2, 142.9, 140.0, 137.7, 137.1, 136.9, 132.5, 129.9, 129.8, 129.1, 128.2, 128.1, 127.7, 123.8, 120.2, 117.9, 65.8, 52.9, 33.1, 31.7, 31.1, 26.5 (2C), 26.1, 25.1, 23.9, 21.7, 21.5, 21.3, 20.2, 13.8; *Extra peaks are observed due to diastereotopic isopropyl and cyclohexyl groups.*

HRMS (ESI) m/z: $[M + Na]^+$ Calcd for $C_{38}H_{47}N_3O_5NaS_2$ 712.2849; Found 712.2844.

***N,N'*-(Benzo[f][1,4]oxazepine-2,3-diyl)bis(*N*-butyl-4-methylbenzenesulfonamide), 2ya**

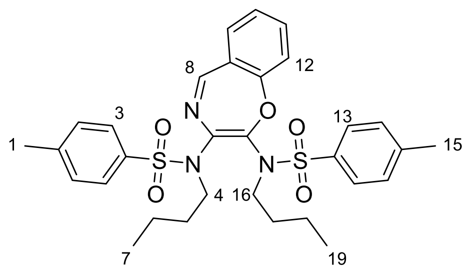

Synthesized from *N,N'*-(ethyne-1,2-diyl)bis(*N*-butyl-4-methylbenzenesulfonamide) (48 mg, 0.10 mmol, 1.0 eq.), benzo[*d*]isoxazole (20.5 μL, 0.20 mmol, 2.0 eq.), chloro[tris(2,4-di-tert-butyl-phenyl)phosphite]gold(I) (4.4 mg, 5.0 μmol, 5 mol%) and silver bis(trifluoromethane-sulfonyl)imide (2.0 mg, 5.2 μmol, 5 mol%) in DCE (anhy., 0.2 mL) with general procedure **2.3** at room temperature for 2 h. Column chromatography (silica gel, 10% → 20% EtOAc/pentane) afforded the title compound (59 mg, 99 μmol, 98%) as a yellow solid;

m.p. 110 – 112 °C;

R$_f$ 0.31 (20% EtOAc/pentane);

IR (thin film, ν_{max} / cm^{-1}) 2962, 2874, 1600, 1346, 1162, 1087, 1040, 814, 685, 658;

^{1}H NMR (400 MHz, C_6D_6) δ_H 8.30 (1H, s, H8), 8.15 (2H, d, *J* = 8.3 Hz, H13), 8.11 (2H, d, *J* = 8.3 Hz, H3), 6.93 – 6.82 (5H, m, H2, H9, H14), 6.83 – 6.76 (1H, m, H11), 6.69 (1H, t, *J* = 7.4 Hz, H10), 6.39 (1H, d, *J* = 8.1 Hz, H12), 4.25 – 3.85 (4H, m, H4, H16), 1.99 – 1.87 (8H, m, H1, H15, H17), 1.64 – 1.48 (2H, brs, H5), 1.32 – 1.22 (2H, m, H18), 1.15 – 1.05 (2H, m, H6), 0.79 (3H, t, *J* = 7.4 Hz, H19), 0.65 (3H, t, *J* = 7.3 Hz, H7);

^{13}C NMR (101 MHz, C_6D_6) δ_C 159.9, 159.1, 143.7, 143.2, 138.9, 138.4, 138.3, 133.6, 133.3, 129.6, 129.5, 129.4, 129.3, 129.2, 129.0, 125.5, 120.9, 51.0, 50.1, 31.1, 30.1, 21.2 (2C), 20.5, 20.2, 13.9, 13.8;

HRMS (ESI) m/z: $[M + H]^+$ Calcd for $C_{31}H_{38}N_3O_5S_2$ 596.2247; Found 596.2239;

***N,N'*-(Naphtho[1,2-*f*][1,4]oxazepine-3,4-diyl)bis(*N*-butyl-4-methylbenzenesulfonamide), *unstable*, 2yb**

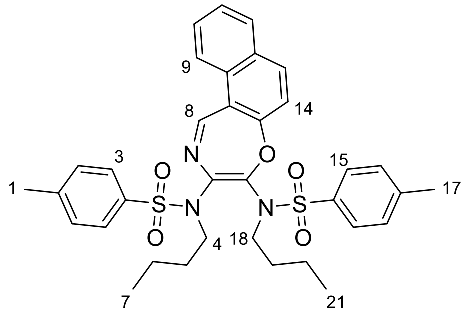

Synthesized from *N,N'*-(ethyne-1,2-diyl)bis(*N*-butyl-4-methylbenzenesulfonamide) (48 mg, 0.10 mmol, 1.0 eq.), naphtho[1,2-*d*]isoxazole (34 mg, 0.20 mmol, 2.0 eq.), chloro[tris(2,4-di-tert-butylphenyl)phosphite]gold(I) (4.4 mg, 5.00 µmol, 5 mol%) and silver bis(trifluoromethanesulfonyl)imide (2.0 mg, 5.15 µmol**,** 5 mol%) in DCE (anhy., 0.2 mL) with general procedure **2.3** at room temperature for 2 h. Upon completion, the reaction mixture was treated with HCl (1.0 M in Et_2O, 0.1 mL) for 30 min before it was concentrated *in vacuo* and purified by column chromatography (silica gel, 10% → 20% EtOAc/pentane) to afford the title compound (63 mg, 0.098 mmol, 97%) as a yellow oil.

R$_f$ 0.34 (20% EtOAc/pentane);

IR (thin film, ν_{max} / cm^{-1}) 2960, 2873, 1624, 1599, 1346, 1197, 1162, 1088, 1039, 816, 736, 663;

^{1}H NMR (400 MHz, $CDCl_3$) δ_H 9.14 (1H, s, H8), 8.09 (1H, d, *J* = 8.4 Hz, H9), 8.05 (2H, d, *J* = 8.3 Hz, H15), 7.87 – 7.83 (2H, m, H12, H13), 7.80 (2H, d, *J* = 8.3 Hz, H3), 7.67 (1H, t, *J* = 7.0 Hz, H10), 7.54 (1H, t, *J* = 7.5 Hz, H11), 7.41 (2H, d, *J* = 8.3 Hz, H16), 7.32 (2H, d, *J* = 8.3 Hz, H2), 6.46 (1H, d, *J* = 8.8 Hz, H14), 3.72 – 3.56 (4H, m, H4, H18), 2.51 (3H, s, H17), 2.46 (3H, s, H1), 1.79 – 1.63 (2H, m, H19), 1.36 – 1.20 (4H, m, H5, H20), 1.05 (2H, sext, *J* = 7.3 Hz, H6), 0.86 (3H, t, *J* = 7.4 Hz, H21), 0.64 (3H, t, *J* = 7.3 Hz, H20);

^{13}C NMR (101 MHz, $CDCl_3$) δ_C 159.5, 158.2, 144.2, 143.6, 137.7, 137.7, 137.0, 134.8, 133.4, 131.2, 131.1, 129.6, 129.4, 129.0, 128.68, 128.65, 128.63, 126.3, 122.8, 120.9, 120.0, 50.9, 49.8, 30.4, 29.7, 21.8, 21.7, 20.3, 19.9, 13.8, 13.6;

HRMS (ESI) m/z: $[M + H]^+$ Calcd for $C_{35}H_{40}N_3O_5S_2$ 646.2404; Found 646.2399;

_N,N'_-(8-(Diethylamino)benzo[_f_][1,4]oxazepine-2,3-diyl)bis(_N_-butyl-4-methylbenzenesulfonamide) 2yc

Synthesized from *N,N'*-(ethyne-1,2-diyl)bis(*N*-butyl-4-methylbenzenesulfonamide) (48 mg, 0.10 mmol, 1.0 eq.), *N,N*-diethylbenzo[*d*]isoxazol-6-amine (39 mg, 0.21 mmol, 2.0 eq.), chloro[tris(2,4-di-tert-butylphenyl)phosphite]gold(I) (4.4 mg, 5.00 μmol, 5 mol%) and silver bis(trifluoromethanesulfonyl)imide (2.0 mg, 5.15 μmol**,** 5 mol%) in DCE (anhy., 0.2 mL) with general procedure **2.3** at room temperature for 2 h. Column chromatography (silica gel, 10% → 20% EtOAc/pentane) afforded the title compound (53 mg, 0.079 mmol, 79%) as a yellow solid;

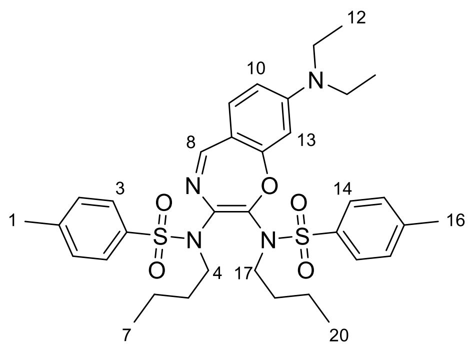

m.p. 159 °C (decomp.);

R_f 0.16 (20% EtOAc/pentane);

IR (thin film, ν_{max} / cm^{-1}) 2963, 1615, 1575, 1519, 1344, 1155, 1116, 1087, 910, 732, 665;

^{1}H NMR (400 MHz, $CDCl_3$) δ_H 8.20 (1H, s, H8), 8.04 (2H, d, *J* = 8.3 Hz, H14), 7.88 (2H, d, *J* = 8.3 Hz, H3), 7.37 (2H, d, *J* = 8.3 Hz, H15), 7.29 (2H, d, *J* = 8.3 Hz, H2), 7.18 (1H, d, *J* = 8.8 Hz, H9), 6.41 (1H, dd, *J* = 8.8, 2.4 Hz, H10), 5.25 (1H, d, *J* = 2.4 Hz, H13), 3.75 (2H, brs., H17), 3.61 (2H, brs., H4), 3.17 (4H, q, *J* = 7.0 Hz, H11), 2.45 (3H, s, H16), 2.42 (3H, s, H1), 1.64 (2H, brs., H18), 1.32 – 1.14 (4H, m, H5, H19), 1.11 – 0.97 (8H, m, H6, H12), 0.84 (3H, t, *J* = 7.3 Hz, H20), 0.62 (3H, t, *J* = 7.2 Hz, H7);

^{13}C NMR (101 MHz, $CDCl_3$) δ_C 160.1, 159.2, 152.5, 143.4, 143.2, 138.8, 137.7, 135.2, 132.6, 131.7, 129.3, 129.2, 128.8, 128.7, 115.8, 108.2, 101.7, 50.7, 49.6, 44.6, 30.9, 29.5, 21.70, 21.68, 20.2, 20.0, 13.8, 13.7, 12.5;

HRMS (ESI) m/z: $[M + H]^+$ Calcd for $C_{35}H_{47}N_4O_5S_2$ 667.2982; Found 667.2981;

***N,N'*-(9-(tert-Butyl)benzo[*f*][1,4]oxazepine-2,3-diyl)bis(*N*-butyl-4-methylbenzenesulfonamide), 2yd**

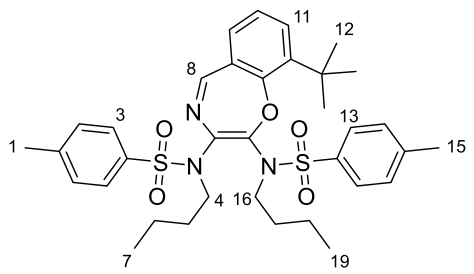

Synthesized from *N,N'*-(ethyne-1,2-diyl)bis(*N*-butyl-4-methylbenzenesulfonamide) (48 mg, 0.10 mmol, 1.0 eq.), 7-(*tert*-butyl)benzo[*d*]isoxazole (36 mg, 0.21 mmol, 2.0 eq.), chloro[tris(2,4-di-tert-butylphenyl)phosphite]gold(I) (4.4 mg, 5.00 µmol, 5 mol%) and silver bis(trifluoromethanesulfonyl)imide (2.0 mg, 5.15 µmol**,** 5 mol%) in DCE (anhy., 0.2 mL) with general procedure **2.3** at room temperature for 2 h. Column chromatography (silica gel, 10% EtOAc/pentane) afforded the title compound (60 mg, 0.092 mmol, 91%) as a yellow oil;

R_f 0.16 (10% EtOAc/pentane);

IR (thin film, ν_{max} / cm^{-1}) 2961, 2874, 1626, 1345, 1163, 1087, 909, 731, 658;

^{1}H NMR (400 MHz, $CDCl_3$) δ_H 8.22 (1H, s, H8), 7.92 (2H, d, *J* = 8.3 Hz, H13), 7.80 (2H, d, *J* = 8.3 Hz, H3), 7.46 (1H, dd, *J* = 7.2, 2.4 Hz, H9), 7.38 – 7.31 (4H, m, H2, H14), 7.22 – 7.12 (2H, m, H10, H11), 3.73 – 3.63 (1H, m, H16), 3.59 – 3.48 (1H, m, H16), 3.47 – 3.34 (2H, m, H4), 2.48 (3H, s, H15), 2.47 (3H, s, H1), 1.90 – 1.71 (3H, m, H5, H17), 1.52 – 1.39 (1H, m, H17), 1.34 – 1.25 (2H, m, H18), 1.23 – 1.11 (11H, m, H6, H12), 0.92 – 0.77 (6H, m, H7, H19);

^{13}C NMR (101 MHz, $CDCl_3$) δ_C 163.2, 163.1, 143.7, 143.6, 141.4, 139.6, 137.6, 137.5, 136.4, 131.7, 129.7, 129.3, 129.0, 128.6, 128.3, 128.1, 125.0, 52.5, 50.6, 35.1, 31.0, 30.2, 29.1, 21.71, 21.68, 20.4 (2C), 13.8 (2C);

HRMS (ESI) m/z: $[M + H]^+$ Calcd for $C_{35}H_{46}N_3O_5S_2$ 652.2873; Found 652.2874;

N,N'-(7-Formyl-1*H*-indole-2,3-diyl)bis(*N*-butyl-4-methylbenzenesulfonamide), 2z

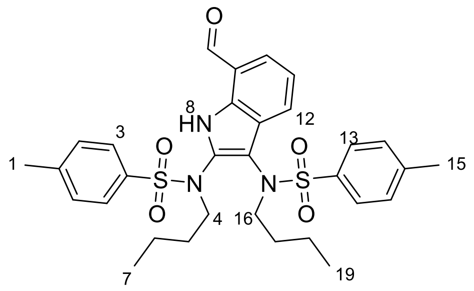

Synthesized from *N*-butyl-*N*-(((*N*-cyclohexyl-4-methylphenyl)sulfonamido)ethynyl)-4-methylbenzenesulfonamide (50 mg, 0.10 mmol, 1.0 eq.), anthranil (20.3 µL, 0.20 mmol, 2.0 eq.), chloro[tris(2,4-di-tert-butyl-phenyl)phosphite]gold(I) (4.4 mg, 5.0 µmol, 5 mol%) and silver bis(trifluoromethane-sulfonyl)imide (2.0 mg, 5.2 µmol, 5 mol%) in DCE (anhy., 0.2 mL) with general procedure **2.3** at room temperature for 2 h. Column chromatography (silica gel, 10% → 20% EtOAc/pentane) afforded the title compound (59 mg, 99 µmol, 98%) as a yellow solid;

m.p. 73 °C ($CHCl_3$);

R_f 0.28 (20% EtOAc/pentane);

IR (thin film, ν_{max} / cm^{-1}) 3420, 2962, 1673, 1344, 1163, 909, 730, 664;

^{1}H NMR (400 MHz, $CDCl_3$) δ_H 10.20 (1H, s, H8), 10.09 (1H, s, H9), 7.78 (2H, d, *J* = 8.2 Hz, H3), 7.66 – 7.57 (3H, m, H12, H13), 7.33 (2H, d, *J* = 8.2 Hz, H2), 7.24 (2H, d, *J* = 8.0 Hz, H14), 7.12 – 7.02 (2H, m, H10, H11), 3.90 – 3.66 (2H, m, H4), 3.61 – 3.48 (1H, m, H16), 3.45 – 3.31 (1H, m, H16), 2.46 – 2.40 (6H, m, H1, H15), 1.56 – 1.43 (2H, m, H5), 1.38 – 1.28 (2H, m, H17), 1.28 – 1.18 (2H, m, H6), 1.16 – 1.05 (2H, m, H18), 0.84 (3H, t, *J* = 7.4 Hz, H7), 0.76 (3H, t, *J* = 7.3 Hz, H19);

^{13}C NMR (101 MHz, $CDCl_3$) δ_C 193.3, 144.5, 143.8, 137.5, 137.0, 132.5, 130.1, 129.7, 129.5, 129.2, 128.0, 127.7, 126.6, 125.9, 120.3, 120.1, 111.4, 51.8, 51.1, 31.4, 30.8, 21.70, 21.66, 19.9, 19.8, 13.8, 13.7;

HRMS (ESI) m/z: $[M + H]^+$ Calcd for $C_{31}H_{38}N_3O_5S_2$ 596.2247; Found 596.2250;

4.4 Derivatizations of the 2,3-Diamino Pyrroles

***N*,*N'*-(5-Bromo-4-formyl-1*H*-pyrrole-2,3-diyl)bis(*N*-butyl-4-methylbenzenesulfonamide), 3ab**

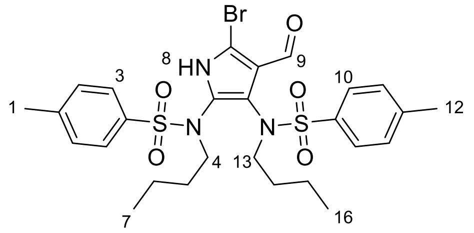

According to a literature procedure.[19] To a stirred solution of *N*,*N'*-(4-formyl-1*H*-pyrrole-2,3-diyl)bis(*N*-butyl-4-methylbenzenesulfonamide) (55 mg, 0.10 mmol, 1.0 eq.) in hexafluoroisopropanol (0.4 mL), *N*-bromosuccinimide (20 mg, 0.11 mmol, 1.1 eq.) was added portion-wise. The reaction mixture was stirred at room temperature until completion by TLC. Upon completion, the reaction mixture was concentrated *in vacuo* and purified by column chromatography (silica gel, 20% → 30% → 40% Et_2O/pentane) to afford the title compound (36 mg, 58 μmol, 58%) as a yellow solid;

m.p. 91 °C;

R_f 0.26 (20% EtOAc/pentane);

IR (thin film, ν_{max} / cm^{-1}) 3232, 2961, 1680, 1662, 1343, 1163, 1090, 910, 732;

^{1}H NMR (400 MHz, $CDCl_3$) δ_H 9.57 (1H, s, H8), 9.20 (1H, s, H9), 7.81 (2H, d, *J* = 8.2 Hz, H3), 7.53 (2H, d, *J* = 8.2 Hz, H10), 7.33 (2H, d, *J* = 8.2 Hz, H2), 7.24 (2H, d, *J* = 8.2 Hz, H11), 3.93 – 3.70 (2H, m, H4), 3.26 – 3.17 (2H, m, H13), 2.44 – 2.39 (6H, m, H1, H12), 1.65 (2H, quin, *J* = 7.6 Hz, H5), 1.36 – 1.26 (2H, m, H6), 1.19 – 1.09 (1H, m, H14), 1.07 – 0.94 (3H, m, H14, H15), 0.90 (3H, t, *J* = 7.4 Hz, H7), 0.71 (3H, t, *J* = 7.1 Hz, H16);

^{13}C NMR (101 MHz, $CDCl_3$) δ_C 183.3, 144.6, 143.9, 136.2, 135.8, 130.0, 129.5, 128.2, 127.9 (2C), 119.0, 118.0, 107.3, 51.6, 51.3, 31.8, 30.5, 21.7 (2C), 20.0, 19.8, 13.9, 13.7;

HRMS (ESI) m/z: $[M + H]^+$ Calcd for $C_{27}H_{35}N_3O_5{}^{79}BrS_2$ 624.1196; Found 624.1191;

***N*,*N'*-(4-cyano-1*H*-pyrrole-2,3-diyl)bis(*N*-butyl-4-methylbenzenesulfonamide), 3ac**

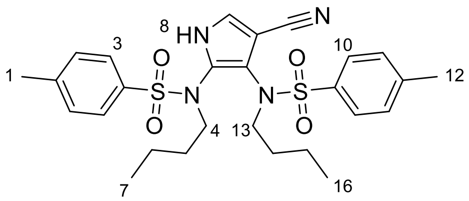

Step 1: To a vial equipped with a stirrer bar was added *N,N'*-(4formyl-1*H*-pyrrole-2,3-diyl)bis(*N*-butyl-4-methylbenzenesulfonamide) (55 mg, 0.10 mmol, 1.0 eq.) and hydroxylamine hydrochloride (21 mg, 0.30 mmol, 3.0 eq.). EtOH (0.4 mL) was added to the vial and the resulting solution was refluxed at 80 °C for 4 h. The reaction mixture was then diluted with water and extracted three times with DCM. The organic phase was collected, washed with brine, and dried over Na_2SO_4. The solvent was removed *in vacuo* to afford the *N,N'*-(4-((hydroxyimino)methyl)-1*H*-pyrrole-2,3-diyl)bis(*N*-butyl-4-methylbenzenesulfonamide) which was used in the next step without further purification.

Step 2: *According to a modified literature procedure.*[20] In a vial equipped with a stirrer bar, was dissolved cyanuric chloride (20 mg, 0.11 mmol, 1.1 eq.) in DMF (0.2 mL). The solution was stirred at room temperature until the disappearance of cyanuric chloride by TLC. A solution of the oxime prepared in the previous step in DMF (0.2 mL) was then added and the reaction mixture was stirred at room temperature until completion by TLC. Upon completion, the reaction mixture was diluted with water and extracted three times with DCM. The organic phase was combined, washes with brine, and dried over Na_2SO_4. Column chromatography (silica gel, 20% → 40% EtOAc/pentane) afforded the title compound (30 mg, 55 μmol, 55%) as a brown solid;

m.p. 74 °C;

R_f 0.63 (40% EtOAc/pentane);

IR (thin film, ν_{max} / cm^{-1}) 3304, 2960, 2231, 1597, 1345, 1164, 1089, 910, 731, 665;

^{1}H NMR (400 MHz, $CDCl_3$) δ_H 9.50 (1H, s, H8), 7.74 (2H, d, *J* = 8.3 Hz, H3), 7.64 (2H, d, *J* = 8.3 Hz, H10), 7.33 – 7.27 (4H, m, H2, H11), 7.09 (1H, d, *J* = 3.5 Hz, H9), 3.92 – 3.83 (2H, m, H4), 3.30 – 3.13 (1H, brs, H13), 3.07 – 2.86 (1H, brs, H13), 2.42 (3H, s, H1), 2.40 (3H, s, H12), 1.61 (2H, quin, *J* = 7.7 Hz, H5), 1.37 – 1.27 (2H, m, H6), 1.12 – 0.94 (4H, m, H14, H15), 0.89 (3H, t, *J* = 7.4 Hz, H7), 0.70 (3H, t, *J* = 7.2 Hz, H16);

^{13}C NMR (101 MHz, $CDCl_3$) δ_C 144.6, 144.4, 136.3, 135.4, 130.0, 129.9, 128.3, 127.8, 125.7, 123.0, 118.6, 114.2, 93.2, 51.0, 50.7, 31.8, 30.1, 21.7, 21.7, 19.8, 19.7, 13.9, 13.6;

HRMS (ESI) m/z: $[M + H]^+$ Calcd for $C_{27}H_{35}N_4O_4S_2$ 543.2094; Found 543.2092;

5 Density Functional Theory (DFT) Calculations

5.1 Computational Details

To reduce the computational cost, the active gold catalyst $(ArO)_3PAu^+$ was simplified as H_3PAu^+[21] and the tosyl group was simplified as benzenesulfonyl group in the computations. The non-coordinating counterion NTf_2^- was also excluded from the model.[22] All DFT calculations were carried out using Gaussian 16 program[23] and ORCA (v 5.0.3)[24]. Geometry optimization of all structures was carried out using the Gaussian 16 program without any constraints of freedom. The hybrid density functional M06[25] in combination with 6-31+G(d) basis set[26] for H, C, N, O, P and S, and SDD basis set[27] for Au was used since this combination was used in a previously published DFT study on a similar reaction system.[21] Each stationary point was confirmed to be either a local minimum or a transition state by vibrational frequency calculations performed at the same level of theory. Single point electronic energies for each optimized structure were obtained using domain-based local pair natural orbital couple cluster theory with single, double, and perturbative triple excitations (DLPNO-CCSD(T))[28] functional implemented in ORCA (v 5.0.3) program and Def2TZVPP basis set[29] for all atoms using 'Tight' convergence criteria. In both structural optimization and single point calculations, the polarizable continuum model (PCM) was used to model the solvent effects with 1,2-dichloroethane (ε = 10.36) as solvent.[30] Natural Bond Orbital (NBO) analysis was performed using the NBO (version 3)[31] analysis module implemented in Gaussian 16 program package. Energies reported are relative Gibbs free energies at 298 K.

5.2 Additional Results and Discussion

The structures of key intermediates and transition states calculated in the catalytic cycles (Scheme 3a) are visualized using GaussView6 program and shown in below (Figure S9 – S10).

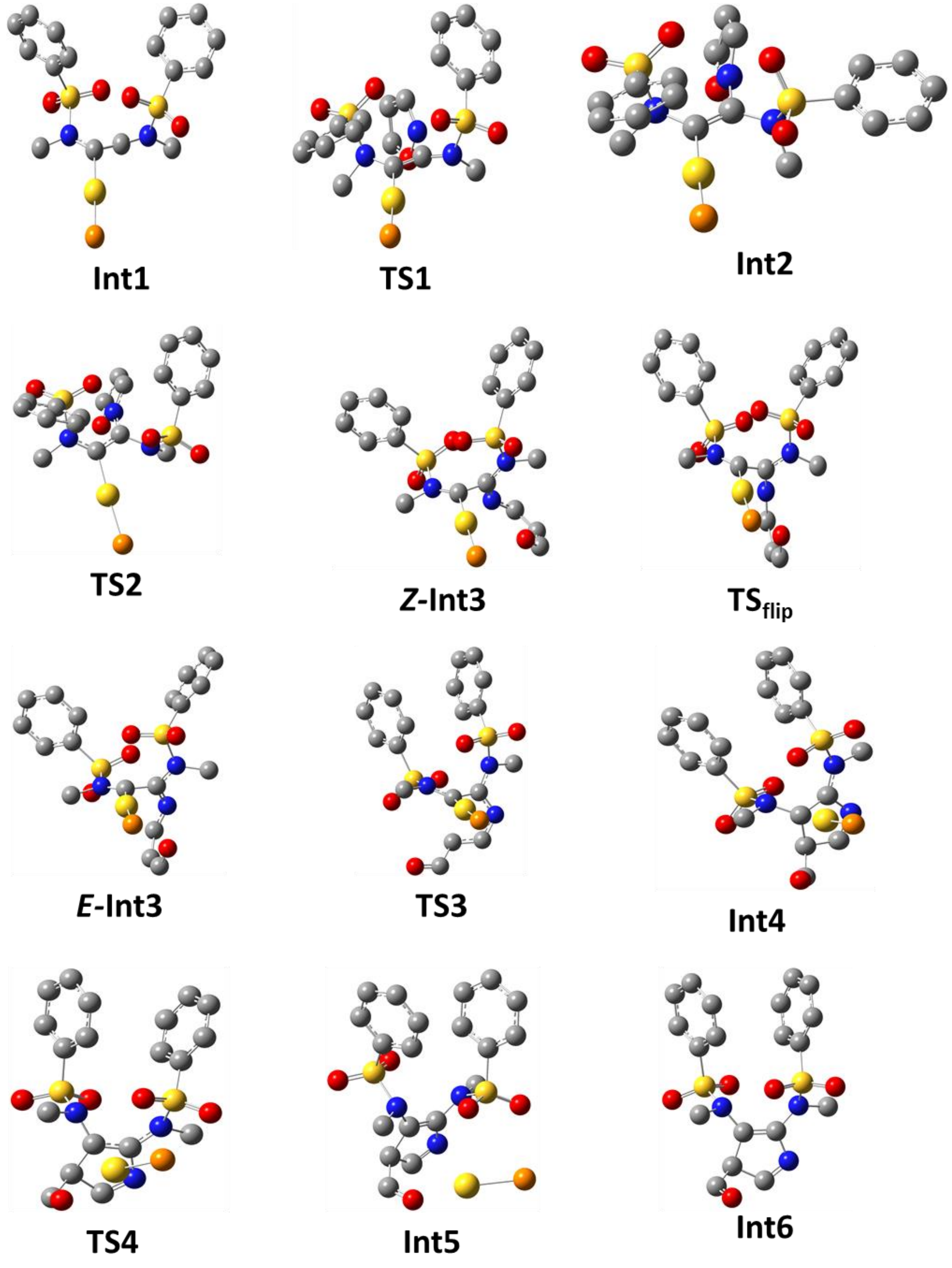

Figure S9. Ball-and-stick structures of key intermediates and transition states in calculated catalytic cycle (Scheme 3a) of symmetrical yndiamide. Colour codes: C – grey; N – blue; O – red; P – orange; S – yellow (bound to O); Au – yellow (bound to P). All hydrogen atoms are hidden for clarity.

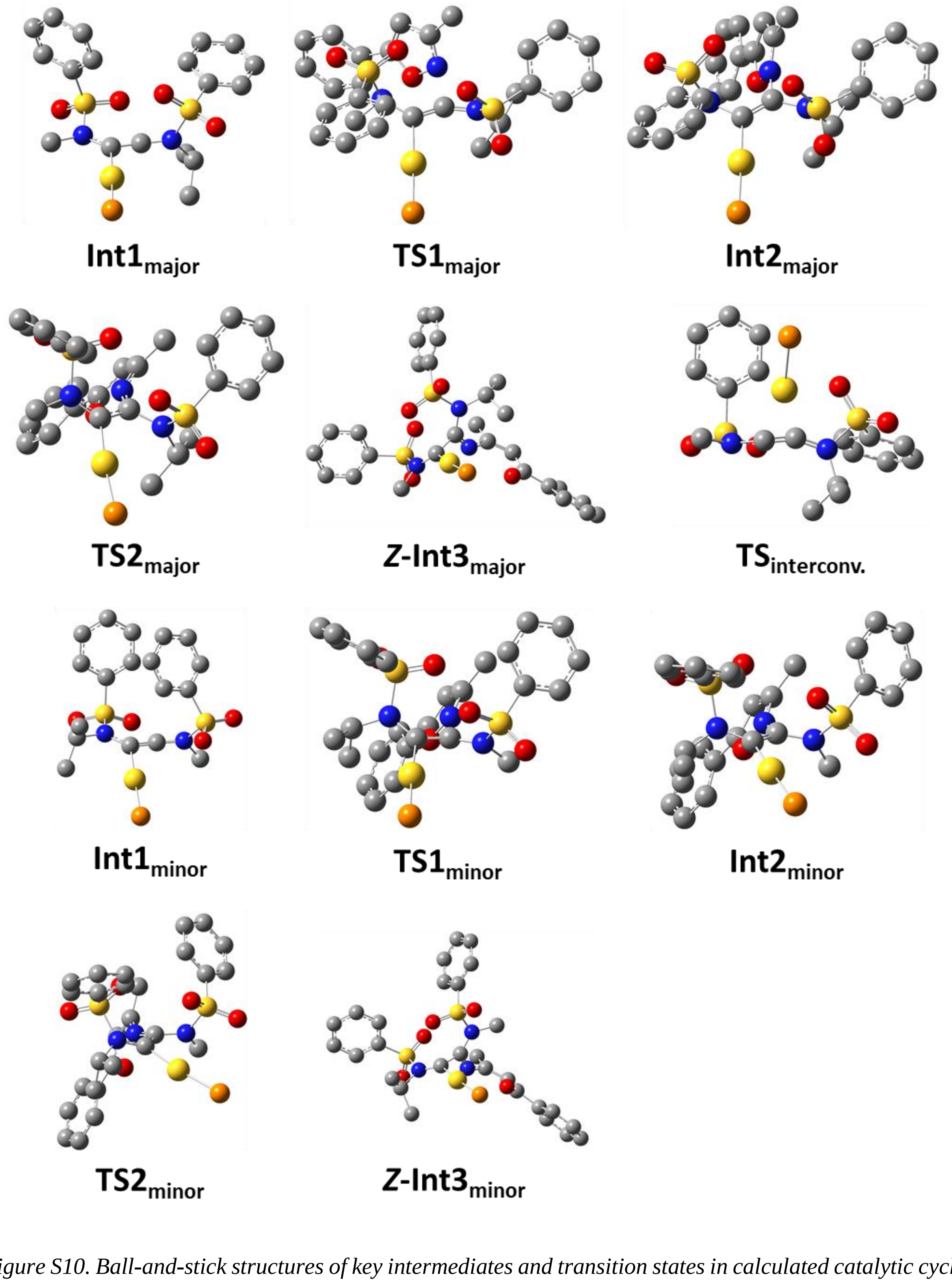

Figure S10. Ball-and-stick structures of key intermediates and transition states in calculated catalytic cycle (Scheme 3b) of unsymmetrical yndiamide. Colour codes: C – grey; N – blue; O – red; P – orange; S – yellow (bound to O); Au – yellow (bound to P).All hydrogen atoms are hidden for clarity.

Ye *et al.* suggested that the conjugation of the phenyl group in ynamide could stabilize the gold carbene moiety.[21] We performed NBO analysis on the gold carbene intermediate ***Z*-Int3** in our catalytic cycle and found a strong stabilization effect from the neighbouring N atom. Based on MO analysis (Figure S11), it is obvious that the N lone pair electrons are in conjugation with the carbene moiety, resulting in 1) enhanced double bond character of the C-N bond with a bond length (1.299 Å) close to that of C=N bond and Wiberg bond index of 1.545; 2) weakened N-S bond with a bond length (1.814 Å), which is significantly longer than that of **Int1** (1.718 Å), and Wiberg bond index of 0.575. This stabilization effect, we believe, has contributed to the large ΔG^o from **Int2** to ***Z*-Int3** that strongly favours the gold carbene formation, and prolonged life-time of the gold carbene species allowing the occurrence of the slower 6π-electrocyclization to afford the oxazepine side-products we observed in our experiments.

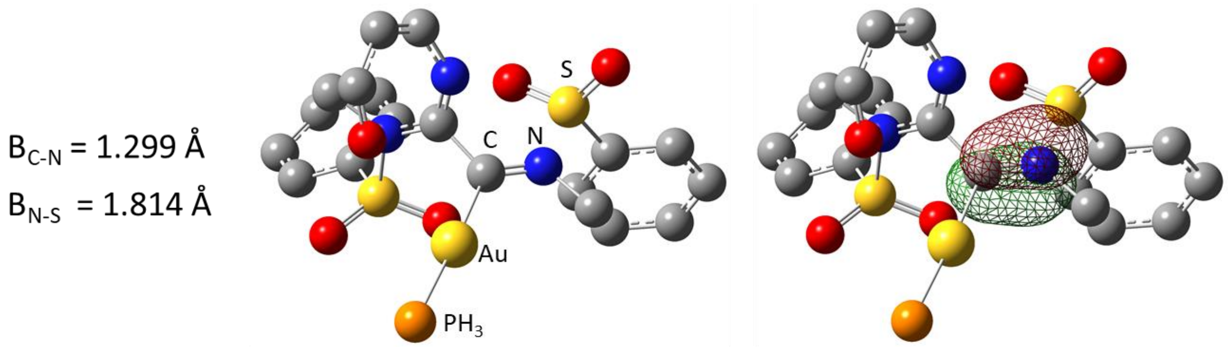

*Figure S11. Ball-and-stick structures of **Z-Int3** (left) and visualization of its carbene-centered MO (isovalue = 0.05) showing conjugation between N atom and the gold carbene moiety (right). All hydrogen atoms are hidden for clarity.*

Apart from the catalytic cycle we discussed in the main text (Scheme 3a), we have also modelled an alternative pathway where the isoxazole attacks the yndiamide in a different orientation (Figure S12), which results in the formation of a series of rotamers of the intermediates and transition states we presented in the main text. The reaction energy profiles of the two cycles are identical until the formation of the keteneiminium-gold complex **Int1**. The isoxazole then approach the yndiamide in a different orientation to afford a rotamer (**Int2$_{rot}$**) of **Int2** via **TS1$_{rot}$**. The major structural difference between **TS1$_{rot}$/Int2$_{rot}$** and **TS1/Int2** is the angle between the plane of isoxazole and the plane of the yndiamide moiety: that of **TS1$_{rot}$/Int2$_{rot}$** is nearly parallel while that of **TS1/Int2** is close to perpendicular. Despite the structural difference, the activation energy barrier of both pathways, however are almost identical ($\Delta\Delta G^‡ \approx 1.0$ kcal mol^{-1}). Even though **Int2$_{rot}$** turns out more stable than **Int2** (presumably due to conjugation of the isoxazole group), the ΔG^o of this process is still small (~ -3.9 kcal mol^{-1}), rendering this process significantly

reversible. Thanks to the relative orientation between isoxazole and yndiamide moiety in **Int2rot**, its subsequent ring-opening/gold carbene formation step produced ***E-Int3rot*** directly ($\Delta G^{\ddagger}$ = 15.5 kcal mol^{-1})

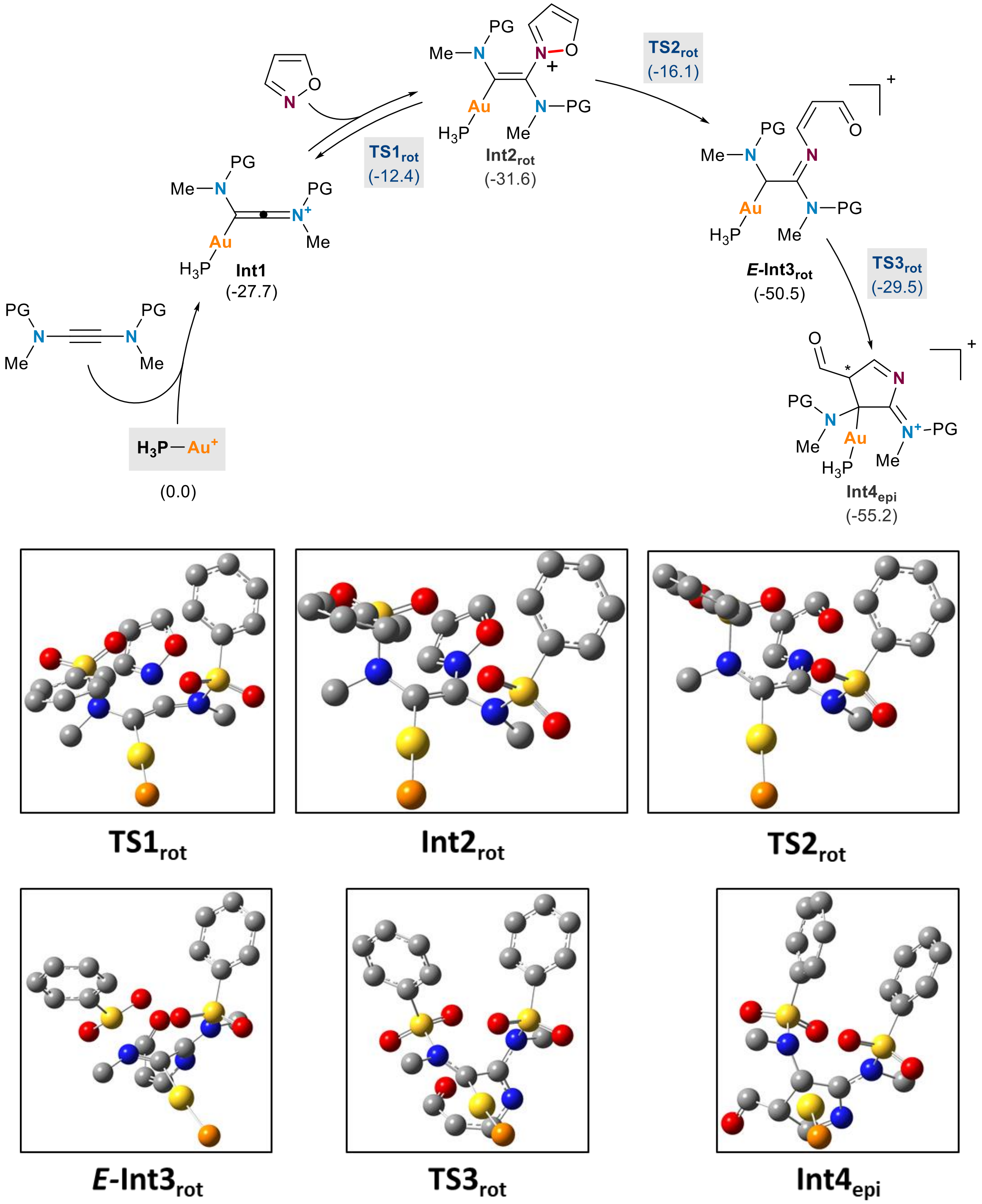

Figure S12. Alternative attacking of isoxazole onto symmetrical yndiamide and ball-and-stick structures of key intermediate and transition states. All hydrogen atoms are hidden for clarity. Relative Gibbs free energies are reported in kcal mol^{-1}.

without the need to go through a flipping from a *Z*-isomer of the imine moiety. However, the α,β-unsaturated aldehyde side-chain on the imine moiety of ***E-Int3$_{rot}$*** lies in a conformation that orients C=C further away from the gold carbene moiety, compared to that of ***E-Int3$_{rot}$***, it hence requires a much higher activation energy barrier to cyclize ($\Delta G^{\ddagger}$(**TS3$_{rot}$**) = 21.0 kcal mol^{-1} *vs.* $\Delta G^{\ddagger}$(**TS3**) = 13.2 kcal mol^{-1}). Considering that both $\Delta G^{\ddagger}$(**TS2$_{rot}$**) and $\Delta G^{\ddagger}$(**TS3$_{rot}$**) are higher than that of their rotamers (in Scheme 3a), the pathway we discussed here is less likely to be favoured compared to the one we discussed in the main text. However, since the formation of **Int2$_{rot}$** is slightly more favoured ($\Delta G^{\ddagger}$(**TS1$_{rot}$**) being 1.0 kcal mol^{-1} smaller than $\Delta G^{\ddagger}$(**TS1**)), it is highly possible that **Int2$_{rot}$** is formed predominantly and readily undergoes conformational isomerization to form **Int2** to finish the rest of the cycle. To simplify the scenario, we hence decided to present the mechanism as Scheme 3a in the main text and to study the regioselectivity on that model.

5.3 Cartesian Coordinates and Total Electronic Energies (E_{total}/hatree) of Optimized Structures

Symmetrical yndiamide (PGMeYndPGMe), E_{total} = - 1823.397136755912

C	-0.58223	1.87188	0.17385	C	-2.90249	-0.63676	0.08022
C	0.58267	1.87200	-0.17223	C	-4.26646	-0.87442	-0.06406
N	-1.84453	1.83684	0.62937	C	-1.96419	-1.12534	-0.82914
N	1.84497	1.83735	-0.62778	C	-4.69766	-1.64389	-1.14133
S	-2.33407	0.38689	1.41414	H	-4.97160	-0.46742	0.65762
O	-1.12502	-0.18318	1.99161	C	-2.41233	-1.88347	-1.90413
O	-3.47106	0.73786	2.25446	H	-0.90215	-0.91358	-0.71380
S	2.33444	0.38807	-1.41386	C	-3.77420	-2.14482	-2.05643
O	1.12545	-0.18123	-1.99221	H	-5.75885	-1.84869	-1.26580
O	3.47172	0.73967	-2.25352	H	-1.69344	-2.27031	-2.62362
C	2.90234	-0.63702	-0.08082	C	-2.89126	2.53605	-0.13539
C	4.26626	-0.87466	0.06386	H	-3.82671	2.50705	0.43038
C	1.96365	-1.12668	0.82757	C	2.89172	2.53571	0.13773
C	4.69703	-1.64523	1.14053	H	3.82711	2.50747	-0.42819
H	4.97171	-0.46683	-0.65705	H	3.03242	2.07813	1.12642
C	2.41136	-1.88588	1.90197	H	-4.11800	-2.74175	-2.89898
H	0.90166	-0.91494	0.71193	H	4.11667	-2.74500	2.89674
C	3.77320	-2.14723	2.05466	H	2.58126	3.57710	0.25506
H	5.75819	-1.85003	1.26528	H	-3.03181	2.07964	-1.12464
H	1.69217	-2.27355	2.62071	H	-2.58088	3.57762	-0.25140

No. of Imaginary frequencies: 0

H_3PAu^+, E_{total} = - 477.923765901479

Au	-0.44167	0.00001	0.00000	H	2.43123	-1.23932	-0.26136
P	1.83971	0.00001	-0.00000	H	2.43265	0.84571	-0.94215
H	2.43240	0.39290	1.20354				

No. of Imaginary frequencies: 0

Isoxazole, E_{total} = - 245.648887840052

C	1.10115	0.22692	0.00006	N	-0.82726	-0.87993	-0.00018
O	0.55571	-0.99765	-0.00019	H	0.24162	2.25098	0.00044
C	-1.06316	0.40974	0.00008	H	2.18342	0.26057	0.00007
C	0.13060	1.17564	0.00022	H	-2.09145	0.75538	0.00014

No. of Imaginary frequencies: 0

Int1, E_{total} = - 2301.384800098657

C	-0.15507	-0.72373	-1.09211	C	4.89431	-3.54833	0.03291
C	-0.89276	0.31506	-0.80039	H	2.94313	-4.36883	0.49348
N	0.50770	-1.79567	-0.90206	C	5.01460	-1.13074	-0.11964
N	-0.67576	1.63829	-0.99716	H	3.16400	-0.06306	0.21224
S	1.22134	-2.10135	0.73441	C	5.62741	-2.38259	-0.18466
O	0.90896	-0.89433	1.48116	H	5.38016	-4.52007	-0.01910
O	0.70038	-3.39952	1.13444	H	5.59482	-0.22708	-0.29224
S	0.89873	2.19789	-1.39738	C	0.74459	-2.84863	-1.90524
O	1.68932	0.97607	-1.52051	H	0.49897	-3.82298	-1.47209
O	0.77652	3.11738	-2.51859	C	-1.69208	2.65097	-0.69570
C	1.38624	3.10460	0.04369	H	-1.35287	3.62280	-1.06681
C	1.61556	4.47363	-0.06216	H	-1.86301	2.71279	0.38621
C	1.51917	2.41330	1.24837	H	6.69025	-2.44940	-0.40874
C	2.00596	5.16909	1.07943	H	2.45453	5.04651	3.17986
H	1.49354	4.97859	-1.01807	H	-2.62185	2.39535	-1.21286
C	1.90698	3.12495	2.37737	Au	-2.71381	-0.43856	-0.00558
H	1.31729	1.34269	1.30741	P	-4.70840	-1.22147	0.94305
C	2.15096	4.49675	2.29094	H	-4.58831	-1.99733	2.10356
H	2.19507	6.23869	1.01964	H	-5.62500	-0.24424	1.35309
H	2.01782	2.60750	3.32797	H	-5.51379	-2.04020	0.14016
C	2.94597	-2.21114	0.38845	H	0.09840	-2.64674	-2.76151
C	3.53520	-3.47277	0.31892	H	1.79397	-2.82739	-2.21811
C	3.65863	-1.03195	0.16833				

No. of Imaginary frequencies: 0

TS1, E_{total} = - 2547.035437506046

C	0.86361	-0.38223	-1.03629	O	0.62259	-1.83660	1.45050
C	-0.24402	0.31490	-0.96533	O	2.11917	-3.59891	0.37949
N	1.46039	-1.53781	-0.95189	S	-0.21832	2.40066	0.65500
N	-0.30696	1.72422	-0.91756	O	0.94769	1.79579	1.28331
S	1.77704	-2.21924	0.66091	O	-0.32124	3.83550	0.45779

C	-1.66866	1.80905	1.49227
C	-2.85546	2.53262	1.38492
C	-1.60207	0.59395	2.17458
C	-4.00602	2.01959	1.97693
H	-2.86802	3.49047	0.86713
C	-2.76085	0.09878	2.76801
H	-0.66010	0.05170	2.24863
C	-3.95738	0.80738	2.66550
H	-4.93855	2.57708	1.91563
H	-2.72033	-0.83769	3.32244
C	3.20441	-1.32989	1.20310
C	4.45616	-1.72745	0.73079
C	3.03769	-0.24746	2.06346
C	5.57219	-0.99799	1.12371
H	4.55319	-2.60194	0.08918
C	4.16831	0.46396	2.45407
H	2.04723	0.03973	2.40876
C	5.42636	0.09345	1.98117
H	6.55941	-1.29034	0.77234
H	4.06134	1.30811	3.13283
C	2.23724	-2.05786	-2.08433
H	3.21624	-1.56171	-2.12595
C	-1.33016	2.31302	-1.78796
H	-1.25539	3.40238	-1.73389
H	-2.34906	1.98752	-1.51711
H	6.30614	0.65414	2.29193
H	-4.85745	0.42186	3.14223
H	-1.11768	1.99229	-2.81337
Au	-1.97800	-0.87926	-0.76865
P	-3.95181	-2.12143	-0.44101
H	-4.00227	-2.92030	0.71324
H	-5.13530	-1.38013	-0.28953
H	-4.34924	-3.04829	-1.41785
N	2.36363	0.96322	-1.52342
O	2.10935	1.39961	-2.80418
C	3.00044	1.93350	-0.90783
C	2.61806	2.62997	-2.93279
C	3.20059	3.03240	-1.77143
H	3.69360	3.97065	-1.55851
H	3.27607	1.80898	0.13506
H	2.50044	3.08445	-3.90884
H	2.35439	-3.13800	-1.96946
H	1.68257	-1.83925	-3.00181

Imaginary frequencies (cm^{-1}): -227.43

TS1$_{rot}$, E$_{total}$ = - 2547.034823034876

C	0.72762	-0.35058	-1.23883
C	-0.44726	0.23719	-1.17885
N	1.43457	-1.41810	-0.99972
N	-0.64494	1.62499	-1.29269
S	1.65488	-2.00781	0.66795
O	0.50766	-1.49369	1.39298
O	1.88819	-3.42645	0.47879
S	-0.29666	2.55353	0.11621
O	0.94918	2.02756	0.65360
O	-0.41774	3.93716	-0.31100
C	-1.61944	2.15600	1.23058
C	-2.78083	2.92658	1.21013
C	-1.49604	1.02406	2.03572
C	-3.84800	2.54530	2.01814
H	-2.83635	3.81638	0.58508
C	-2.57087	0.66050	2.84234
H	-0.57481	0.44422	2.03960
C	-3.74323	1.41492	2.82822
H	-4.75965	3.13941	2.02316
H	-2.48298	-0.20934	3.49153
C	3.13025	-1.22156	1.23120
C	4.35035	-1.85766	1.00464
C	3.03322	0.01907	1.85673
C	5.51355	-1.21659	1.41696
H	4.38148	-2.84103	0.53849
C	4.20711	0.64137	2.26678
H	2.06535	0.49438	2.00239
C	5.43953	0.02697	2.04514
H	6.47788	-1.69613	1.26222
H	4.15575	1.60796	2.76413
C	2.18866	-2.12171	-2.04934
H	1.93259	-3.18410	-2.03415
C	-1.87985	2.02366	-1.97836
H	-1.87942	3.10945	-2.10992
H	-2.78264	1.71220	-1.42626
H	6.35375	0.51740	2.37578
H	-4.57850	1.12966	3.46581
H	-1.88732	1.54832	-2.96391
Au	-2.03008	-1.06499	-0.66877
P	-3.81948	-2.43475	0.01199

H	-3.50086	-3.68113	0.57437
H	-4.64758	-1.90377	1.01497
H	-4.77920	-2.81651	-0.93910
N	2.12317	1.02439	-1.84848
O	3.30843	0.77044	-1.20662
C	2.19892	2.24458	-2.32797
C	4.08414	1.85542	-1.31648
C	3.44716	2.82827	-2.02099
H	3.80477	3.81544	-2.27838
H	1.35220	2.66340	-2.85971
H	5.05881	1.77766	-0.85021
H	3.26618	-1.99331	-1.88998
H	1.91170	-1.68053	-3.00993

Imaginary frequencies (cm^{-1}): -232.17

Int2, E_{total} = - 2547.065238218542

C	0.70259	1.15387	-0.75500
C	-0.59631	0.80387	-0.86886
N	1.81333	0.28297	-0.77790
N	-1.58493	1.83115	-0.76212
S	2.06142	-0.57488	0.67505
O	1.68613	0.38076	1.71538
O	1.41315	-1.88467	0.63189
S	-2.09879	2.18523	0.84012
O	-0.90261	2.57859	1.57175
O	-3.22803	3.08559	0.69065
C	-2.65926	0.63675	1.50593
C	-3.98644	0.25481	1.31615
C	-1.73121	-0.19368	2.13467
C	-4.39069	-0.99811	1.76861
H	-4.69047	0.93829	0.84388
C	-2.15133	-1.44507	2.57848
H	-0.70403	0.13952	2.28243
C	-3.47448	-1.84470	2.39355
H	-5.42673	-1.30777	1.64504
H	-1.44013	-2.10159	3.07794
C	3.81622	-0.82025	0.66700
C	4.32065	-2.11093	0.54036
C	4.64673	0.29005	0.81065
C	5.70160	-2.28933	0.55499
H	3.63916	-2.95342	0.44038
C	6.02195	0.09680	0.82299
H	4.21961	1.28649	0.91717
C	6.54556	-1.19053	0.69457
H	6.11700	-3.29030	0.45916
H	6.68829	0.94920	0.93729
C	2.11842	-0.42289	-2.02546
H	3.10007	-0.90362	-1.94183
C	-2.68885	1.67724	-1.71883
H	-3.35872	2.53615	-1.63308
H	-3.24809	0.73926	-1.56122
H	7.62409	-1.33628	0.70620
H	-3.80118	-2.81872	2.75608
H	-2.25751	1.66098	-2.72466
Au	-1.15471	-1.21060	-0.97752
P	-1.74143	-3.49781	-0.82551
H	-1.11576	-4.17422	0.23627
H	-3.08601	-3.82284	-0.57582
H	-1.46539	-4.37789	-1.88545
N	1.07590	2.53716	-0.58181
O	1.00903	3.34373	-1.69045
C	1.45010	3.24912	0.46279
C	1.34983	4.56646	-1.27857
C	1.64686	4.57665	0.05294
H	1.94614	5.41778	0.66213
H	1.53719	2.76008	1.42848
H	1.33605	5.32887	-2.04787
H	1.35919	-1.18057	-2.27067
H	2.16605	0.31780	-2.83065

Imaginary frequencies: none

Int2$_{rot}$, E_{total} = - 2547.068534403312

C	0.85930	-0.23502	-1.20945
C	-0.44915	0.12405	-1.14985
N	1.36686	-1.47043	-0.80753
N	-0.81295	1.46479	-1.43106
S	1.56630	-1.81417	0.86693
O	0.43171	-1.20374	1.54458
O	1.80084	-3.24705	0.91492
S	-0.40111	2.67337	-0.27347
O	0.91145	2.30282	0.24193
O	-0.60793	3.94293	-0.95047
C	-1.60840	2.48785	1.01082
C	-2.68710	3.36762	1.06561

C	-1.47409	1.41792	1.89534
C	-3.65444	3.17147	2.04734
H	-2.75254	4.19439	0.36034
C	-2.45375	1.23420	2.86539
H	-0.62315	0.74028	1.83312
C	-3.53823	2.10772	2.94050
H	-4.49958	3.85355	2.11498
H	-2.36070	0.41011	3.57049
C	3.03749	-0.96366	1.37466
C	4.27190	-1.57209	1.15190
C	2.92734	0.31403	1.91760
C	5.42852	-0.86786	1.47150
H	4.32118	-2.58580	0.75710
C	4.09298	1.00276	2.23786
H	1.94919	0.76755	2.06738
C	5.33748	0.41605	2.00996
H	6.40213	-1.32822	1.31508
H	4.02640	1.99872	2.67199
C	2.21623	-2.20918	-1.74371
H	2.30384	-3.24475	-1.40622
C	-2.15801	1.64651	-1.99108
H	-2.29520	2.69764	-2.26178
H	-2.94287	1.33266	-1.28321
H	6.24603	0.95757	2.26872
H	-4.29795	1.96164	3.70639
H	-2.23825	1.03822	-2.89711
Au	-1.84517	-1.31174	-0.54285
P	-3.38262	-2.95726	0.16852
H	-3.00340	-3.74070	1.27101
H	-4.65612	-2.53674	0.58713
H	-3.74326	-3.96527	-0.74105
N	1.84624	0.66823	-1.76425
O	2.98545	0.90266	-1.04108
C	1.81718	1.48972	-2.80061
C	3.63389	1.88646	-1.65944
C	2.95363	2.30563	-2.76807
H	3.23216	3.07876	-3.47011
H	0.98310	1.45736	-3.49177
H	4.57752	2.16559	-1.20541
H	3.22304	-1.77320	-1.83973
H	1.72323	-2.20469	-2.72222

Imaginary frequencies: none

TS2, E_{total} = - 2547.041018840560

C	-0.81127	-0.42526	0.86197
C	0.56425	-0.13798	0.92536
N	-1.22131	-1.56215	0.11651
N	1.01632	1.05996	1.36288
S	-1.48779	-1.31555	-1.54689
O	-0.38695	-0.47465	-1.99962
O	-1.71754	-2.63827	-2.10352
S	0.30040	2.61605	0.92716
O	-0.98242	2.34629	0.30139
O	0.41349	3.46741	2.09539
C	1.44090	3.13147	-0.32617
C	2.28135	4.21309	-0.08434
C	1.45372	2.41753	-1.52434
C	3.16067	4.60047	-1.09248
H	2.23697	4.74000	0.86739
C	2.34233	2.81625	-2.51513
H	0.78547	1.56763	-1.67758
C	3.19022	3.90388	-2.29850
H	3.82390	5.44805	-0.93304
H	2.37040	2.27861	-3.46067
C	-2.98593	-0.36464	-1.65240
C	-4.20968	-1.01936	-1.50982
C	-2.90387	1.01730	-1.81069
C	-5.37712	-0.26151	-1.50499
H	-4.24533	-2.10474	-1.42647
C	-4.08065	1.76168	-1.80878
H	-1.93425	1.49838	-1.92102
C	-5.31110	1.12443	-1.65020
H	-6.34126	-0.75543	-1.40170
H	-4.03404	2.84152	-1.93725
C	-2.00998	-2.58295	0.80032
H	-2.16853	-3.42762	0.12480
C	2.43065	1.19238	1.74670
H	2.57151	2.13612	2.28243
H	3.08534	1.16138	0.86438
H	-6.22811	1.71107	-1.65254
H	3.88203	4.21086	-3.08074
H	2.67956	0.37394	2.42640
Au	1.84981	-1.63224	0.22164
P	3.23483	-3.34318	-0.64454
H	3.75538	-4.29173	0.25051
H	2.65908	-4.18790	-1.60705
H	4.40738	-2.95630	-1.31313
N	-1.68792	0.15307	1.69627

O	-1.62250	-0.44878	3.25928	H	-1.43460	-2.92809	1.66781
C	-2.90548	0.67271	1.68897	H	-2.98695	-2.20447	1.14554
C	-2.72726	-0.04580	3.77409	H	-3.22324	1.16872	0.77106
C	-3.58873	0.58877	2.89204	H	-2.86339	-0.19507	4.84665
H	-4.55659	1.01502	3.11611				

Imaginary frequencies: -531.32 cm^{-1}

TS2$_{rot}$, E$_{total}$ = - 2547.043234790500

C	-0.72038	-0.48080	1.13446	C	-5.40269	0.25250	-1.76437
C	0.60198	-0.03754	0.99277	H	-6.22637	-1.70584	-1.38468
N	-1.10698	-1.68958	0.52862	H	-4.31603	2.07829	-2.14086
N	1.00727	1.23250	1.26525	C	-1.95667	-2.60317	1.29215
S	-1.34712	-1.66973	-1.17166	H	-2.02796	-3.55188	0.75399
O	-0.32398	-0.77213	-1.69753	C	2.43549	1.48396	1.51532
O	-1.39217	-3.07142	-1.55364	H	2.55540	2.48968	1.93032
S	0.16457	2.66663	0.69928	H	3.02321	1.38992	0.59111
O	-1.13933	2.21960	0.24599	H	-6.37849	0.71754	-1.89460
O	0.30208	3.66164	1.74825	H	3.30885	4.06285	-3.72784
C	1.16056	3.11459	-0.69410	H	2.79151	0.76217	2.25474
C	1.91048	4.28567	-0.64073	Au	1.95482	-1.51152	0.34744
C	1.16511	2.26495	-1.80053	P	3.38806	-3.19963	-0.46471
C	2.68302	4.62455	-1.74834	H	3.91522	-4.12421	0.45118
H	1.87813	4.91613	0.24615	H	2.83311	-4.06870	-1.41779
C	1.94775	2.61849	-2.89332	H	4.56025	-2.80406	-1.12879
H	0.57552	1.34559	-1.80703	N	-1.60772	0.09194	1.98257
C	2.70078	3.79366	-2.86614	O	-3.05818	0.69815	1.40661
H	3.27235	5.53898	-1.73558	C	-1.48200	0.76311	3.12084
H	1.96717	1.97544	-3.77085	C	-3.43544	1.46933	2.35599
C	-2.93305	-0.92883	-1.44644	C	-2.53023	1.62106	3.39963
C	-4.07821	-1.71461	-1.31815	H	-2.65661	2.21472	4.29423
C	-2.99717	0.43087	-1.73906	H	-0.61084	0.56369	3.74187
C	-5.32044	-1.10961	-1.47467	H	-4.44593	1.87862	2.29286
H	-3.99435	-2.78293	-1.12521	H	-2.96475	-2.19718	1.46583
C	-4.24713	1.01853	-1.90275	H	-1.47277	-2.78839	2.25746
H	-2.08305	1.01285	-1.83306				

Imaginary frequencies: -551.02 cm^{-1}

***Z*-Int3, E$_{total}$ = - 2547.090365723968**

C	-0.42918	0.47508	1.23047	O	2.11963	-0.15149	1.66962
C	-0.47360	-0.75948	0.38962	O	1.76559	-2.46546	2.64283
N	-0.25193	1.71101	0.65127	C	3.06841	-2.24008	0.34657
N	0.36759	-1.73379	0.56610	C	3.65092	-3.47752	0.61258
S	0.32863	1.89124	-0.94719	C	3.36146	-1.49478	-0.79632
O	0.80946	0.56064	-1.31815	C	4.57509	-3.97611	-0.30007
O	-0.70968	2.53310	-1.73752	H	3.39167	-4.02843	1.51443
S	1.91333	-1.57914	1.50362	C	4.28031	-2.01607	-1.69849

H	2.87096	-0.54013	-0.98295
C	4.88471	-3.24894	-1.44836
H	5.05174	-4.93562	-0.11285
H	4.52637	-1.45763	-2.59879
C	1.68849	3.00988	-0.77039
C	1.62351	4.24916	-1.40306
C	2.79369	2.60706	-0.02162
C	2.71218	5.10845	-1.28423
H	0.74174	4.52901	-1.97552
C	3.86877	3.48026	0.08914
H	2.80592	1.63577	0.47035
C	3.82714	4.72464	-0.54161
H	2.68591	6.08033	-1.77206
H	4.74135	3.18898	0.66979
C	-0.58351	2.93389	1.38578
H	-0.33576	3.80175	0.76825
C	0.18361	-3.06792	-0.03109
H	0.59311	-3.82493	0.64456
H	0.69107	-3.11465	-1.00073
H	4.67369	5.40251	-0.45076
H	5.60580	-3.64774	-2.15912
H	-0.88738	-3.24362	-0.15478
Au	-2.22375	-0.88419	-0.76718
P	-4.20675	-0.94082	-2.03393
H	-4.10210	-0.62986	-3.39640
H	-4.90444	-2.15533	-2.08151
H	-5.21400	-0.05686	-1.62258
N	-0.76716	0.23304	2.43107
O	-3.40350	0.98003	1.45403
C	-1.28922	0.86530	3.50506
C	-3.57829	1.29588	2.63084
C	-2.56415	1.32375	3.65857
H	-2.85552	1.67995	4.64452
H	-1.65339	2.96079	1.61017
H	0.00891	2.98862	2.30616
H	-0.62813	0.86574	4.37669
H	-4.59477	1.58957	2.97056

Imaginary frequencies: none

TS_{flip}, E_{total} = - 2547.078093619125

C	-0.38990	0.40271	1.23690
C	-0.46781	-0.75682	0.30012
N	-0.19246	1.67745	0.77109
N	0.34053	-1.76834	0.38666
S	0.44294	1.99919	-0.78425
O	0.91003	0.69596	-1.25382
O	-0.56017	2.73211	-1.53327
S	1.89533	-1.73455	1.34164
O	2.12984	-0.33002	1.61478
O	1.70406	-2.70953	2.39480
C	3.03410	-2.33306	0.13669
C	3.57784	-3.60427	0.31222
C	3.35395	-1.51384	-0.94687
C	4.48900	-4.06328	-0.63312
H	3.30005	-4.20709	1.17503
C	4.25884	-1.99748	-1.88376
H	2.89195	-0.53317	-1.06146
C	4.82378	-3.26362	-1.72444
H	4.93719	-5.04747	-0.51627
H	4.52499	-1.38324	-2.74114
C	1.80818	3.07242	-0.44663
C	1.77233	4.36737	-0.95837
C	2.88146	2.59188	0.30393
C	2.85680	5.20474	-0.71380
H	0.91467	4.70232	-1.53853
C	3.95243	3.44501	0.54048
H	2.87266	1.57821	0.70385
C	3.93955	4.74414	0.03156
H	2.85326	6.21927	-1.10655
H	4.80006	3.09494	1.12590
C	-0.60640	2.80435	1.60861
H	-0.60574	3.71522	1.00281
C	0.11290	-3.04208	-0.31056
H	0.42134	-3.86636	0.34111
H	0.68696	-3.06497	-1.24368
H	4.78355	5.40474	0.22114
H	5.53437	-3.63118	-2.46228
H	-0.95506	-3.13093	-0.52443
Au	-2.23680	-0.64867	-0.82701
P	-4.22442	-0.29782	-2.03713
H	-4.12771	0.31900	-3.29367
H	-5.04017	-1.39725	-2.34629
H	-5.13510	0.54538	-1.38263
N	-0.72463	0.15690	2.42530
O	-3.38831	0.67044	1.36997
C	-1.47040	-0.08352	3.48559
C	-3.72095	0.35981	2.52047
C	-2.84031	-0.01295	3.58536

H	-3.27834	-0.25449	4.55089
H	-1.62508	2.62517	1.96839
H	0.08394	2.92455	2.45238
H	-0.89724	-0.36640	4.37322
H	-4.79957	0.36898	2.78728

Imaginary frequencies: -98.07 cm^{-1}

***E*-Int3, E_{total} = - 2547.095061749579**

C	0.31647	-0.43345	1.32282
C	0.56754	0.61279	0.28798
N	0.03612	-1.72114	0.91274
N	-0.08653	1.73220	0.25740
S	-0.66011	-2.06509	-0.59638
O	-0.96409	-0.74761	-1.15775
O	0.22124	-2.97329	-1.30692
S	-1.65566	2.02409	1.14976
O	-2.07378	0.71020	1.59664
O	-1.35072	3.09408	2.07815
C	-2.68527	2.60681	-0.15551
C	-3.03892	3.95552	-0.16681
C	-3.11742	1.70051	-1.12499
C	-3.86752	4.40672	-1.18815
H	-2.68241	4.62491	0.61404
C	-3.93729	2.17666	-2.14070
H	-2.80578	0.65666	-1.09378
C	-4.31041	3.52089	-2.16919
H	-4.16782	5.45178	-1.21647
H	-4.28772	1.49477	-2.91234
C	-2.15041	-2.92341	-0.17786
C	-2.30910	-4.23058	-0.63058
C	-3.12329	-2.26888	0.57837
C	-3.49036	-4.89898	-0.32164
H	-1.52292	-4.70511	-1.21452
C	-4.29401	-2.95447	0.87910
H	-2.96381	-1.25095	0.93472
C	-4.47614	-4.26251	0.42852
H	-3.63792	-5.91986	-0.66753
H	-5.06628	-2.46678	1.47031
C	0.30340	-2.83004	1.82895
H	0.26826	-3.76769	1.26589
C	0.32530	2.87421	-0.57264
H	0.06539	3.80478	-0.05808
H	-0.17856	2.82680	-1.54489
H	-5.39668	-4.79120	0.66882
H	-4.95593	3.88212	-2.96749
H	1.40915	2.82946	-0.70644
Au	2.30650	0.16969	-0.81063
P	4.23755	-0.52655	-1.96084
H	4.08648	-1.56819	-2.88857
H	4.95754	0.40650	-2.72254
H	5.24698	-1.03975	-1.13168
N	0.63351	-0.22644	2.53613
O	3.41093	-0.29856	1.74445
C	1.11268	0.87921	3.16714
C	3.50990	0.61269	2.56323
C	2.40950	1.28396	3.22400
H	2.65109	2.11985	3.87769
H	-0.43664	-2.85159	2.63789
H	1.30629	-2.70094	2.24876
H	0.36910	1.40167	3.77870
H	4.51967	0.97492	2.85331

Imaginary frequencies: none

***E*-Int3$_{rot}$, E_{total} = - 2547.093290532666**

C	-0.37909	0.21770	1.42316
C	-0.75811	-0.65042	0.27441
N	-0.01839	1.53827	1.22896
N	-0.09182	-1.71651	-0.04383
S	0.19986	2.20241	-0.30934
O	0.31074	1.03413	-1.19048
O	-0.84477	3.18135	-0.55983
S	1.64698	-2.01243	0.47512
O	2.05651	-0.74994	1.04971
O	1.60818	-3.25057	1.22299
C	2.40703	-2.27500	-1.09570
C	2.85216	-3.55887	-1.40796
C	2.55012	-1.19233	-1.96469
C	3.47396	-3.75758	-2.63603
H	2.72391	-4.37311	-0.69709
C	3.16660	-1.41660	-3.18967
H	2.17117	-0.20572	-1.69834
C	3.62727	-2.69142	-3.52055
H	3.83990	-4.74715	-2.90070
H	3.28896	-0.59197	-3.88849

C	1.75477	3.03663	-0.17996
C	1.81138	4.38049	-0.54177
C	2.87648	2.32651	0.24859
C	3.04141	5.02910	-0.48126
H	0.90980	4.89965	-0.86074
C	4.09340	2.99423	0.30787
H	2.79507	1.28183	0.54838
C	4.17465	4.33814	-0.05887
H	3.11118	6.07819	-0.76074
H	4.98080	2.46440	0.64773
C	0.05564	2.40694	2.40553
H	0.35671	3.41026	2.08994
C	-0.60625	-2.74459	-0.95893
H	-0.19994	-3.71841	-0.66748
H	-0.31148	-2.50884	-1.98821
H	5.13249	4.85269	-0.00908
H	4.11217	-2.85512	-4.48106
H	-1.69582	-2.77385	-0.87434
Au	-2.62664	-0.12192	-0.52512
P	-4.66689	0.66692	-1.41389
H	-4.57847	1.85210	-2.15891
H	-5.36372	-0.16625	-2.30135
H	-5.67629	0.99306	-0.49634
N	-0.60098	-0.19197	2.60836
O	1.58967	-1.00126	4.11424
C	-1.06724	-1.42568	2.99081
C	0.90742	-1.97088	4.38931
C	-0.41222	-2.26073	3.82529
H	-0.90324	-3.18416	4.12692
H	-2.07660	-1.69078	2.65028
H	1.26667	-2.71516	5.13559
H	0.79603	1.99668	3.10252
H	-0.92147	2.45845	2.89705

Imaginary frequencies: none

TS3, E_{total} = - 2547.077181850981

C	0.54674	-0.34228	1.60750
C	0.94701	0.47449	0.43399
N	-0.51554	-1.20874	1.62026
N	0.12319	1.27413	-0.25845
S	-0.97282	-2.13839	0.24216
O	-0.43205	-1.43972	-0.91939
O	-0.58359	-3.51419	0.48712
S	-1.12577	2.20874	0.55216
O	-1.34806	1.51533	1.81530
O	-0.67066	3.58785	0.57116
C	-2.51094	2.04826	-0.54327
C	-3.50568	3.01759	-0.41252
C	-2.62048	0.98074	-1.43171
C	-4.65108	2.89708	-1.19193
H	-3.38087	3.85211	0.27592
C	-3.76640	0.88905	-2.21488
H	-1.82979	0.23611	-1.51821
C	-4.77884	1.83766	-2.08991
H	-5.43985	3.64139	-1.10612
H	-3.86609	0.06810	-2.92268
C	-2.74394	-2.01475	0.28037
C	-3.43284	-2.98493	-0.44770
C	-3.39815	-0.97189	0.93242
C	-4.81810	-2.89333	-0.53041
H	-2.89591	-3.80065	-0.92975
C	-4.78549	-0.90560	0.84986
H	-2.84024	-0.22751	1.49963
C	-5.49080	-1.85742	0.11691
H	-5.37384	-3.64117	-1.09214
H	-5.31355	-0.10216	1.36052
C	-0.84841	-1.83777	2.91120
H	-1.76065	-2.42834	2.79040
C	0.58336	1.87391	-1.52323
H	-0.18818	2.53951	-1.92124
H	0.75431	1.06471	-2.24280
H	-6.57591	-1.79785	0.05636
H	-5.67351	1.75723	-2.70449
H	1.49521	2.45941	-1.34701
Au	2.59870	-0.41395	-0.58742
P	4.34641	-1.49425	-1.71703
H	4.53884	-2.85084	-1.41773
H	4.24817	-1.53428	-3.11558
H	5.64396	-0.98833	-1.54932
N	1.33684	-0.35250	2.67424
O	3.30444	3.24465	0.41562
C	2.28152	0.58413	2.60964
C	3.34711	2.40662	1.29062
C	2.15520	1.69768	1.78300
H	1.23330	2.27829	1.78747
H	-1.02706	-1.03942	3.63821
H	-0.03465	-2.48444	3.25364
H	3.22078	0.36123	3.12761
H	4.31889	2.08990	1.73961

Imaginary frequencies: -433.93 cm^{-1}

TS3$_{rot}$, E$_{total}$ = - 2547.062740593153

C	-0.69464	0.43092	1.55675
C	-1.00353	-0.60183	0.53878
N	0.39943	1.26344	1.50439
N	-0.17814	-1.51796	0.01947
S	0.69027	2.10604	0.02592
O	0.44181	1.12803	-1.03034
O	-0.08658	3.33439	0.04253
S	1.30317	-2.17604	0.76488
O	1.75518	-1.16202	1.69706
O	0.97291	-3.52152	1.19978
C	2.39461	-2.30168	-0.63221
C	3.07377	-3.50946	-0.78932
C	2.60433	-1.21305	-1.47714
C	4.00571	-3.61619	-1.81727
H	2.87399	-4.34305	-0.11912
C	3.53396	-1.34325	-2.50271
H	2.03282	-0.29300	-1.36126
C	4.23526	-2.53728	-2.66789
H	4.54976	-4.54827	-1.95422
H	3.70589	-0.50798	-3.17870
C	2.41388	2.49268	0.14159
C	2.80286	3.77481	-0.24031
C	3.32955	1.51422	0.52896
C	4.16136	4.07702	-0.24973
H	2.05547	4.51571	-0.51699
C	4.68065	1.83951	0.51963
H	2.99026	0.53447	0.86578
C	5.09369	3.11276	0.12588
H	4.48942	5.07130	-0.54507
H	5.41314	1.09709	0.82994
C	0.66608	2.05430	2.71753
H	1.63195	2.55477	2.60153
C	-0.64144	-2.31062	-1.12931
H	0.02226	-3.16382	-1.29670
H	-0.64348	-1.68046	-2.02805
H	6.15424	3.35732	0.12145
H	4.96258	-2.62913	-3.47233
H	-1.64813	-2.69638	-0.92429
Au	-2.56836	0.14687	-0.72895
P	-4.17226	1.12901	-2.12733
H	-3.98245	2.49069	-2.40512
H	-4.29600	0.60151	-3.42120
H	-5.50977	1.12063	-1.70421
N	-1.60353	0.69986	2.48857
O	-0.53419	-2.00612	3.45422
C	-2.59237	-0.18397	2.51120
C	-1.35819	-2.36739	2.64826
C	-2.41340	-1.49125	2.05976
H	-3.30514	-2.04237	1.74690
H	-3.57506	0.19752	2.80345
H	-1.40752	-3.42131	2.30103
H	0.72772	1.35336	3.55546
H	-0.12308	2.79232	2.89832

Imaginary frequencies: -412.57 cm^{-1}

Int4, E$_{total}$ = - 2547.116452858524

C	-0.67224	0.07212	1.58183
C	-0.98035	-0.92607	0.57449
N	0.12150	1.15836	1.58473
N	0.04168	-1.36817	-0.33374
S	0.59026	2.08697	0.17467
O	0.20607	1.32353	-1.00068
O	0.02068	3.40472	0.38302
S	1.28491	-2.24175	0.42085
O	1.44187	-1.60429	1.73380
O	1.01228	-3.67726	0.39510
C	2.69988	-1.90987	-0.60251
C	3.78953	-2.76236	-0.42615
C	2.74628	-0.81966	-1.46891
C	4.96055	-2.50124	-1.12935
H	3.71660	-3.61757	0.24421
C	3.92087	-0.58410	-2.17740
H	1.88045	-0.17069	-1.59210
C	5.02465	-1.41575	-2.00251
H	5.82141	-3.15461	-1.00318
H	3.97112	0.25841	-2.86519
C	2.35272	2.15586	0.35015
C	2.98562	3.22142	-0.29055
C	3.05919	1.15611	1.01468
C	4.37511	3.27264	-0.26862
H	2.40351	3.99784	-0.78456
C	4.44797	1.23473	1.03653

H	2.54461	0.33054	1.50695
C	5.10176	2.28281	0.39258
H	4.89022	4.09523	-0.76010
H	5.01753	0.46610	1.55591
C	0.33129	1.88456	2.85721
H	1.10837	2.63708	2.70350
C	-0.39928	-1.93675	-1.61182
H	0.44940	-2.40508	-2.12165
H	-0.76728	-1.11884	-2.24465
H	6.18877	2.33461	0.41191
H	5.94096	-1.22260	-2.55749
H	-1.18496	-2.69386	-1.48377
Au	-2.50933	0.29822	-0.50718
P	-4.08040	1.65148	-1.58965
H	-5.34995	1.10381	-1.82816
H	-4.41962	2.85948	-0.96208
H	-3.73651	2.10478	-2.87222
N	-1.34063	-0.16294	2.78950
O	-3.50141	-2.38235	-0.18765
C	-1.91981	-1.30787	2.69079
C	-2.99038	-2.64181	0.87306
C	-1.71662	-2.00339	1.39312
H	-1.02994	-2.85611	1.58794
H	0.66574	1.16993	3.61363
H	-0.59113	2.37576	3.17892
H	-2.53888	-1.68616	3.50669
H	-3.43480	-3.39752	1.55708

Imaginary frequencies: none

Int4$_{epi}$, E$_{total}$ = - 2547.107125237819

C	0.78626	0.19864	1.51447
C	1.14082	0.98786	0.34577
N	-0.09927	-0.80336	1.71713
N	0.14030	1.40017	-0.59801
S	-0.58365	-1.96545	0.51483
O	-0.19641	-1.42353	-0.78077
O	-0.01909	-3.23416	0.93651
S	-1.17047	2.30767	-0.01400
O	-1.24949	1.96632	1.40914
O	-1.04272	3.71623	-0.38067
C	-2.57947	1.68028	-0.90131
C	-3.75062	2.42527	-0.75115
C	-2.53828	0.52112	-1.66939
C	-4.90985	1.97993	-1.37516
H	-3.75020	3.34191	-0.16276
C	-3.70580	0.09755	-2.29976
H	-1.61784	-0.04993	-1.76432
C	-4.88661	0.81886	-2.14843
H	-5.83127	2.54865	-1.26726
H	-3.68767	-0.80477	-2.90853
C	-2.34337	-1.99306	0.70876
C	-2.98116	-3.16114	0.29140
C	-3.04329	-0.88367	1.17963
C	-4.37020	-3.20747	0.33901
H	-2.40277	-4.01791	-0.05058
C	-4.43082	-0.95933	1.23699
H	-2.52342	0.01754	1.50729
C	-5.09000	-2.11097	0.81111
H	-4.88942	-4.10843	0.01950
H	-4.99654	-0.10958	1.61438
C	-0.32009	-1.27457	3.10298
H	-1.14842	-1.98745	3.09874
C	0.58649	1.74299	-1.94861
H	-0.22606	2.22462	-2.50249
H	0.86430	0.82096	-2.47750
H	-6.17640	-2.15862	0.85671
H	-5.79552	0.48015	-2.64252
H	1.44040	2.43722	-1.93716
Au	2.46831	-0.56075	-0.57367
P	3.92295	-2.15229	-1.49295
H	3.56824	-3.49181	-1.27457
H	4.08559	-2.13328	-2.88618
H	5.25728	-2.14339	-1.06169
N	1.51595	0.55890	2.64292
O	2.66186	4.28937	1.39093
C	2.26066	1.56210	2.32037
C	1.85610	3.54422	0.90039
C	2.13920	2.02030	0.91981
H	3.14501	1.97042	0.46410
H	2.94861	2.01022	3.03838
H	0.92204	3.89755	0.42305
H	-0.58903	-0.41313	3.71971
H	0.57671	-1.75988	3.49758

Imaginary frequencies: none

TS4, E_{total} = - 2547.105750209463

C	0.73833	0.12991	1.60731
C	0.86643	1.13931	0.65108
N	-0.04126	-0.99415	1.68498
N	-0.12315	1.48037	-0.30117
S	-0.45442	-1.99576	0.35605
O	-0.09655	-1.28853	-0.86884
O	0.16450	-3.29288	0.59395
S	-1.43938	2.32475	0.38945
O	-1.59667	1.75535	1.72863
O	-1.23392	3.76635	0.27342
C	-2.80165	1.85044	-0.65020
C	-3.94460	2.64432	-0.55976
C	-2.75719	0.70648	-1.44460
C	-5.07591	2.26770	-1.27565
H	-3.94358	3.54303	0.05551
C	-3.89352	0.35435	-2.16693
H	-1.85115	0.10482	-1.50187
C	-5.04936	1.12627	-2.07662
H	-5.97744	2.87401	-1.21561
H	-3.87293	-0.53258	-2.79816
C	-2.21423	-2.14572	0.51379
C	-2.79054	-3.26050	-0.09587
C	-2.97566	-1.15839	1.13505
C	-4.17608	-3.37753	-0.08629
H	-2.16694	-4.02552	-0.55573
C	-4.35981	-1.30035	1.14307
H	-2.50666	-0.29240	1.60316
C	-4.95642	-2.40004	0.53078
H	-4.64623	-4.24046	-0.55318
H	-4.97071	-0.54007	1.62665
C	-0.18669	-1.65894	2.99732
H	-0.97254	-2.41518	2.91929
C	0.33137	2.03820	-1.57462
H	-0.53095	2.32179	-2.18763
H	0.88884	1.25883	-2.11166
H	-6.04010	-2.50166	0.53971
H	-5.93558	0.84220	-2.64135
H	0.96142	2.93165	-1.44582
Au	2.71673	-0.20192	-0.62516
P	2.95213	-2.33631	-1.49066
H	4.22361	-2.84925	-1.78914
H	2.39843	-3.34915	-0.69148
H	2.27195	-2.53302	-2.70125
N	1.52861	0.37518	2.75084
O	3.53093	1.97844	-0.20808
C	2.06178	1.53253	2.61460
C	2.96239	2.65383	0.63174
C	1.70023	2.21563	1.32238
H	1.08081	3.10683	1.54258
H	-0.49042	-0.90849	3.73216
H	0.74763	-2.13688	3.30547
H	2.75527	1.92961	3.35623
H	3.40567	3.61953	0.93694

Imaginary frequencies: -64.97 cm^{-1}

Int5, E_{total} = - 2547.114633863254

C	-0.62007	-0.47360	1.52203
C	-0.38602	-1.52773	0.69388
N	0.04803	0.73859	1.65119
N	0.66739	-1.74349	-0.19370
S	0.16964	1.78374	0.33332
O	-0.03414	0.98550	-0.87410
O	-0.72722	2.92463	0.53404
S	2.13035	-2.25177	0.53745
O	2.16868	-1.58318	1.83363
O	2.23012	-3.70606	0.48460
C	3.34251	-1.54762	-0.55808
C	4.62311	-2.09538	-0.49022
C	3.04965	-0.45286	-1.36964
C	5.63669	-1.51801	-1.24771
H	4.81826	-2.96154	0.14049
C	4.07228	0.10046	-2.13443
H	2.04108	-0.04167	-1.40827
C	5.36092	-0.42411	-2.06753
H	6.64252	-1.93093	-1.20506
H	3.85757	0.95114	-2.77939
C	1.84733	2.35150	0.44347
C	2.13862	3.55978	-0.18974
C	2.83305	1.57342	1.04808
C	3.45970	3.99272	-0.22011
H	1.34449	4.15406	-0.63860
C	4.14753	2.02913	1.01625
H	2.58710	0.62848	1.53415
C	4.45980	3.22871	0.38110
H	3.70680	4.93403	-0.70665
H	4.92930	1.43335	1.48475

C	0.04963	1.37612	2.97685
H	0.72928	2.23377	2.95785
C	0.34198	-2.40610	-1.45115
H	1.25134	-2.56614	-2.03944
H	-0.32976	-1.75596	-2.02505
H	5.49104	3.57654	0.35868
H	6.15682	0.01747	-2.66466
H	-0.12300	-3.39594	-1.29273
Au	-3.04525	0.05694	-0.63694
P	-3.41084	2.32394	-0.72086
H	-4.64789	2.69241	-1.26992
H	-3.40322	3.00036	0.50349
H	-2.49780	3.04807	-1.49456
N	-1.68212	-0.72534	2.42640
O	-2.98886	-2.13707	-0.69330
C	-2.12346	-1.90240	2.18913
C	-2.36178	-2.95615	-0.03012
C	-1.39199	-2.59851	1.05846
H	-0.87711	-3.50839	1.41056
H	-2.97373	-2.32525	2.72257
H	0.42957	0.64633	3.69928
H	-0.95230	1.70928	3.26765
H	-2.54144	-4.02377	-0.24595

Imaginary frequencies: none

Int6, E_{total} = - 2069.144075078438

C	2.08652	0.98318	-0.54416
C	2.15525	-0.28759	-0.07278
N	1.02157	1.87865	-0.62885
N	1.13316	-1.09823	0.44665
S	0.05425	2.28001	0.69540
O	0.32121	1.31682	1.75431
O	0.18918	3.70412	0.96902
S	0.32524	-2.05433	-0.69566
O	0.27548	-1.26752	-1.92483
O	0.89804	-3.39882	-0.74776
C	-1.31123	-2.17659	0.00402
C	-2.13741	-3.15863	-0.53981
C	-1.76740	-1.29203	0.97806
C	-3.45500	-3.24507	-0.10445
H	-1.74894	-3.84563	-1.29037
C	-3.08384	-1.40389	1.41680
H	-1.10679	-0.52785	1.38728
C	-3.92605	-2.36980	0.87349
H	-4.11292	-4.00403	-0.52353
H	-3.45127	-0.72056	2.18066
C	-1.58711	2.01796	0.04327
C	-2.63246	2.66291	0.70269
C	-1.81784	1.14723	-1.01723
C	-3.93810	2.41807	0.29096
H	-2.42139	3.35269	1.51846
C	-3.12969	0.91770	-1.42284
H	-0.98923	0.64266	-1.51448
C	-4.18557	1.54643	-0.76926
H	-4.76473	2.91528	0.79515
H	-3.32018	0.23144	-2.24670
C	1.06833	2.89476	-1.68785
H	0.07606	3.34587	-1.79016
C	1.43052	-1.76983	1.71130
H	0.54442	-2.30150	2.07481
H	1.70225	-0.99769	2.43935
H	-5.20965	1.35944	-1.08829
H	-4.95658	-2.44575	1.21647
H	2.24551	-2.50483	1.61009
N	3.30805	1.41306	-1.11542
O	4.80296	-0.38720	1.65986
C	4.13032	0.43528	-1.01618
C	4.36873	-1.18552	0.86756
C	3.55940	-0.78295	-0.35869
H	3.51062	-1.63567	-1.05946
H	1.80259	3.67694	-1.46835
H	1.32491	2.39716	-2.62766
H	5.16675	0.52578	-1.34015
H	4.53817	-2.27803	1.00004

Imaginary frequencies: none

Product (2), E_{total} = - 2069.189018041992

C	-1.20865	3.17626	0.66009
C	0.04246	3.05558	0.07138
C	0.34847	1.65344	0.03774
C	-0.72207	0.99290	0.59482
C	0.80072	4.21258	-0.35448
O	1.89890	4.20189	-0.88882
N	-0.89524	-0.37472	0.81813
N	1.49677	1.00850	-0.45477

S	-1.58849	-1.28015	-0.43661
O	-1.39110	-2.67672	-0.06443
O	-1.03334	-0.75135	-1.67451
C	-3.32872	-0.92210	-0.39582
C	-4.15676	-1.68052	0.43062
C	-3.81763	0.14382	-1.15045
C	-5.50876	-1.35841	0.49960
H	-3.75127	-2.51803	0.99535
C	-5.17133	0.45372	-1.06964
H	-3.14925	0.71109	-1.79592
C	-6.01230	-0.29425	-0.24644
H	-6.17058	-1.94504	1.13324
H	-5.57138	1.27724	-1.65749
S	2.73327	0.63174	0.60995
O	2.14424	0.65401	1.94760
O	3.92280	1.44056	0.34186
C	3.12523	-1.04735	0.16509
C	2.18174	-2.03998	0.42423
C	4.35283	-1.33623	-0.42140
C	2.48066	-3.35364	0.08569
H	1.22585	-1.78093	0.87791
C	4.64082	-2.65803	-0.75552
H	5.06719	-0.53751	-0.60994
C	3.70817	-3.66087	-0.50314
H	1.75292	-4.13934	0.27919
H	5.59635	-2.90119	-1.21579
C	1.85624	1.17195	-1.86359
H	0.95369	0.99171	-2.45646
H	2.59806	0.41313	-2.14072
N	-1.65846	1.94186	0.97313
H	-2.55176	1.74271	1.41036
H	3.93679	-4.69146	-0.76858
H	-7.07112	-0.04888	-0.19084
H	2.25597	2.17360	-2.06586
C	-1.15559	-0.81250	2.19500
H	-1.03890	-1.89801	2.25688
H	-2.16127	-0.53713	2.54787
H	-0.40288	-0.33887	2.83363
H	-1.79049	4.06603	0.87186
H	0.28896	5.18050	-0.14975

Imaginary frequencies: none

Product (4), E_{total} = - 2069.184558711796

C	2.13018	0.41945	-0.13955
C	1.99596	-0.88458	0.34510
N	1.16849	1.35992	-0.50797
N	0.78538	-1.52467	0.69027
S	0.28546	2.14374	0.70871
O	0.28283	1.25047	1.85973
O	0.75762	3.51488	0.86430
S	-0.08227	-2.22332	-0.57361
O	0.13217	-1.37073	-1.74056
O	0.18735	-3.65423	-0.68525
C	-1.76271	-2.03069	0.00167
C	-2.72098	-2.83467	-0.61305
C	-2.11266	-1.07437	0.95215
C	-4.05922	-2.66704	-0.27389
H	-2.41664	-3.58553	-1.34076
C	-3.45437	-0.93027	1.29411
H	-1.34912	-0.45153	1.41897
C	-4.42439	-1.71754	0.67954
H	-4.81784	-3.28681	-0.74824
H	-3.73866	-0.18887	2.03943
C	-1.34064	2.21071	-0.02201
C	-2.21768	3.15470	0.51025
C	-1.73680	1.31985	-1.01621
C	-3.52531	3.19612	0.03932
H	-1.87483	3.85012	1.27482
C	-3.04474	1.38552	-1.48835
H	-1.03859	0.58327	-1.41534
C	-3.93661	2.31430	-0.95949
H	-4.22208	3.92631	0.44649
H	-3.36544	0.69480	-2.26676
C	1.41977	2.14384	-1.71778
H	0.51158	2.68968	-1.99353
C	0.80942	-2.29436	1.93392
H	-0.18971	-2.68915	2.14715
H	1.09340	-1.60675	2.73865
H	-4.95968	2.35547	-1.32959
H	-5.47251	-1.59489	0.94763
H	1.50895	-3.14127	1.89026
N	3.45796	0.66022	-0.30818
O	6.26866	0.49482	-0.47383
C	4.18640	-0.44794	0.05484
C	5.62261	-0.45240	-0.05327
C	3.29198	-1.42092	0.46906
H	3.55345	-2.41853	0.80678
H	2.22626	2.87991	-1.58882
H	1.66108	1.44287	-2.52484

H	3.88948	1.51785	-0.63462	H	6.11679	-1.39173	0.27858

Imaginary frequencies: none

Unsymmetrical yndiamide (PGMeYndPGiPr), E_{total} = - 1901.886251825621

C	0.54817	1.55082	-0.38214	C	4.23540	-2.34524	1.18815
C	-0.61540	1.75943	-0.09203	H	4.64667	-1.29339	-0.66119
N	1.80326	1.29909	-0.76805	C	1.92060	-2.35979	1.89886
N	-1.86539	1.88921	0.37641	H	0.52590	-1.32691	0.62201
S	2.10963	-0.22003	-1.49633	C	3.25357	-2.71813	2.10359
O	0.83636	-0.66825	-2.04421	H	5.27275	-2.62992	1.35099
O	3.27350	-0.04715	-2.35655	H	1.15624	-2.65006	2.61688
S	-2.46249	0.59565	1.34102	C	2.94885	2.00890	-0.11149
O	-1.29349	-0.03583	1.93702	C	2.94942	3.45146	-0.57834
O	-3.51771	1.15348	2.17723	C	2.87958	1.88276	1.39916
C	-3.19650	-0.50004	0.15191	H	3.85082	1.50913	-0.49007
C	-4.57968	-0.65163	0.12590	H	2.02626	3.95788	-0.26340
C	-2.36273	-1.13973	-0.76582	H	3.02593	3.51236	-1.67032
C	-5.13964	-1.49002	-0.83469	H	1.96847	2.35924	1.78635
H	-5.20054	-0.12567	0.84800	H	2.88529	0.83404	1.72454
C	-2.93844	-1.96591	-1.72342	C	-2.86765	2.60005	-0.43416
H	-1.28348	-0.99033	-0.74878	H	-3.78148	2.72439	0.15381
C	-4.32199	-2.14312	-1.75417	H	-3.08643	2.05521	-1.36256
H	-6.21825	-1.62866	-0.86529	H	3.52834	-3.29269	2.98608
H	-2.30261	-2.47072	-2.44794	H	-4.76588	-2.79359	-2.50533
C	2.55880	-1.25613	-0.12767	H	3.79725	3.98892	-0.13606
C	3.89366	-1.60193	0.06116	H	3.74272	2.38631	1.85175
C	1.56208	-1.62493	0.77559	H	-2.46511	3.58765	-0.67348

Imaginary frequencies: none

3-methyl-5-phenyl-isoxazole, E_{total} = - 515.522300078345

C	0.57205	-0.05351	-0.00001	C	-2.90979	1.33426	0.00012
O	1.14172	-1.27518	0.00020	H	-0.93131	2.17198	0.00025
C	2.76059	0.15332	-0.00001	C	-3.67568	0.16874	-0.00001
C	1.55199	0.89651	-0.00013	H	-3.63814	-1.98706	-0.00025
N	2.52657	-1.14166	0.00020	H	-3.39587	2.30820	0.00024
H	1.44496	1.97404	-0.00044	H	-4.76253	0.23001	-0.00001
C	-0.88333	0.01097	-0.00001	C	4.15579	0.66531	-0.00013
C	-1.65720	-1.15635	-0.00016	H	4.86950	-0.16507	-0.00005
C	-1.52284	1.25740	0.00013	H	4.34240	1.28688	-0.88417
C	-3.04540	-1.07405	-0.00015	H	4.34242	1.28708	0.88377
H	-1.16824	-2.12847	-0.00028				

Imaginary frequencies: none

$Int1_{major}$, E_{total} = - 2379.877894249095

C	0.01335	-0.62083	0.73190	N	-0.75251	-1.60626	0.48336
C	0.92752	0.30008	0.74551	N	0.89332	1.58909	1.18175

S	-1.48457	-1.74158	-1.15374
O	-1.02034	-0.55145	-1.84724
O	-1.13845	-3.07641	-1.61703
S	-0.62530	2.33183	1.47680
O	-1.57901	1.22620	1.44386
O	-0.48414	3.15912	2.66542
C	-0.85498	3.37942	0.06685
C	-0.83098	4.76116	0.23424
C	-1.02395	2.77976	-1.18211
C	-0.99980	5.56741	-0.88878
H	-0.68907	5.19143	1.22341
C	-1.18799	3.60061	-2.29152
H	-1.02172	1.69434	-1.29305
C	-1.17811	4.98856	-2.14346
H	-0.99069	6.64985	-0.78053
H	-1.32374	3.15524	-3.27480
C	-3.20636	-1.63962	-0.79279
C	-3.96457	-2.80926	-0.78128
C	-3.74316	-0.39023	-0.48077
C	-5.31072	-2.71664	-0.44275
H	-3.51140	-3.76437	-1.03922
C	-5.08885	-0.32005	-0.14265
H	-3.12012	0.50236	-0.48870
C	-5.86621	-1.47887	-0.12198
H	-5.92575	-3.61365	-0.43119
H	-5.53198	0.64182	0.10535
C	-1.04909	-2.70640	1.47085
C	0.25232	-3.37215	1.86350
C	-1.81892	-2.12190	2.63673
H	-1.67498	-3.42247	0.92195
H	0.90846	-2.66397	2.38855
H	0.78108	-3.75758	0.98220
H	-1.21883	-1.35691	3.14847
H	-2.75932	-1.66016	2.30795
C	2.05258	2.47629	1.04975
H	1.85767	3.40272	1.59805
H	2.25171	2.70566	-0.00529
H	-6.91908	-1.41505	0.14573
H	-1.30843	5.62412	-3.01728
H	0.04735	-4.20994	2.54038
H	-2.05323	-2.91485	3.35697
H	2.92354	1.99025	1.49965
Au	2.65275	-0.60698	-0.10406
P	4.55523	-1.53091	-1.11240
H	4.44181	-1.86900	-2.46734
H	5.69949	-0.72208	-1.12016
H	5.05689	-2.72009	-0.56703

Imaginary frequencies: none

Int1$_{minor}$, E$_{total}$ = - 2379.869854338089

C	-0.54506	-0.61107	0.52847
C	-0.33464	0.51285	1.12313
N	0.18448	-1.75543	0.42253
N	-0.24910	1.69843	1.59174
S	1.54422	-1.96384	1.45248
O	1.40136	-0.95803	2.49834
O	1.57473	-3.38471	1.77137
S	0.02203	3.05301	0.43361
O	-1.18495	3.07186	-0.37884
O	0.37696	4.17897	1.28006
C	1.39191	2.53573	-0.54696
C	2.63476	3.11358	-0.28891
C	1.18140	1.64422	-1.60176
C	3.69494	2.79578	-1.12975
H	2.75725	3.81395	0.53444
C	2.26278	1.31807	-2.41246
H	0.19209	1.23367	-1.79859
C	3.50891	1.89996	-2.18152
H	4.66922	3.25052	-0.96230
H	2.12617	0.62408	-3.23964
C	2.98247	-1.57062	0.49359
C	3.44836	-2.49545	-0.44194
C	3.65419	-0.37842	0.75375
C	4.61117	-2.20345	-1.14524
H	2.93087	-3.43978	-0.60135
C	4.83349	-0.11743	0.06195
H	3.27395	0.31675	1.50069
C	5.30574	-1.02148	-0.88636
H	4.98937	-2.91275	-1.87836
H	5.38642	0.79651	0.26961
C	-0.18392	-2.86913	-0.50195
C	-1.44325	-3.57719	-0.04049
C	-0.24642	-2.36964	-1.93358
H	0.64544	-3.58142	-0.41849
H	-2.32084	-2.91339	-0.08193
H	-1.33311	-3.94277	0.98735
H	-1.07797	-1.66355	-2.08176
H	0.68710	-1.86583	-2.21783

C	-0.70798	2.09720	2.93976	H	-0.80080	1.18205	3.52875
H	-1.67611	2.60484	2.86708	Au	-2.53974	-0.29977	-0.20449
H	0.03203	2.76327	3.38865	P	-4.71480	0.07817	-0.99060
H	6.22818	-0.80858	-1.42376	H	-5.36701	-1.01697	-1.57195
H	4.34644	1.65212	-2.83154	H	-5.65839	0.49674	-0.04327
H	-1.64739	-4.43285	-0.69620	H	-4.86097	1.05407	-1.98526
H	-0.40569	-3.21534	-2.61383				

Imaginary frequencies: none

TS$_{interconv.}$, E$_{total}$ = - 2379.867395613075

C	-0.40566	-0.17148	1.01186	H	-2.19892	1.74083	-0.86730
C	0.64585	0.22512	1.62667	C	-5.54857	1.80821	-1.47606
N	-1.64067	-0.58155	0.90837	H	-6.85187	0.09113	-1.53994
N	1.35718	1.33011	1.86529	H	-4.03338	3.34156	-1.34691
S	-2.22264	-1.13272	-0.65438	C	-2.56448	-0.66344	2.09218
O	-1.10183	-0.90529	-1.55589	C	-2.14095	-1.82525	2.96767
O	-2.77276	-2.45850	-0.44848	C	-2.58550	0.67100	2.80915
S	1.00845	2.71226	0.83988	H	-3.55378	-0.86540	1.65581
O	-0.42596	2.65691	0.61352	H	-1.12587	-1.66466	3.35809
O	1.67203	3.83184	1.47581	H	-2.16237	-2.76961	2.41088
C	1.85792	2.26038	-0.64912	H	-1.60863	0.89555	3.25963
C	3.21509	2.56223	-0.76611	H	-2.84886	1.49157	2.13056
C	1.17669	1.52839	-1.62190	C	2.70028	1.25359	2.45119
C	3.90338	2.10773	-1.88645	H	2.95679	2.22365	2.88364
H	3.71290	3.16013	-0.00422	H	3.44762	0.96831	1.69615
C	1.88089	1.08107	-2.73643	H	-6.34982	2.51569	-1.68090
H	0.11488	1.31761	-1.52267	H	3.78388	1.02462	-3.74314
C	3.23918	1.36633	-2.86449	H	-2.81718	-1.91343	3.82661
H	4.95877	2.34524	-2.00298	H	-3.32270	0.63845	3.62025
H	1.35582	0.51885	-3.50740	H	2.67742	0.49979	3.24246
C	-3.52350	0.02384	-0.95841	Au	1.38371	-1.41162	0.19241
C	-4.81628	-0.46427	-1.13055	P	2.69699	-2.89259	-1.05087
C	-3.21573	1.38270	-1.03154	H	3.60013	-3.71984	-0.36804
C	-5.83397	0.44724	-1.39706	H	1.99085	-3.82562	-1.82419
H	-5.01277	-1.53242	-1.05964	H	3.53084	-2.30584	-2.01482
C	-4.24667	2.27631	-1.29110				

Imaginary frequencies: -71.29 cm^{-1}

TS1$_{major}$, E$_{total}$ = - 2895.396756350550

C	-0.43179	-0.74566	-0.47731	S	1.66503	0.69007	1.91183
C	0.35704	0.29279	-0.36207	O	0.69323	-0.18581	2.54263
N	-1.61364	-1.22897	-0.71870	O	3.07329	0.66150	2.27229
N	1.62888	0.32311	0.24133	C	1.06751	2.36124	2.01413
S	-2.98332	-0.53140	0.18169	C	1.97048	3.41317	1.86892
O	-2.39543	0.02081	1.38612	C	-0.30293	2.57754	2.16089
O	-3.66960	0.30379	-0.79061	C	1.48410	4.71725	1.86664

H	3.03677	3.20987	1.78231
C	-0.77192	3.88976	2.16479
H	-0.98267	1.73493	2.28247
C	0.11732	4.95334	2.01512
H	2.17608	5.55130	1.76719
H	-1.83590	4.07832	2.30215
C	-3.97726	-1.93719	0.56757
C	-5.03245	-2.27727	-0.27692
C	-3.66593	-2.67460	1.70919
C	-5.77748	-3.41321	0.02219
H	-5.26984	-1.65627	-1.13885
C	-4.42022	-3.80684	1.99149
H	-2.86092	-2.35446	2.36780
C	-5.46477	-4.17835	1.14467
H	-6.60771	-3.69779	-0.62059
H	-4.19972	-4.39538	2.87955
C	-1.92772	-2.05650	-1.93733
C	-1.02101	-1.64848	-3.08022
C	-1.85020	-3.53830	-1.61347
H	-2.96041	-1.78113	-2.19654
H	0.02797	-1.88840	-2.86099
H	-1.09917	-0.57389	-3.29007
H	-0.82388	-3.82627	-1.35691
H	-2.50914	-3.81157	-0.77887
C	2.66093	0.97537	-0.57014
H	3.62620	0.87397	-0.06534
H	2.43651	2.04021	-0.75411
H	-6.05066	-5.06728	1.37044
H	-0.25468	5.97687	2.03139
H	-1.31237	-2.19680	-3.98408
H	-2.16351	-4.11660	-2.49216
H	2.70789	0.44951	-1.53029
Au	-0.55667	2.05342	-1.10640
P	-1.58779	4.06019	-1.78666
H	-2.81280	4.36017	-1.16825
H	-0.87908	5.24750	-1.53768
H	-1.92846	4.24059	-3.13694
N	0.81465	-2.43817	-0.06374
O	2.01409	-2.27316	-0.73129
C	1.11205	-2.84243	1.16222
C	3.02182	-2.57762	0.10483
C	2.50675	-2.96126	1.31505
C	4.37759	-2.42425	-0.39427
H	3.04419	-3.27841	2.19980
C	4.64034	-2.38071	-1.76956
C	5.43274	-2.29903	0.51843
C	5.94493	-2.22508	-2.22151
H	3.82125	-2.48270	-2.47979
C	6.73430	-2.14251	0.05976
H	5.22686	-2.29693	1.58839
C	6.99274	-2.10727	-1.30963
H	6.14641	-2.19961	-3.29084
H	7.54962	-2.03960	0.77328
H	8.01365	-1.98535	-1.66727
C	0.02914	-3.11756	2.14063
H	-0.63683	-2.24870	2.20868
H	-0.55429	-3.99784	1.83862
H	0.44483	-3.29677	3.13701

Imaginary frequencies: -185.49 cm^{-1}

TS1$_{minor}$, E$_{total}$ = - 2895.394604875156

C	-0.01750	0.53284	-0.86454
C	0.09288	-0.66623	-0.30837
N	0.60803	1.40633	-1.61018
N	-0.49071	-1.02224	0.89281
S	2.28998	1.87593	-1.25741
O	2.79632	0.85969	-0.35434
O	2.85620	2.08400	-2.57913
S	-0.28634	-0.03140	2.26869
O	0.33737	1.20540	1.81601
O	-1.55200	-0.00589	2.98772
C	0.91593	-0.92956	3.21440
C	0.52042	-1.65227	4.33468
C	2.23536	-0.91549	2.76101
C	1.48129	-2.39305	5.01931
H	-0.51753	-1.62495	4.66279
C	3.18092	-1.65935	3.45651
H	2.50976	-0.33457	1.87859
C	2.80266	-2.39899	4.57844
H	1.19585	-2.96419	5.90051
H	4.21745	-1.66053	3.12483
C	2.16752	3.42754	-0.41687
C	2.05871	4.58909	-1.18330
C	2.19550	3.45240	0.97652
C	1.95724	5.80992	-0.52593
H	2.08015	4.53631	-2.27051
C	2.10748	4.68525	1.61492
H	2.26509	2.52699	1.54407
C	1.98288	5.85554	0.86804

H	1.87509	6.72844	-1.10332
H	2.13513	4.72914	2.70193
C	-1.02591	-2.38746	1.15586
H	-1.40935	-2.33634	2.18431
H	1.91360	6.81553	1.37664
H	3.54847	-2.98105	5.11688
Au	1.40099	-1.87267	-1.44645
P	2.91883	-3.10542	-2.75333
H	3.42702	-4.30201	-2.22202
H	2.49281	-3.55128	-4.01506
H	4.11314	-2.45503	-3.10283
N	-1.69049	1.41722	-0.19071
O	-2.71629	0.58785	-0.58130
C	-2.22480	2.43031	0.46871
C	-3.87893	1.10053	-0.13581
C	-3.62612	2.26776	0.53190
C	-5.09440	0.36570	-0.44506
H	-4.33585	2.94105	0.99592
C	-5.08460	-0.68288	-1.37400
C	-6.29231	0.71628	0.19005
C	-6.25905	-1.36785	-1.65973
H	-4.15695	-0.95331	-1.87599
C	-7.46280	0.02817	-0.10031
H	-6.30404	1.52100	0.92423
C	-7.44845	-1.01476	-1.02513
H	-6.24725	-2.17964	-2.38463
H	-8.38930	0.30328	0.39992
H	-8.36680	-1.55356	-1.25134
C	-0.07645	2.15246	-2.67212
H	-0.47024	3.10152	-2.28380
H	0.62323	2.34083	-3.49083
H	-0.90613	1.53955	-3.03503
C	-2.20689	-2.64728	0.23847
H	-2.62803	-3.64123	0.43647
H	-2.99142	-1.89639	0.39376
H	-1.89836	-2.61248	-0.81681
C	0.01783	-3.49146	1.09338
H	0.31788	-3.71144	0.05798
H	0.91690	-3.24802	1.67489
H	-0.41185	-4.41465	1.50355
C	-1.40351	3.53354	1.02288
H	-1.94552	4.48348	0.95553
H	-1.17188	3.33797	2.07783
H	-0.44962	3.62331	0.49181

Imaginary frequencies: -245.25 cm^{-1}

Int2$_{major}$, E$_{total}$ = - 2895.438127629667

C	-0.56666	0.49476	-0.07563
C	0.08214	-0.67911	-0.22590
N	-0.11473	1.76690	-0.45428
N	-0.59164	-1.83436	0.31686
S	1.34312	2.37122	0.15150
O	1.59969	1.66376	1.40189
O	2.34887	2.36563	-0.91020
S	-0.15353	-2.27952	1.88754
O	-0.34156	-1.09229	2.71378
O	-0.89912	-3.50041	2.17898
C	1.57919	-2.66433	1.83375
C	1.97266	-3.96093	1.50377
C	2.50513	-1.63834	2.02486
C	3.33101	-4.23469	1.37142
H	1.22804	-4.74317	1.36644
C	3.86085	-1.93064	1.89440
H	2.17087	-0.63083	2.26765
C	4.27071	-3.22234	1.56731
H	3.65592	-5.24342	1.12411
H	4.59699	-1.14331	2.04736
C	0.97201	4.07395	0.51212
C	1.23867	5.05146	-0.44476
C	0.42797	4.39501	1.75429
C	0.91445	6.37482	-0.16030
H	1.70537	4.78364	-1.39072
C	0.11248	5.72167	2.02730
H	0.27609	3.62129	2.50338
C	0.34523	6.70677	1.06772
H	1.11528	7.14846	-0.89849
H	-0.30712	5.98741	2.99544
C	-0.67738	2.42885	-1.67189
C	-0.74982	1.46238	-2.83946
C	-2.00714	3.09916	-1.36054
H	0.04462	3.21218	-1.93718
H	-1.45685	0.64269	-2.64903
H	0.23463	1.02421	-3.05047
H	-2.80493	2.36641	-1.17707
H	-1.92418	3.75201	-0.48031
C	-0.71560	-2.93215	-0.64651
H	-1.29012	-3.74610	-0.19697
H	0.26683	-3.29961	-0.98796
H	0.09465	7.74338	1.28512

H	5.33206	-3.44398	1.46763
H	-1.09187	1.99732	-3.73414
H	-2.32101	3.71273	-2.21465
H	-1.26585	-2.54634	-1.51300
Au	1.94091	-0.88940	-1.15442
P	4.08111	-1.16800	-2.13080
H	5.14181	-0.50587	-1.49183
H	4.59542	-2.47385	-2.18561
H	4.28300	-0.75661	-3.45739
N	-1.91225	0.48642	0.44322
O	-2.83014	-0.18119	-0.33007
C	-2.48473	0.88452	1.57876
C	-3.98781	-0.19125	0.35452
C	-3.82196	0.47335	1.54823
C	-5.11145	-0.85755	-0.26887
H	-4.55464	0.63587	2.32788
C	-4.96150	-1.52429	-1.49367
C	-6.36058	-0.83155	0.36667
C	-6.05408	-2.15658	-2.07149
H	-3.99257	-1.54850	-1.98933
C	-7.44735	-1.46681	-0.21822
H	-6.48227	-0.31313	1.31623
C	-7.29572	-2.12899	-1.43653
H	-5.93747	-2.67471	-3.02104
H	-8.41596	-1.44507	0.27653
H	-8.14932	-2.62637	-1.89322
C	-1.75178	1.64144	2.61129
H	-0.67235	1.47497	2.53493
H	-1.97014	2.71225	2.49546
H	-2.09170	1.33333	3.60498

Imaginary frequencies: none

Int2$_{minor}$, E$_{total}$ = - 2895.427630006539

C	-0.49516	0.58127	-0.26690
C	0.16580	-0.60484	-0.26955
N	-0.08652	1.71355	-0.98214
N	-0.27377	-1.58383	0.66231
S	1.32106	2.57404	-0.59626
O	2.07633	1.76139	0.34647
O	1.90058	2.96928	-1.87734
S	0.00468	-1.30772	2.31210
O	0.01638	0.13591	2.51873
O	-0.94344	-2.13773	3.05097
C	1.64407	-1.91973	2.60817
C	1.81811	-3.14956	3.23647
C	2.71836	-1.16826	2.13045
C	3.11159	-3.64393	3.38555
H	0.95553	-3.70606	3.59847
C	4.00283	-1.68002	2.28221
H	2.55026	-0.20549	1.64625
C	4.19741	-2.91431	2.90442
H	3.26967	-4.60154	3.87766
H	4.85594	-1.11104	1.91732
C	0.82633	4.05894	0.24778
C	0.20521	5.08105	-0.47026
C	1.08789	4.17297	1.61124
C	-0.18911	6.23021	0.20748
H	0.04461	4.99207	-1.54321
C	0.69859	5.33389	2.27342
H	1.58621	3.36273	2.14046
C	0.05504	6.35432	1.57561
H	-0.67674	7.03600	-0.33728
H	0.90023	5.43969	3.33749
C	-0.36974	-3.02318	0.26579
H	-0.66107	-3.53909	1.18983
H	-0.25026	7.25872	2.09859
H	5.20595	-3.30752	3.01950
Au	1.78915	-0.83222	-1.57234
P	3.60242	-0.97096	-3.08454
H	4.80049	-1.53649	-2.62067
H	3.42350	-1.69632	-4.27282
H	4.07763	0.24606	-3.59778
N	-1.82117	0.68675	0.28794
O	-2.73604	-0.14632	-0.32677
C	-2.47045	1.50578	1.12144
C	-3.94462	0.14311	0.18170
C	-3.82794	1.17314	1.08577
C	-5.06757	-0.63623	-0.29609
H	-4.61318	1.65133	1.65667
C	-4.93784	-1.47772	-1.41063
C	-6.29936	-0.53486	0.36582
C	-6.03138	-2.21132	-1.85078
H	-3.98671	-1.54745	-1.93582
C	-7.38718	-1.27225	-0.08116
H	-6.40215	0.10889	1.23789
C	-7.25453	-2.11076	-1.18791
H	-5.93086	-2.86235	-2.71666
H	-8.34061	-1.19649	0.43724
H	-8.10892	-2.68825	-1.53588

C	-0.94374	2.19247	-2.06789
H	-1.82560	2.72994	-1.68751
H	-0.37381	2.85284	-2.72673
H	-1.26870	1.32512	-2.65598
C	-1.50605	-3.17714	-0.72986
H	-1.61434	-4.23302	-1.00946
H	-2.45484	-2.82963	-0.30243
H	-1.30417	-2.60251	-1.64608
C	0.91385	-3.66121	-0.25148
H	1.12427	-3.37785	-1.29207
H	1.79056	-3.41213	0.35985
H	0.78873	-4.75209	-0.23139
C	-1.80053	2.57519	1.88566
H	-2.39454	2.82870	2.76871
H	-0.79990	2.26528	2.19873
H	-1.71743	3.47890	1.26460

Imaginary frequencies: none

TS2$_{major}$, E_{total} = - 2895.403347606784

C	-0.41160	0.46037	-0.09470
C	0.25067	-0.76469	-0.32282
N	0.02169	1.60932	-0.80524
N	0.13741	-1.79291	0.55267
S	1.55131	2.22615	-0.41748
O	2.28744	1.14776	0.23524
O	2.05964	2.84210	-1.63675
S	0.18573	-1.60627	2.29744
O	0.25213	-0.18320	2.57905
O	-0.86586	-2.44200	2.84798
C	1.77443	-2.31500	2.63680
C	1.85020	-3.52107	3.32498
C	2.90385	-1.62447	2.19679
C	3.11055	-4.05061	3.59057
H	0.94040	-4.02370	3.64931
C	4.15200	-2.17330	2.46551
H	2.80513	-0.68108	1.65585
C	4.25300	-3.37979	3.16040
H	3.19797	-4.98934	4.13371
H	5.05017	-1.65505	2.13563
C	1.31035	3.49973	0.79759
C	0.90283	4.76583	0.37927
C	1.52575	3.20087	2.14053
C	0.68302	5.74816	1.33882
H	0.78150	4.98181	-0.68154
C	1.31432	4.19871	3.08842
H	1.84937	2.20188	2.42993
C	0.88769	5.46362	2.68962
H	0.36757	6.74324	1.03116
H	1.48576	3.98573	4.14207
C	-0.77393	2.26542	-1.88015
C	-1.12454	1.26312	-2.96561
C	-2.00581	2.98682	-1.35260
H	-0.09324	3.00932	-2.31279
H	-1.73609	0.43941	-2.57265
H	-0.21605	0.83794	-3.41447
H	-2.77767	2.28807	-1.00500
H	-1.75582	3.67306	-0.53212
C	0.44433	-3.16122	0.11611
H	0.02475	-3.87128	0.83626
H	1.52841	-3.32057	0.02656
H	0.72346	6.23946	3.43536
H	5.23517	-3.80040	3.36851
H	-1.69847	1.75845	-3.75925
H	-2.45280	3.58238	-2.15913
H	-0.03972	-3.33067	-0.84884
Au	1.50248	-0.92086	-1.99617
P	3.01186	-0.90709	-3.81759
H	2.55756	-1.29540	-5.08894
H	3.56799	0.34760	-4.11724
H	4.17675	-1.68560	-3.72027
N	-1.56960	0.45439	0.57682
O	-2.79254	-0.34894	-0.27648
C	-2.30882	1.20272	1.40629
C	-3.88057	-0.02414	0.34564
C	-3.66802	0.95778	1.31788
C	-5.12466	-0.70710	0.00766
H	-4.40840	1.40207	1.97092
C	-5.12724	-1.71561	-0.96562
C	-6.32174	-0.35107	0.64334
C	-6.31167	-2.36109	-1.29169
H	-4.19580	-1.98747	-1.45830
C	-7.50383	-0.99760	0.31052
H	-6.33319	0.43255	1.39894
C	-7.49960	-2.00313	-0.65534
H	-6.31111	-3.14558	-2.04571
H	-8.43138	-0.71866	0.80620
H	-8.42774	-2.50970	-0.91397
C	-1.67010	2.17385	2.33097
H	-2.41442	2.55859	3.03455

H	-0.84668	1.70667	2.87961
H	-1.25794	3.01821	1.76051

Imaginary frequencies: -508.58 cm^{-1}

TS2$_{minor}$, E$_{total}$ = - 2895.403294713927

C	-0.37934	0.62063	-0.15465
C	0.17912	-0.66928	-0.19029
N	-0.00370	1.54665	-1.15668
N	0.14693	-1.44629	0.91894
S	1.55309	2.22165	-1.09794
O	2.38759	1.24179	-0.41374
O	1.83657	2.63715	-2.46471
S	0.38499	-0.81472	2.53216
O	0.58854	0.62099	2.42225
O	-0.66006	-1.36580	3.37885
C	1.94919	-1.55460	2.91718
C	2.01436	-2.58942	3.84360
C	3.07419	-1.06846	2.25069
C	3.25666	-3.16165	4.10745
H	1.11164	-2.93000	4.34850
C	4.30453	-1.65127	2.52760
H	2.98082	-0.25960	1.52354
C	4.39281	-2.69599	3.44959
H	3.33534	-3.97134	4.83007
H	5.19788	-1.29032	2.02193
C	1.47561	3.66757	-0.07075
C	1.03250	4.86877	-0.62415
C	1.84999	3.56408	1.26743
C	0.94808	5.99010	0.19511
H	0.78442	4.92702	-1.68296
C	1.77065	4.69790	2.07062
H	2.19000	2.60894	1.66482
C	1.31627	5.90319	1.53796
H	0.60965	6.93770	-0.21937
H	2.06742	4.63896	3.11622
C	0.06658	-2.94115	0.86640
H	0.00438	-3.25054	1.91798
H	1.25768	6.78644	2.17162
H	5.36017	-3.14910	3.65888
Au	1.08319	-1.17508	-2.01554
P	2.14102	-1.46332	-4.10803
H	2.66416	-0.28544	-4.66675
H	3.26573	-2.30164	-4.18423
H	1.39479	-1.95068	-5.19405
N	-1.50666	0.82070	0.54979
O	-2.76319	-0.04957	-0.08287
C	-2.16678	1.81984	1.15862
C	-3.83216	0.50333	0.40648
C	-3.54308	1.67635	1.10630
C	-5.11731	-0.15497	0.21404
H	-4.24565	2.32882	1.60895
C	-5.19131	-1.34504	-0.52348
C	-6.28411	0.40315	0.75381
C	-6.41688	-1.96829	-0.71103
H	-4.28392	-1.77254	-0.94676
C	-7.50713	-0.22333	0.56020
H	-6.23868	1.32642	1.32892
C	-7.57429	-1.40880	-0.17068
H	-6.47277	-2.89238	-1.28274
H	-8.41090	0.21168	0.98176
H	-8.53459	-1.89886	-0.32069
C	-1.02538	2.16968	-1.98802
H	-1.65700	2.87687	-1.42578
H	-0.54535	2.69877	-2.81616
H	-1.65998	1.37749	-2.40545
C	-1.24041	-3.33456	0.20044
H	-1.36599	-4.42411	0.23621
H	-2.09110	-2.86624	0.71304
H	-1.25514	-3.02241	-0.85344
C	1.28305	-3.61152	0.25183
H	1.32655	-3.46841	-0.83700
H	2.22080	-3.24540	0.68930
H	1.22098	-4.69209	0.43477
C	-1.43180	2.91283	1.84187
H	-2.13035	3.52263	2.42236
H	-0.65574	2.50425	2.49723
H	-0.93557	3.55664	1.10207

Imaginary frequencies: -520.13 cm^{-1}

***Z*-Int3$_{major}$, E$_{total}$ = - 2895.450102776813**

C	0.06809	0.43233	-0.21901
C	-0.42947	-0.95217	0.08102
N	-0.29398	1.47258	0.64227
N	-1.05422	-1.65322	-0.81608
S	-1.80265	1.40946	1.43550
O	-2.51261	0.28036	0.83752

O	-1.59093	1.43271	2.87718
S	-1.74066	-0.95829	-2.35414
O	-1.81461	0.47571	-2.14748
O	-0.93858	-1.53819	-3.41633
C	-3.36180	-1.65195	-2.35005
C	-3.63705	-2.72574	-3.19509
C	-4.31818	-1.08985	-1.50337
C	-4.92501	-3.25020	-3.19034
H	-2.86437	-3.13082	-3.84555
C	-5.59698	-1.63373	-1.51078
H	-4.06252	-0.25423	-0.85346
C	-5.89639	-2.70784	-2.34970
H	-5.16923	-4.08347	-3.84531
H	-6.36305	-1.21563	-0.86164
C	-2.54892	2.93635	0.94164
C	-2.67460	3.96229	1.87422
C	-2.95242	3.07797	-0.38633
C	-3.22206	5.17282	1.45714
H	-2.35274	3.81316	2.90292
C	-3.49771	4.29258	-0.78406
H	-2.83238	2.25689	-1.09239
C	-3.62792	5.33599	0.13430
H	-3.33060	5.98839	2.16887
H	-3.82092	4.42651	-1.81426
C	0.64564	2.50575	1.20587
C	1.87649	1.84031	1.79010
C	0.94314	3.65639	0.26161
H	0.08008	2.92703	2.04744
H	2.45744	1.29927	1.03360
H	1.59168	1.12822	2.57765
H	1.65000	3.38266	-0.52931
H	0.02517	4.05119	-0.19431
C	-1.30337	-3.09716	-0.67985
H	-1.14529	-3.58181	-1.64853
H	-2.32761	-3.27071	-0.33311
H	-4.05269	6.28539	-0.18624
H	-6.90130	-3.12552	-2.35002
H	2.52222	2.60675	2.23720
H	1.40431	4.46625	0.84134
H	-0.58844	-3.50111	0.04079
Au	0.31440	-1.70169	1.89135
P	1.24176	-2.42768	3.93375
H	0.34779	-2.81017	4.94302
H	2.09653	-3.53730	3.88509
H	2.03910	-1.49265	4.60851
N	0.95993	0.41058	-1.11524
O	3.45468	-0.33404	-0.21549
C	1.69881	1.30949	-1.82741
C	3.90212	0.39686	-1.10766
C	3.06145	1.31513	-1.85798
C	5.35919	0.34340	-1.44106
H	3.52665	2.02656	-2.53601
C	6.22852	-0.22831	-0.50324
C	5.88391	0.81445	-2.65119
C	7.59164	-0.31286	-0.75893
H	5.81452	-0.59991	0.43252
C	7.24773	0.71790	-2.91404
H	5.22971	1.23706	-3.41170
C	8.10452	0.16011	-1.96729
H	8.25808	-0.74913	-0.01646
H	7.64159	1.07732	-3.86332
H	9.17194	0.09127	-2.17160
C	0.89374	2.16063	-2.76615
H	1.51083	2.95328	-3.20226
H	0.50241	1.53398	-3.57908
H	0.02534	2.60393	-2.26296

Imaginary frequencies: none

***Z*-Int3$_{minor}$, E$_{total}$ = - 2895.458901038114**

C	-0.10336	0.53714	0.50747
C	0.41106	-0.80328	0.08229
N	0.00161	1.61846	-0.35281
N	1.29610	-1.43594	0.78815
S	1.16510	1.67854	-1.59073
O	2.02231	0.51619	-1.35899
O	0.47812	1.81785	-2.86642
S	2.27192	-0.64741	2.09974
O	2.09332	0.78461	1.93455
O	1.87834	-1.32647	3.32185
C	3.91489	-1.10144	1.63563
C	4.58129	-2.07069	2.38382
C	4.49533	-0.46257	0.53898
C	5.87788	-2.41076	2.01304
H	4.09674	-2.53858	3.23872
C	5.79061	-0.81993	0.18417
H	3.94175	0.28006	-0.03451
C	6.47549	-1.78991	0.91694
H	6.42115	-3.16132	2.58263
H	6.26647	-0.33930	-0.66766

C	2.03675	3.18377	-1.25287
C	2.00721	4.19770	-2.20703
C	2.73212	3.30861	-0.04975
C	2.70862	5.37211	-1.94784
H	1.44802	4.06602	-3.13081
C	3.42418	4.48949	0.19195
H	2.72652	2.50384	0.68489
C	3.41282	5.51541	-0.75439
H	2.70116	6.17602	-2.68068
H	3.97332	4.60959	1.12344
C	1.57263	-2.91372	0.65431
H	2.31712	-3.12774	1.43059
H	3.95763	6.43655	-0.55643
H	7.48940	-2.06311	0.63119
Au	-0.80656	-1.55984	-1.46277
P	-2.30066	-2.21920	-3.15725
H	-1.94214	-1.91553	-4.47754
H	-2.60786	-3.58285	-3.26036
H	-3.58004	-1.65089	-3.07744
N	-0.81127	0.44888	1.55271
O	-3.17802	0.24865	0.11663
C	-1.59325	1.29857	2.29112
C	-3.71398	0.73372	1.12240
C	-2.94684	1.38599	2.17295
C	-5.19994	0.68454	1.26219
H	-3.46749	1.99134	2.91155
C	-5.96917	0.46340	0.11291
C	-5.84621	0.82481	2.49634
C	-7.35506	0.40080	0.19093
H	-5.45906	0.34882	-0.84242
C	-7.23388	0.74717	2.57767
H	-5.26786	0.97000	3.40747
C	-7.99033	0.54155	1.42553
H	-7.94444	0.24142	-0.71071
H	-7.72543	0.84572	3.54413
H	-9.07616	0.48878	1.48909
C	-0.91829	2.75651	-0.25917
H	-0.68432	3.38399	0.60929
H	-0.82209	3.36322	-1.16459
H	-1.94410	2.38245	-0.20880
C	0.32558	-3.70909	0.98820
H	0.58679	-4.77266	1.04561
H	-0.08976	-3.39910	1.95526
H	-0.45138	-3.59377	0.21980
C	2.18697	-3.22804	-0.69484
H	1.49309	-3.00569	-1.51772
H	3.11314	-2.66169	-0.85821
H	2.42558	-4.29761	-0.73468
C	-0.83912	1.99821	3.37867
H	-1.50218	2.61256	3.99594
H	-0.32517	1.26474	4.01455
H	-0.05416	2.63548	2.94461

Imaginary frequencies: none

6 X-Ray Crystallography

Single crystal X-ray diffraction data were collected using a (Rigaku) Oxford Diffraction SuperNova A diffractometer and reduced using CrysAlisPro. Structures were solved using Super-Flip[32] and refined using CRYSTALS[33] as per the ESI (CIF). Data for **2g** and **4a** are deposited with the CCDC: CCDC 2213976 and CCDC 2213977.

Table S2. Crystal data and structure refinement for **2g.**

CCDC Identification code	CCDC 2213976	
Empirical formula	C39 H43 N3 O5 S2	
Formula weight	697.92	
Temperature	150 K	
Wavelength	1.54184 Å	
Crystal system	Triclinic	
Space group	P -1	
Unit cell dimensions	a = 9.7137(2) Å	α= 107.043(3)°.
	b = 11.0676(4) Å	β= 99.017(2)°.
	c = 17.5505(5) Å	γ = 90.606(2)°.
Volume	1778.46(10) $Å^3$	
Z	2	
Density (calculated)	1.303 Mg/m^3	
Absorption coefficient	1.745 mm^{-1}	
F(000)	740	
Crystal size	0.18 x 0.17 x 0.10 mm^3	
Theta range for data collection	4.186 to 76.220°.	
Index ranges	-12<=h<=12, -13<=k<=11, -22<=l<=22	
Reflections collected	43066	
Independent reflections	7365 [R(int) = 0.030]	
Completeness to theta = 73.933°	99.8 %	
Absorption correction	Semi-empirical from equivalents	
Max. and min. transmission	0.84 and 0.67	
Refinement method	Full-matrix least-squares on F2	
Data / restraints / parameters	7365 / 0 / 442	
Goodness-of-fit on F2	0.9916	
Final R indices [I>2sigma(I)]	R1 = 0.0330, wR2 = 0.0895	
R indices (all data)	R1 = 0.0363, wR2 = 0.0927	
Largest diff. peak and hole	0.62 and -0.43 e.$Å^{-3}$	

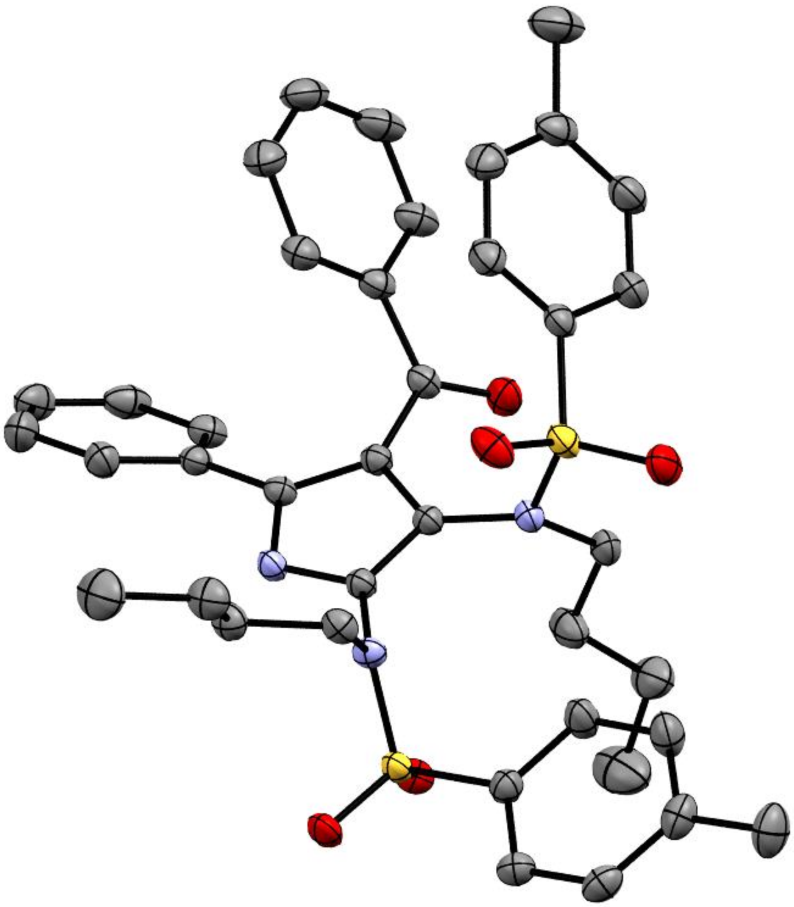

Figure S13. The structure of **2g** determined from X-ray diffraction studies, displacement ellipsoids are drawn at 50% probability and hydrogen atoms are omitted for clarity.

Table S3. Crystal data and structure refinement for **4a.**

CCDC Identification code	CCDC 2213977	
Empirical formula	C27 H35 N3 O5 S2 (C6 H6)	
Formula weight	623.83	
Temperature	150 K	
Wavelength	1.54180 Å	
Crystal system	Triclinic	
Space group	P -1	
Unit cell dimensions	a = 9.2395(2) Å	α= 93.824(2)°.
	b = 13.2474(4) Å	β= 96.199(2)°.
	c = 13.6052(4) Å	γ = 95.206(2)°.
Volume	1543.77(8) $Å^3$	
Z	2	
Density (calculated)	1.260 Mg/m^3	
Absorption coefficient	1.822 mm^{-1}	
F(000)	664	
Crystal size	0.04 x 0.18 x 0.30 mm^3	
Theta range for data collection	3.277 to 76.267°.	
Index ranges	-11<=h<=11, -16<=k<=16, -18<=l<=16.	
Reflections collected	38196	
Independent reflections	6825 [R(int) = 0.0307]	
Completeness to theta = 76.276°	98.9 %	
Absorption correction	Semi-empirical from equivalents	
Max. and min. transmission	0.93 and 0.75	
Refinement method	Full-matrix least-squares on F2	
Data / restraints / parameters	6825 / 11 / 401	
Goodness-of-fit on F2	0.9958	
Final R indices [I>2sigma(I)]	R1 = 0.0307, wR2 = 0.0819	
R indices (all data)	R1 = 0.0327, wR2 = 0.0844	
Largest diff. peak and hole	0.93 and -0.34 e.$Å^{-3}$	

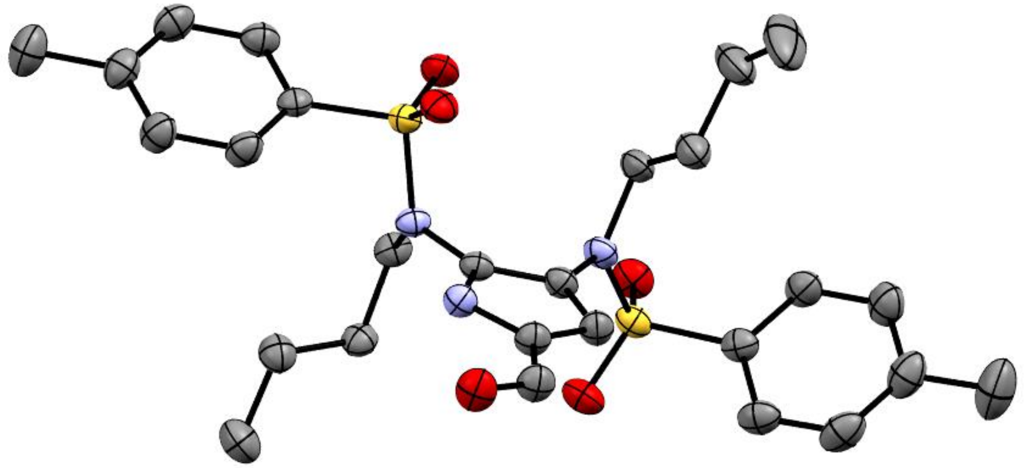

Figure S14. The structure of **4a** determined from X-ray diffraction studies, displacement ellipsoids are drawn at 50% probability, hydrogen atoms and residual solvent are omitted for clarity.

7 NMR Spectra for Novel Compounds

***N*-Butyl-*N*-(((*N*-cyclohexyl-4-methylphenyl)sulfonamido)ethynyl)-4-methylbenzenesulfonamide, 1b**

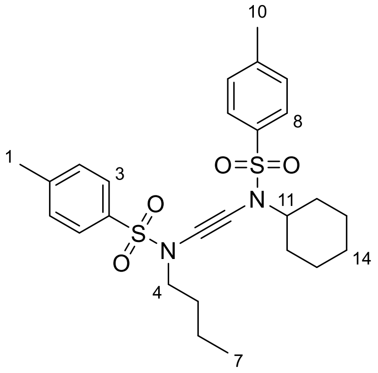

^{1}H NMR (400 MHz, $CDCl_3$)

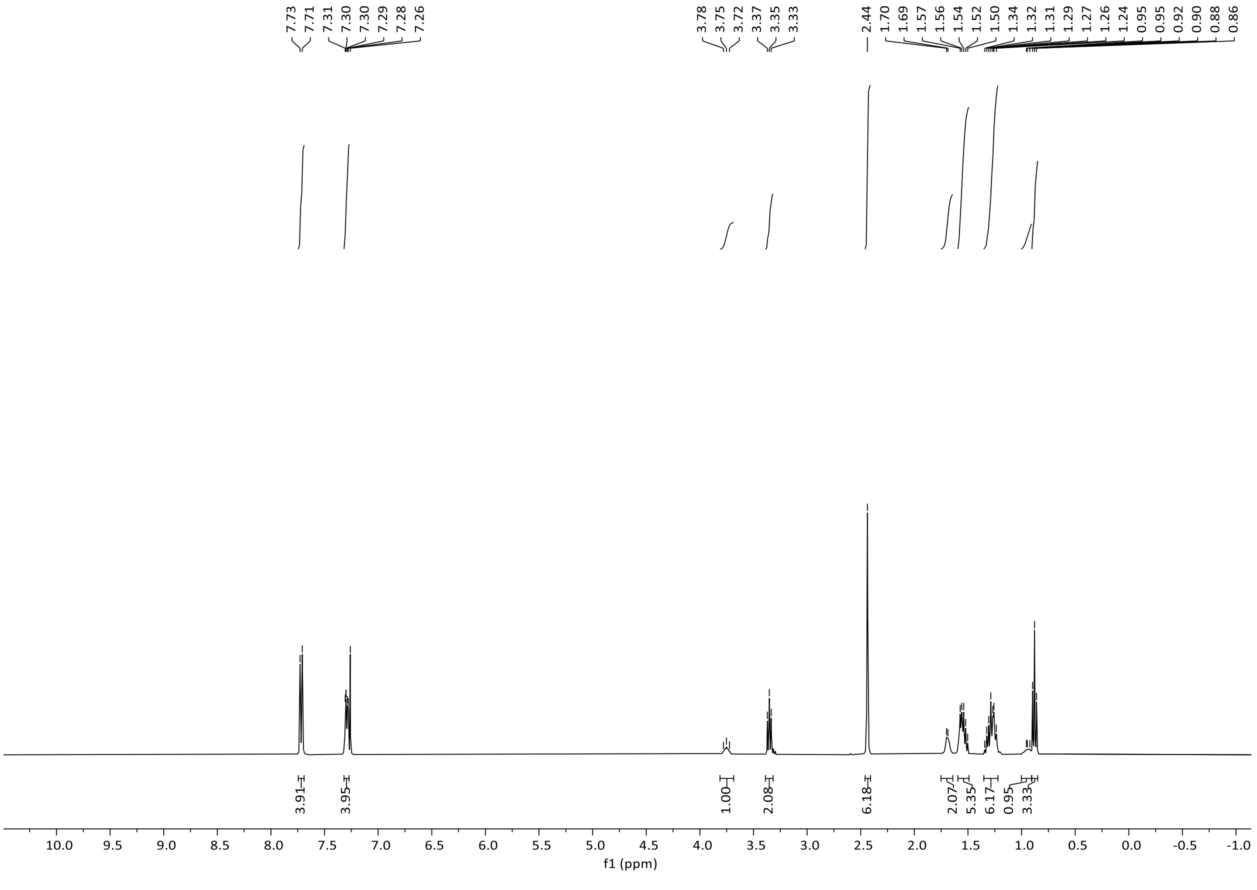

^{13}C NMR (101 MHz, $CDCl_3$)

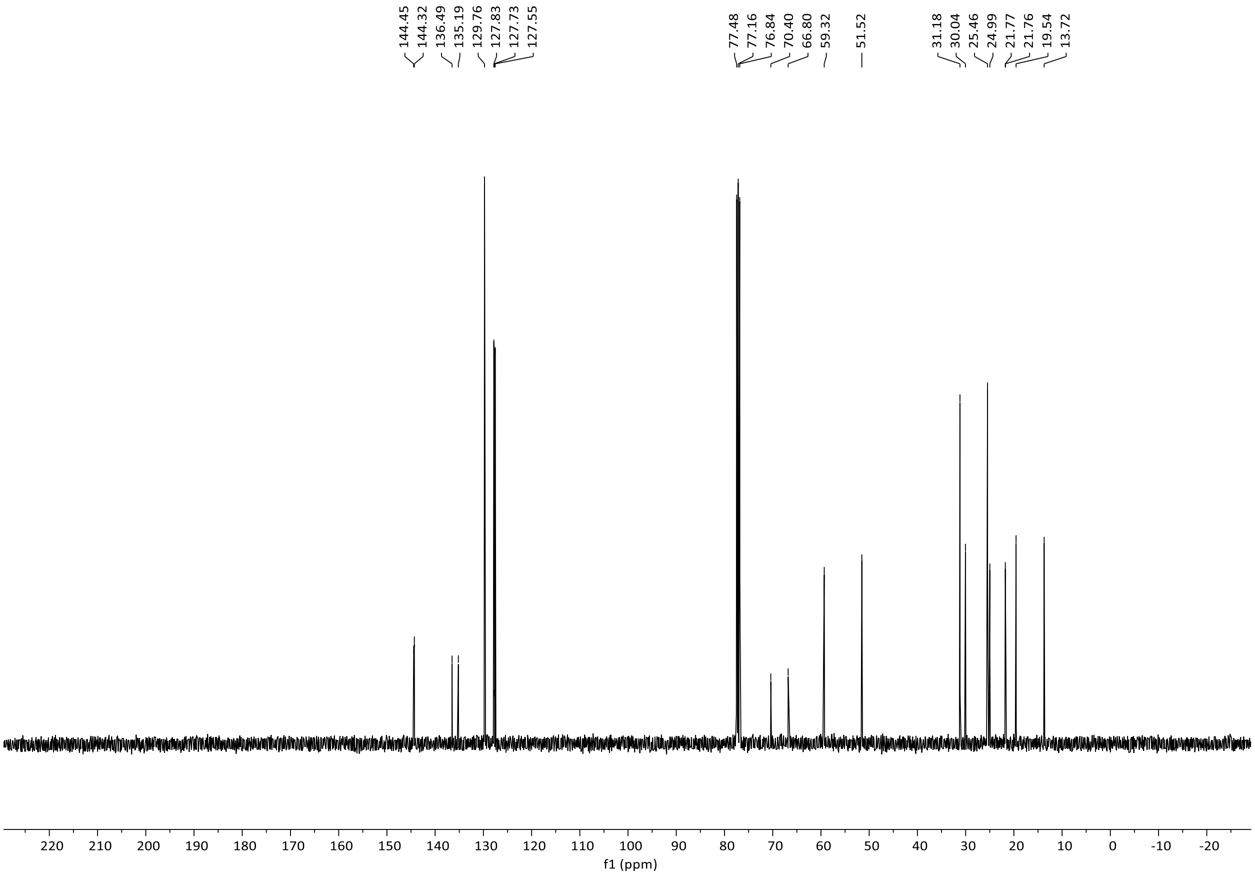

***N*-(5-(*tert*-Butyl)isoxazol-3-yl)-4-methylbenzenesulfonamide, S2d**

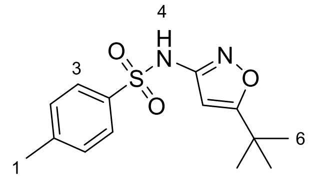

^{1}H NMR (400 MHz, DMSO)

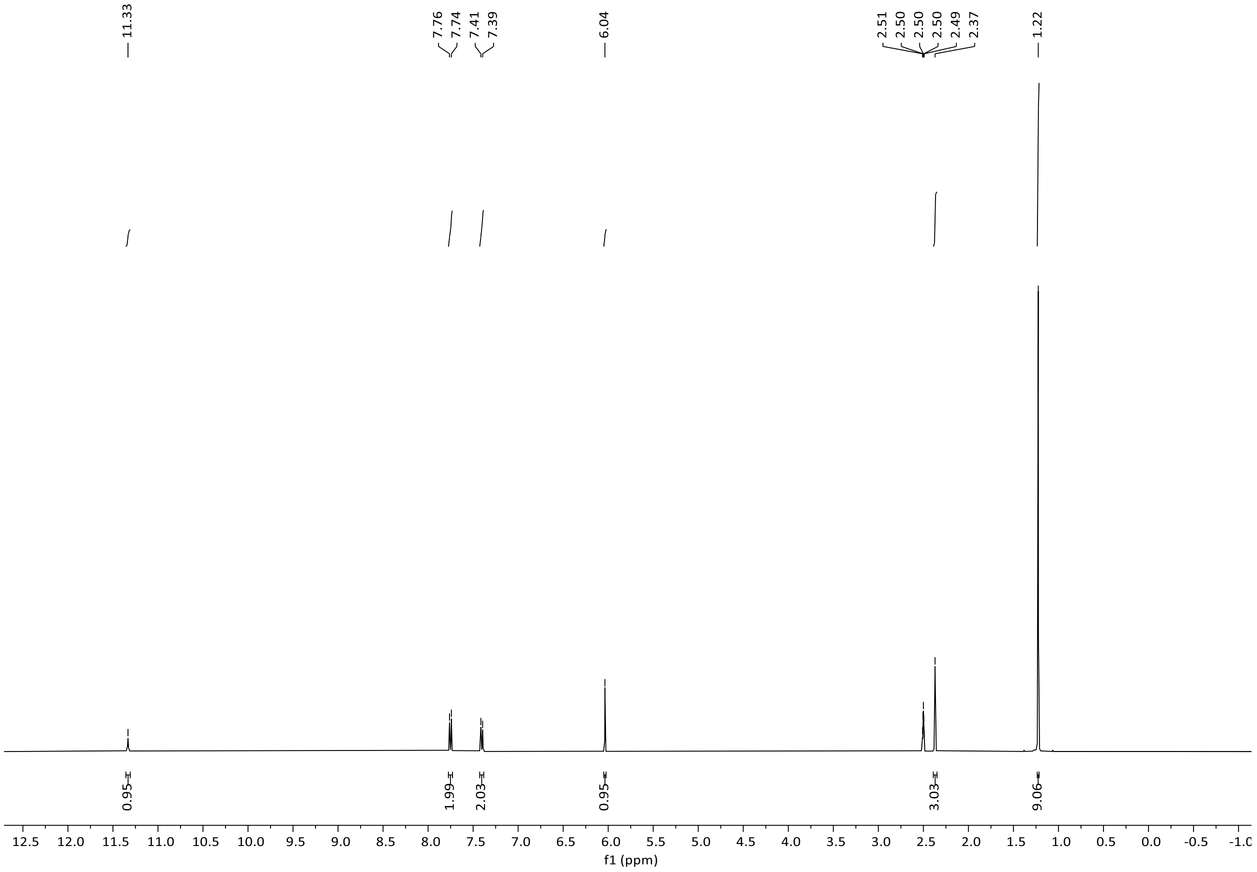

^{13}C NMR (101 MHz, DMSO)

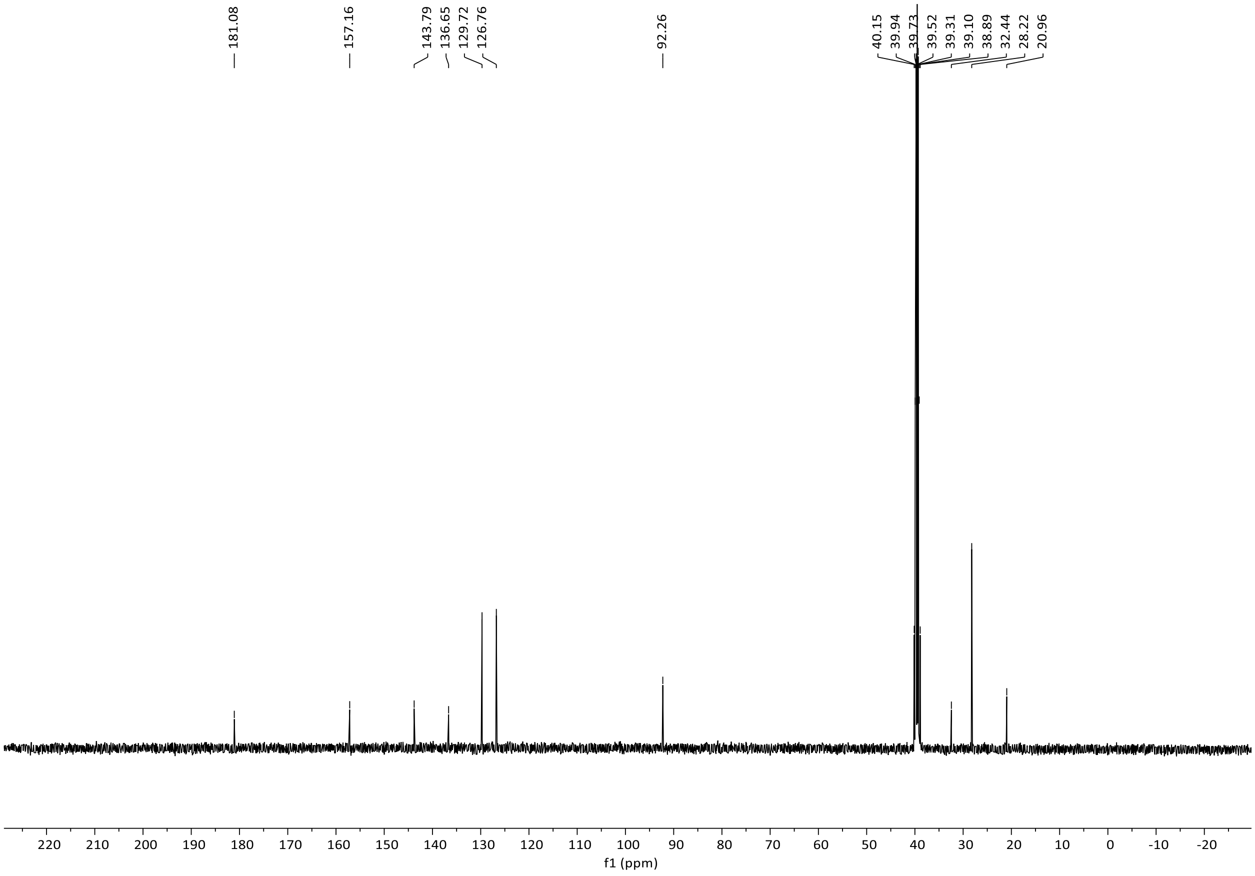

7-(*tert*-Butyl)benzo[*d*]isoxazole, S2yd

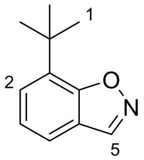

^{1}H NMR (400 MHz, $CDCl_3$)

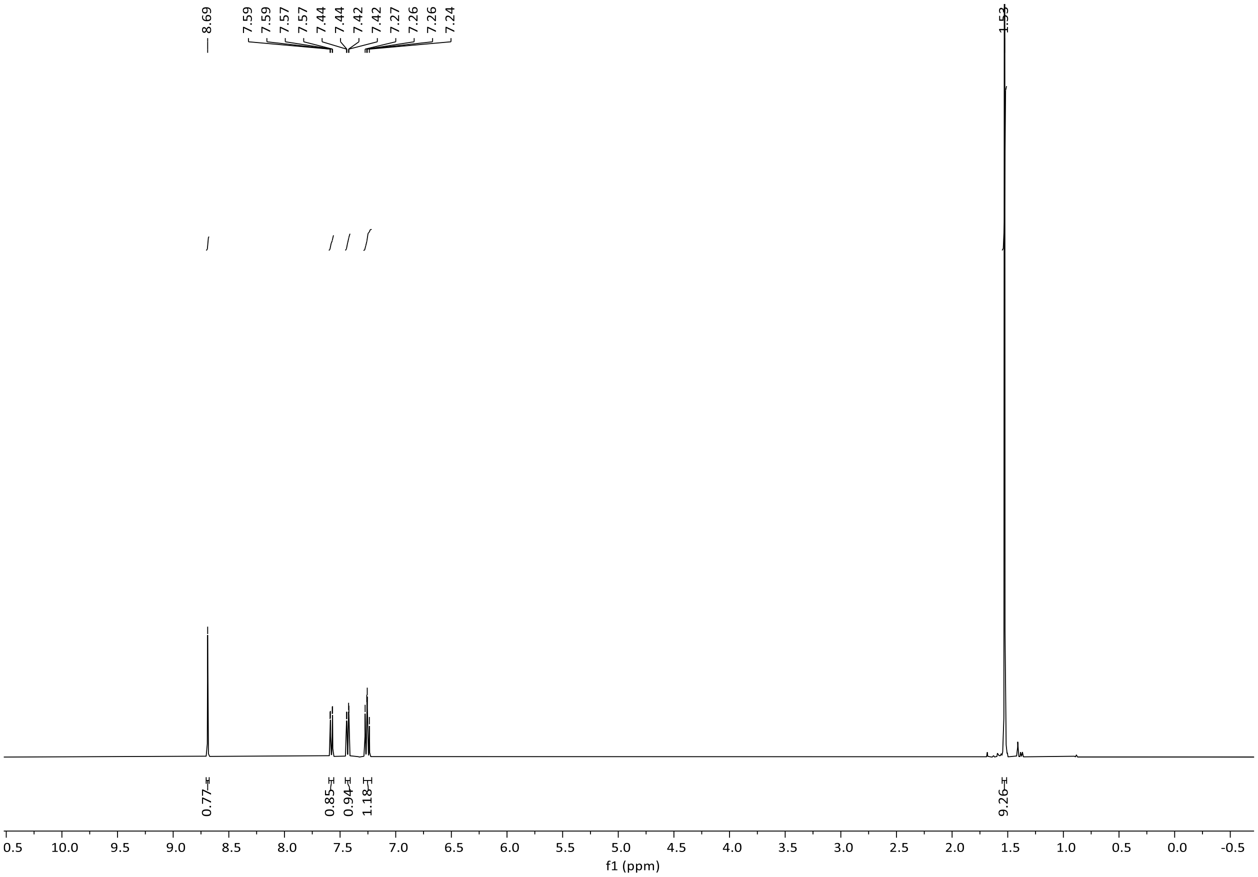

^{13}C NMR (101 MHz, $CDCl_3$)

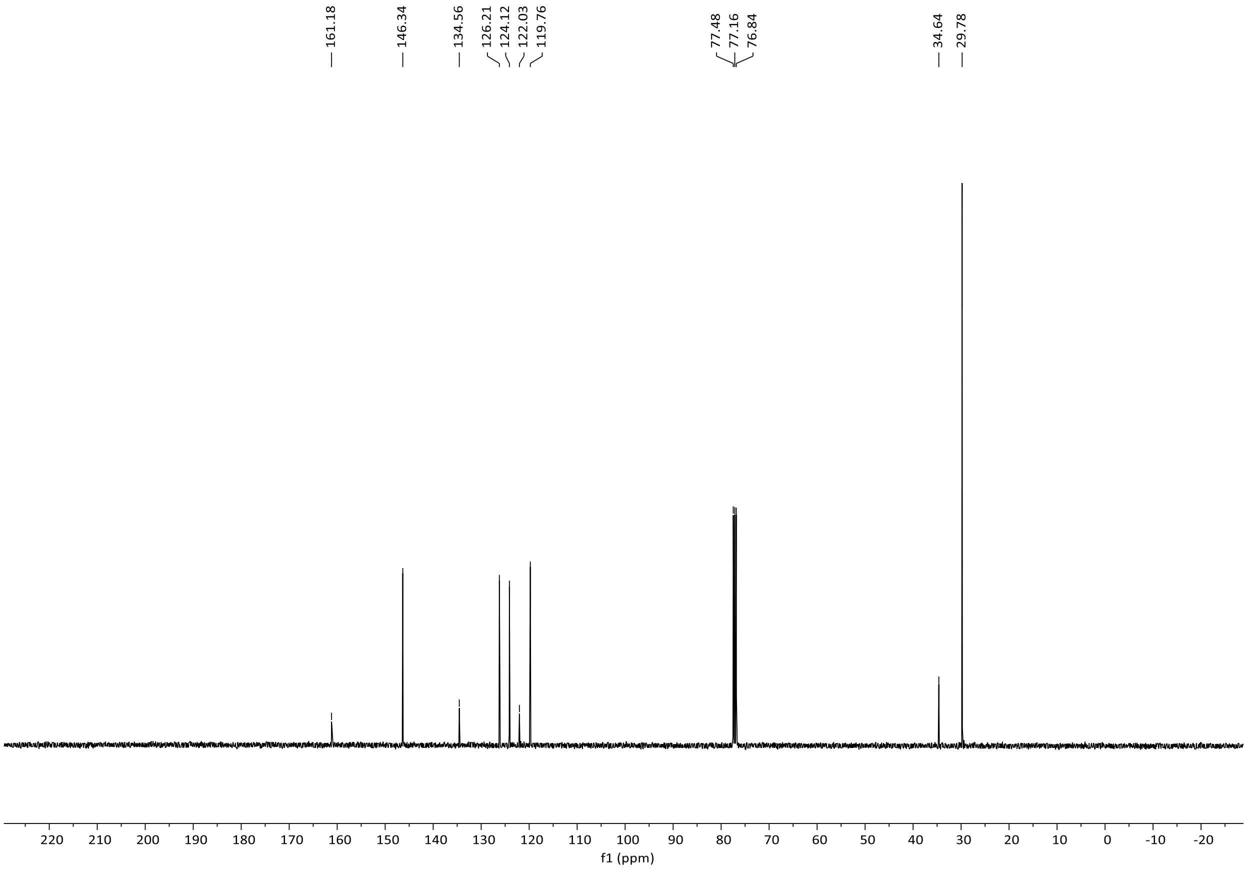

***N*,*N'*-(4-Formyl-1*H*-pyrrole-2,3-diyl)bis(*N*-butyl-4-methylbenzenesulfonamide), 2a**

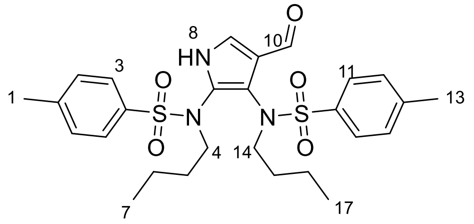

^{1}H NMR (500 MHz, C_6D_6)

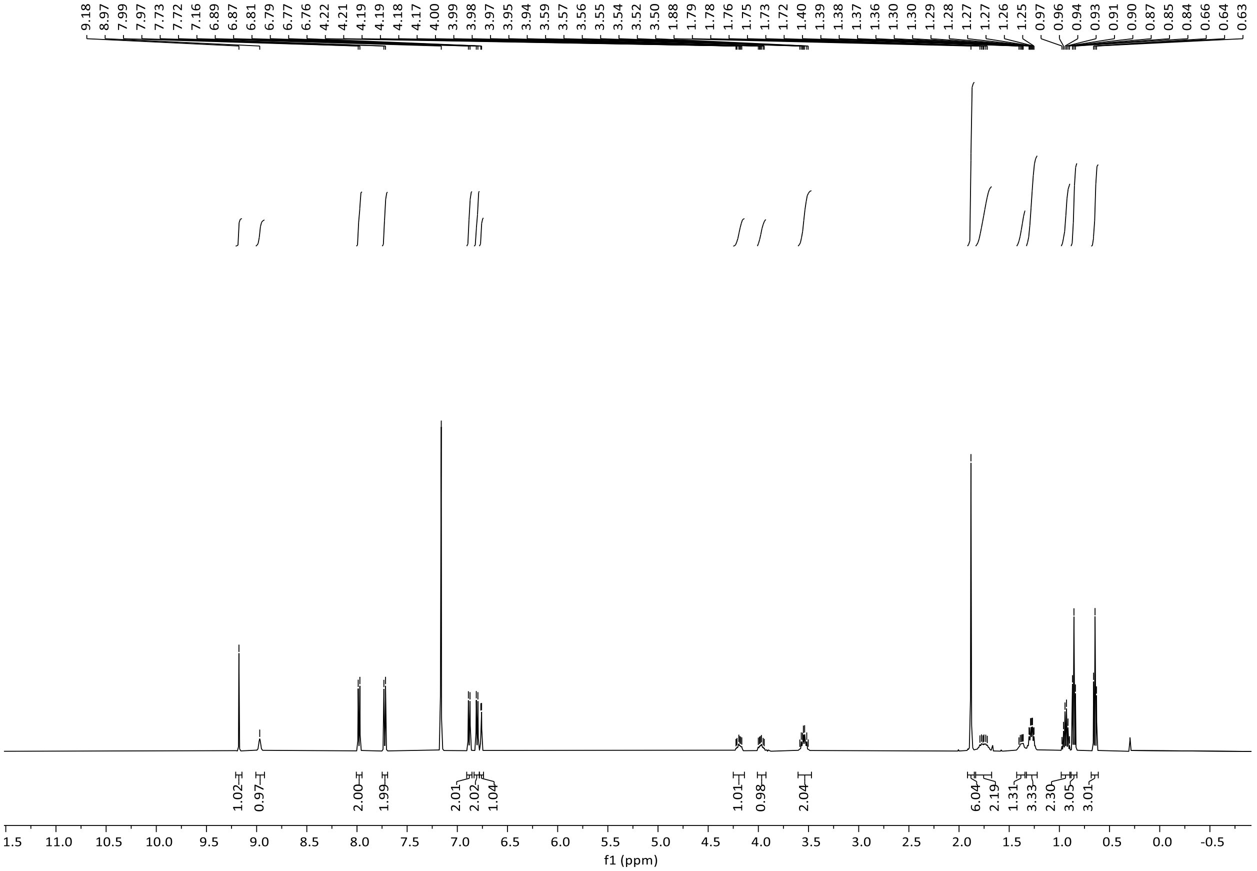

^{13}C NMR (126 MHz, C_6D_6)

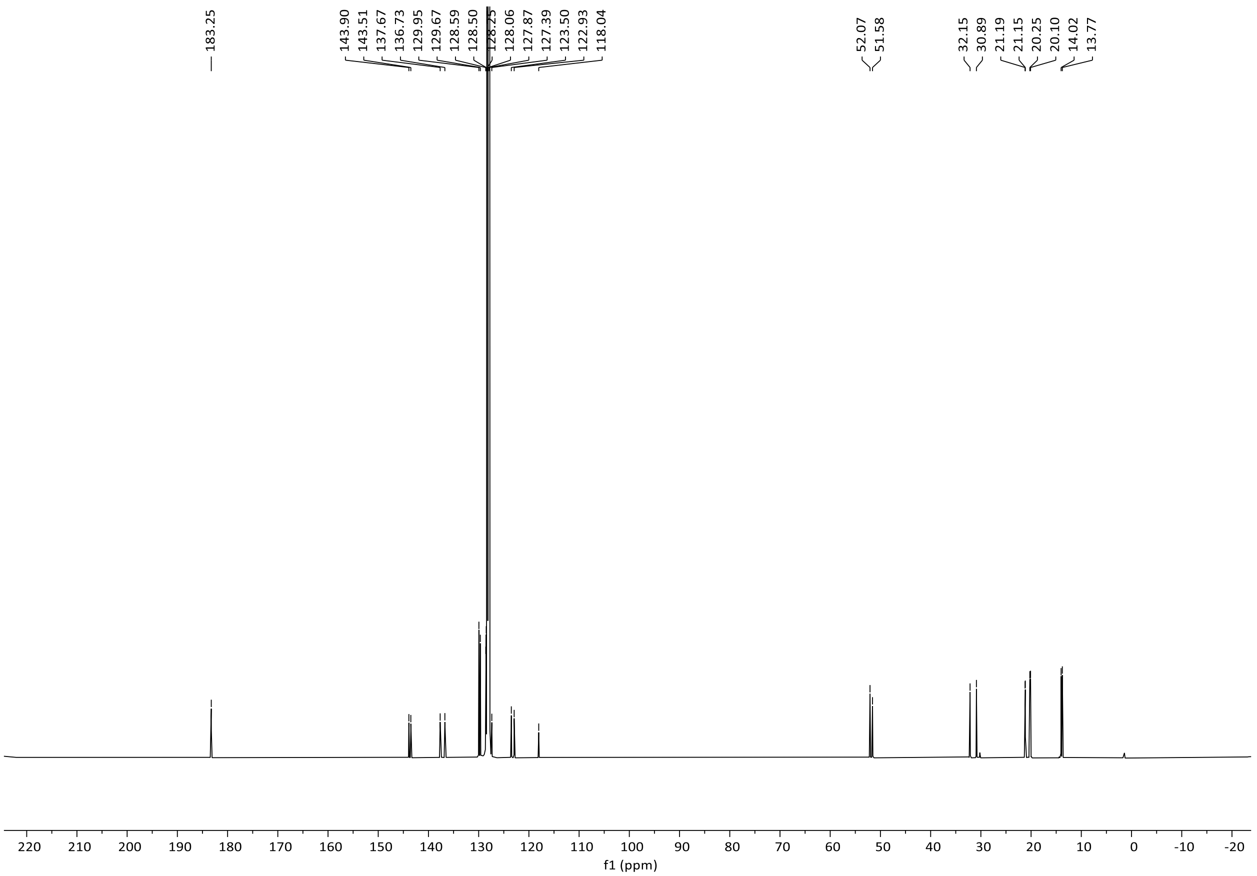

***N*,*N'*-(5-Formyl-1*H*-pyrrole-2,3-diyl)bis(*N*-butyl-4-methylbenzenesulfonamide), 4a**

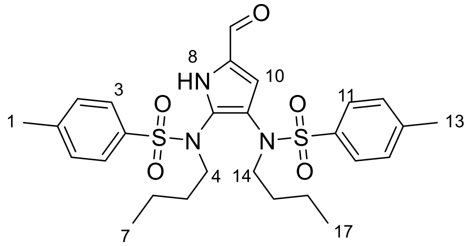

^{1}H NMR (400 MHz, C_6D_6)

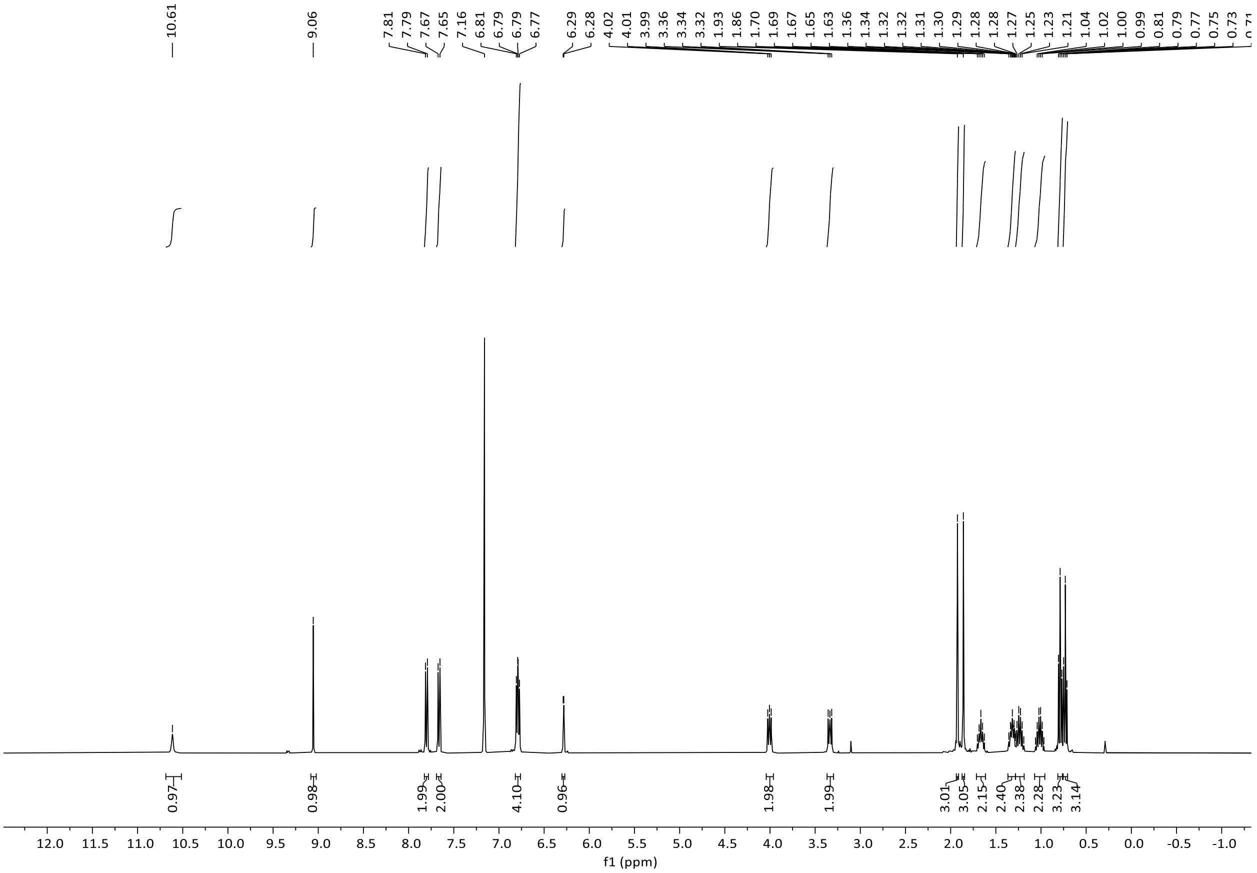

^{13}C NMR (101 MHz, C_6D_6)

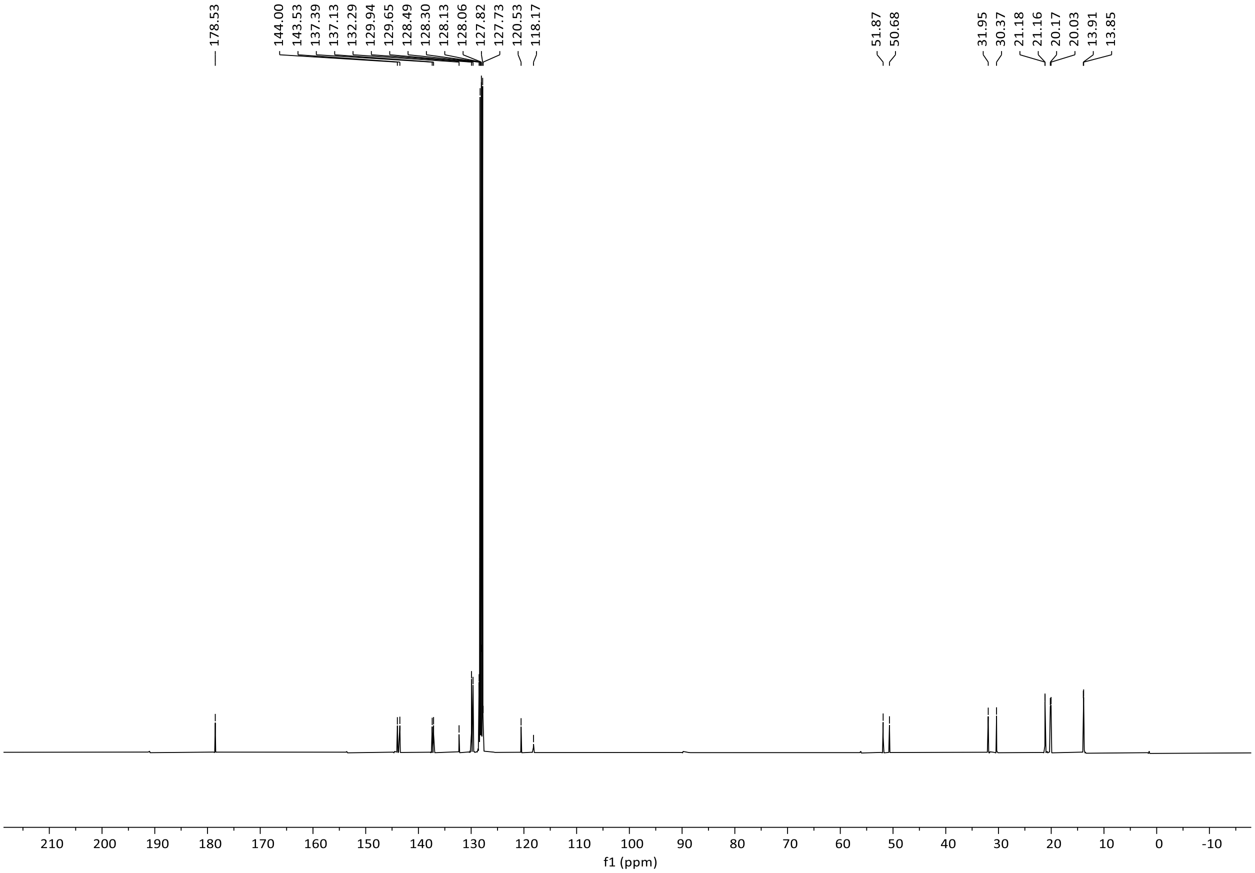

***N*,*N'*-(4-Acetyl-5-methyl-1*H*-pyrrole-2,3-diyl)bis(*N*-butyl-4-methylbenzenesulfonamide), 2b**

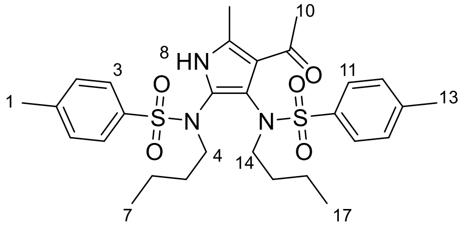

^{1}H NMR (400 MHz, $CDCl_3$)

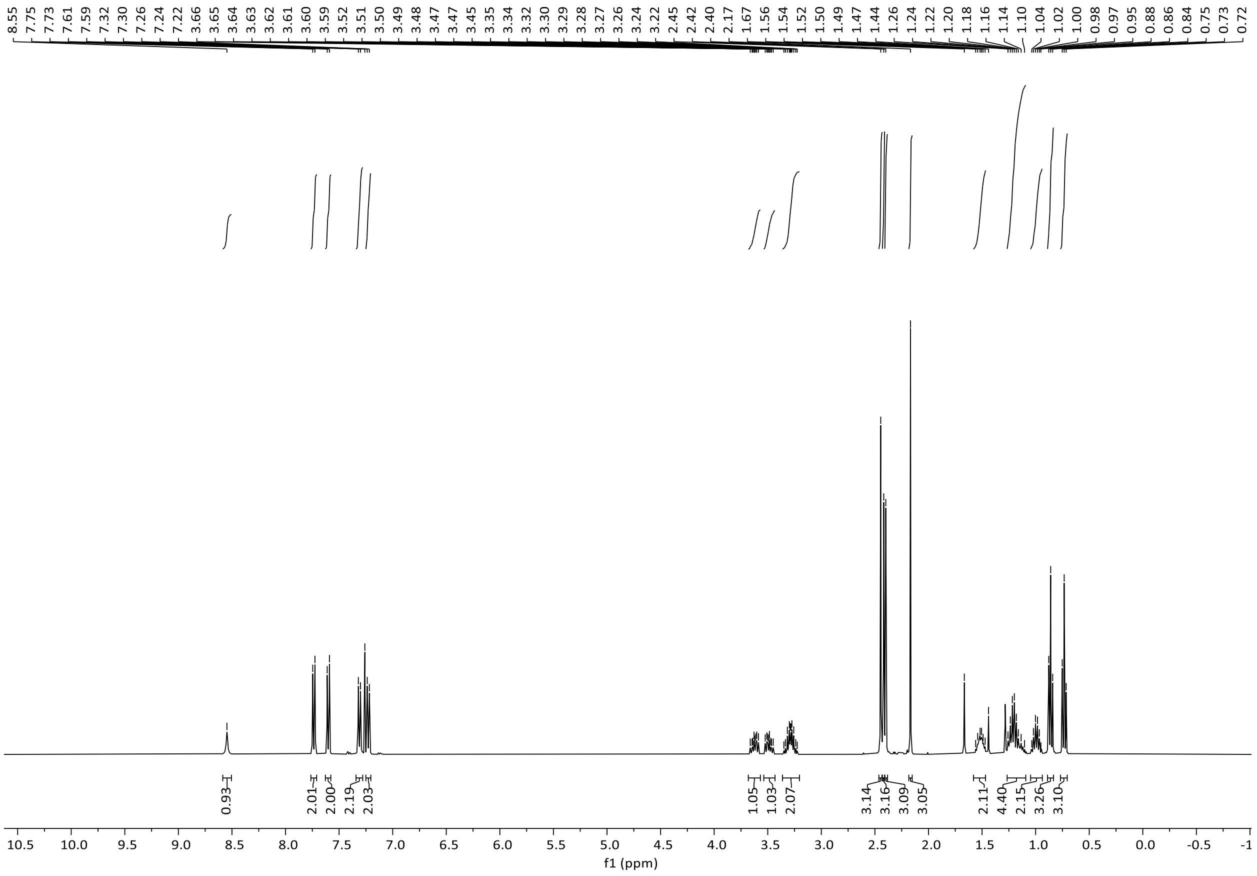

^{13}C NMR (101 MHz, $CDCl_3$)

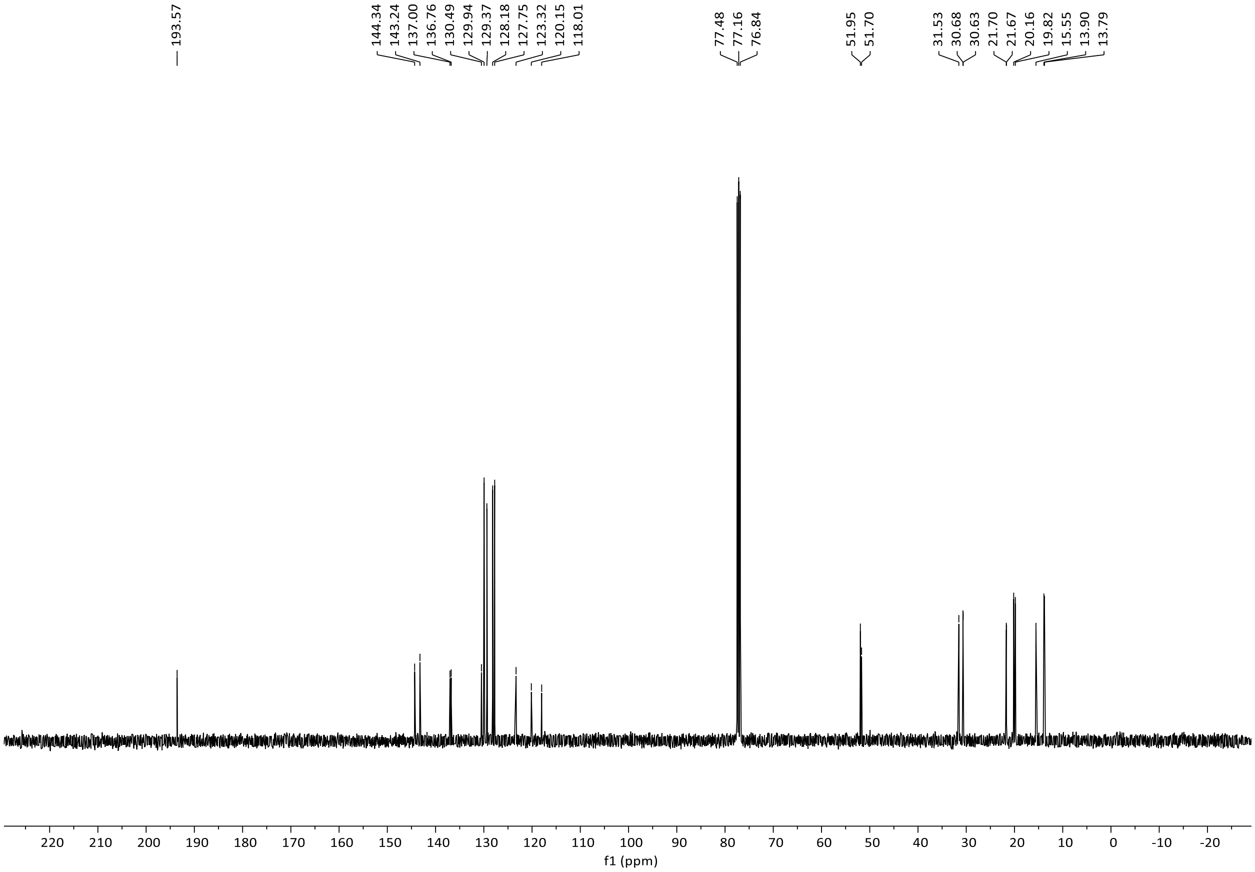

***N*,*N'*-(5-Methyl-7-methylene-2,7-dihydro-1,4-oxazepine-2,3-diyl)bis(*N*-butyl-4-methylbenzenesulfonamide), 5b**

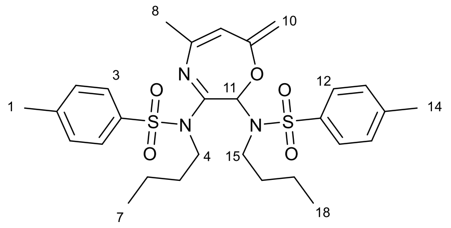

^{1}H NMR (400 MHz, C_6D_6)

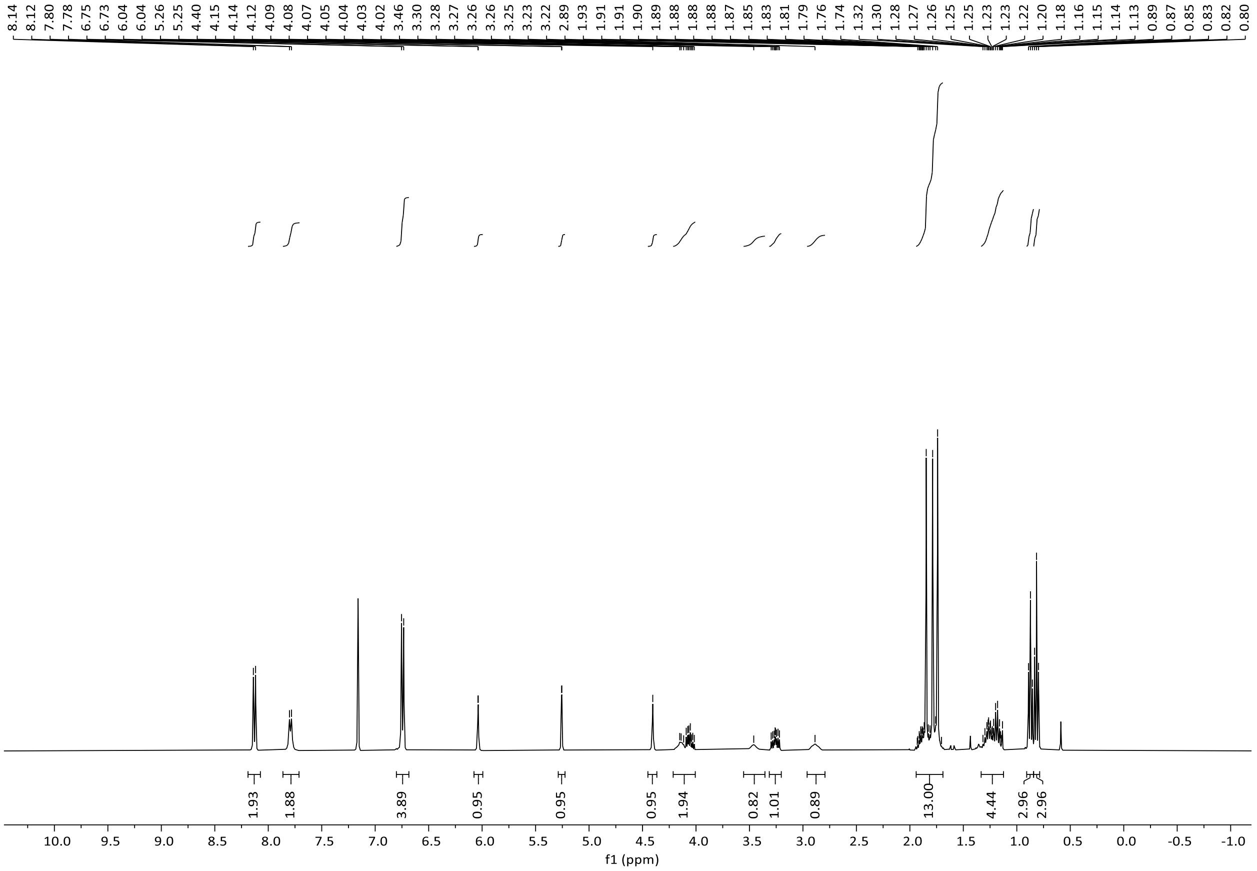

^{13}C NMR (101 MHz, C_6D_6)

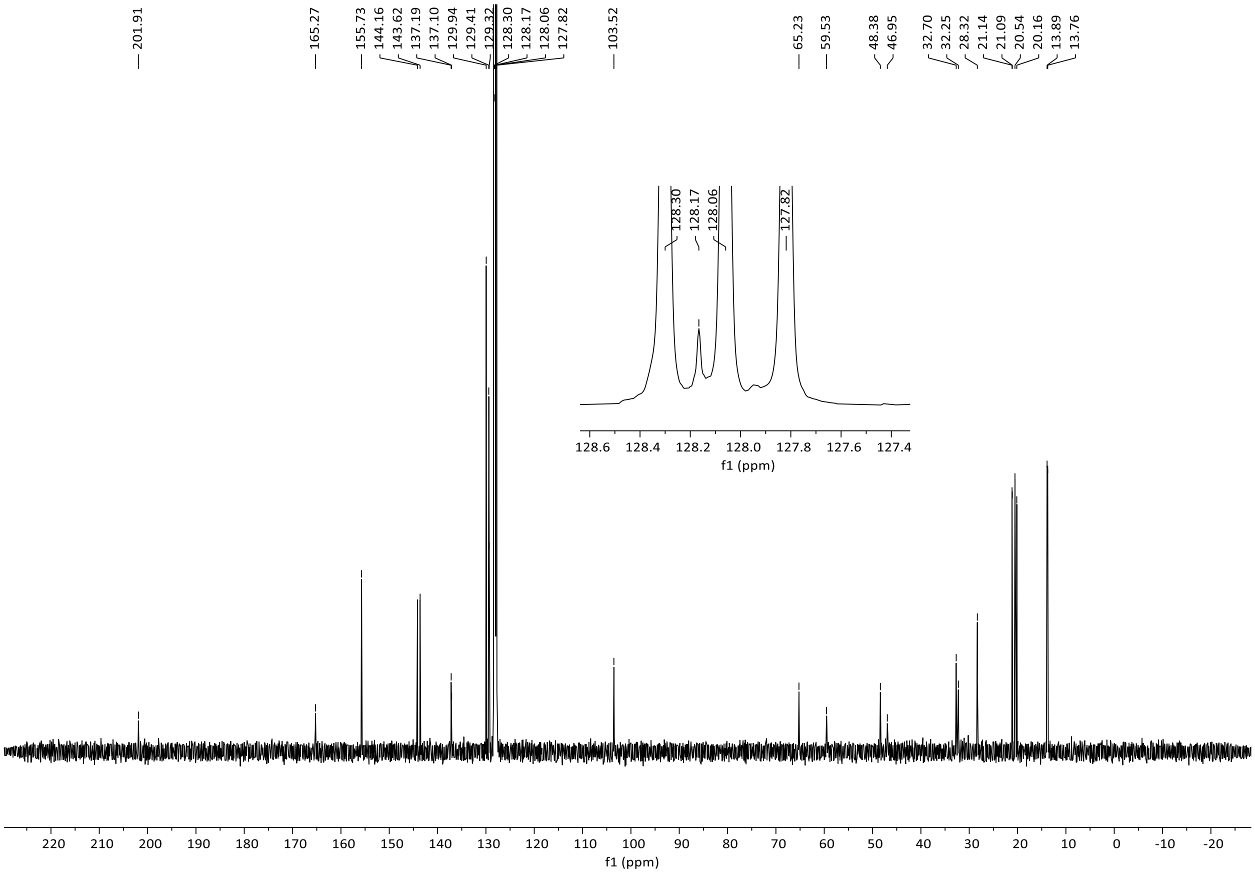

***N*,*N'*-(4-Acetyl-1*H*-pyrrole-2,3-diyl)bis(*N*-butyl-4-methylbenzenesulfonamide), 2c**

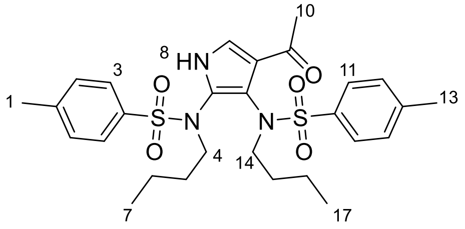

^{1}H NMR (400 MHz, $CDCl_3$)

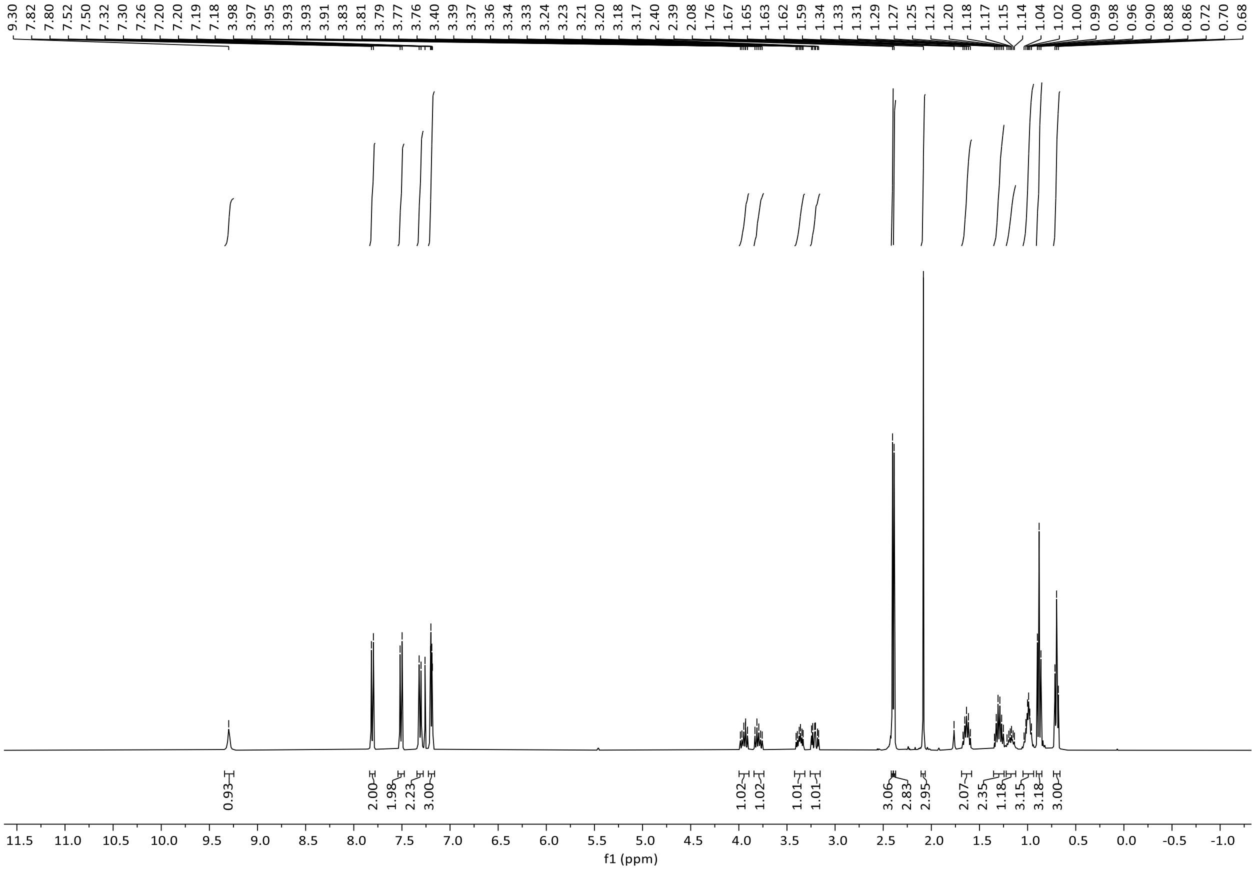

^{13}**C NMR** (101 MHz, $CDCl_3$)

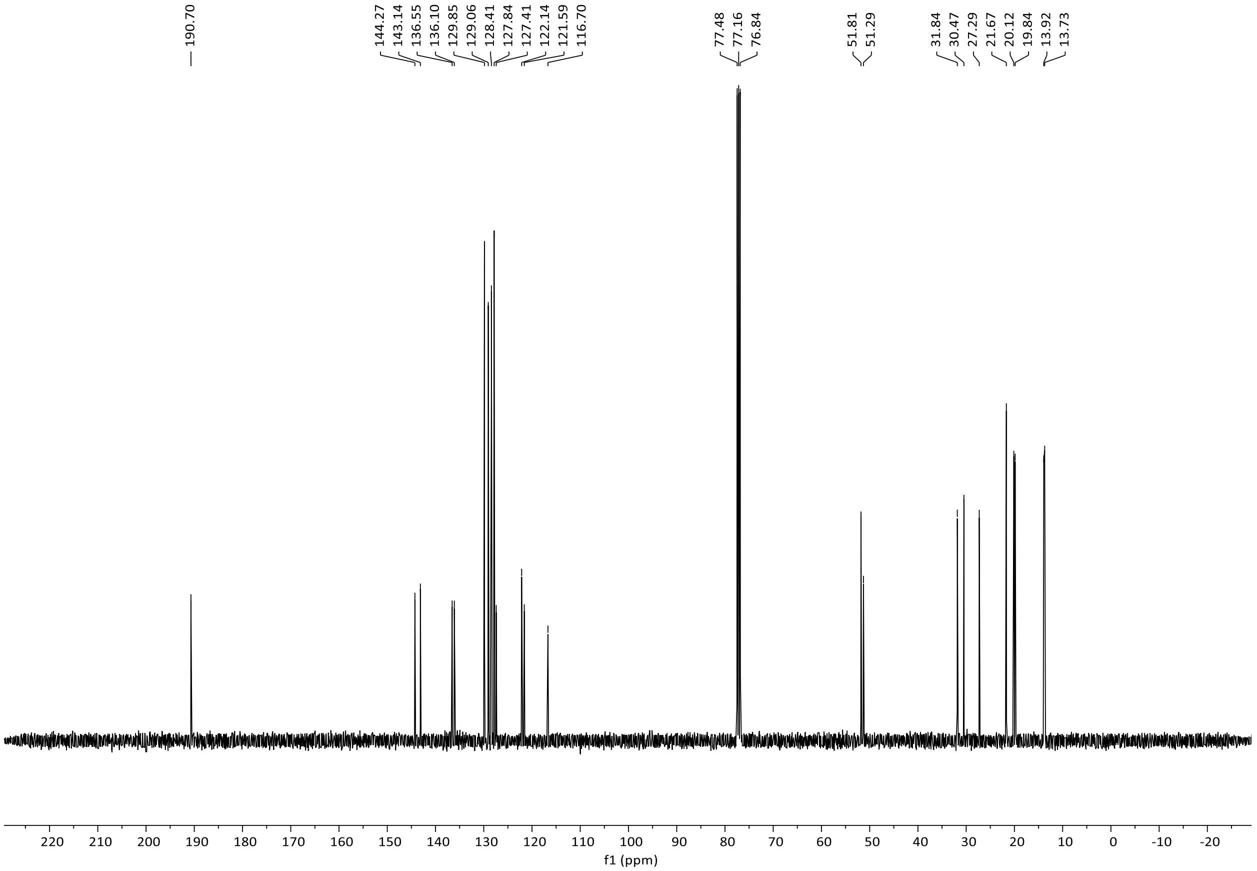

***N*,*N'*-(5-((4-Methylphenyl)sulfonamido)-4-pivaloyl-1*H*-pyrrole-2,3-diyl)bis(*N*-butyl-4-methylbenzenesulfonamide), 2d**

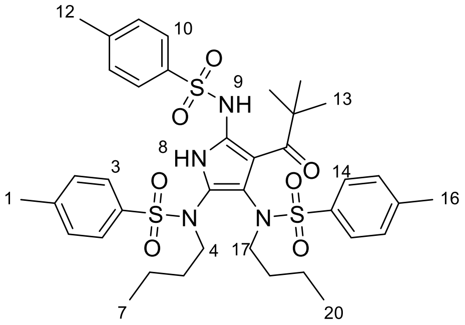

^{1}H NMR (400 MHz, $CDCl_3$)

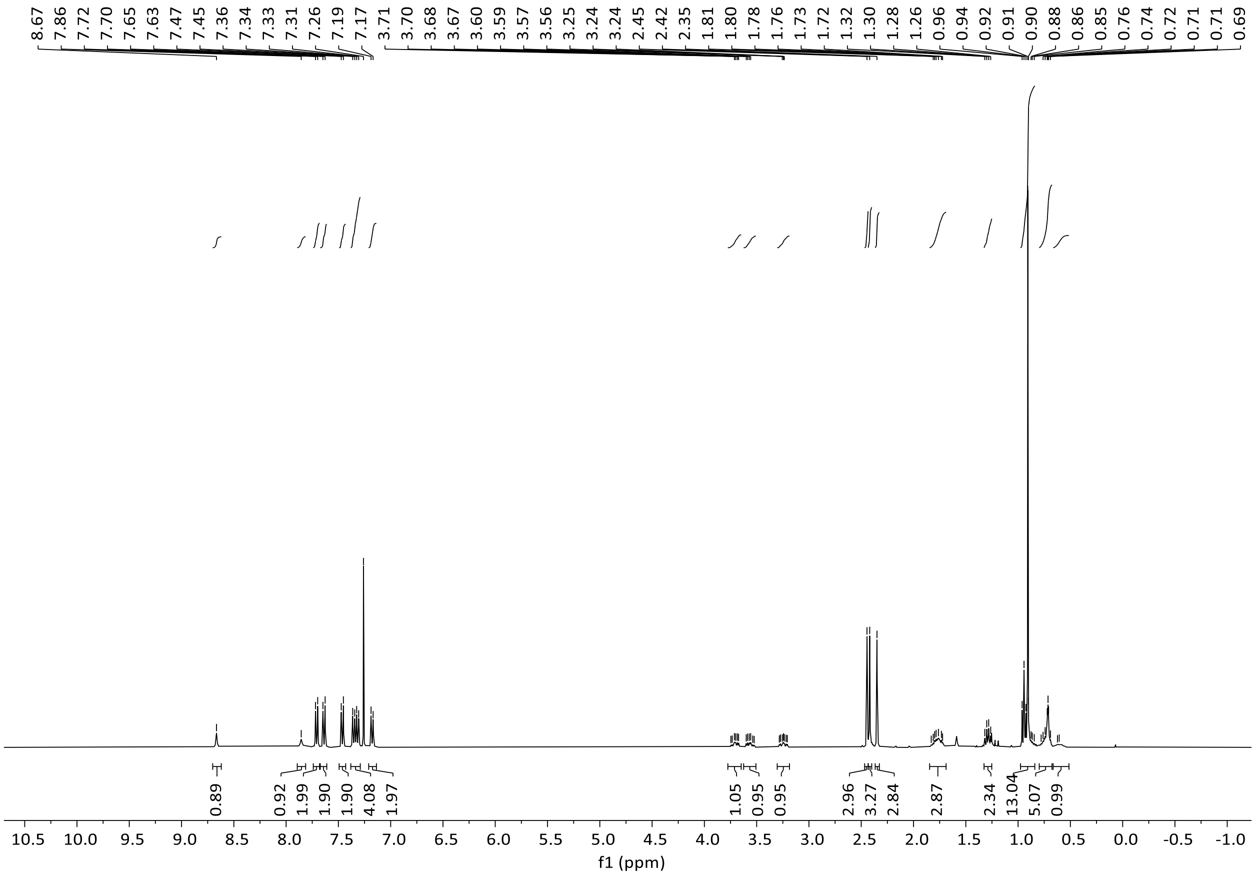

^{13}C NMR (101 MHz, $CDCl_3$)

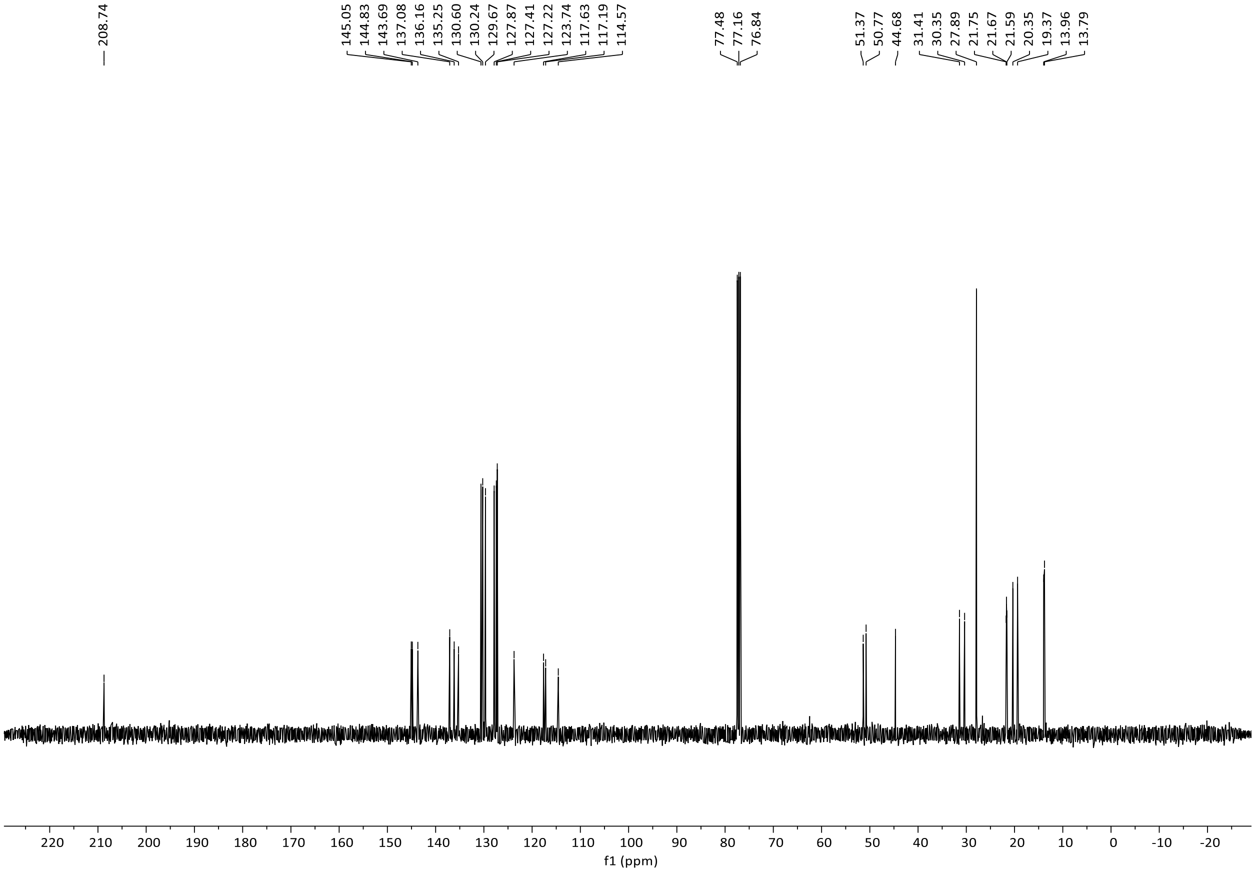

Methyl 2-(4,5-bis((*N*-butyl-4-methylphenyl)sulfonamido)-1*H*-pyrrol-3-yl)-2-oxoacetate, 2e

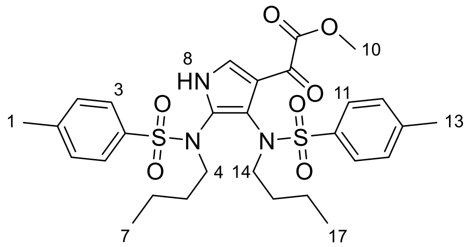

^{1}H NMR (400 MHz, $CDCl_3$)

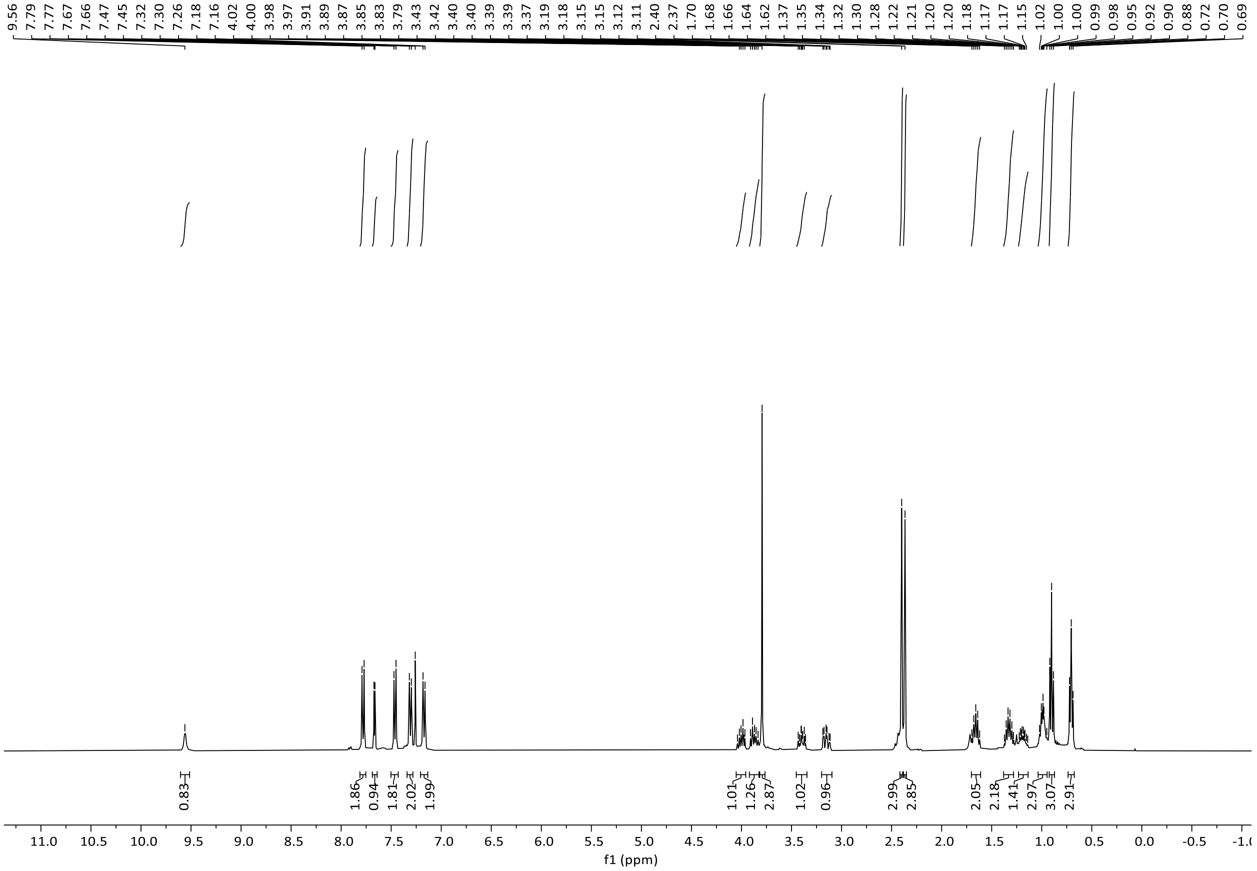

^{13}C NMR (101 MHz, $CDCl_3$)

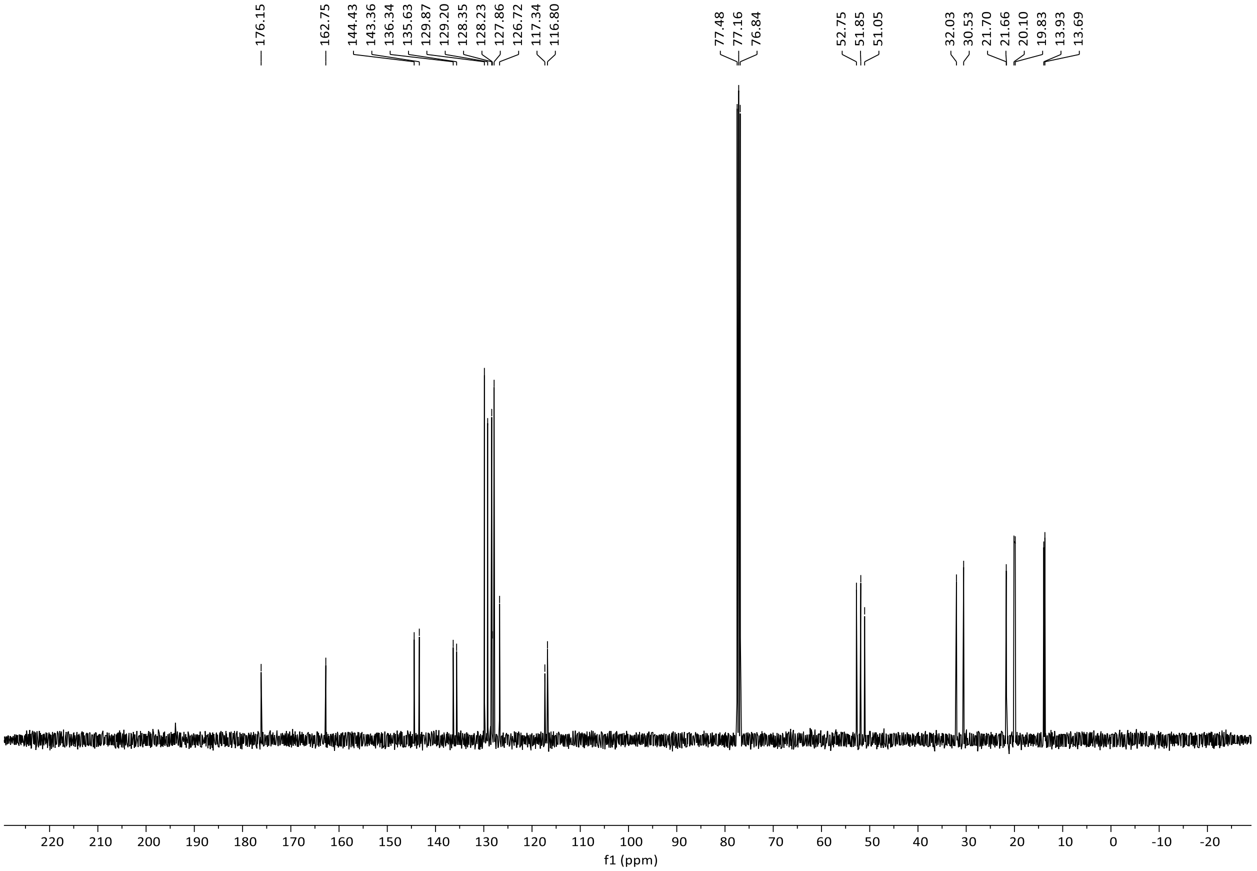

Methyl 3-acetyl-4,5-bis((N-butyl-4-methylphenyl)sulfonamido)-1*H*-pyrrole-2-carboxylate, 2f

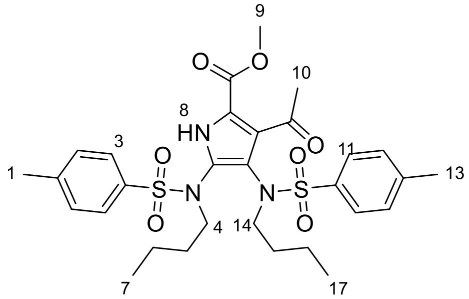

^{1}H NMR (400 MHz, C_6D_6)

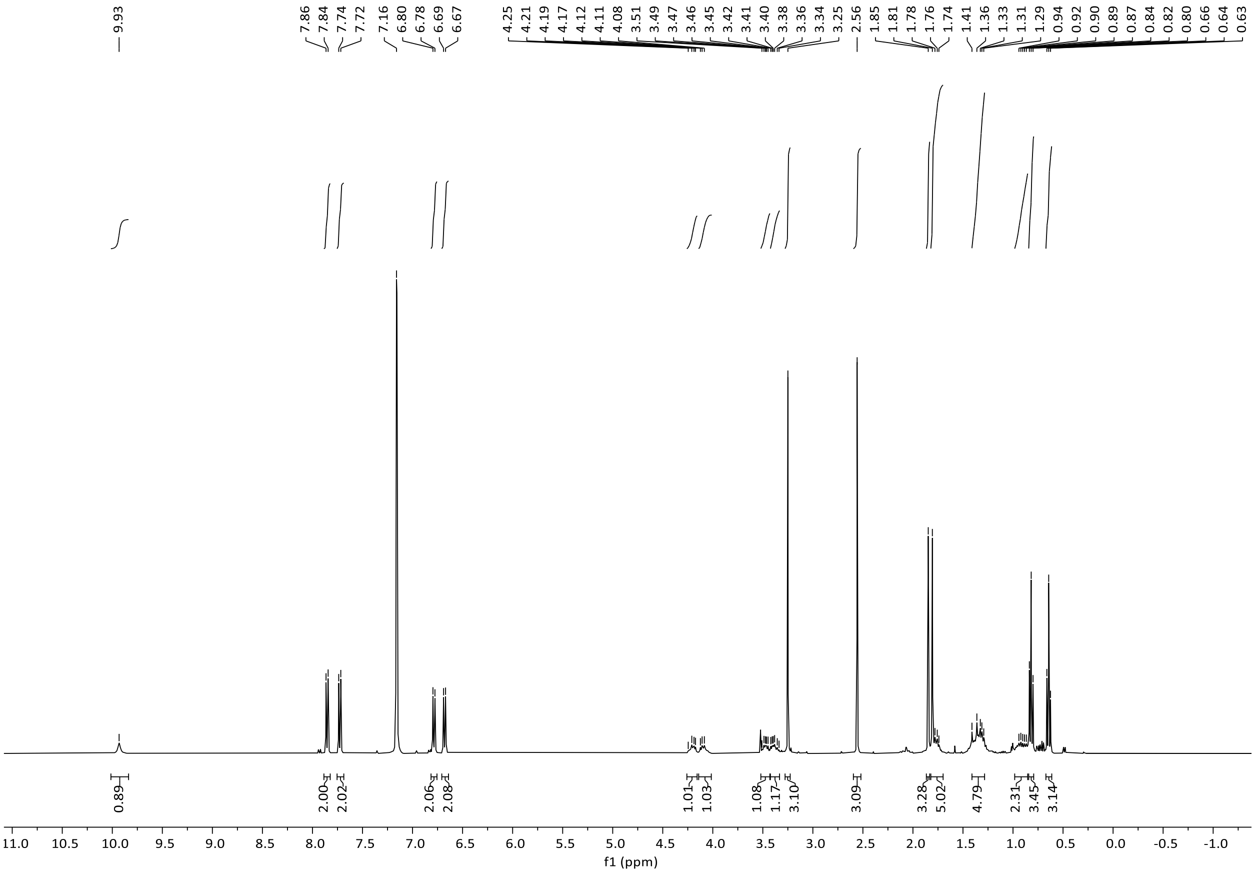

^{13}C NMR (101 MHz, C_6D_6)

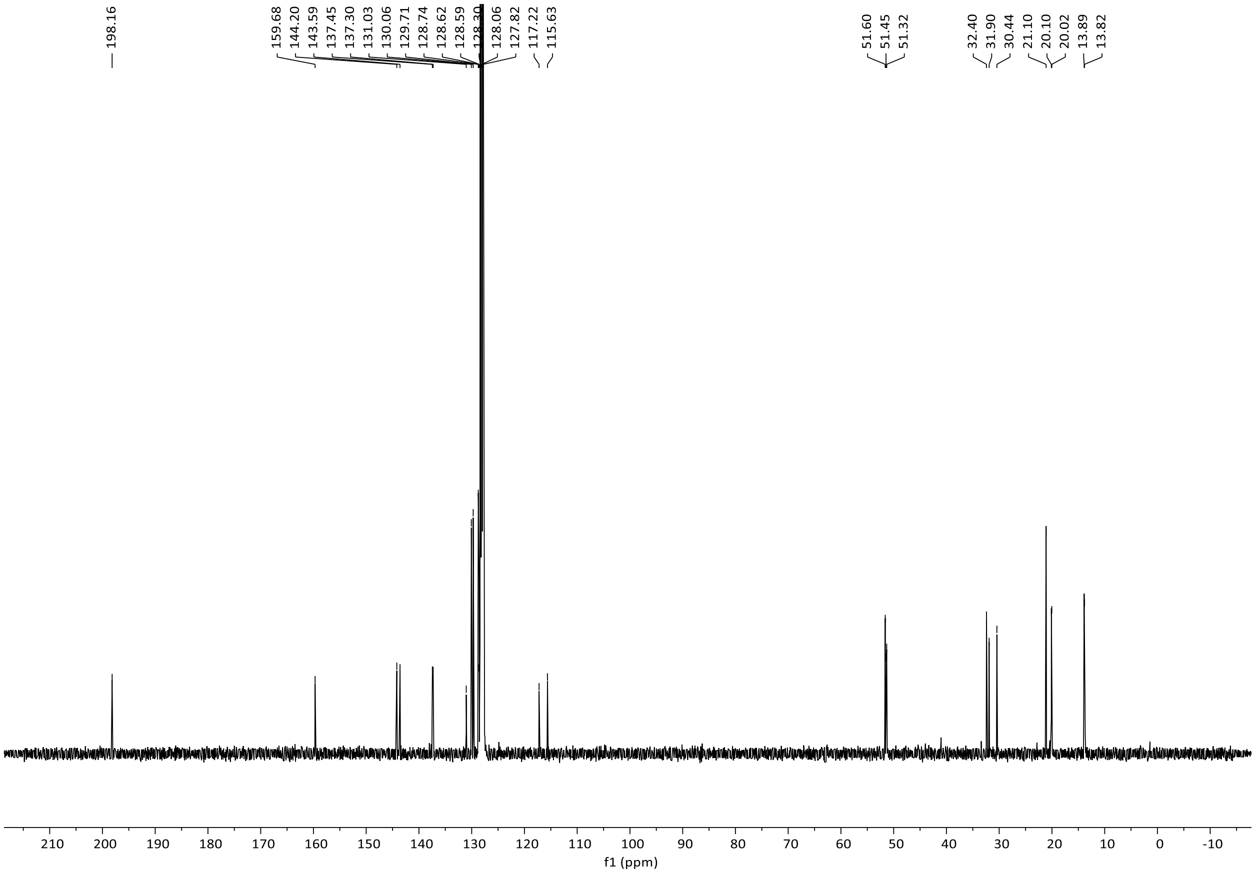

***N*,*N'*-(4-Benzoyl-5-phenyl-1*H*-pyrrole-2,3-diyl)bis(*N*-butyl-4-methylbenzenesulfonamide), 2g**

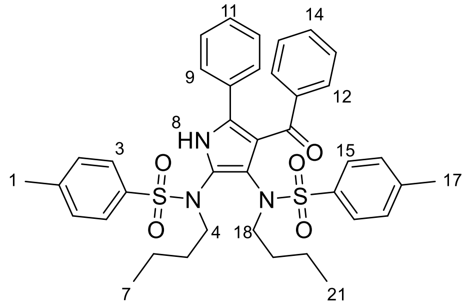

^{1}H NMR (400 MHz, $CDCl_3$)

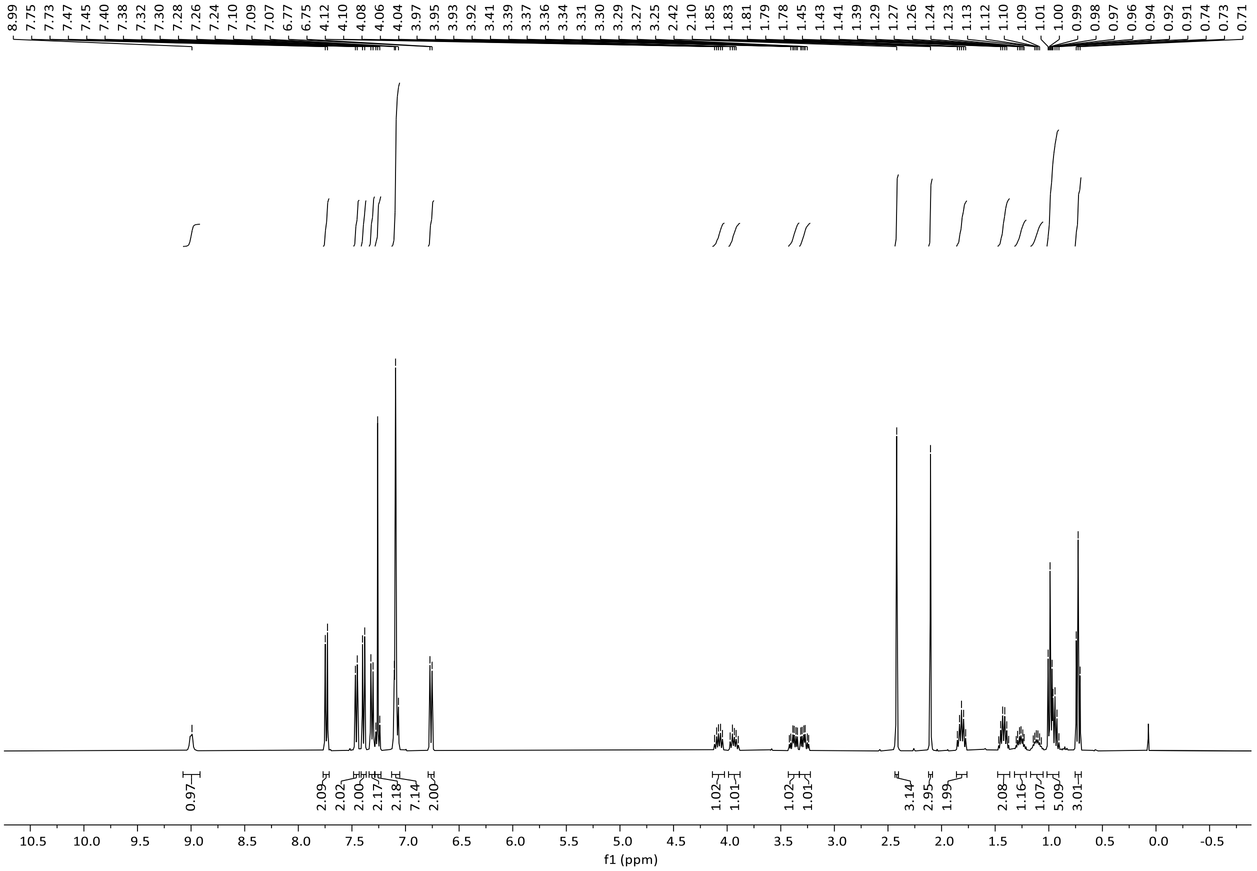

^{13}C NMR (101 MHz, $CDCl_3$)

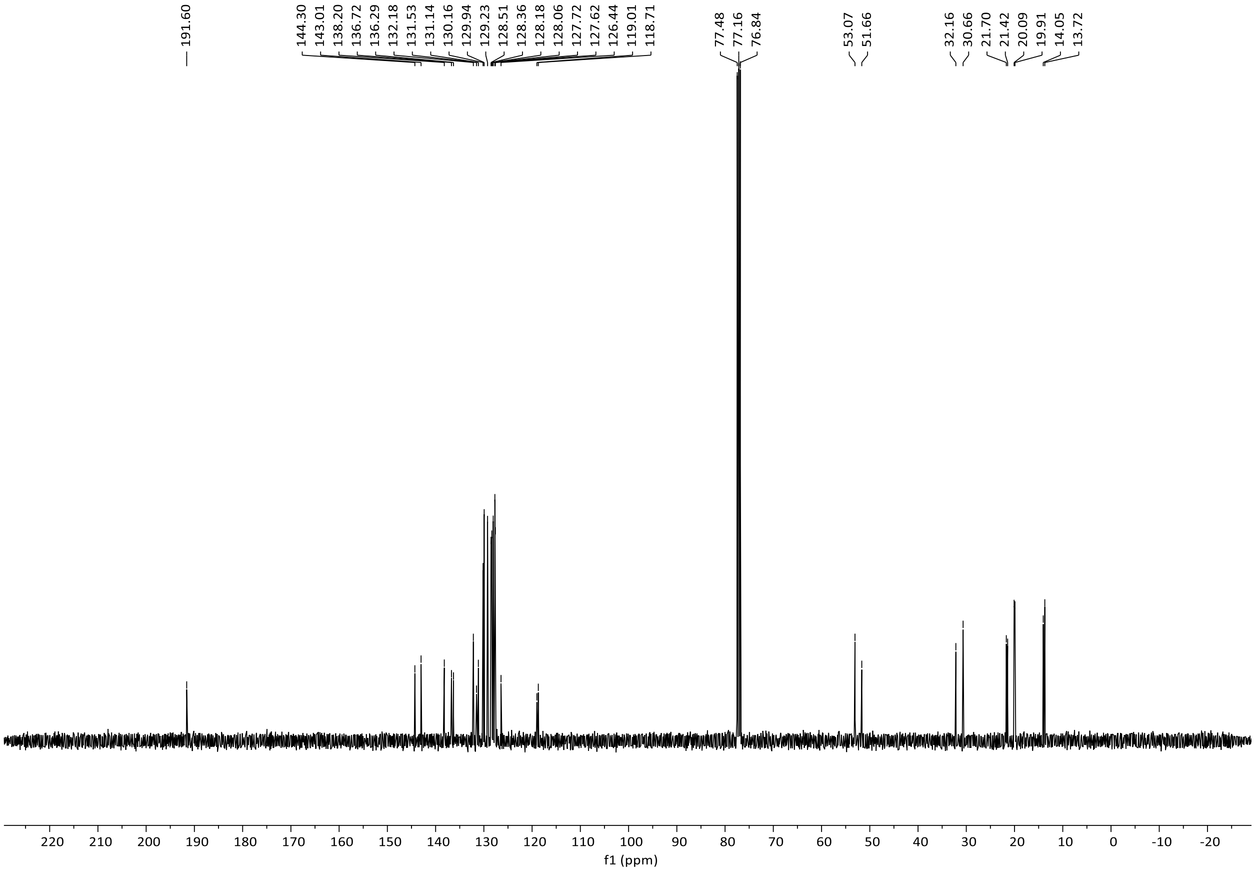

_N_,_N'_-(4-Pentanoyl-5-phenyl-1_H_-pyrrole-2,3-diyl)bis(_N_-butyl-4-methylbenzenesulfonamide), 2h

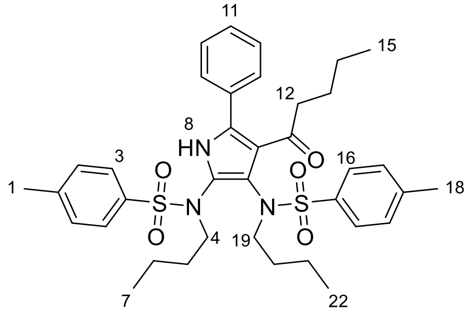

^{1}H NMR (400 MHz, $CDCl_3$)

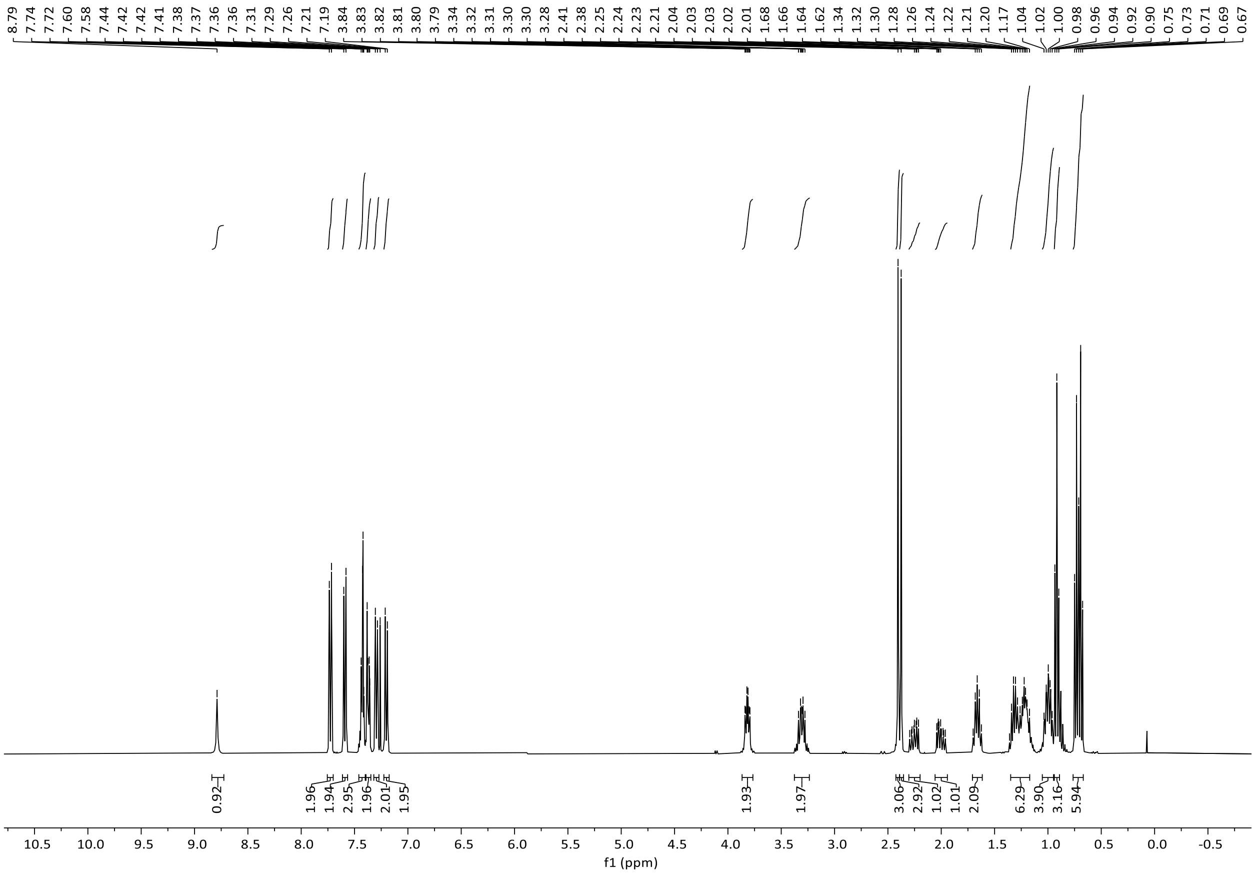

^{13}C NMR (101 MHz, $CDCl_3$)

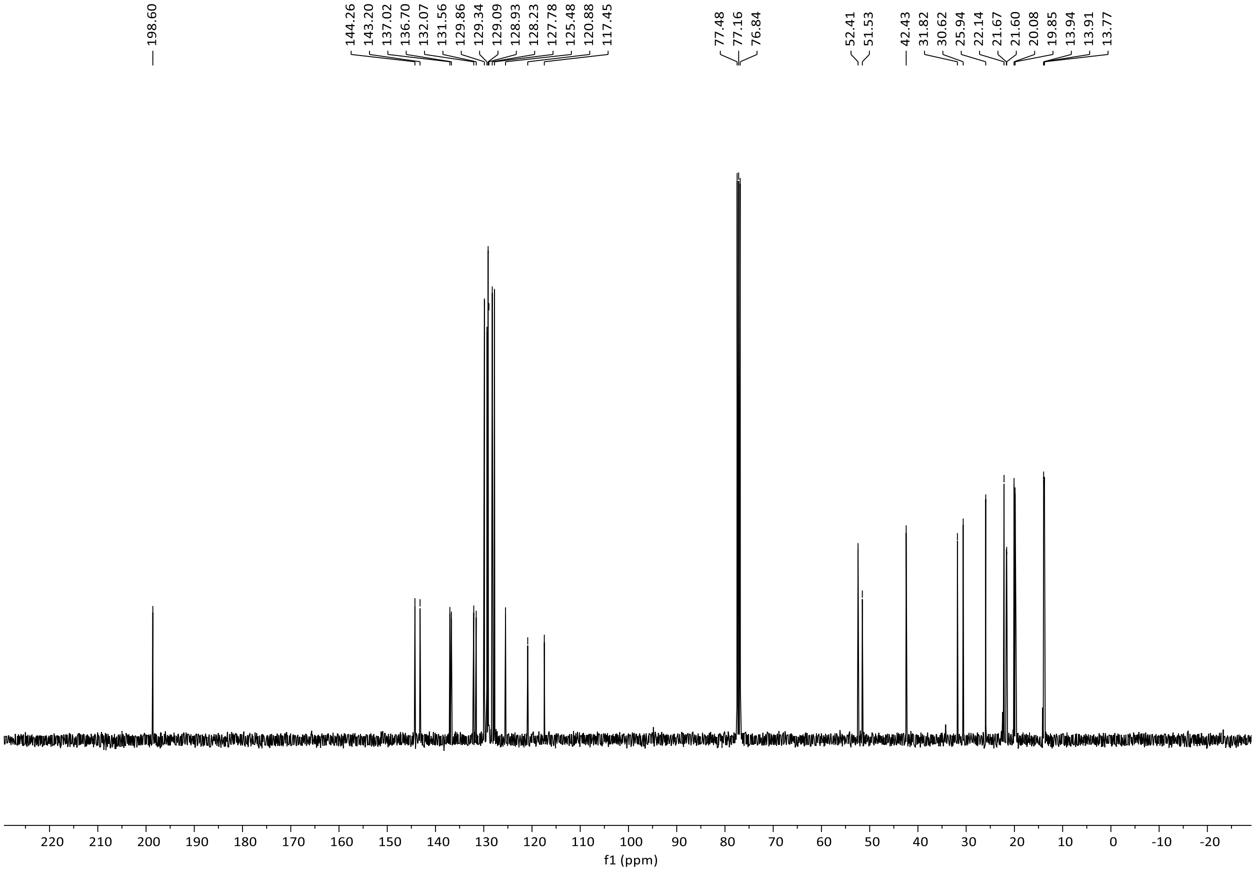

***N*,*N'*-(4-(Cyclohexanecarbonyl)-5-phenyl-1*H*-pyrrole-2,3-diyl)bis(*N*-butyl-4-methylbenzenesulfonamide), 2i**

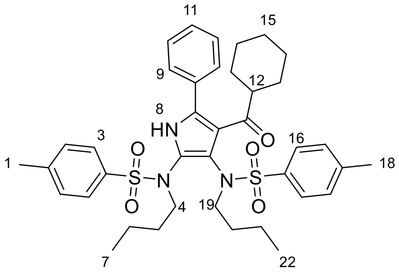

^{1}H NMR (400 MHz, $CDCl_3$)

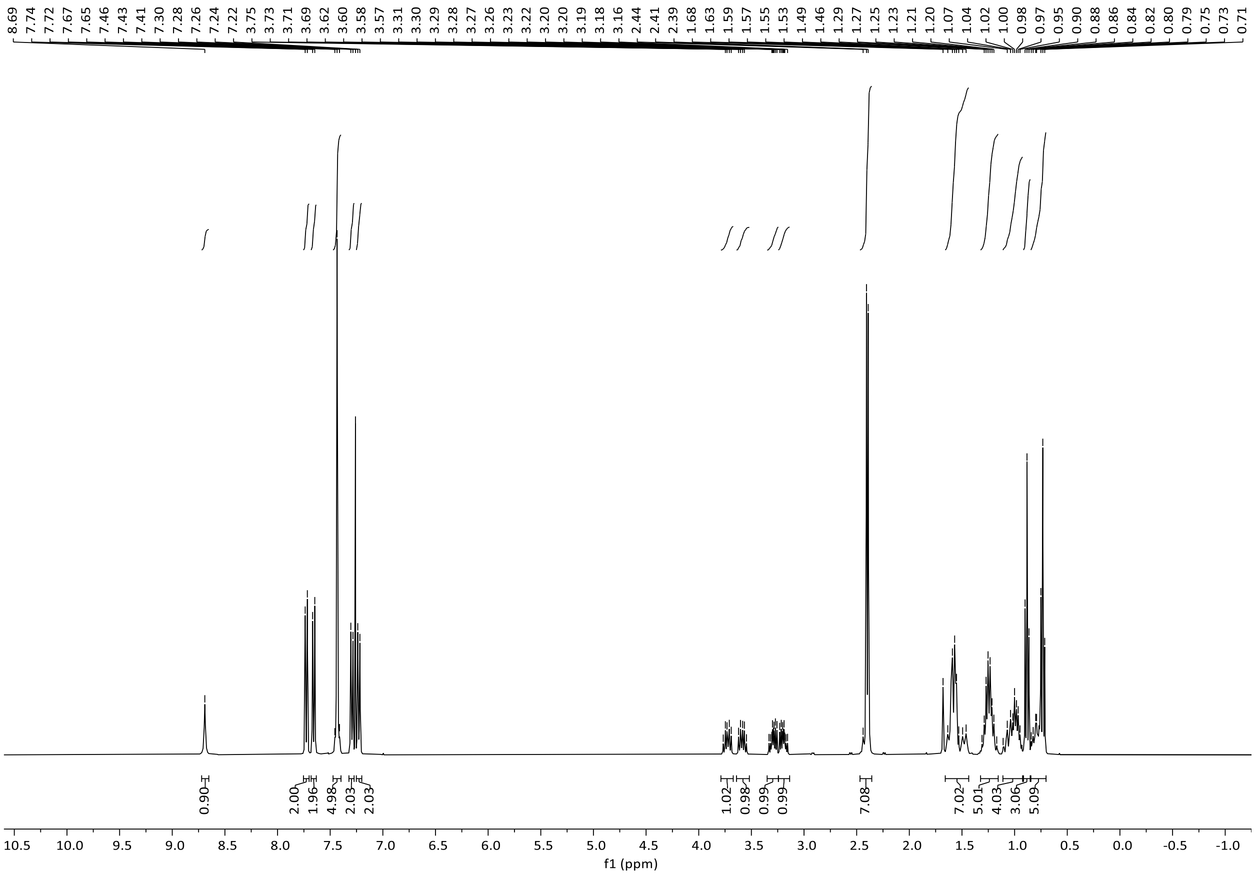

^{13}C NMR (101 MHz, $CDCl_3$)

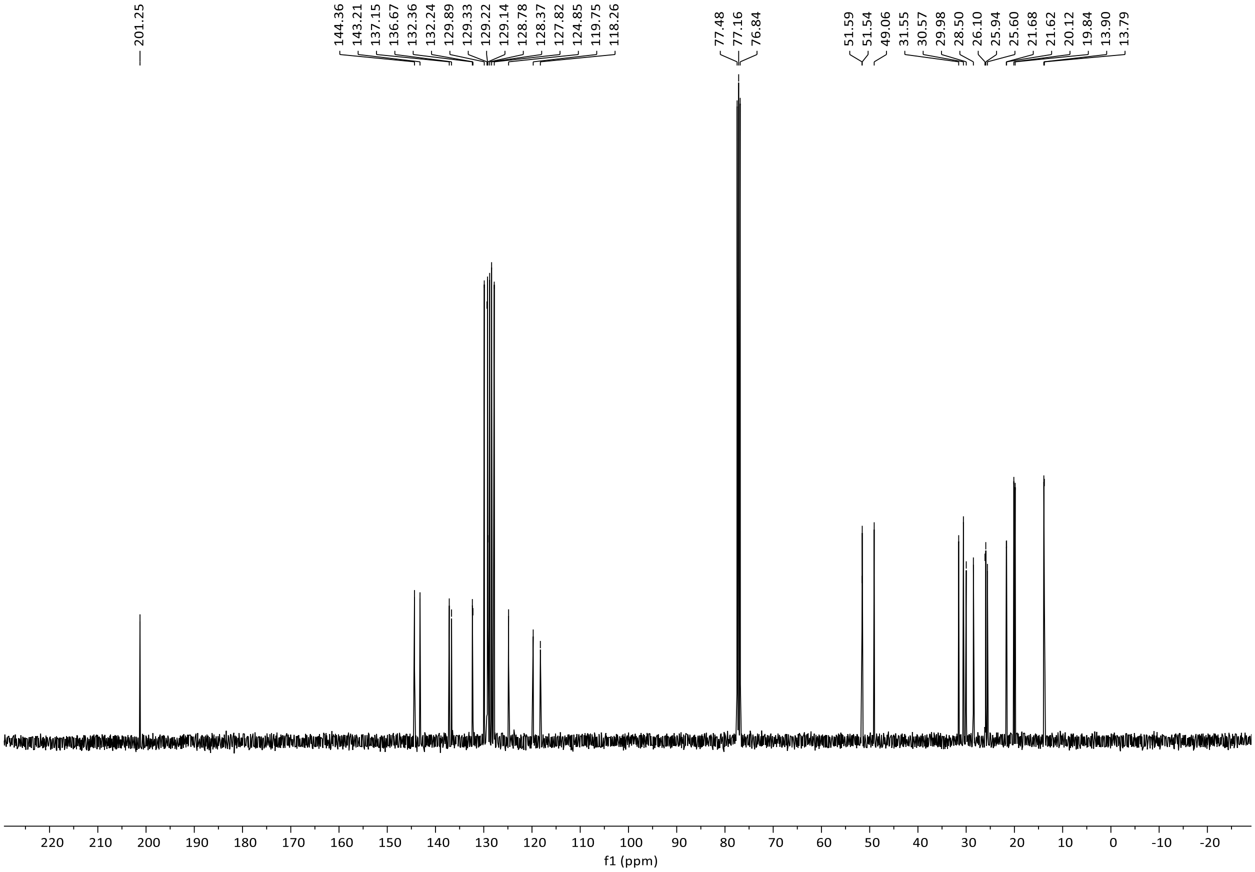

***N*,*N'*-(4-(Cyclohex-1-ene-1-carbonyl)-5-phenyl-1*H*-pyrrole-2,3-diyl)bis(*N*-butyl-4-methylbenzenesulfonamide), 2j**

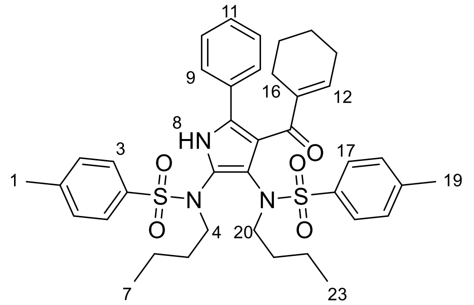

^{1}H NMR (400 MHz, $CDCl_3$)

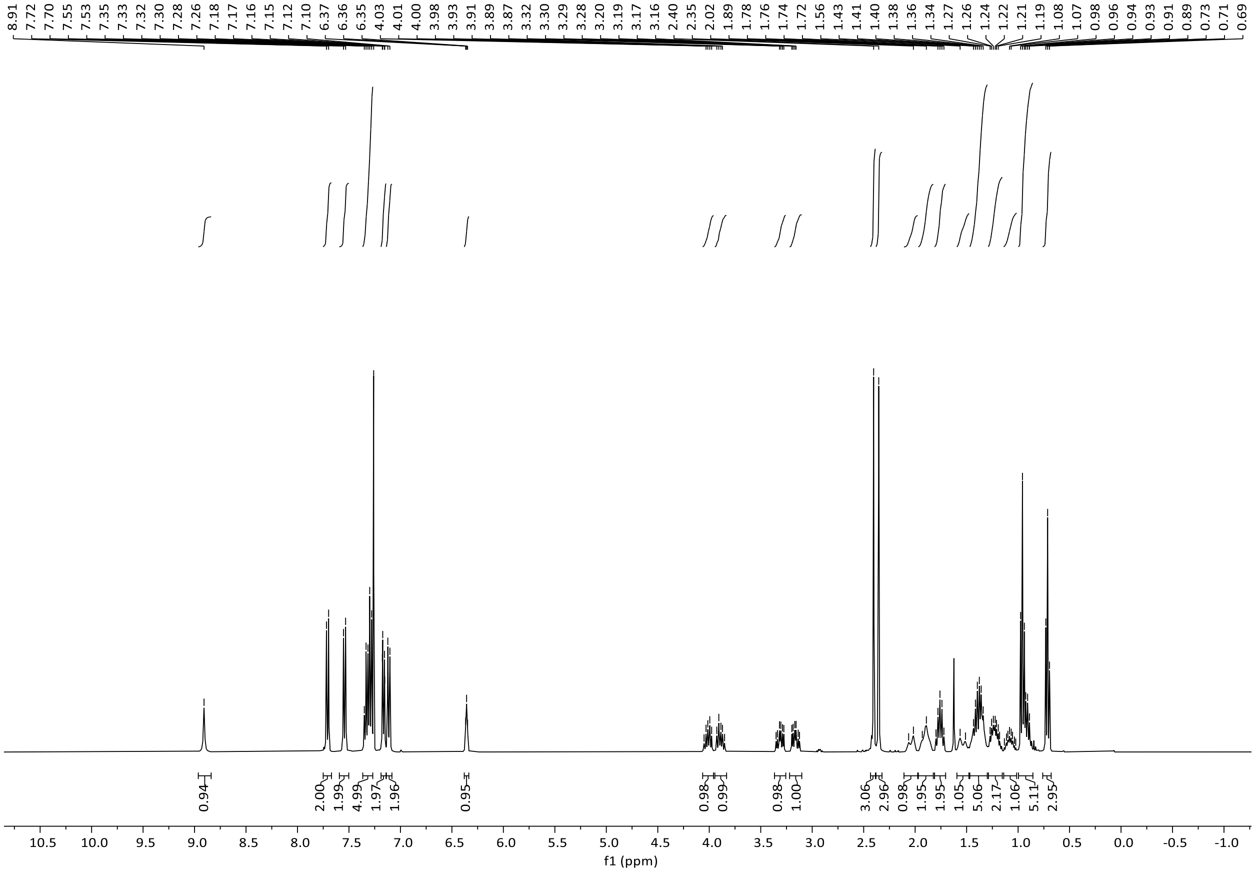

^{13}C NMR (101 MHz, $CDCl_3$)

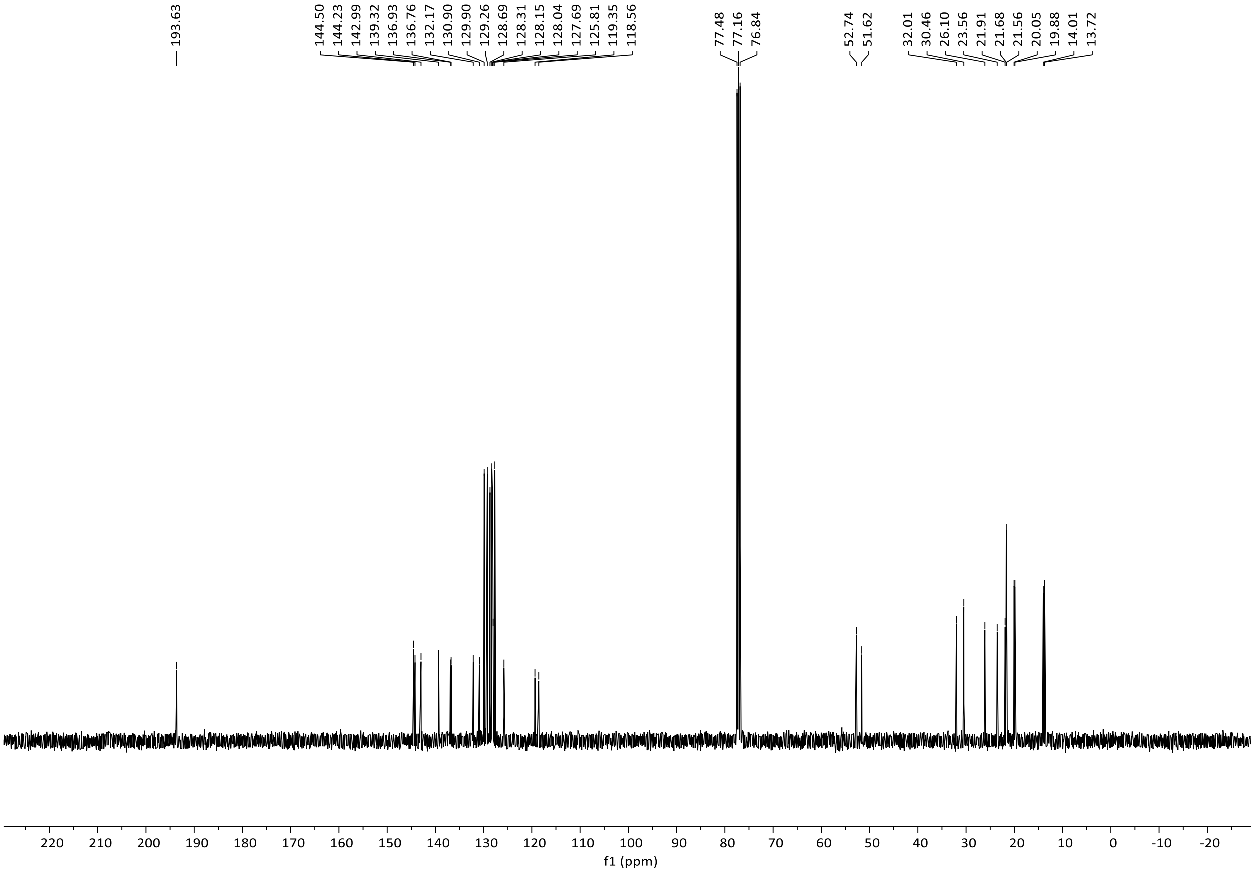

***N*,*N'*-(4-(4-Fluorobenzoyl)-5-phenyl-1*H*-pyrrole-2,3-diyl)bis(*N*-butyl-4-methylbenzenesulfonamide), 2k**

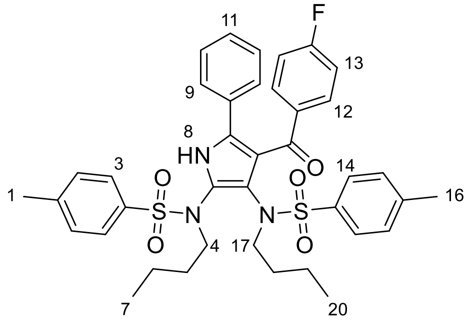

^{1}H NMR (500 MHz, $CDCl_3$)

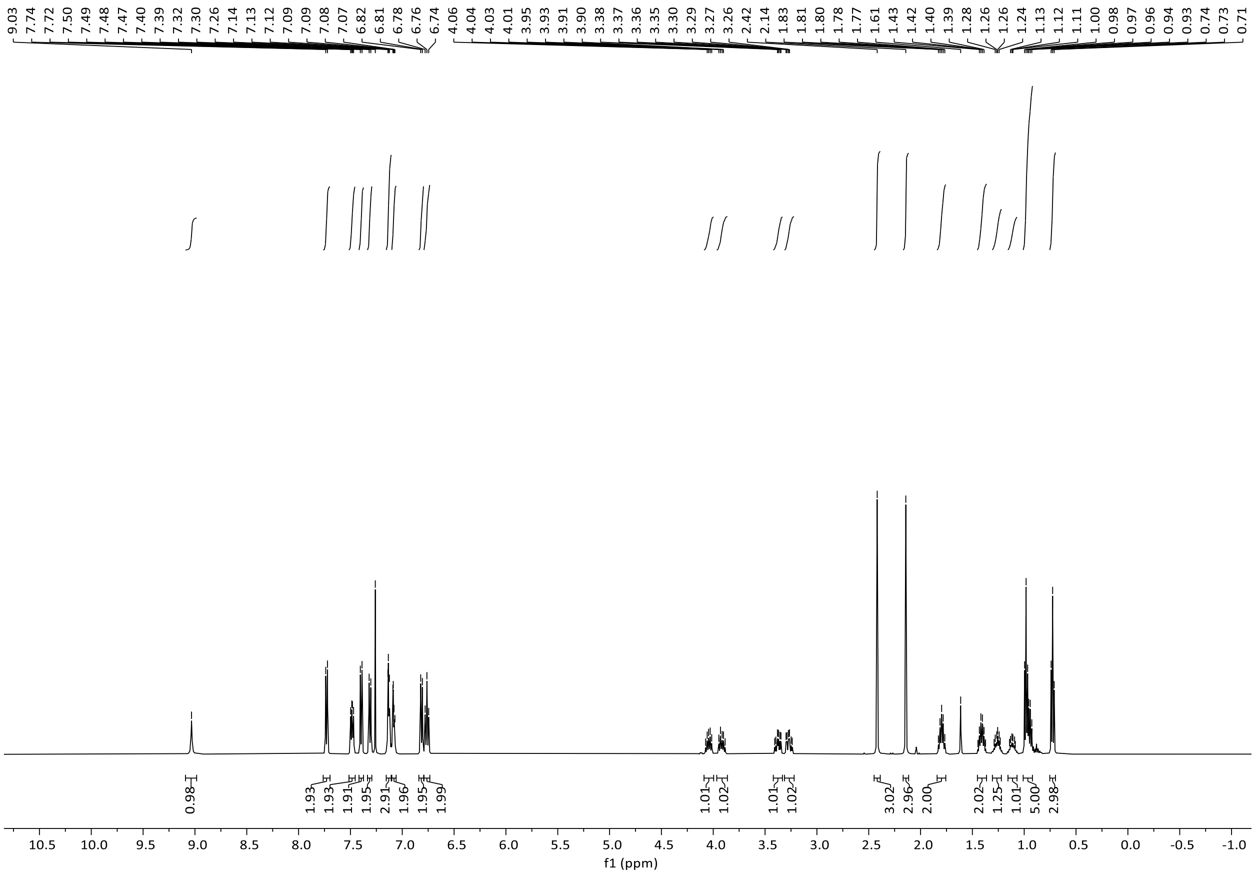

^{13}C NMR {^{19}F} (126 MHz, CDCl$_3$)

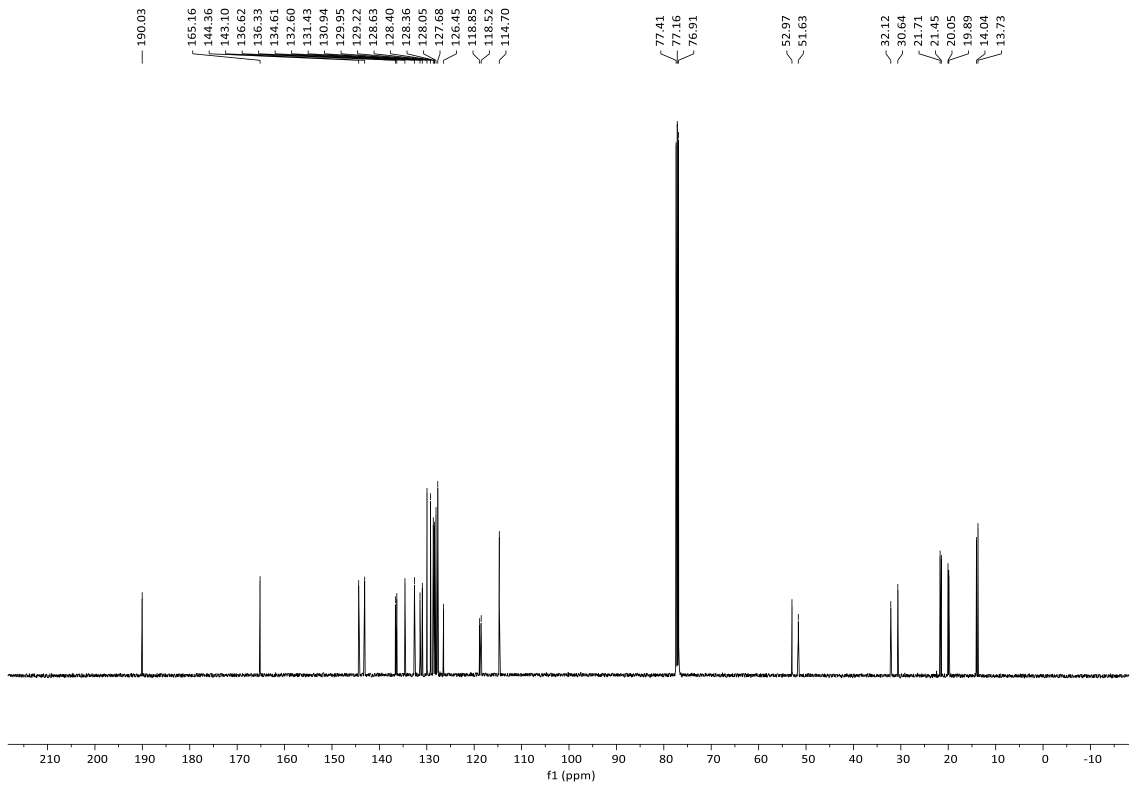

^{19}F NMR (471 MHz, CDCl$_3$)

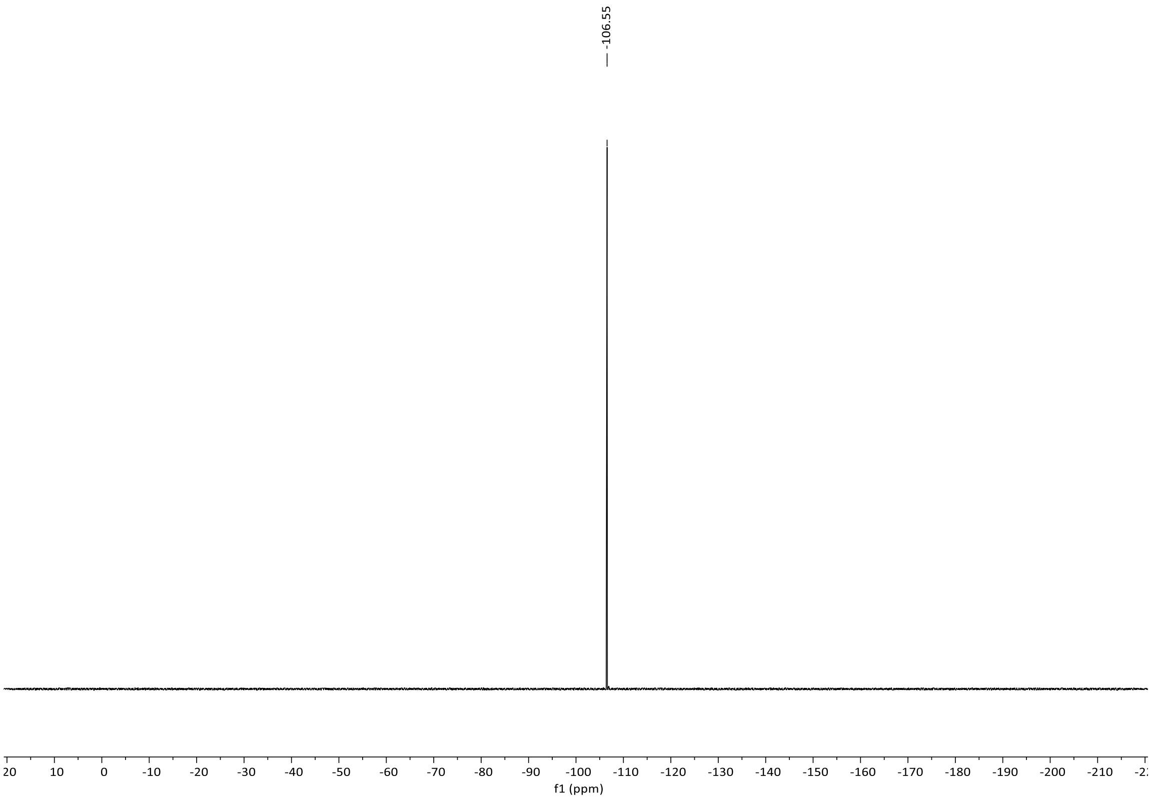

***N*,*N'*-(4-(4-Cyanobenzoyl)-5-phenyl-1*H*-pyrrole-2,3-diyl)bis(*N*-butyl-4-methylbenzenesulfonamide), 2l**

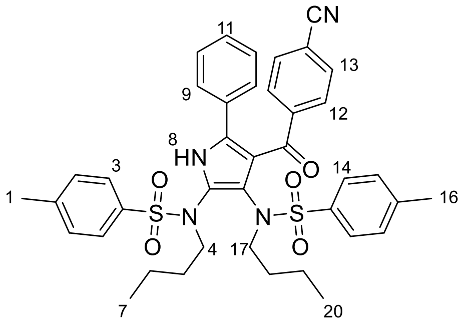

^{1}H NMR (500 MHz, $CDCl_3$)

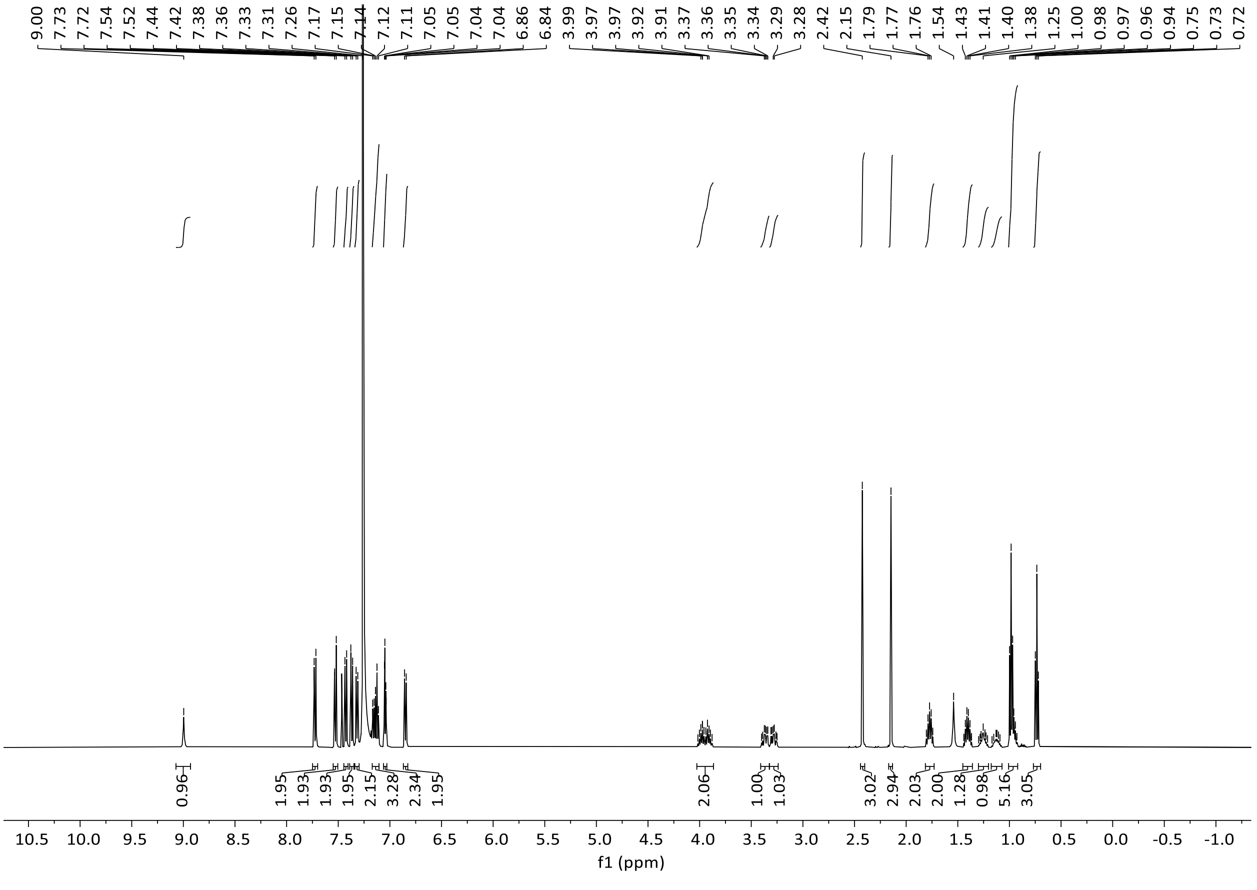

^{13}C NMR (126 MHz, $CDCl_3$)

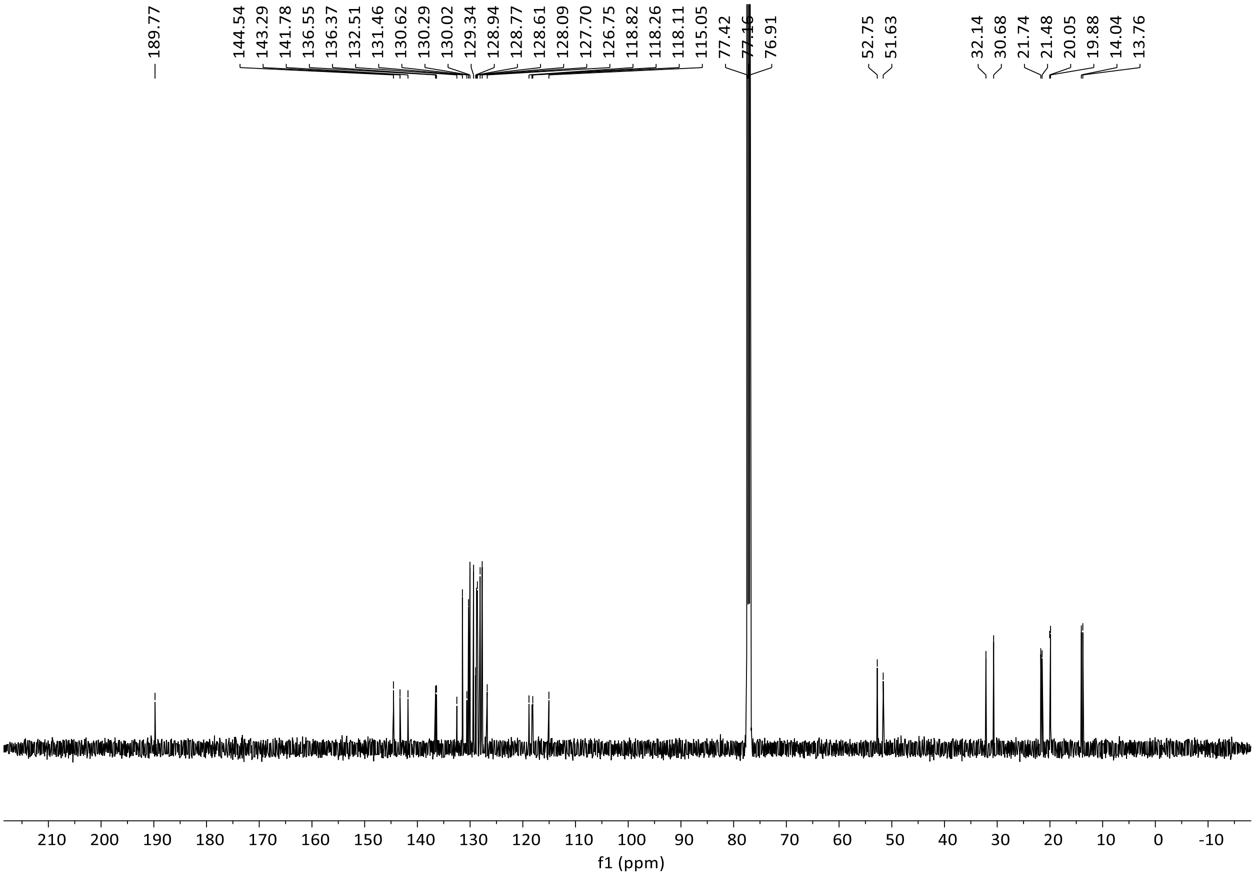

***N*,*N'*-(4-(4-Methoxybenzoyl)-5-phenyl-1*H*-pyrrole-2,3-diyl)bis(*N*-butyl-4-methylbenzenesulfonamide), 2m**

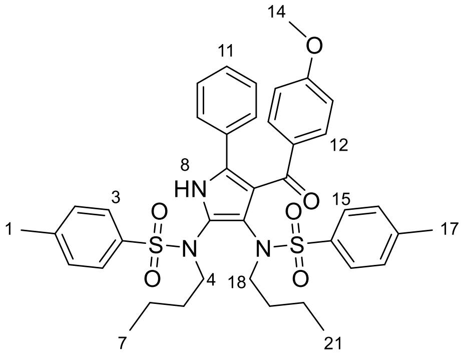

^{1}H NMR (400 MHz, $CDCl_3$)

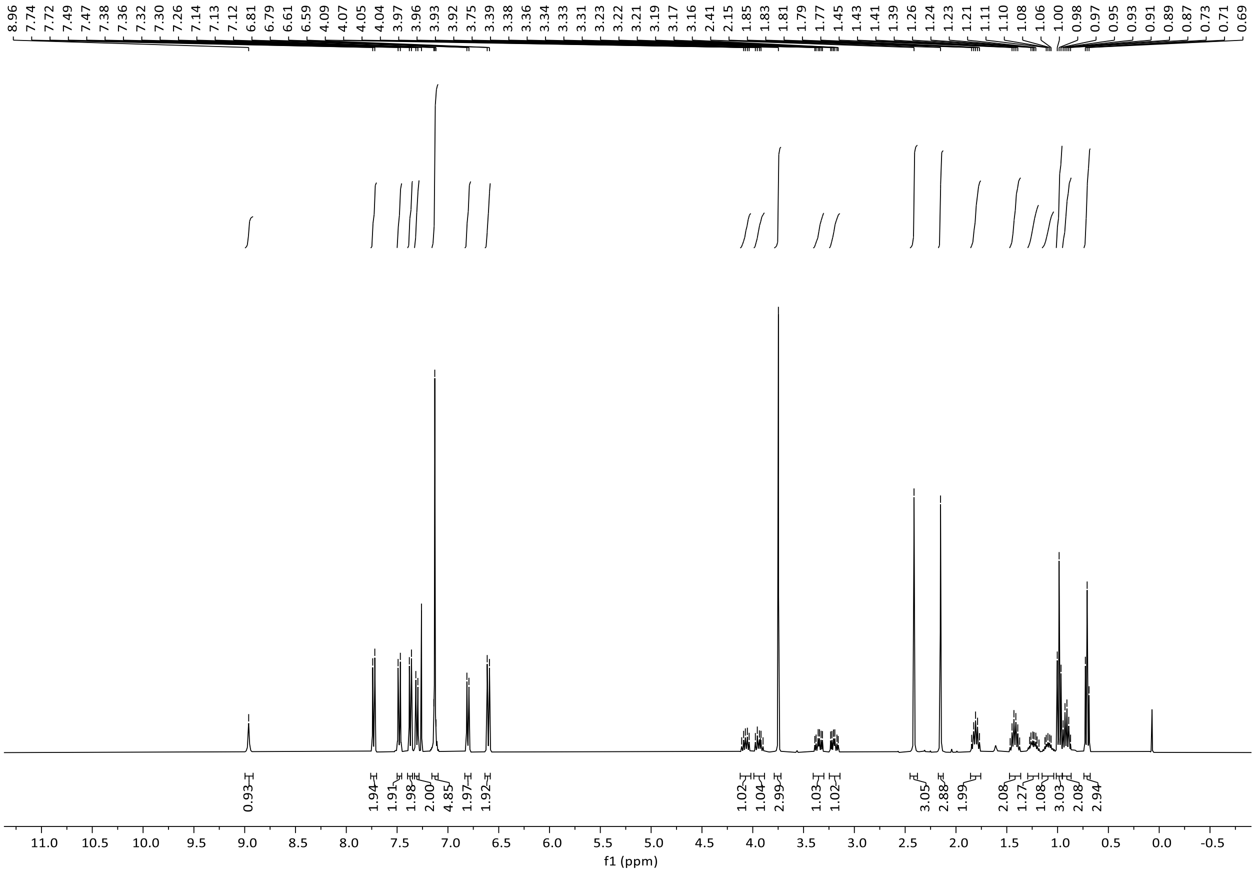

^{13}C NMR (101 MHz, $CDCl_3$)

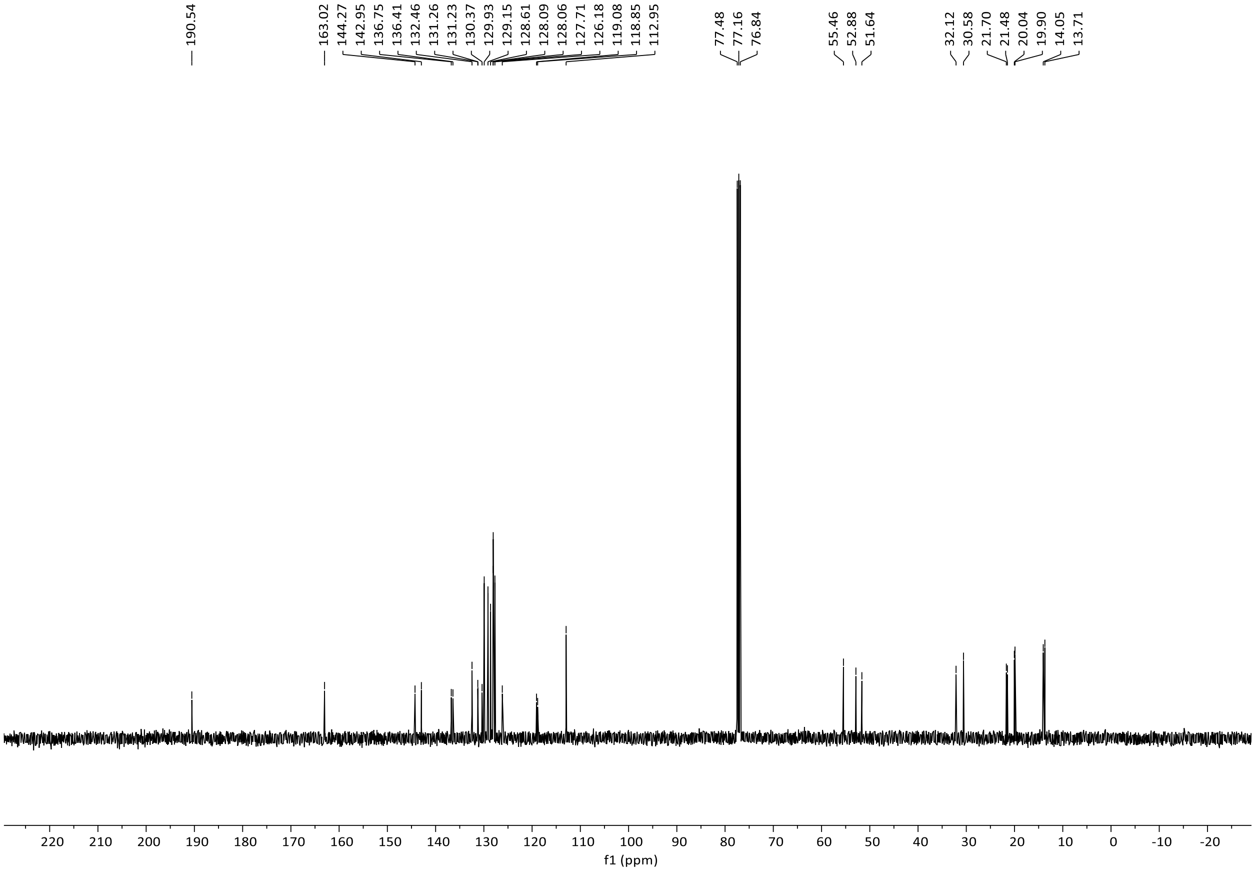

N,N'-(4-Benzoyl-5-methyl-1H-pyrrole-2,3-diyl)bis(N-butyl-4-methylbenzenesulfonamide), 2n

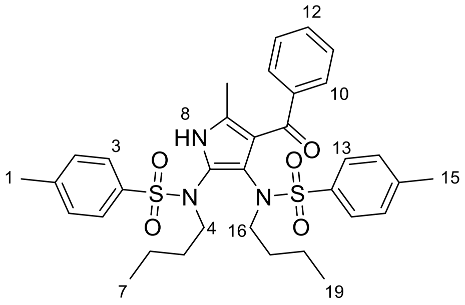

^{1}H NMR (400 MHz, $CDCl_3$)

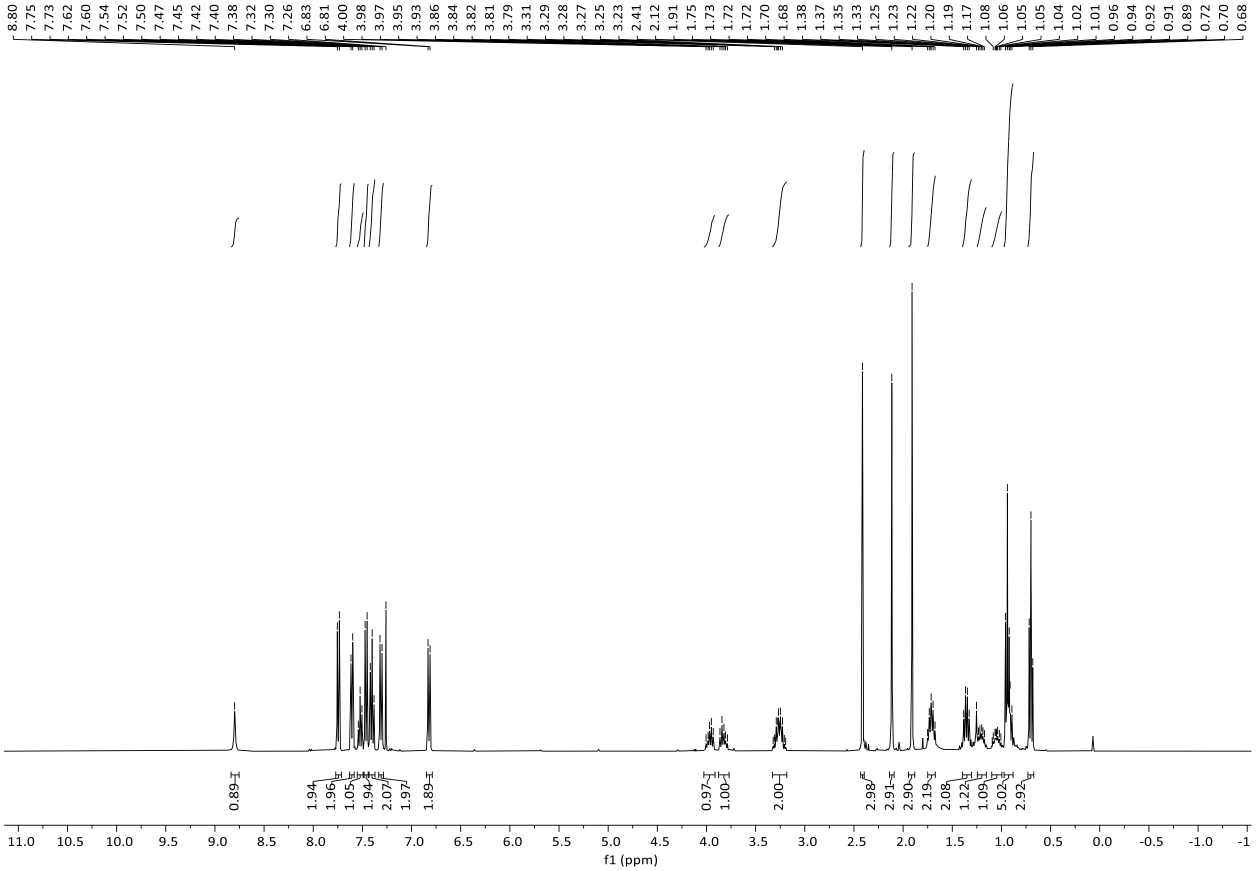

^{13}C NMR (101 MHz, $CDCl_3$)

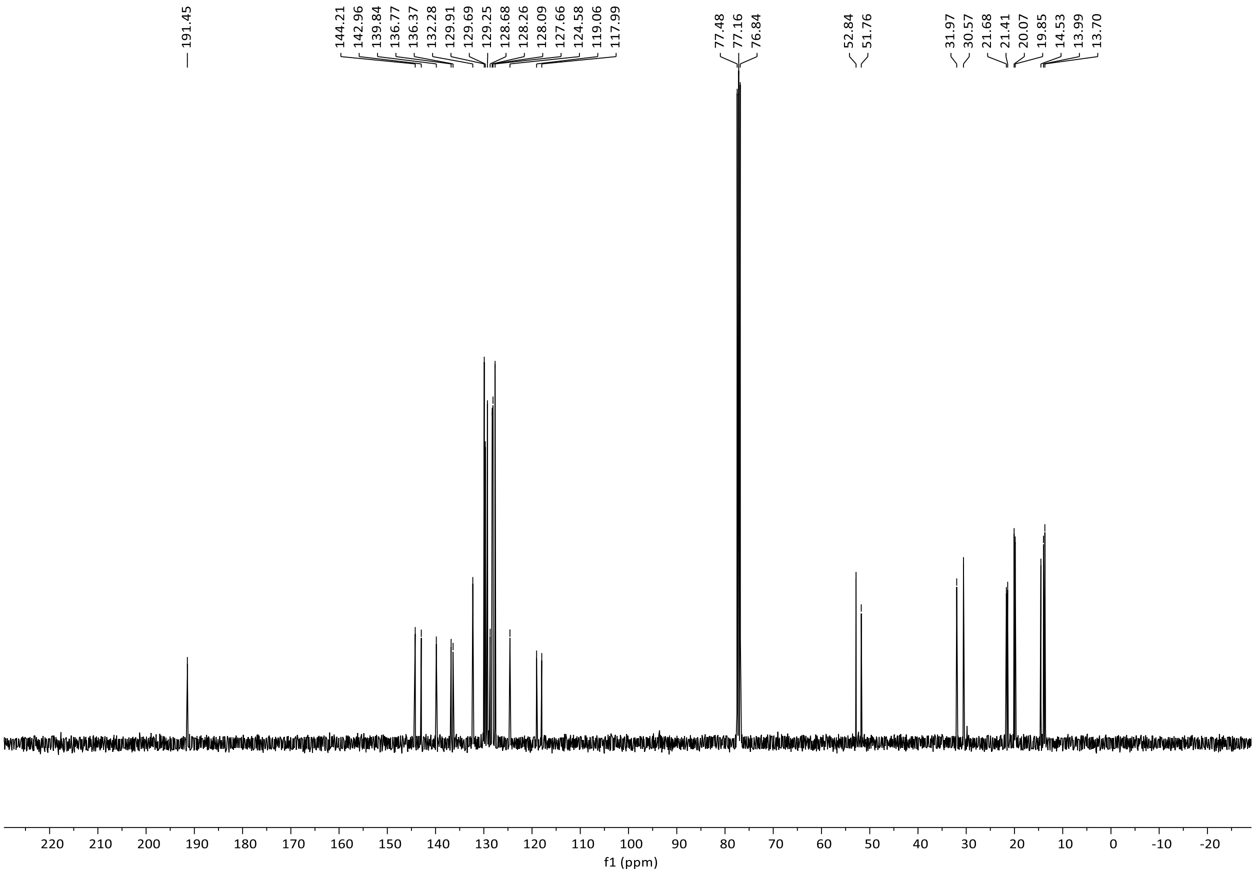

***N*,*N'*-(4-Benzoyl-5-(tert-butyl)-1*H*-pyrrole-2,3-diyl)bis(*N*-butyl-4-methylbenzenesulfonamide), 2o**

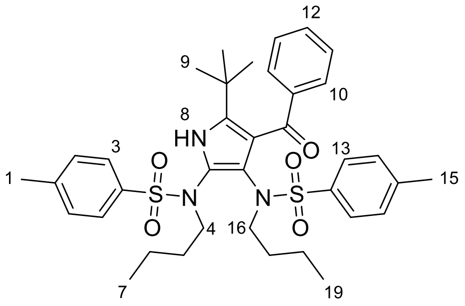

^{1}H NMR (500 MHz, $CDCl_3$)

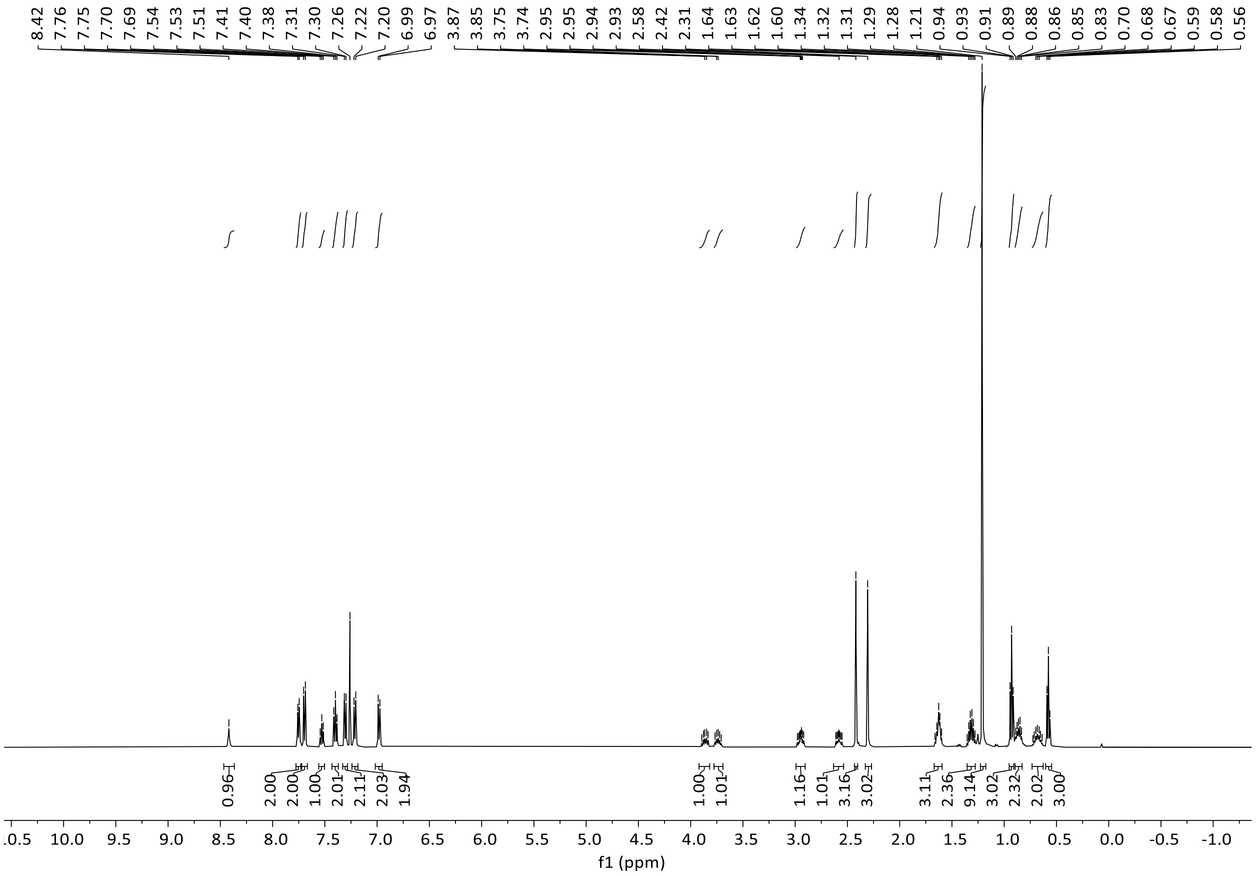

^{13}C NMR (126 MHz, $CDCl_3$)

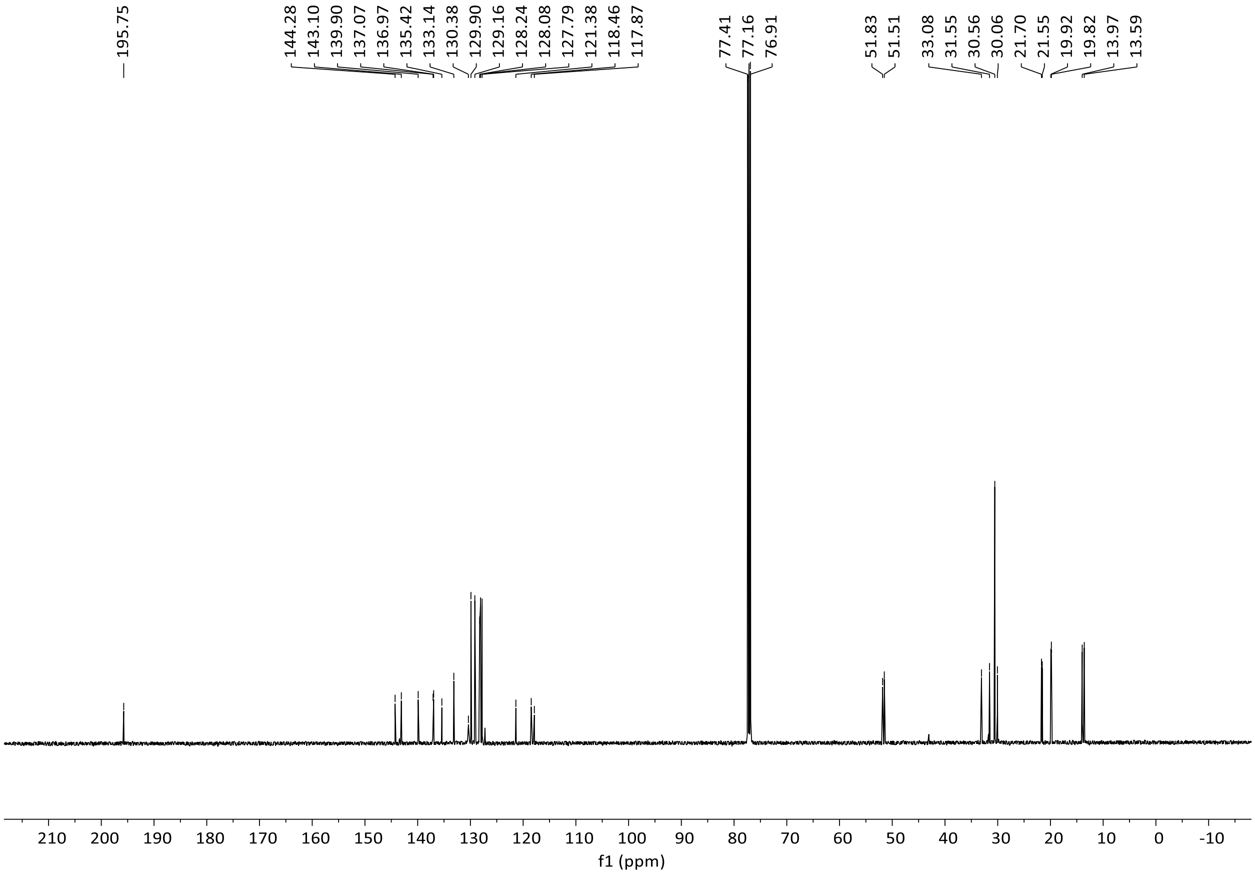

***N*,*N'*-(4-Benzoyl-5-(4-fluorophenyl)-1*H*-pyrrole-2,3-diyl)bis(*N*-butyl-4-methylbenzenesulfonamide), 2p**

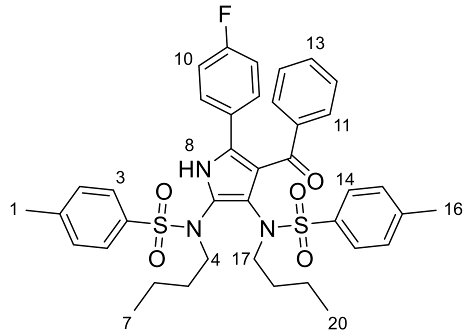

^{1}H NMR (400 MHz, $CDCl_3$)

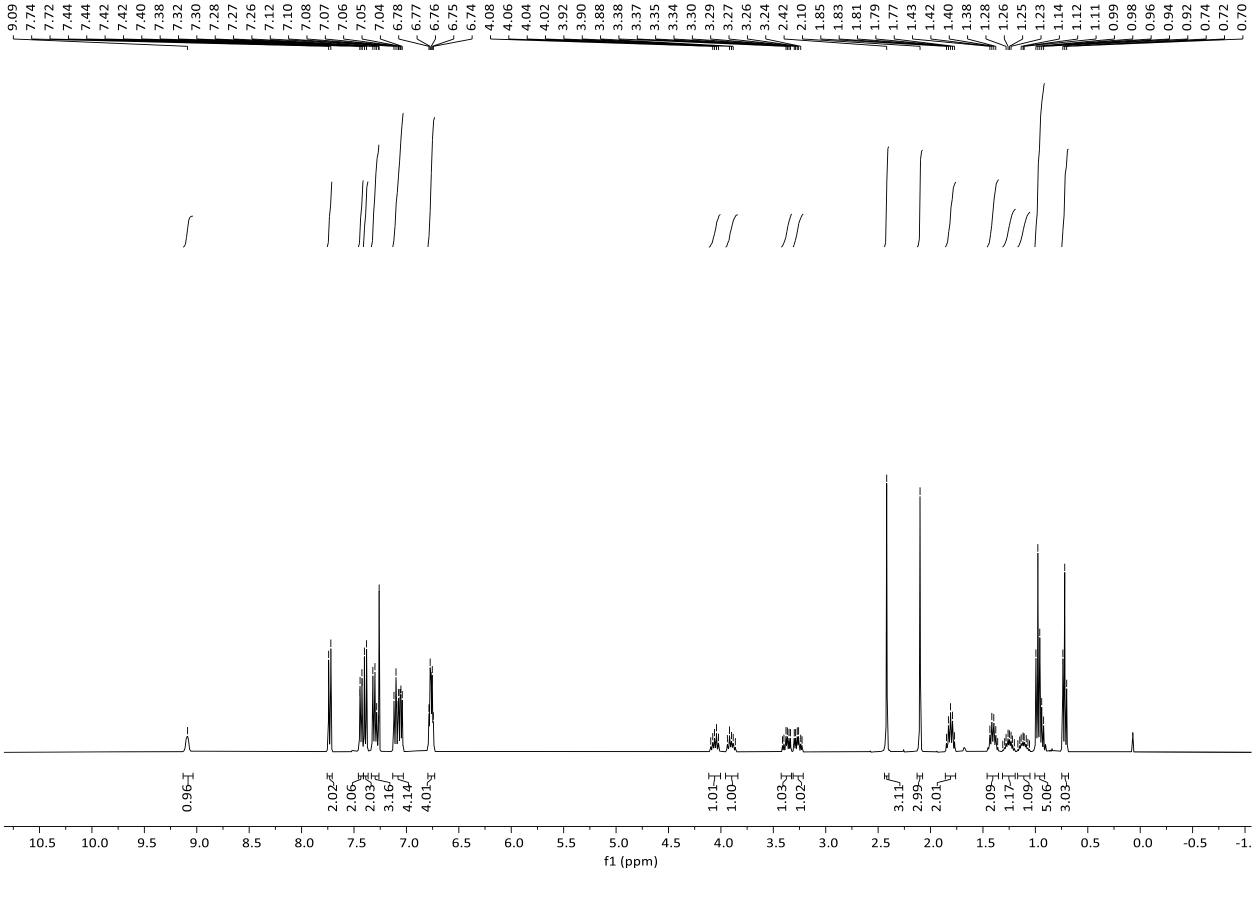

^{13}C NMR (101 MHz, $CDCl_3$)

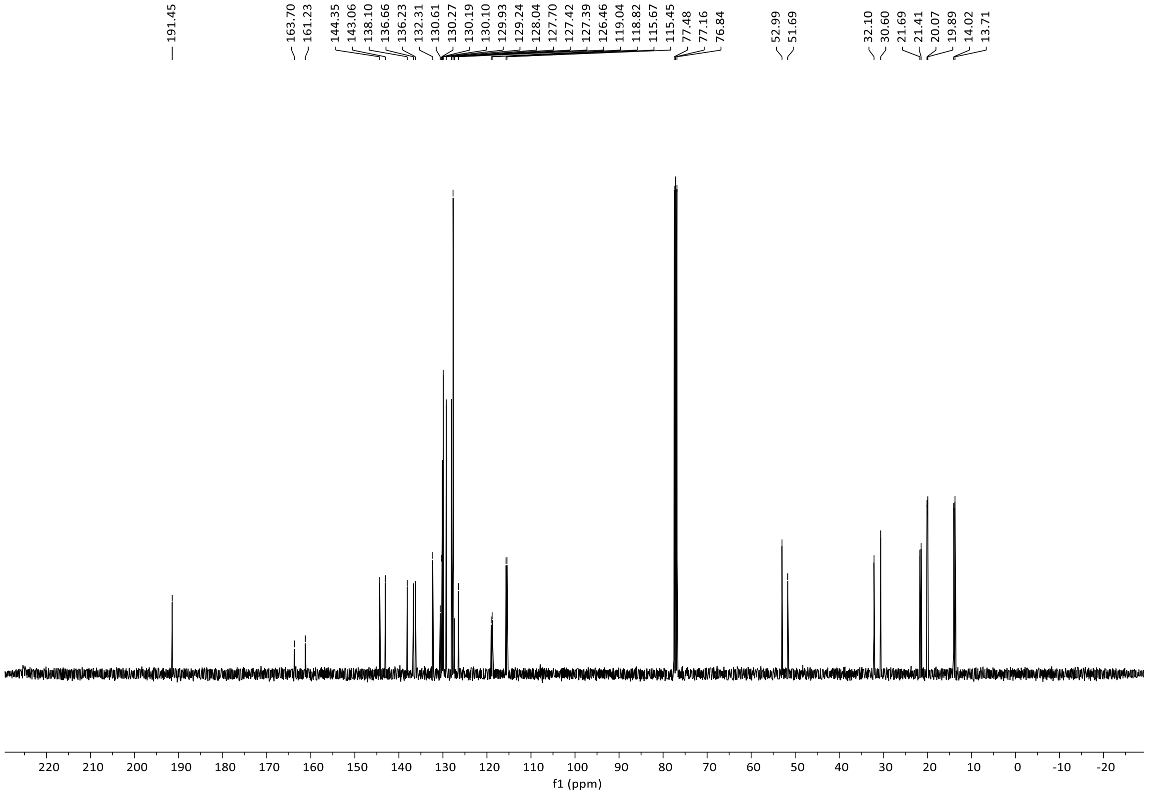

^{19}F NMR (377 MHz, $CDCl_3$)

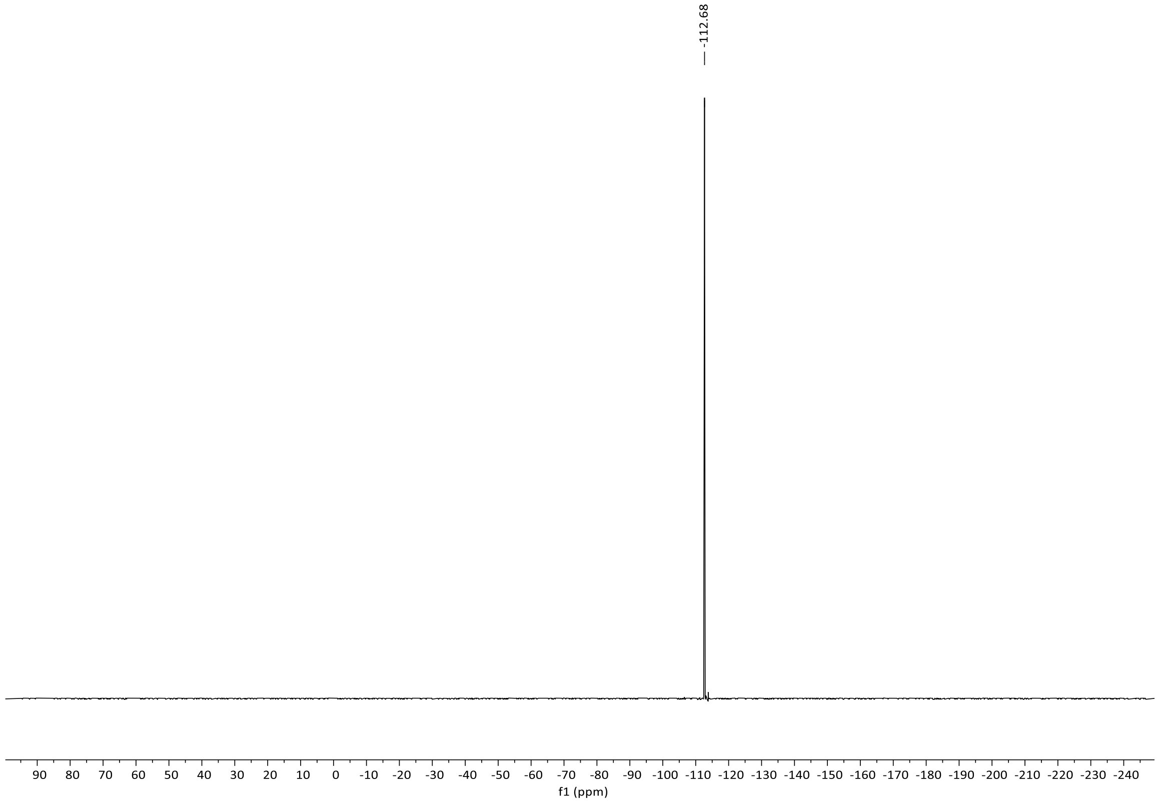

***N*,*N'*-(4-Benzoyl-5-(4-cyanophenyl)-1*H*-pyrrole-2,3-diyl)bis(*N*-butyl-4-methylbenzenesulfonamide), 2q**

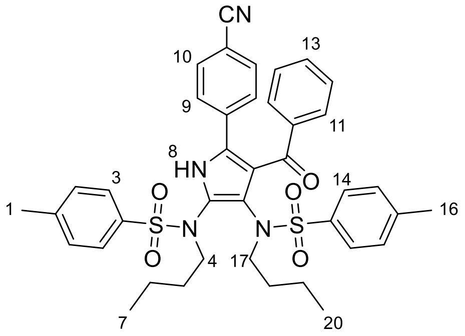

^{1}H NMR (500 MHz, $CDCl_3$)

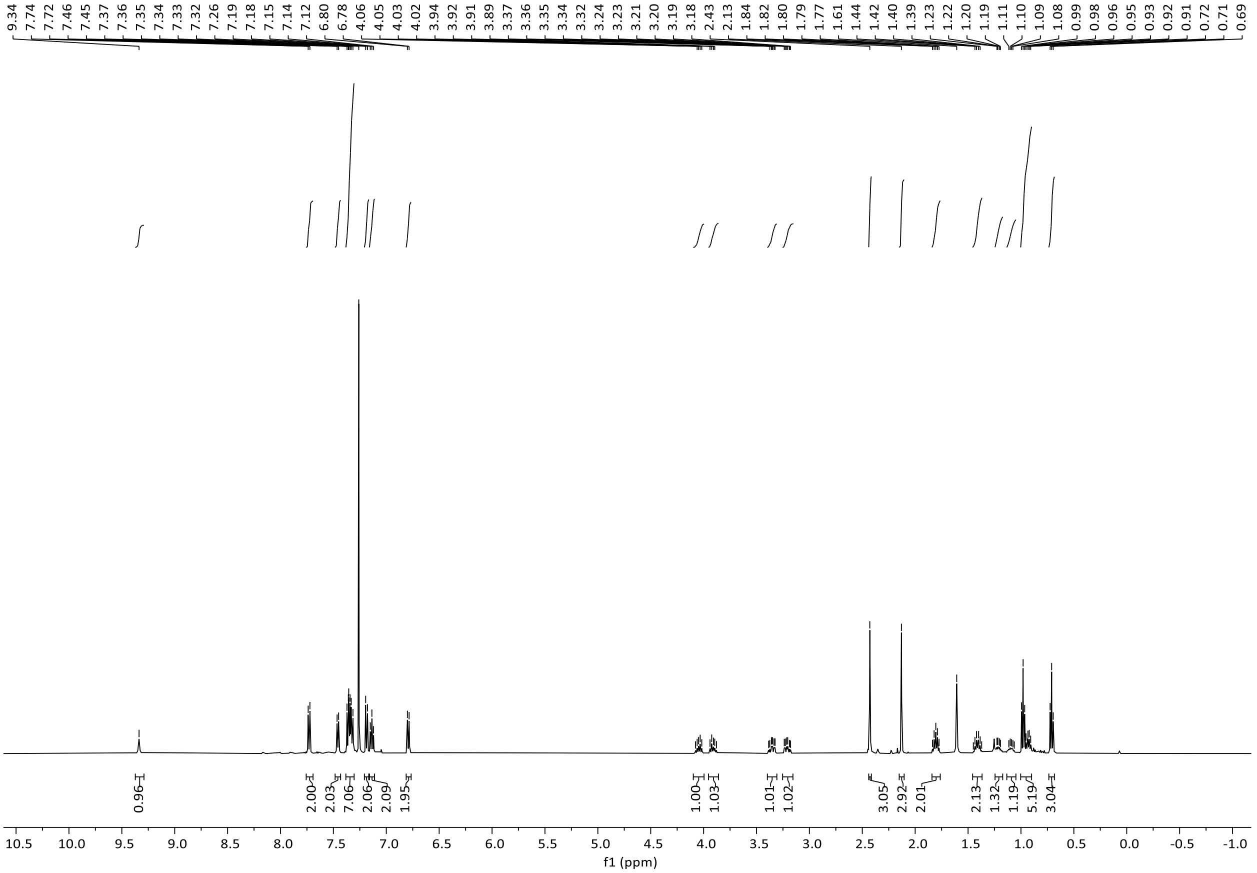

^{13}C NMR (126 MHz, $CDCl_3$)

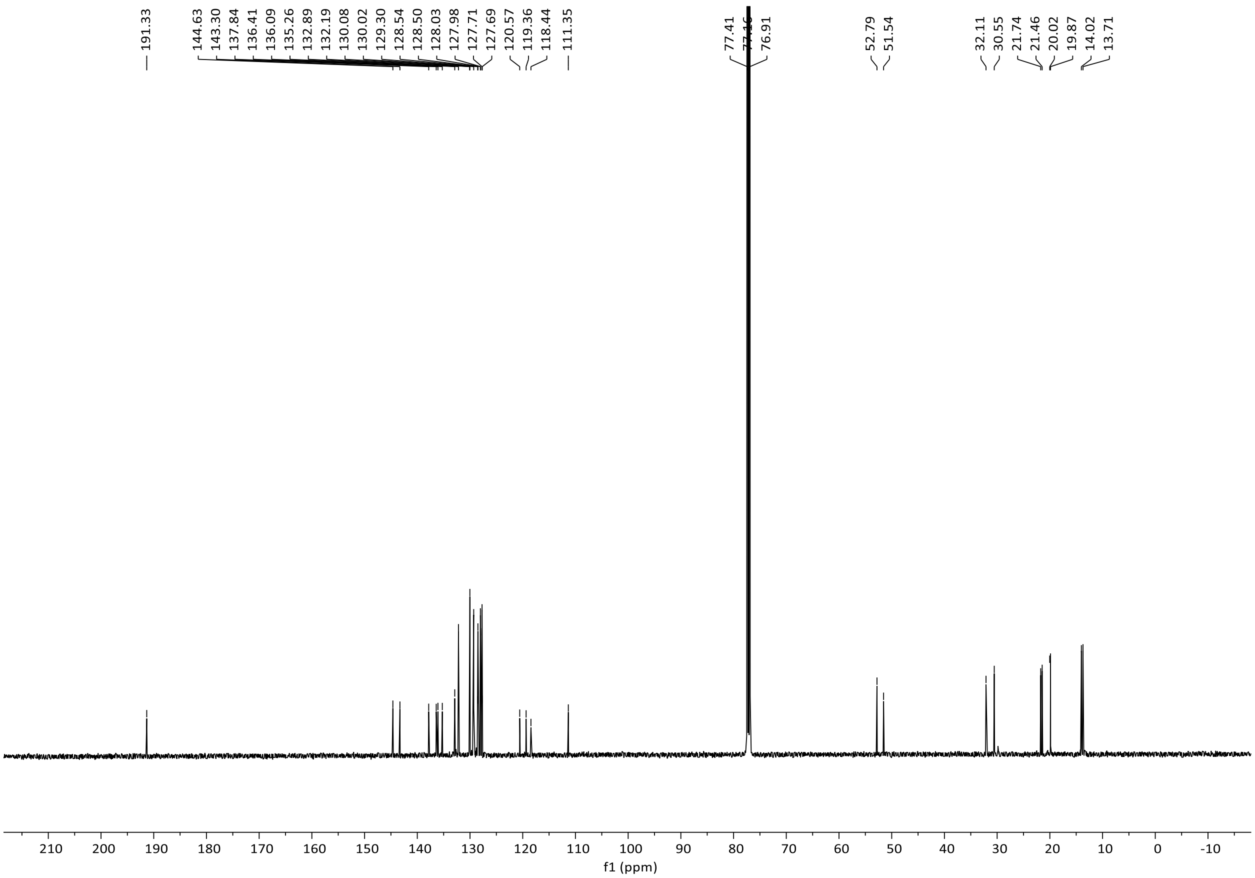

***N*,*N'*-(4-Benzoyl-5-(4-methoxyphenyl)-1*H*-pyrrole-2,3-diyl)bis(*N*-butyl-4-methylbenzenesulfonamide), 2r**

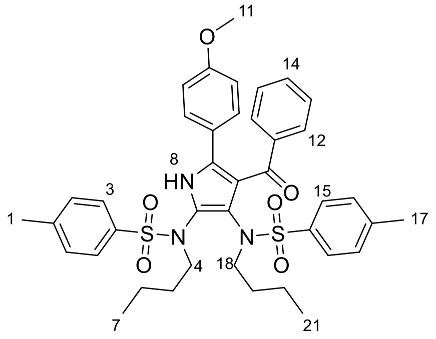

^{1}H NMR (400 MHz, $CDCl_3$)

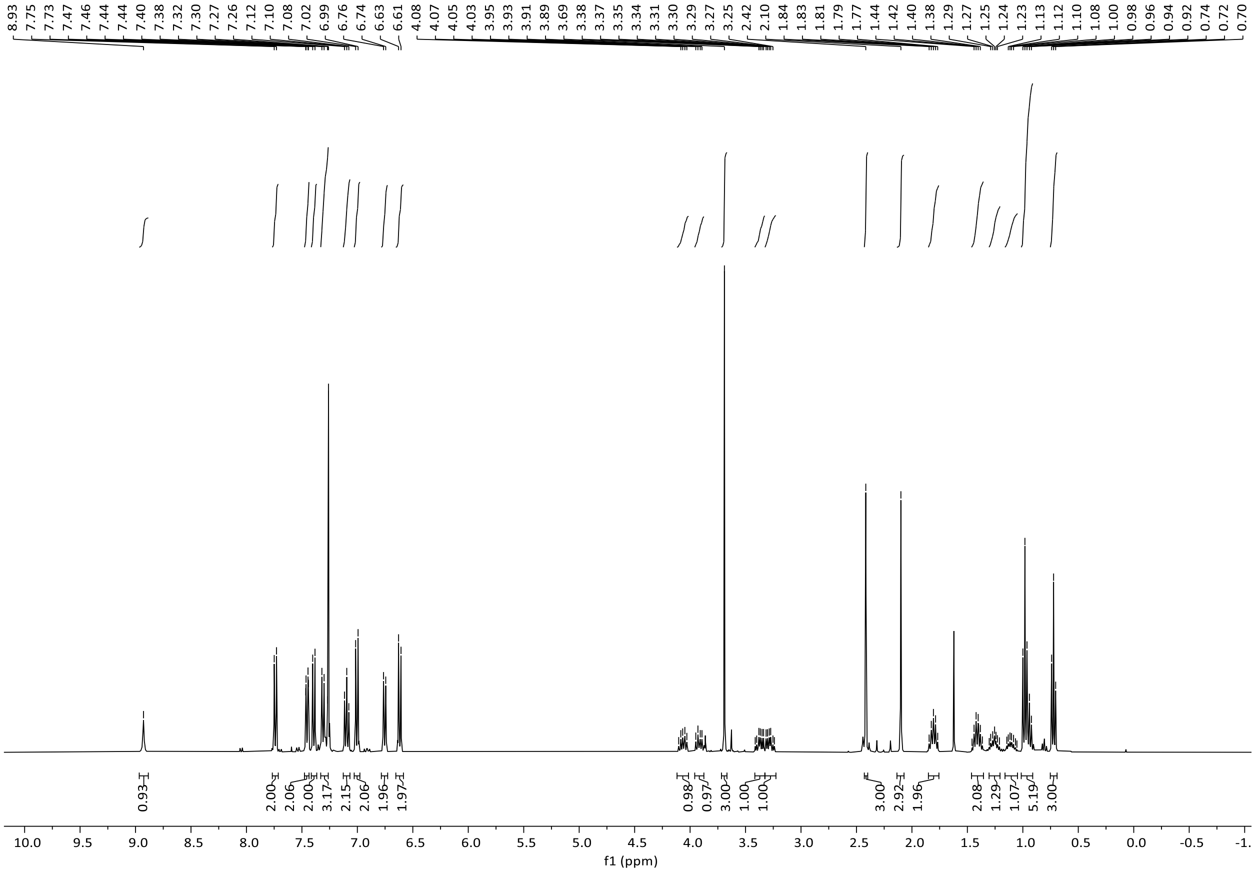

^{13}C NMR (101 MHz, $CDCl_3$)

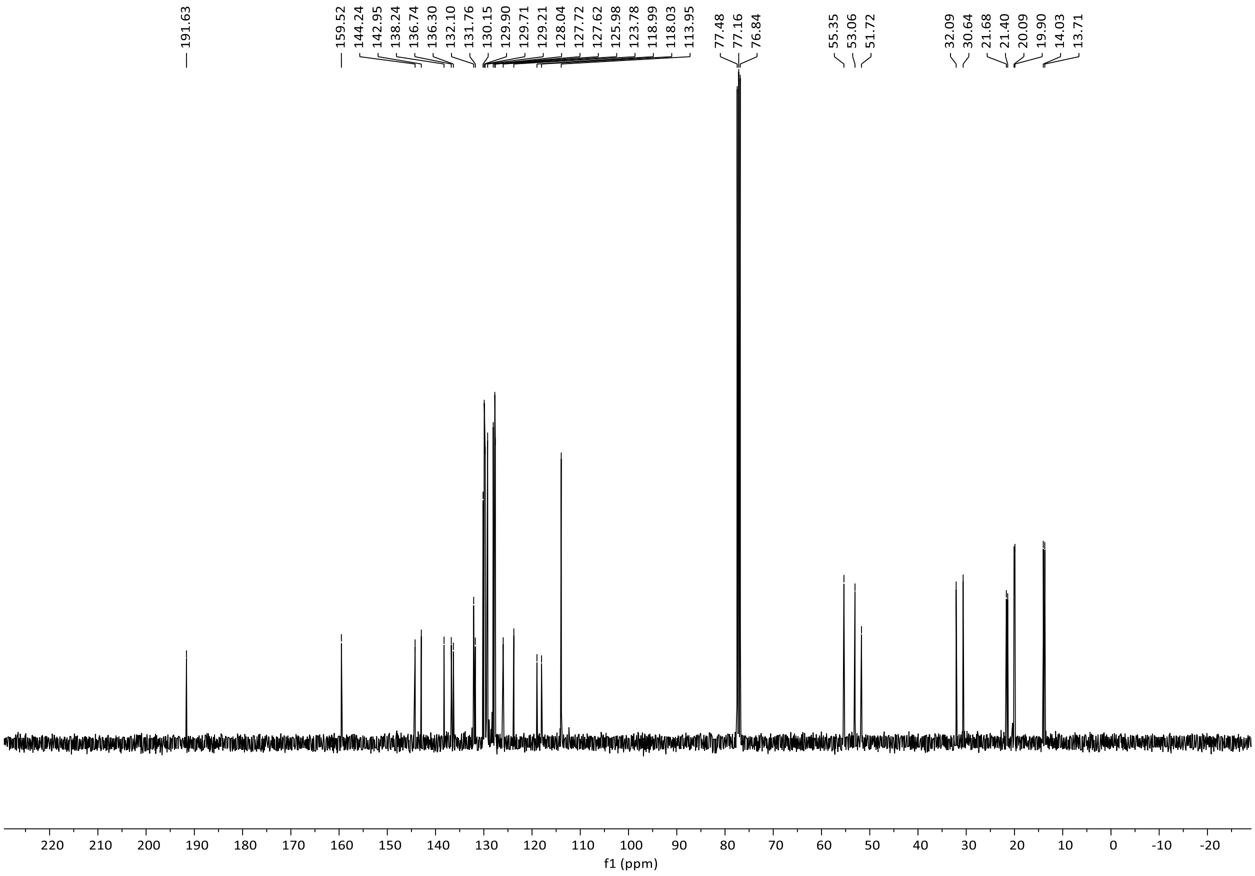

***N*,*N'*-(4-Benzoyl-5-(furan-2-yl)-1*H*-pyrrole-2,3-diyl)bis(*N*-butyl-4-methylbenzenesulfonamide), 2s**

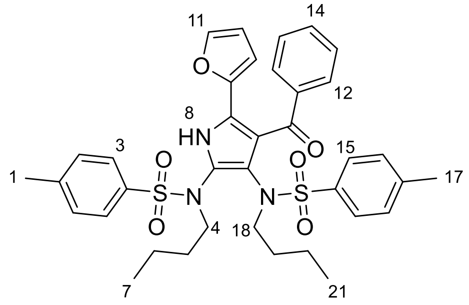

^{1}H NMR (400 MHz, $CDCl_3$)

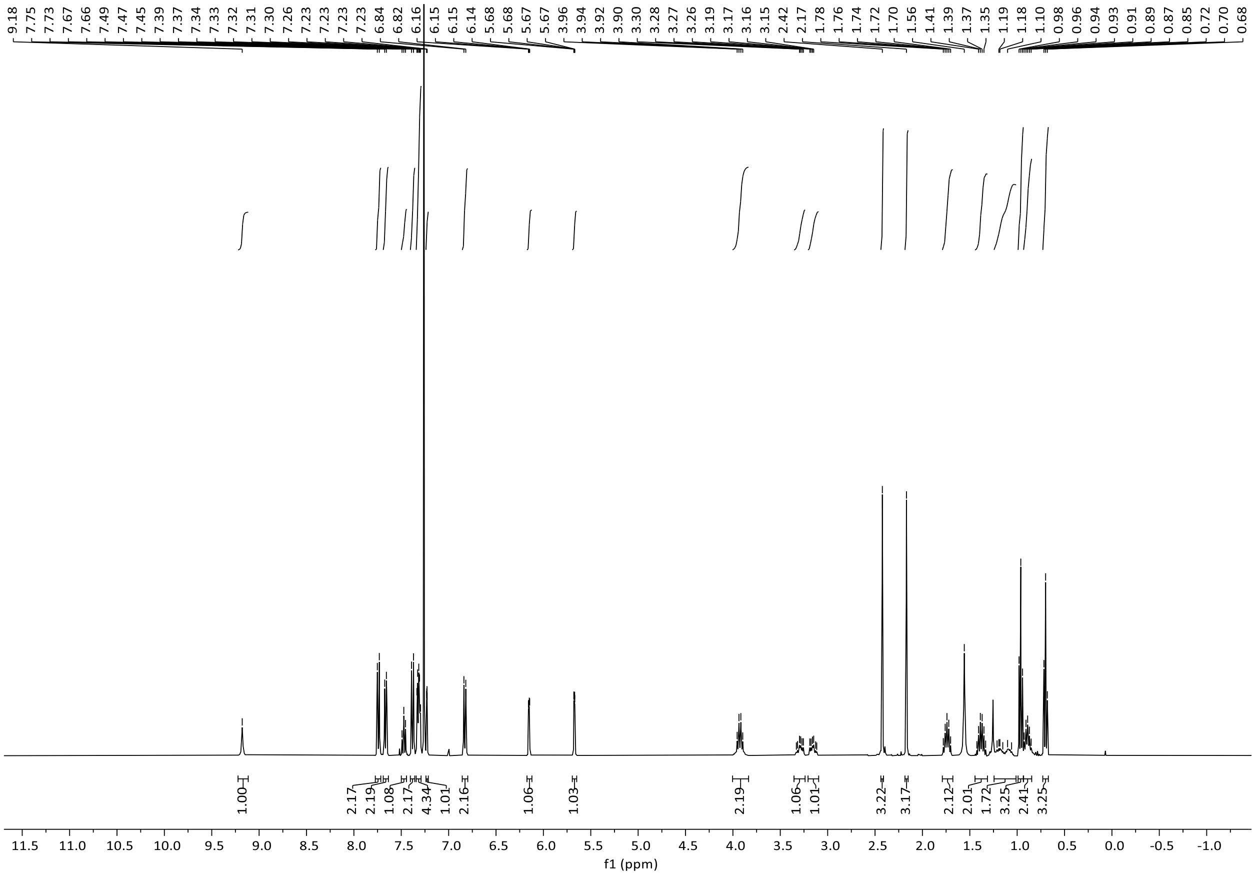

^{13}C NMR (101 MHz, $CDCl_3$)

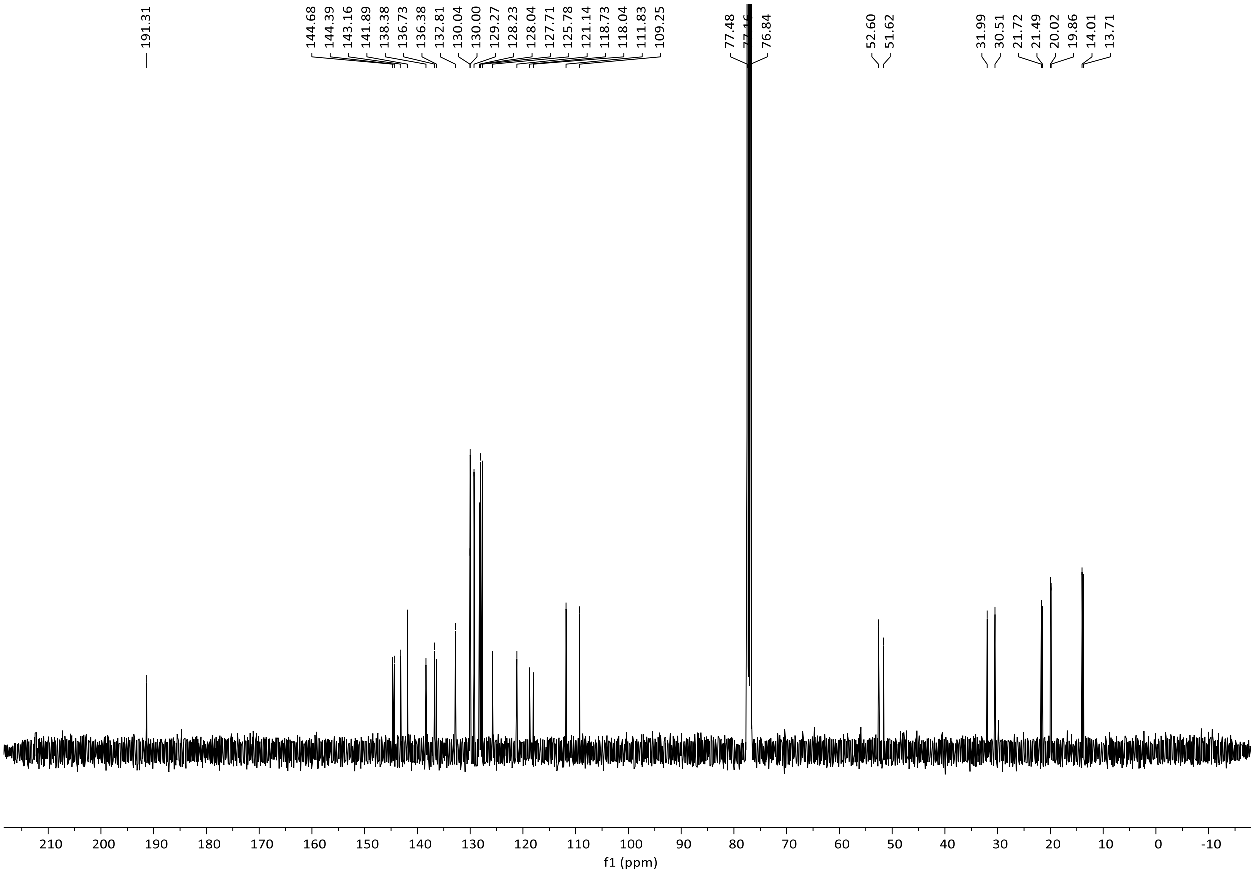

***N*-(4-Benzoyl-2-((*N*-cyclohexyl-4-methylphenyl)sulfonamido)-5-methyl-1*H*-pyrrol-3-yl)-*N*-butyl-4-methylbenzenesulfonamide, 2dc**

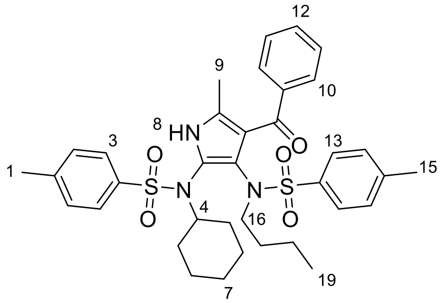

^{1}H NMR (500 MHz, C_6D_6)

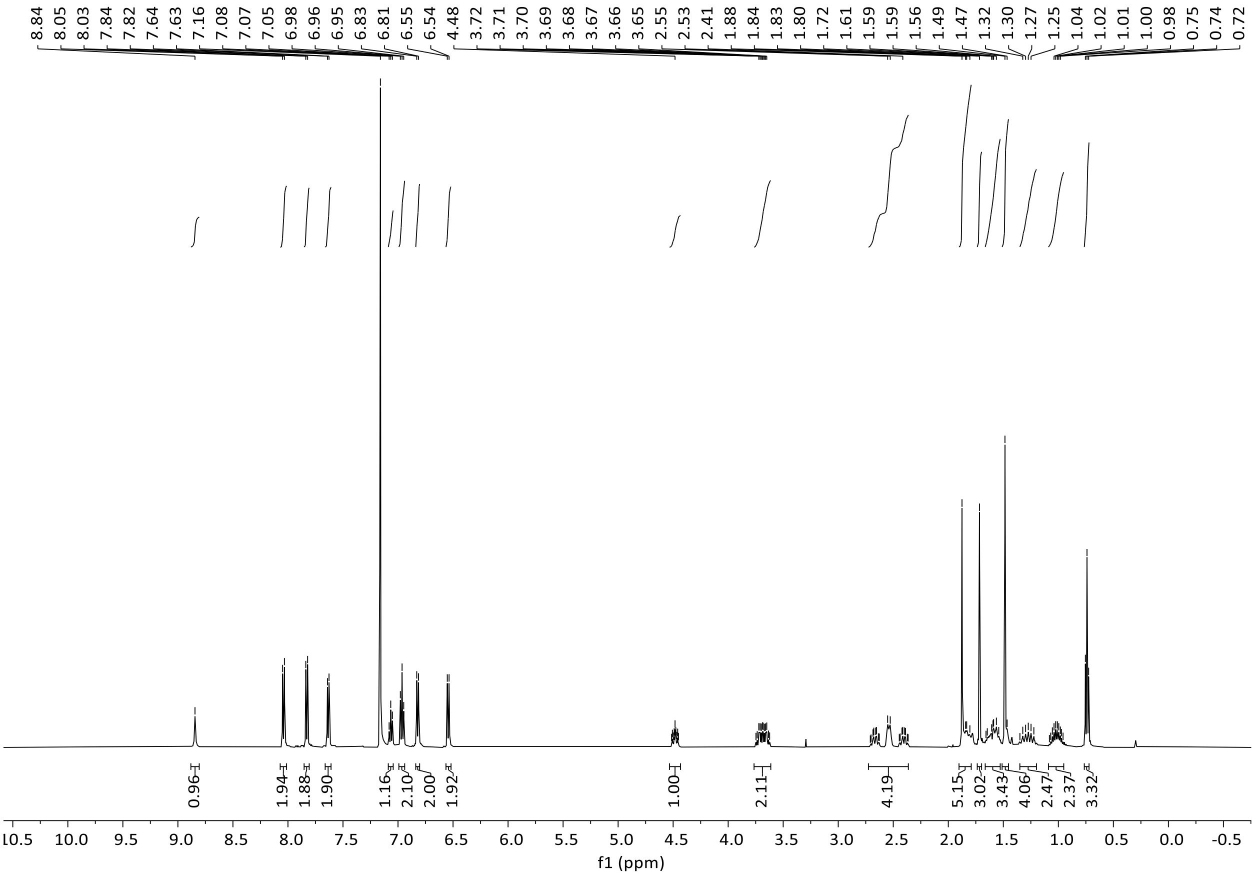

^{13}C NMR (126 MHz, C_6D_6)

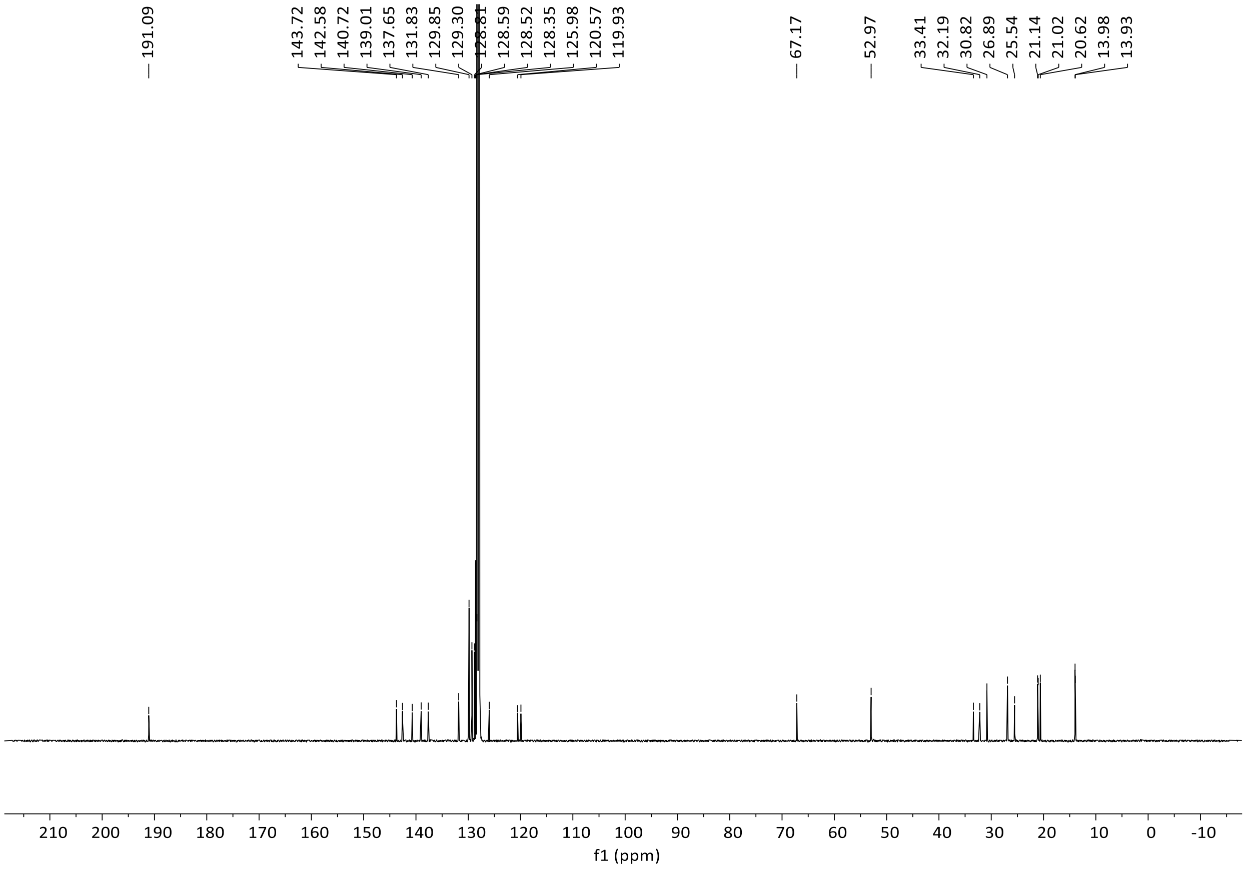

^{1}H – ^{1}H NOESY (500 MHz, C_6D_6)

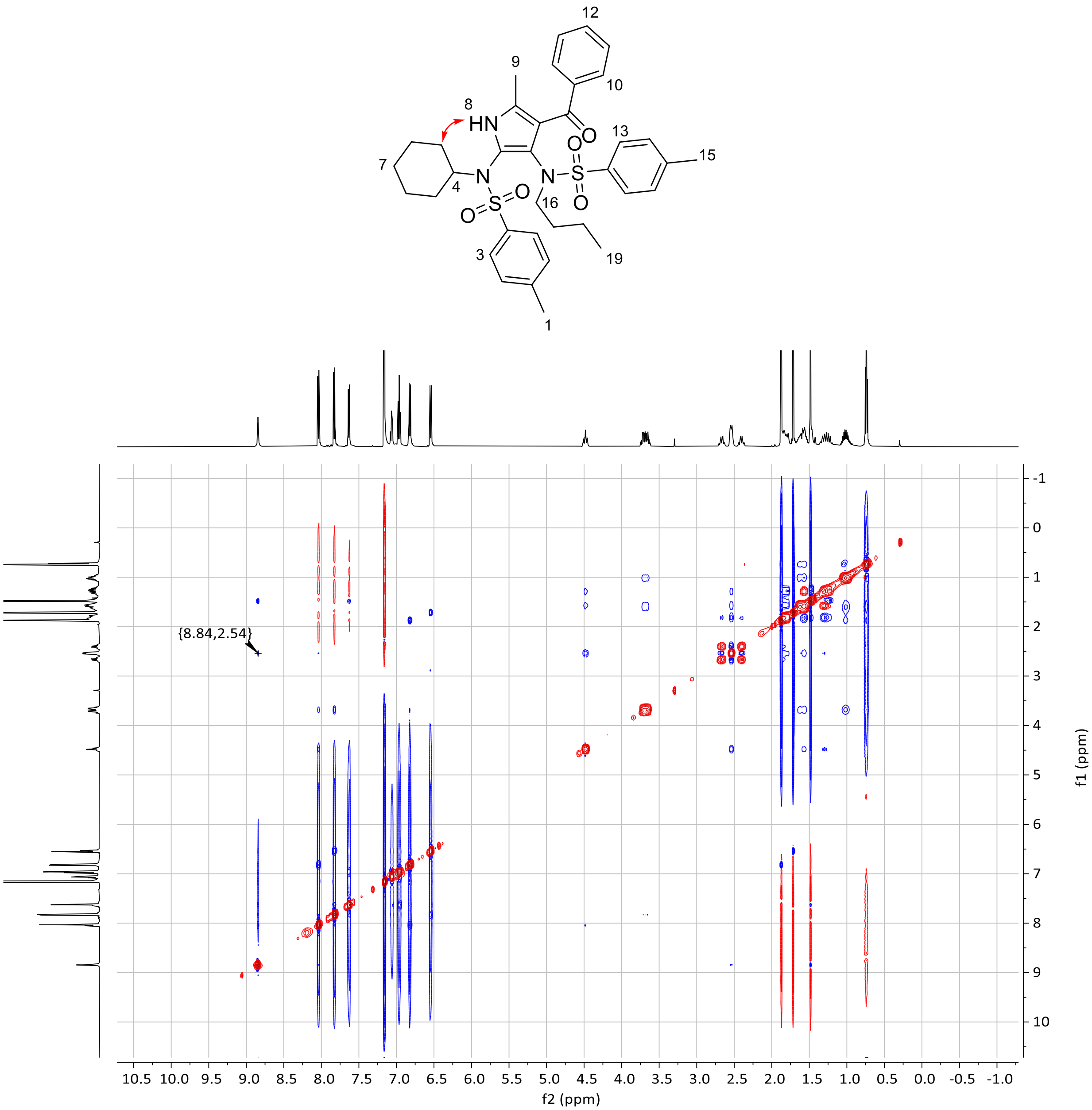

***N*-(4-Benzoyl-2-((*N*-cyclohexyl-4-methylphenyl)sulfonamido)-5-isopropyl-1*H*-pyrrol-3-yl)-*N*-butyl-4-methylbenzenesulfonamide, 2dd**

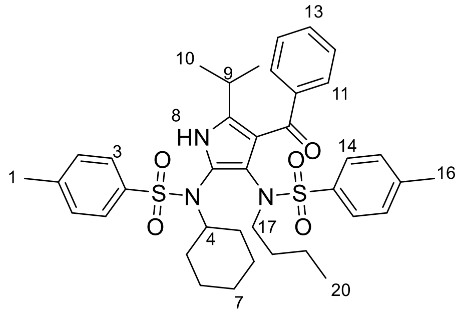

^{1}H NMR (500 MHz, $CDCl_3$)

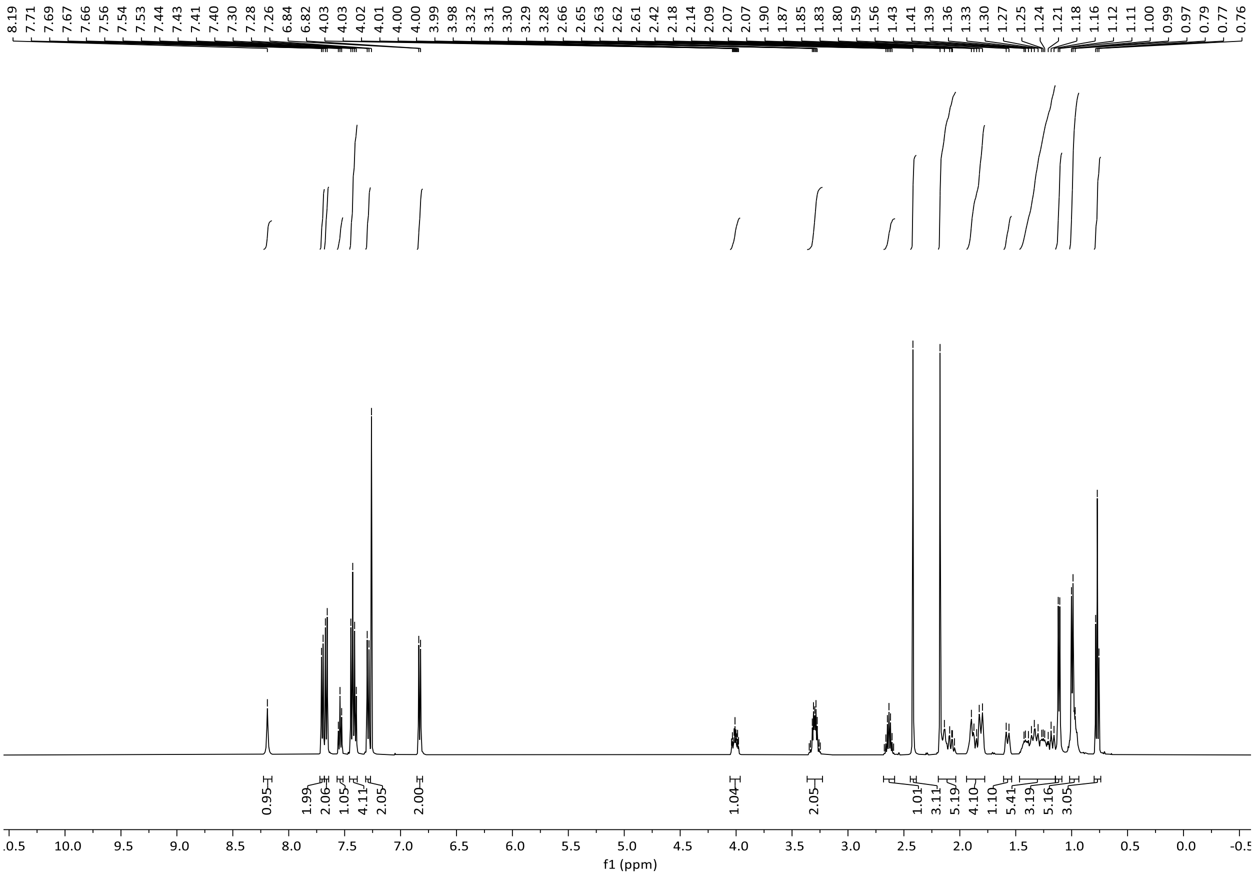

^{13}C NMR (126 MHz, $CDCl_3$)

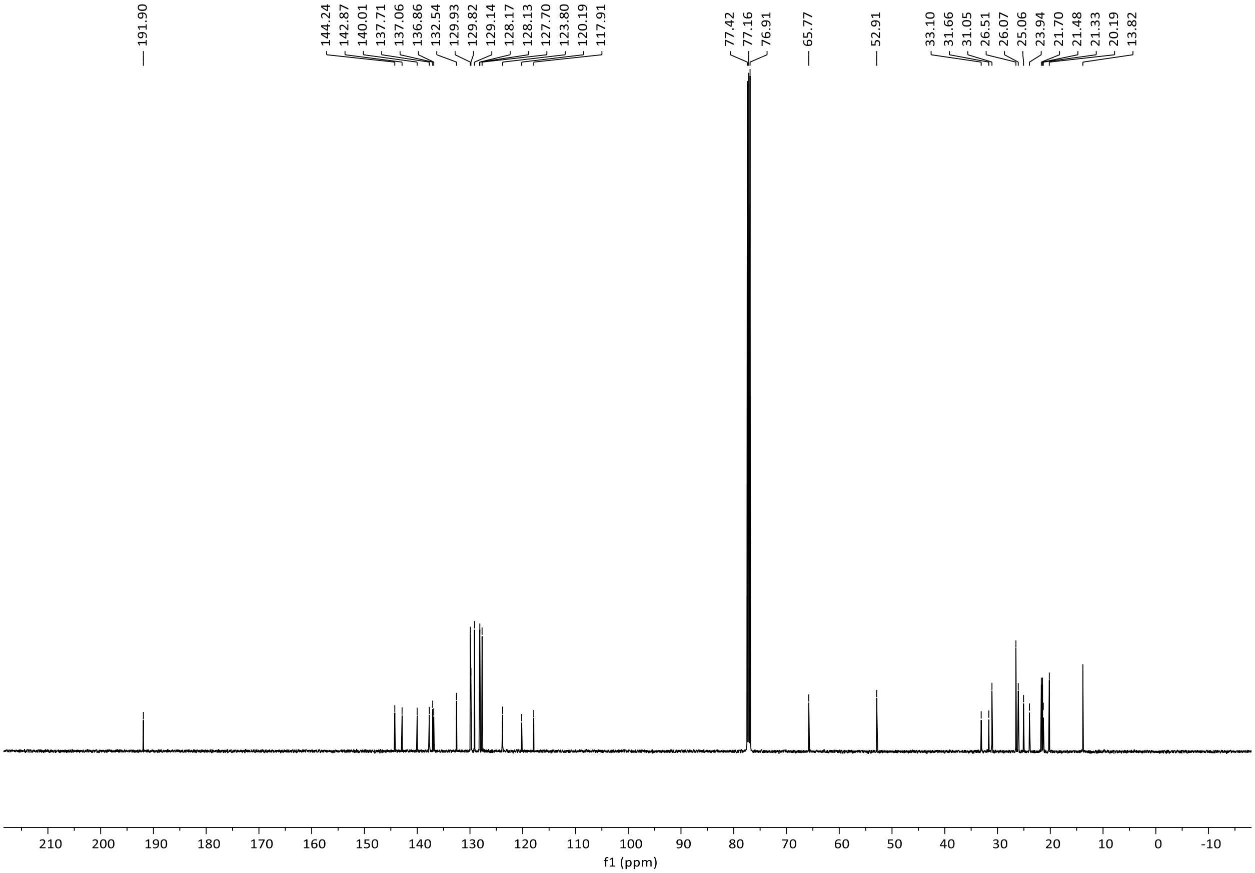

^{1}H – ^{1}H NOESY (500 MHz, $CDCl_3$)

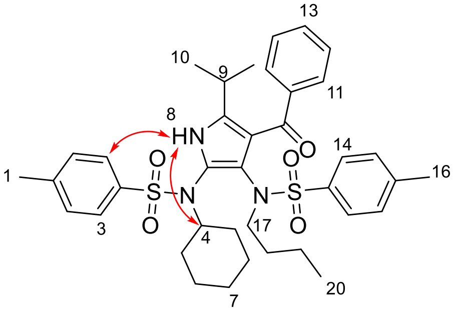

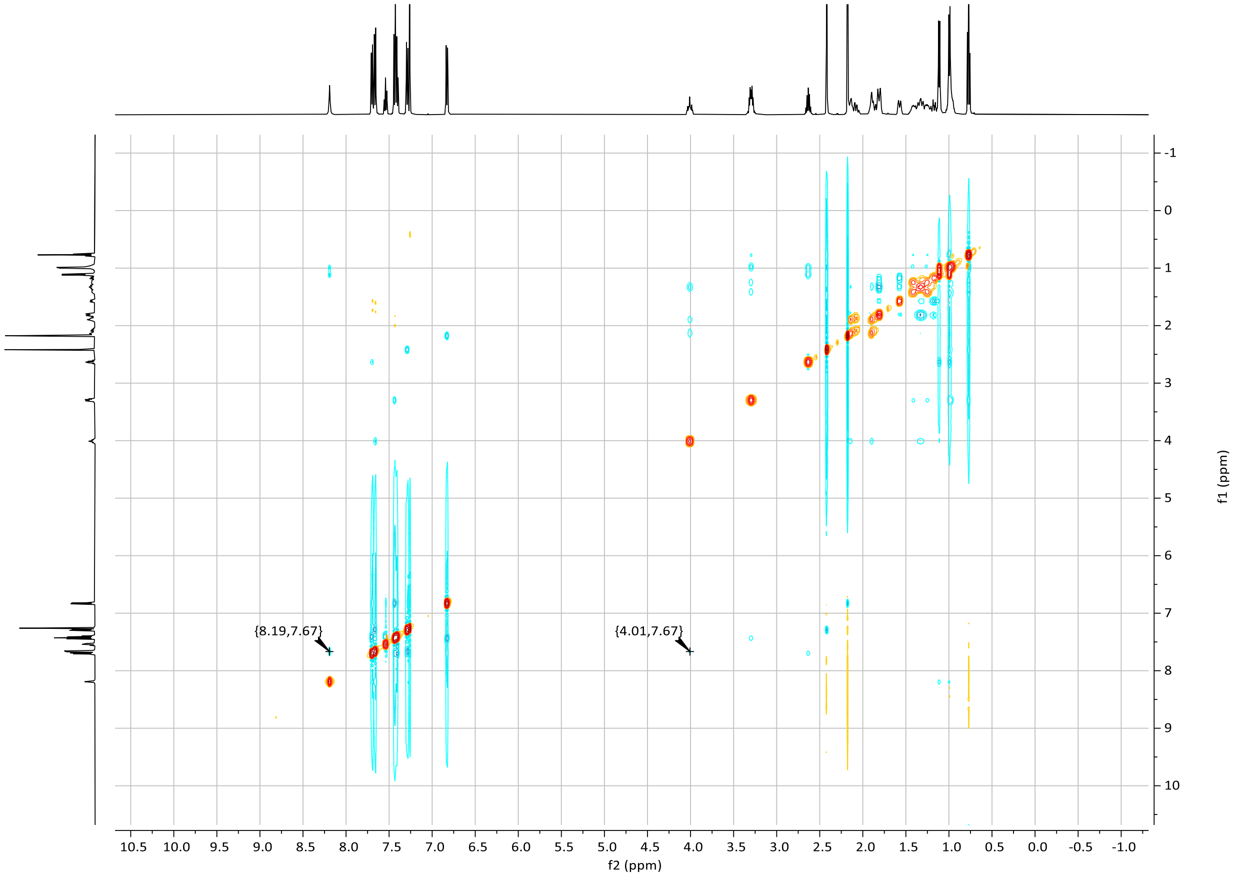

N,N'-(Benzo[f][1,4]oxazepine-2,3-diyl)bis(N-butyl-4-methylbenzenesulfonamide), 2ya

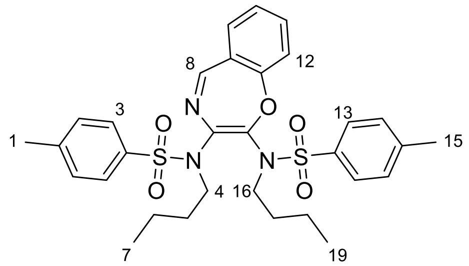

^{1}H NMR (400 MHz, C_6D_6)

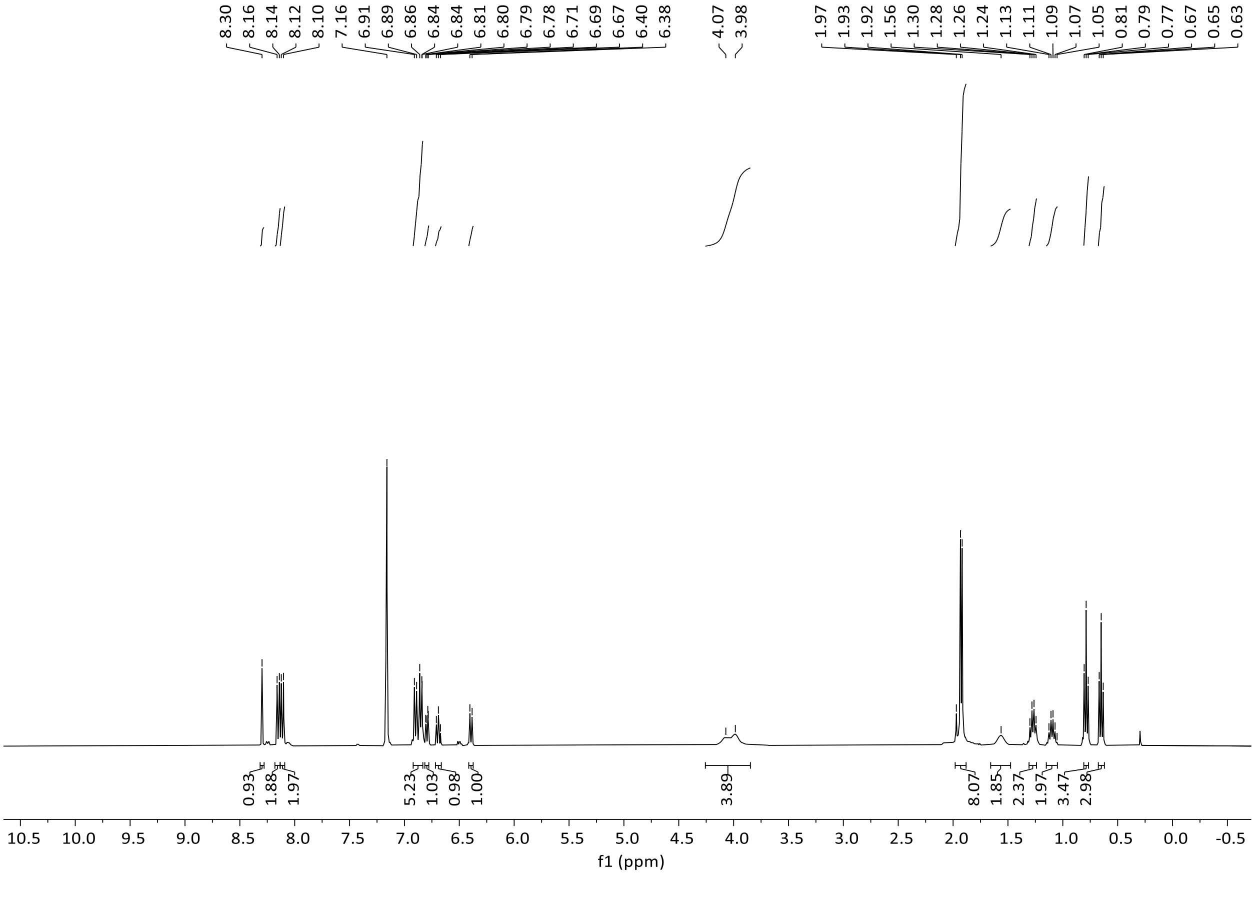

^{13}C NMR (101 MHz, C_6D_6)

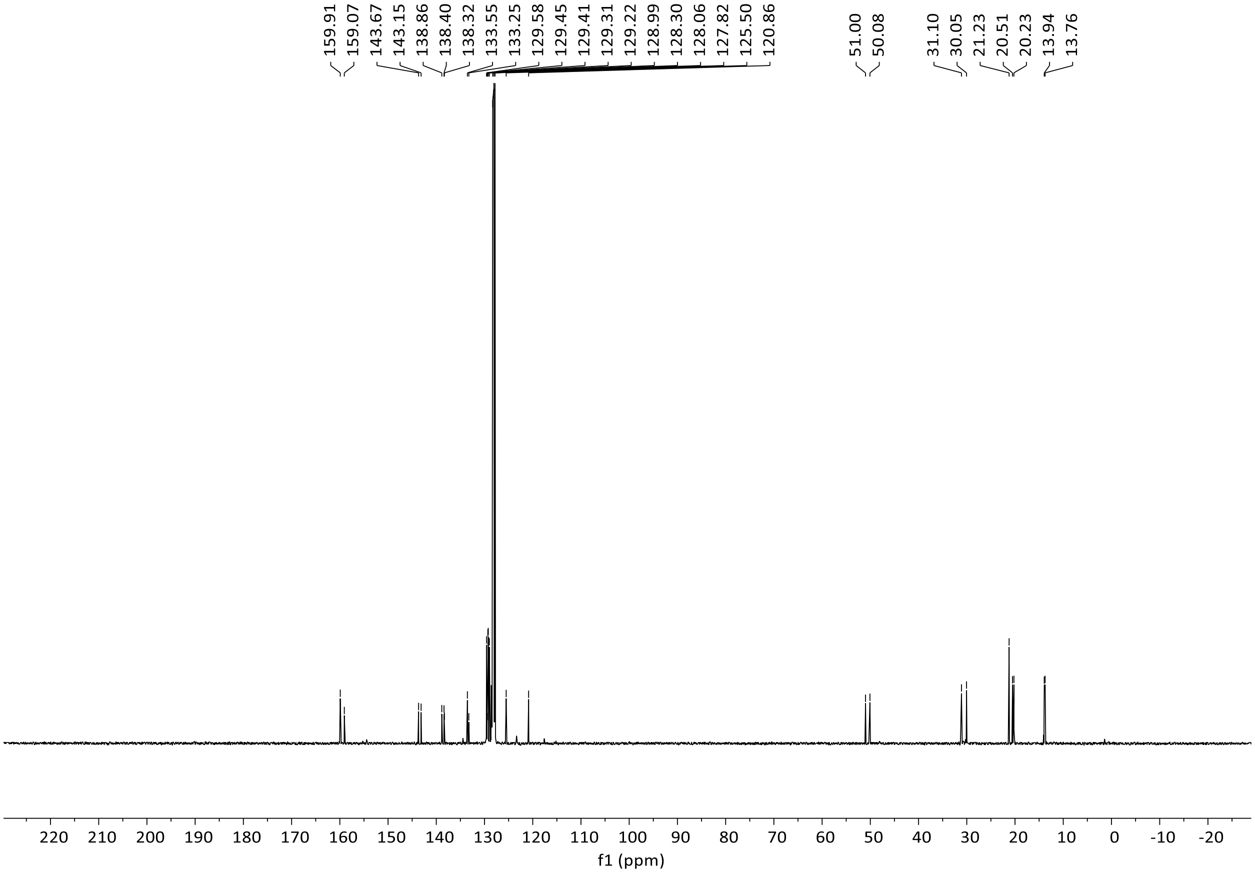

_N_,_N'_-(Naphtho[1,2-_f_][1,4]oxazepine-3,4-diyl)bis(_N_-butyl-4-methylbenzenesulfonamide), 2yb

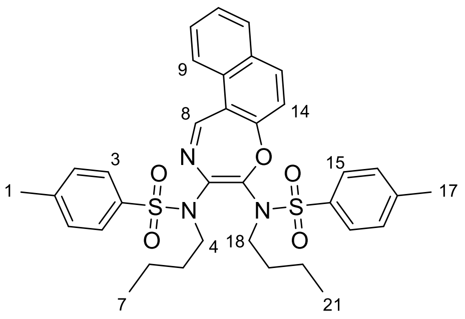

^{1}H NMR (400 MHz, $CDCl_3$)

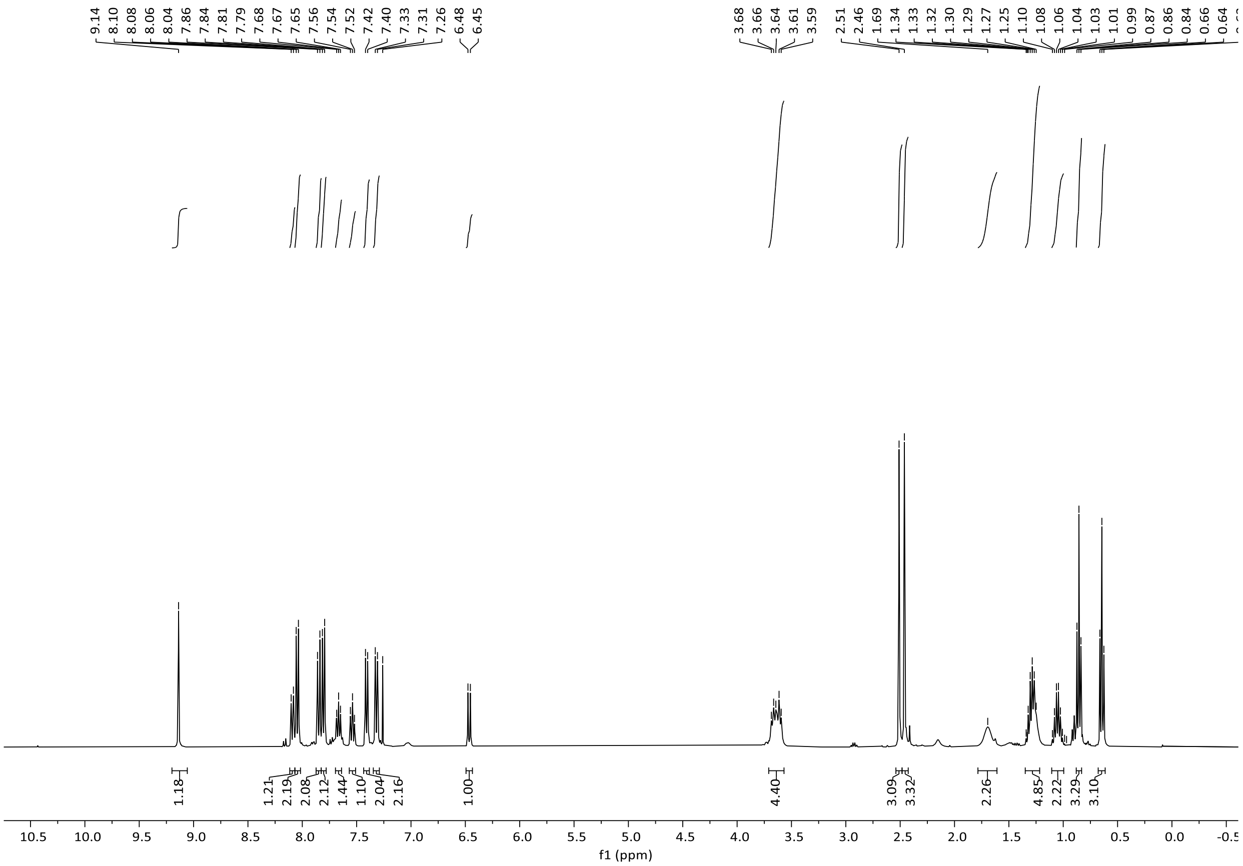

^{13}C NMR (101 MHz, $CDCl_3$)

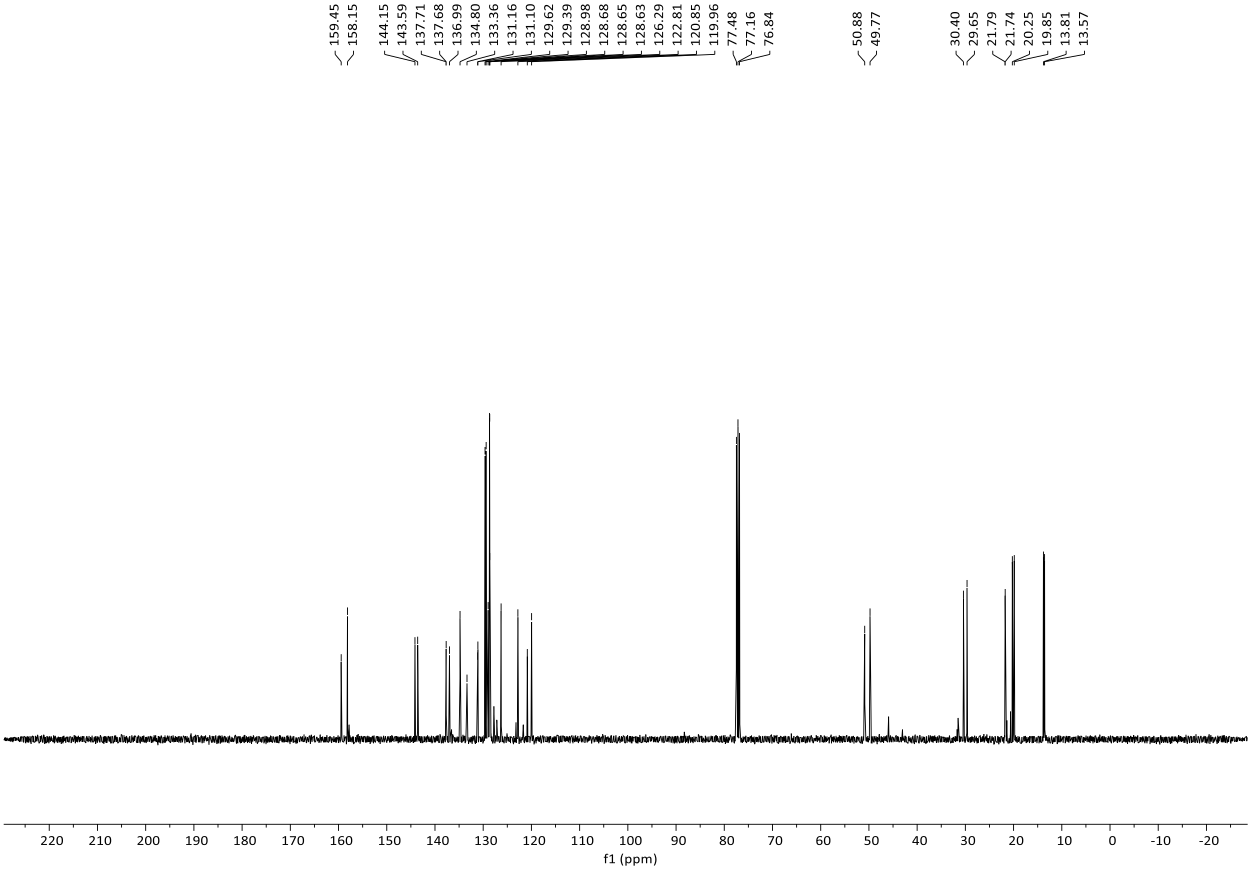

***N*,*N'*-(8-(Diethylamino)benzo[*f*][1,4]oxazepine-2,3-diyl)bis(*N*-butyl-4-methylbenzenesulfonamide). 2yc**

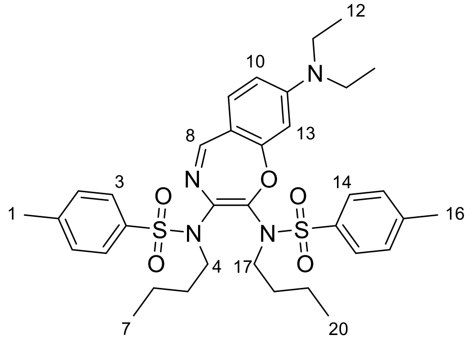

^{1}H NMR (400 MHz, $CDCl_3$)

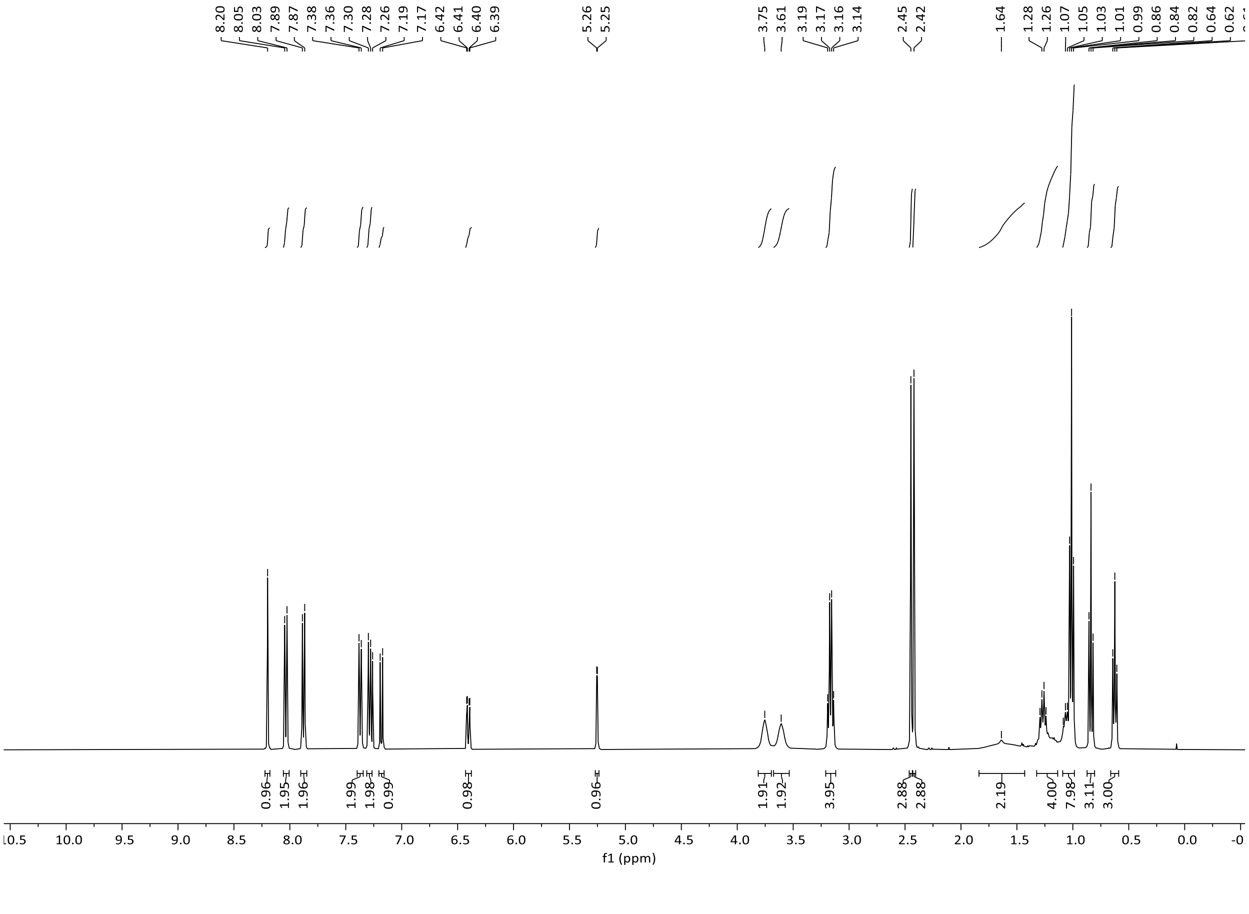

^{13}C NMR (101 MHz, $CDCl_3$)

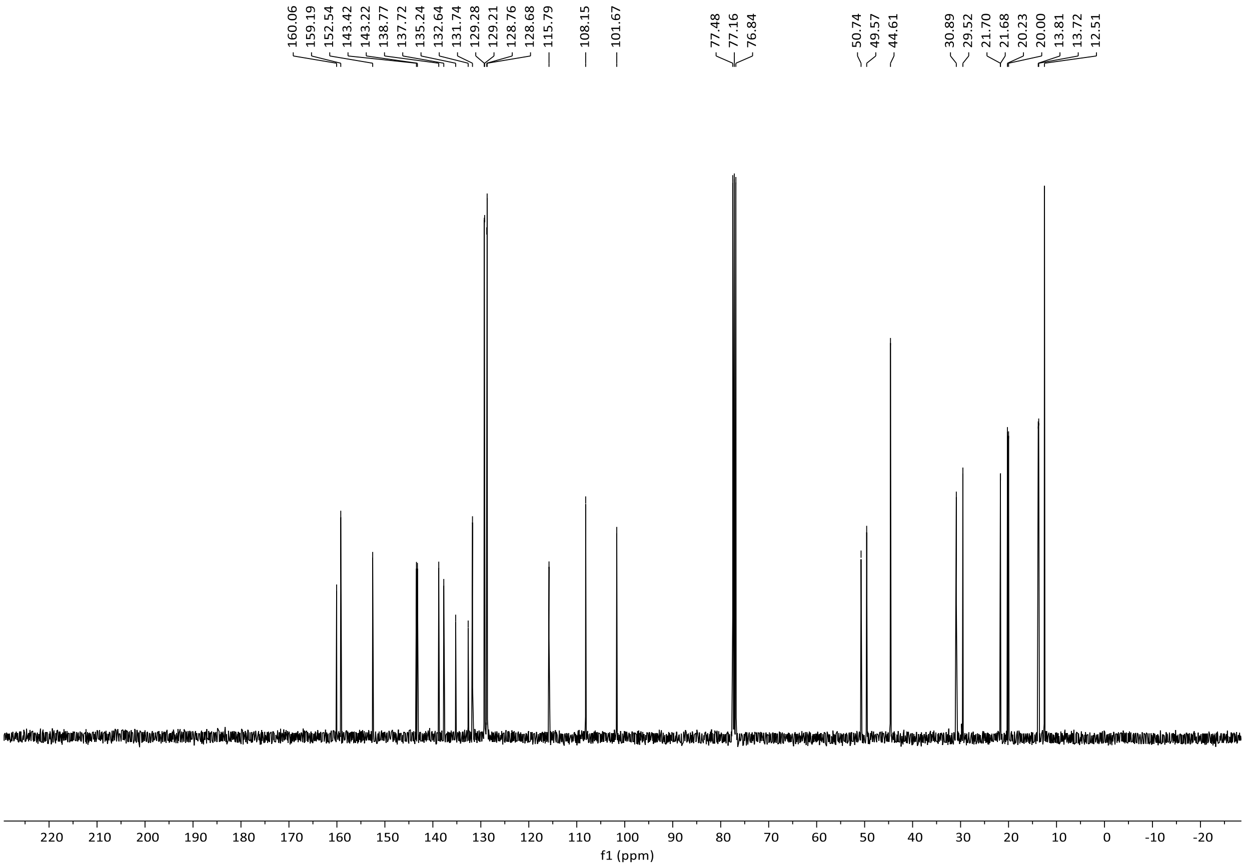

***N*,*N'*-(9-(tert-Butyl)benzo[*f*][1,4]oxazepine-2,3-diyl)bis(*N*-butyl-4-methylbenzenesulfonamide), 2yd**

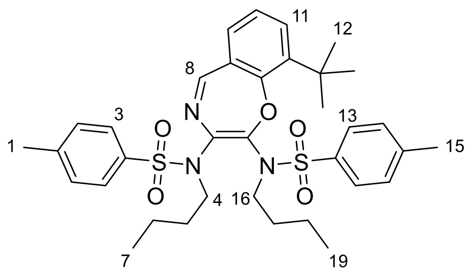

^{1}H NMR (400 MHz, $CDCl_3$)

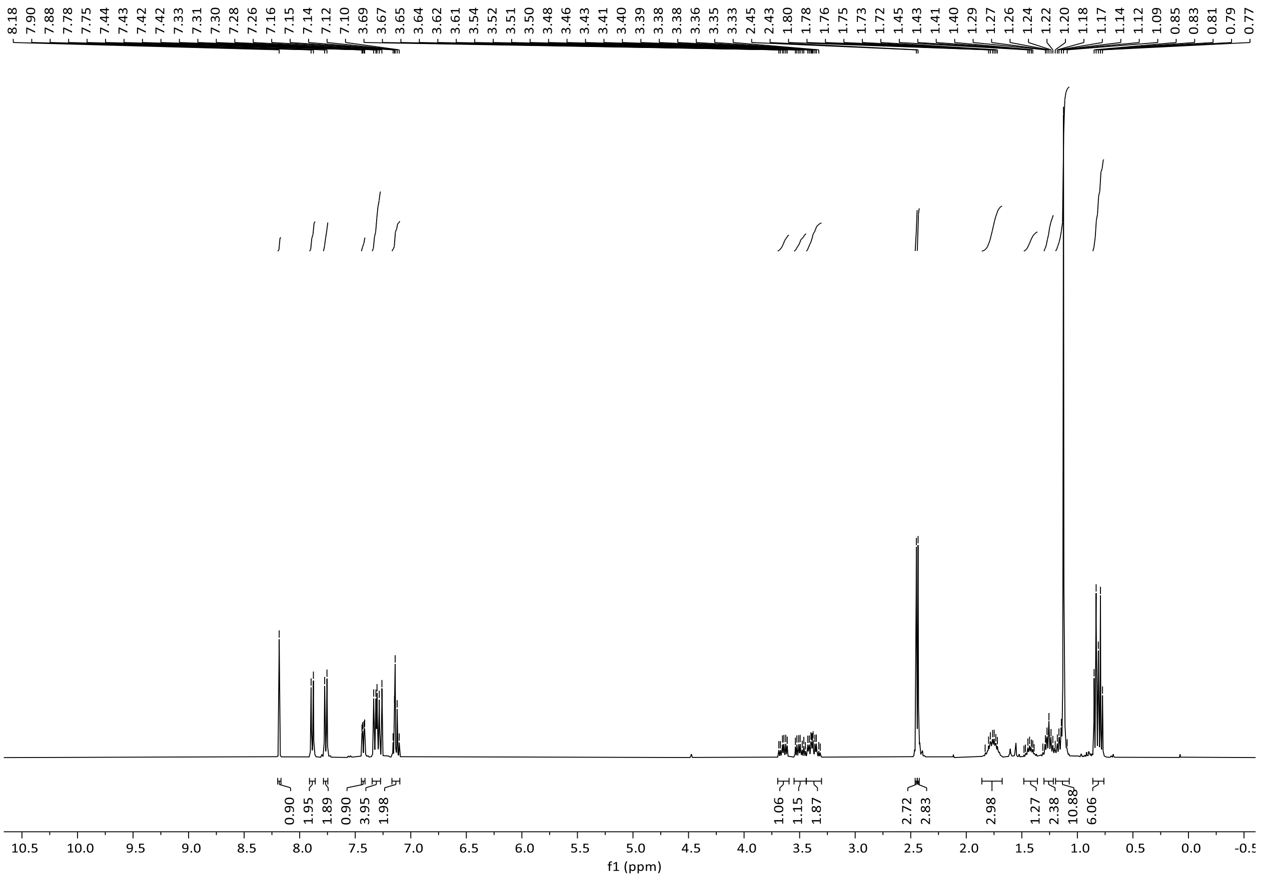

^{13}C NMR (101 MHz, $CDCl_3$)

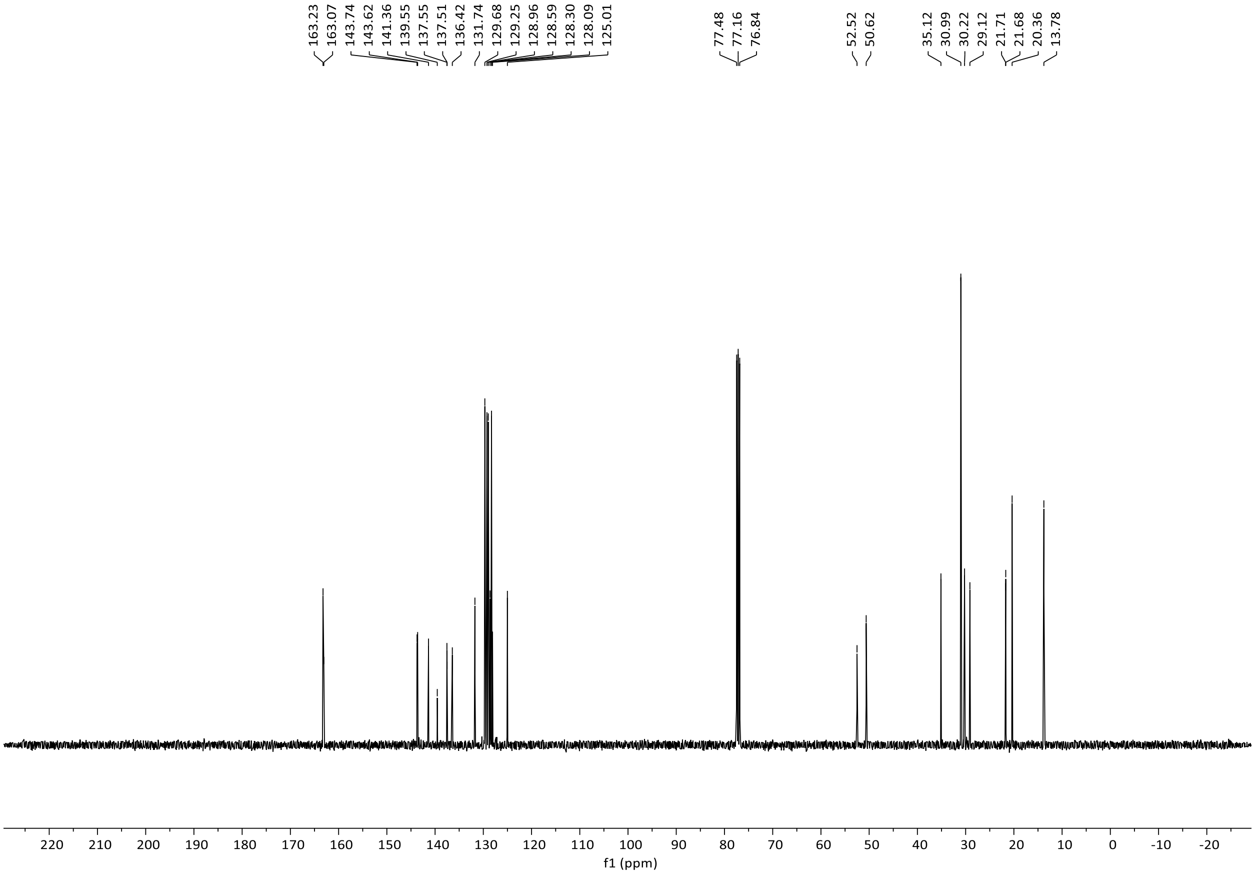

N,N'-(7-Formyl-1*H*-indole-2,3-diyl)bis(*N*-butyl-4-methylbenzenesulfonamide), 2z

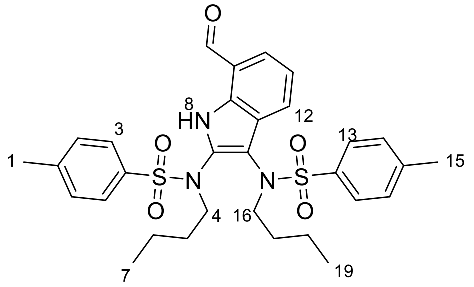

^{1}H NMR (400 MHz, $CDCl_3$)

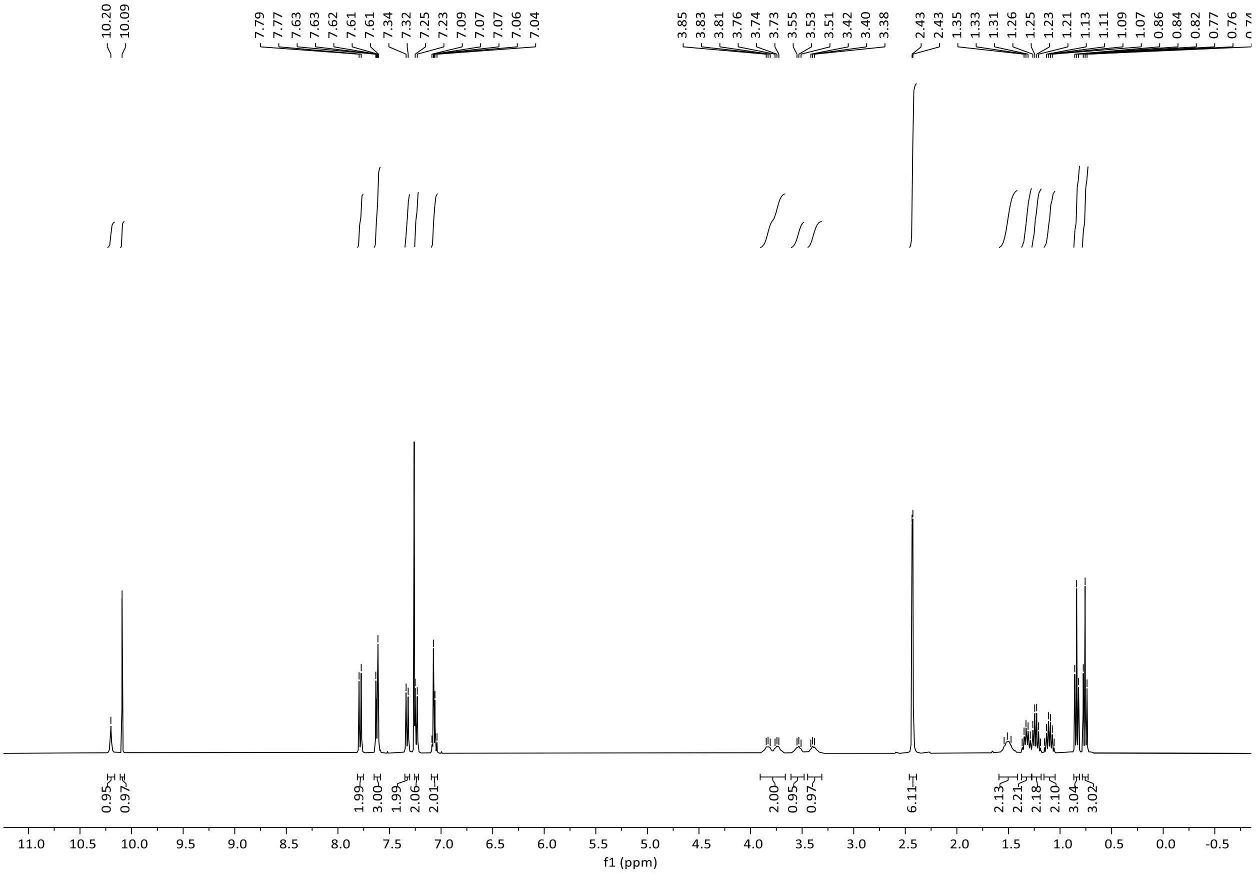

^{13}C NMR (101 MHz, $CDCl_3$)

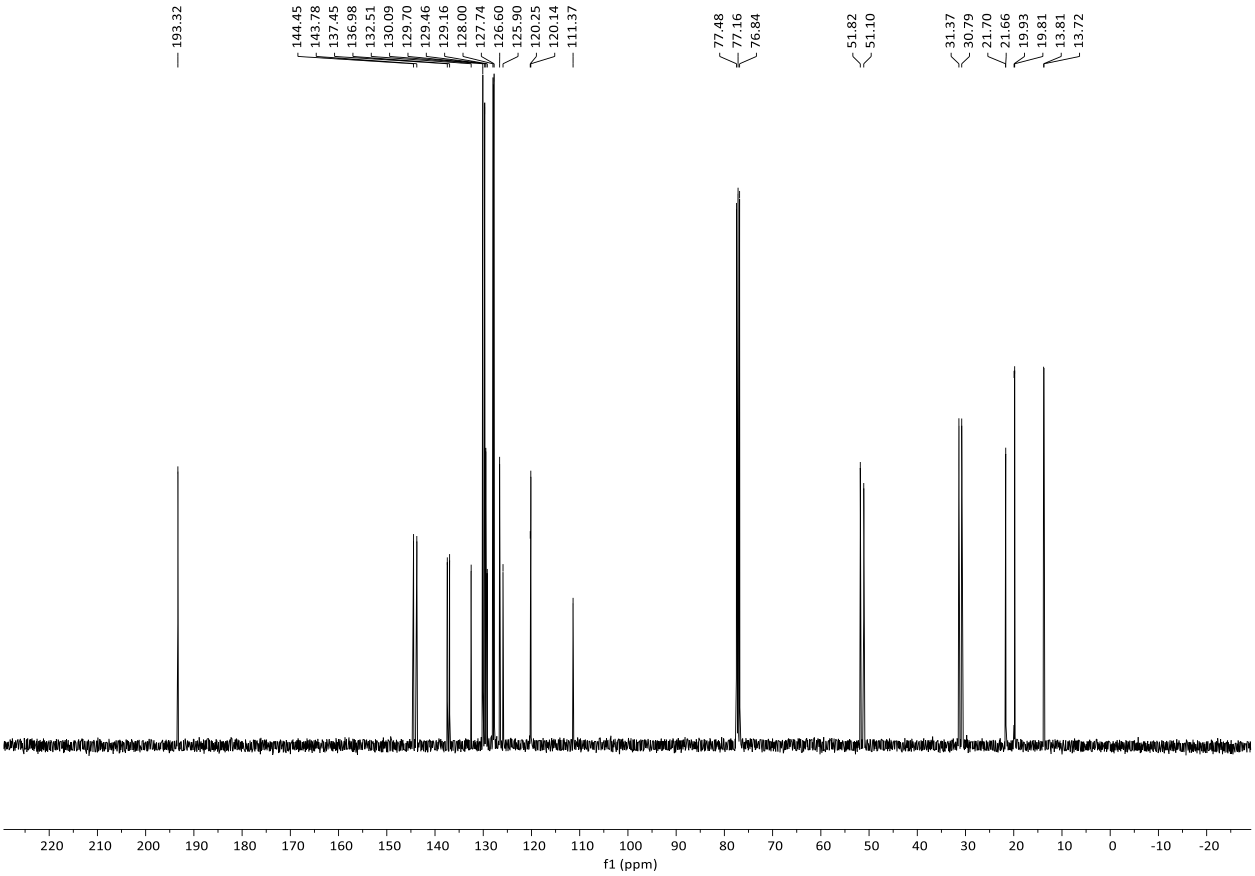

***N*,*N'*-(5-Bromo-4-formyl-1*H*-pyrrole-2,3-diyl)bis(*N*-butyl-4-methylbenzenesulfonamide), 3ab**

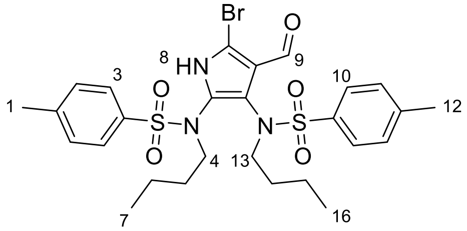

^{1}H NMR (400 MHz, $CDCl_3$)

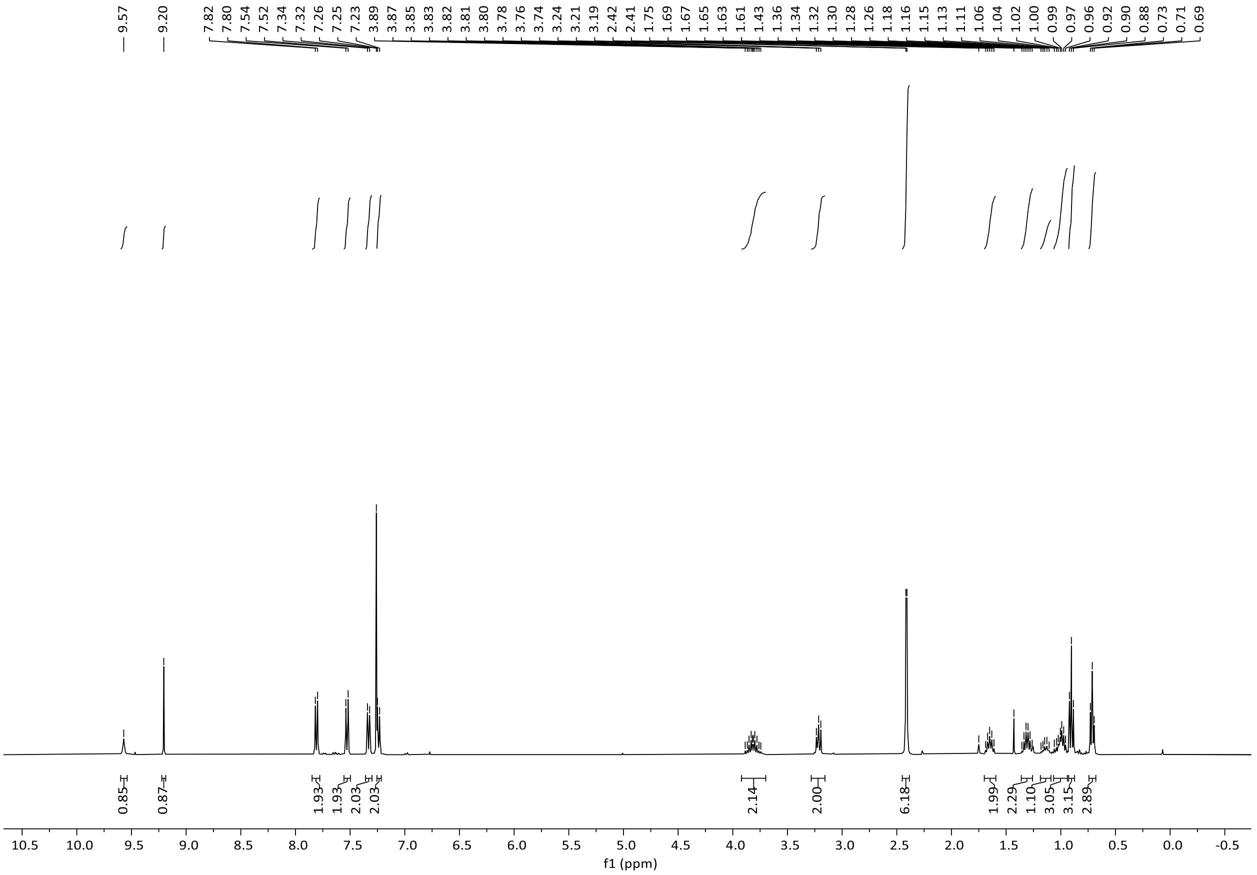

^{13}C NMR (101 MHz, $CDCl_3$)

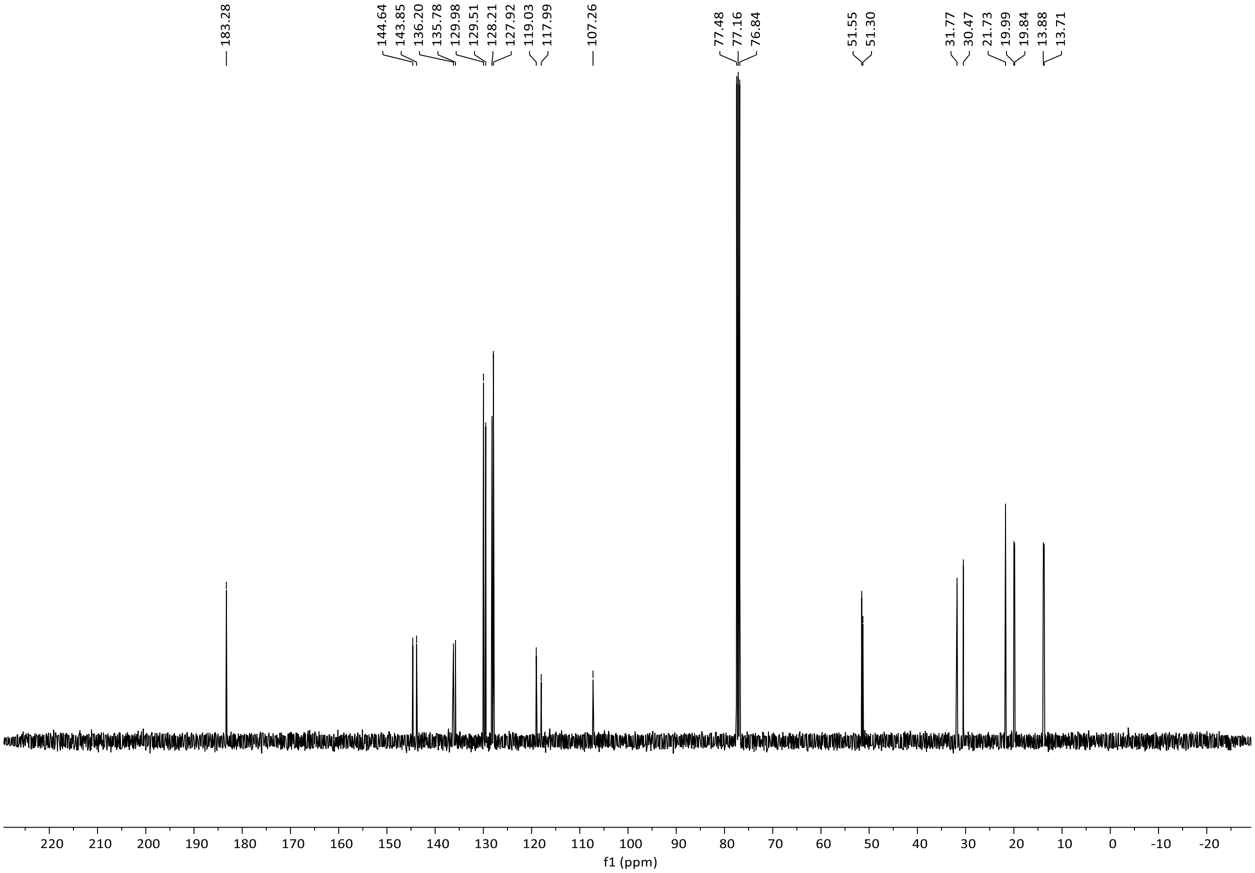

***N*,*N'*-(4-cyano-1*H*-pyrrole-2,3-diyl)bis(*N*-butyl-4-methylbenzenesulfonamide), 3ac**

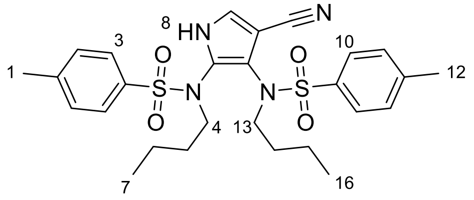

^{1}H NMR (400 MHz, $CDCl_3$)

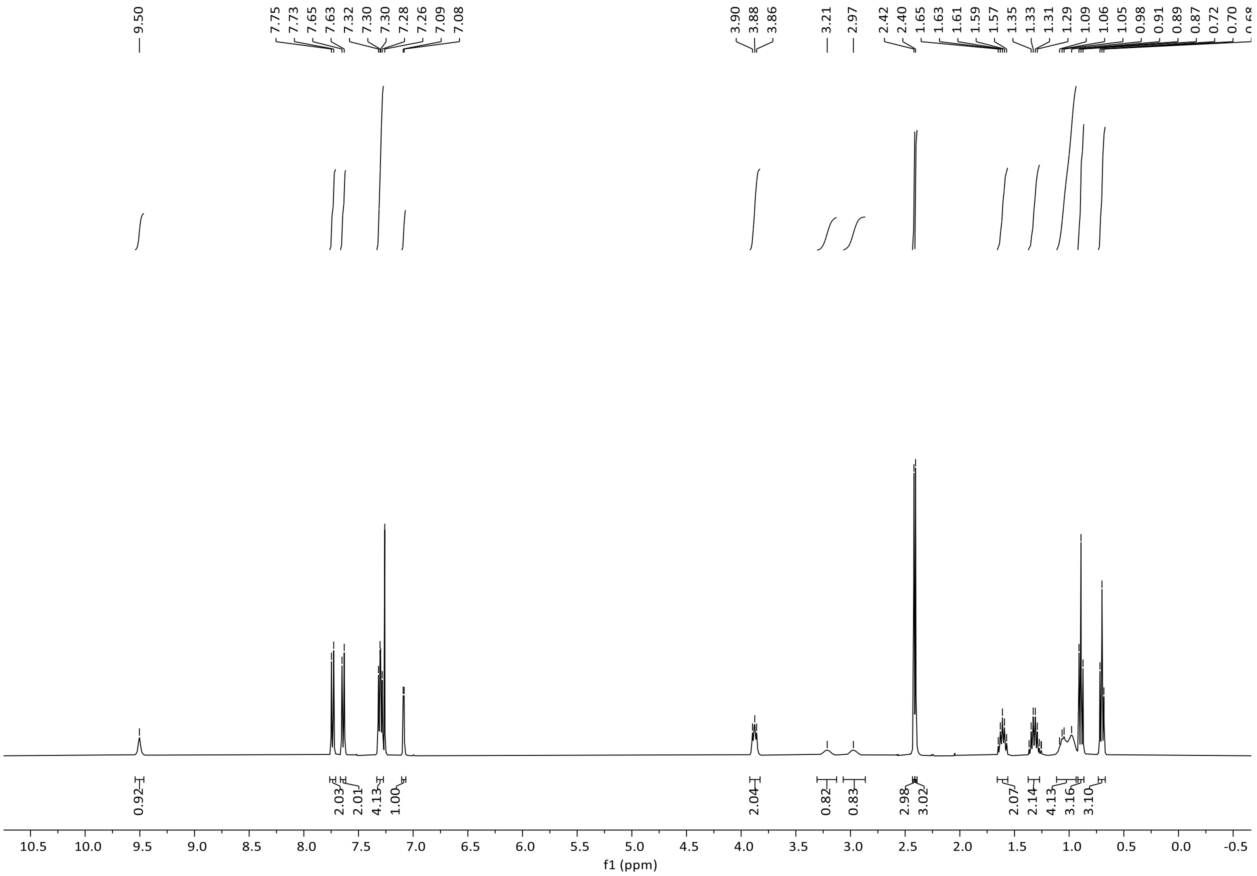

^{13}C NMR (101 MHz, $CDCl_3$)

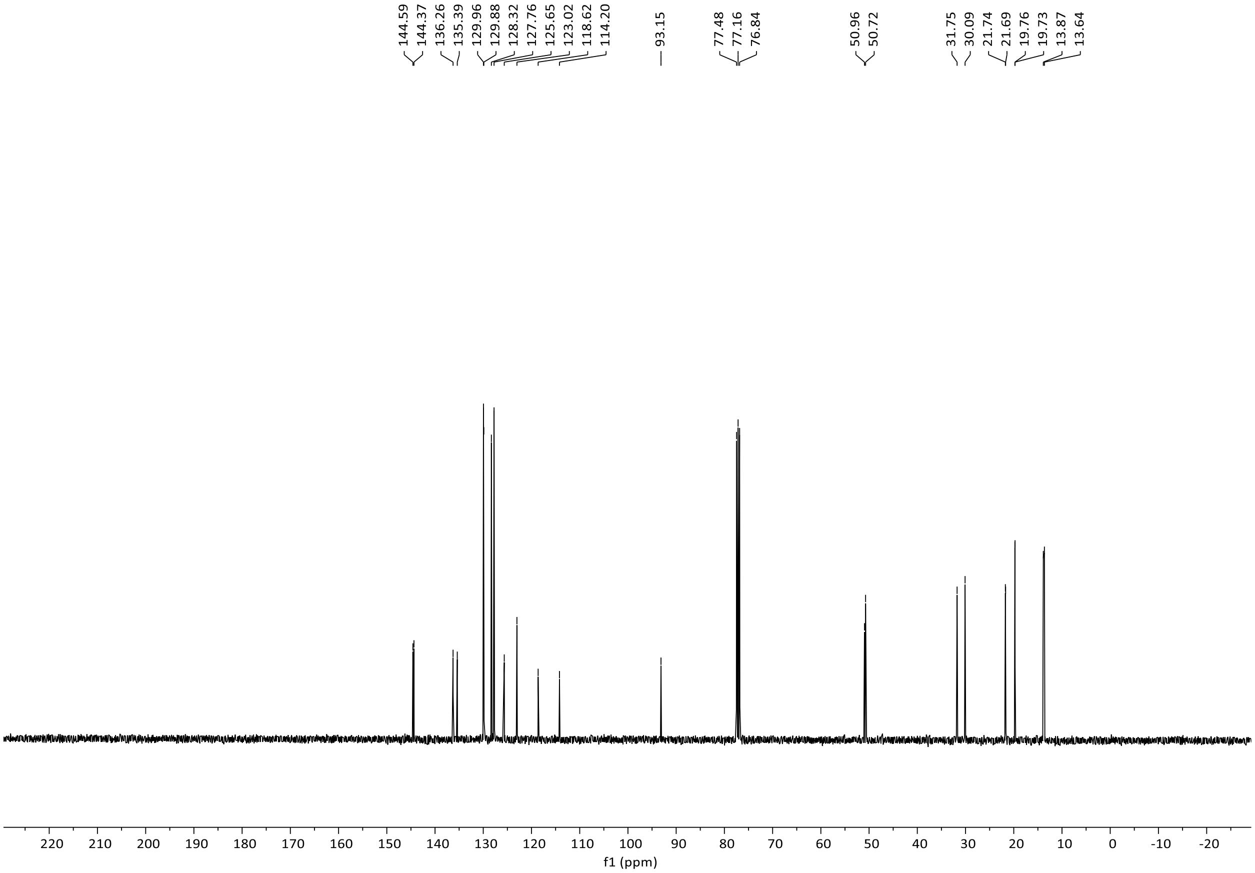

8 References for Supporting Information

(1) Mansfield, S. J.; Christensen, K. E.; Thompson, A. L.; Ma, K.; Jones, M. W.; Mekareeya, A.; Anderson, E. A. Copper-Catalyzed Synthesis and Applications of Yndiamides. *Angew. Chem. Int. Ed.* **2017**, *56* (46), 14428-14432.

(2) Tong, Z.; Garry, O. L.; Smith, P. J.; Jiang, Y.; Mansfield, S. J.; Anderson, E. A. Au(I)-Catalyzed Oxidative Functionalization of Yndiamides. *Org. Lett.* **2021**, *23* (12), 4888-4892.

(3) Hansen, T. V.; Wu, P.; Fokin, V. V. One-Pot Copper(I)-Catalyzed Synthesis of 3,5-Disubstituted Isoxazoles. *J. Org. Chem.* **2005**, *70* (19), 7761-7764.

(4) Poissonnet, G. A Simple and Convenient Synthesis of 1,2-Benzoxazoles Via Intramolecular Mitsunobu Reaction from Salicylaldoximes and Orthohydroxyarylketoximes. *Synth. Commun.* **1997**, *27* (22), 3839-3846.

(5) (a) Giri, S. S.; Liu, R.-S. Gold-catalyzed [4+3]- and [4+2]-annulations of 3-en-1-ynamides with isoxazoles via novel 6π-electrocyclizations of 3-azahepta trienyl cations. *Chem. Sci.* **2018**, *9* (11), 2991-2995. (b) Sahani, R. L.; Liu, R.-S. Development of Gold-catalyzed [4+1] and [2+2+1]/[4+2] Annulations between Propiolate Derivatives and Isoxazoles. *Angew. Chem. Int. Ed.* **2017**, *56* (4), 1026-1030.

(6) Pasunooti, K. K.; Banerjee, B.; Yap, T.; Jiang, Y.; Liu, C.-F. Auxiliary-Directed Pd-Catalyzed *γ*-C(sp^3)–H Bond Activation of α-Aminobutanoic Acid Derivatives. *Org. Lett.* **2015**, *17* (24), 6094-6097.

(7) Mondal, S.; Biswas, S.; Ghosh, K. G.; Sureshkumar, D. TEMPO-Mediated Selective Synthesis of Isoxazolines, 5-Hydroxy-2-isoxazolines, and Isoxazoles via Aliphatic *δ*-C(sp3)-H Bond Oxidation of Oximes. *Chem. Asian. J.* **2021**, *16* (17), 2439-2446.

(8) Debleds, O.; Gayon, E.; Ostaszuk, E.; Vrancken, E.; Campagne, J.-M. A Versatile Iron-Catalyzed Protocol for the One-Pot Synthesis of Isoxazoles or Isoxazolines from the Same Propargylic Alcohols. *Chem. Eur. J.* **2010**, *16* (40), 12207-12213.

(9) Kumar, G. R.; Kumar, Y. K.; Reddy, M. S. A direct access to isoxazoles from ynones using trimethylsilyl azide as amino surrogate under metal/catalyst free conditions. *Chem. Commun.* **2016**, *52* (39), 6589-6592.

(10) Pusch, S.; Schollmeyer, D.; Opatz, T. A Light-Induced Vinylogous Nazarov-Type Cyclization. *Org. Lett.* **2016**, *18* (13), 3043-3045.

(11) Fernandes, A. A. G.; da Silva, A. F.; Okada Jr., C. Y.; Suzukawa, V.; Cormanich, R. A.; Jurberg, I. D. General Platform for the Conversion of Isoxazol-5-ones to 3,5-Disubstituted Isoxazoles via Nucleophilic Substitutions and Palladium Catalyzed Cross-Coupling Strategies. *Eur. J. Org. Chem.* **2019**, *2019* (19), 3022-3034.
(12) Jiao, H.; Xinting, Z.; Wenquan, Y.; Junbiao, C. I-2-Mediated Oxidative C-O Bond Formation for the Synthesis of Isoxazoles. *Chinese J. Org. Chem.* **2018**, *38* (12), 3236-3241.
(13) Kung, K. K.-Y.; Lo, V. K.-Y.; Ko, H.-M.; Li, G.-L.; Chan, P.-Y.; Leung, K.-C.; Zhou, Z.; Wang, M.-Z.; Che, C.-M.; Wong, M.-K. Cyclometallated Gold(III) Complexes as Effective Catalysts for Synthesis of Propargylic Amines, Chiral Allenes and Isoxazoles. *Adv. Synth. Catal.* **2013**, *355* (10), 2055-2070.
(14) Khairnar, P. V.; Lung, T.-H.; Lin, Y.-J.; Wu, C.-Y.; Koppolu, S. R.; Edukondalu, A.; Karanam, P.; Lin, W. An Intramolecular Wittig Approach toward Heteroarenes: Synthesis of Pyrazoles, Isoxazoles, and Chromenone-oximes. *Org. Lett.* **2019**, *21* (11), 4219-4223.
(15) Kumar, P.; Kapur, M. Catalyst Control in Positional-Selective C–H Alkenylation of Isoxazoles and a Ruthenium-Mediated Assembly of Trisubstituted Pyrroles. *Org. Lett.* **2019**, *21* (7), 2134-2138.
(16) Tang, S.; He, J.; Sun, Y.; He, L.; She, X. Efficient and Regioselective One-Pot Synthesis of 3-Substituted and 3,5-Disubstituted Isoxazoles. *Org. Lett.* **2009**, *11* (17), 3982-3985.
(17) Murarka, S.; Studer, A. Zinc Triflate Catalyzed Aerobic Cross-Dehydrogenative Coupling (CDC) of Alkynes with Nitrones: A New Entry to Isoxazoles. *Org. Lett.* **2011**, *13* (10), 2746-2749.
(18) Kitamura, M.; Sakata, R.; Tashiro, N.; Ikegami, A.; Okauchi, T. Synthesis of Diazonaphthoquinones from Naphthols by Diazo-Transfer Reaction. *Bull. Chem. Soc. Jpn.* **2015**, *88* (6), 824-833.
(19) Tang, R.-J.; Milcent, T.; Crousse, B. Regioselective Halogenation of Arenes and Heterocycles in Hexafluoroisopropanol. *J. Org. Chem.* **2018**, *83* (2), 930-938.
(20) De Luca, L.; Giacomelli, G.; Porcheddu, A. Beckmann Rearrangement of Oximes under Very Mild Conditions. *J. Org. Chem.* **2002**, *67* (17), 6272-6274.
(21) Zhou, A. H.; He, Q.; Shu, C.; Yu, Y. F.; Liu, S.; Zhao, T.; Zhang, W.; Lu, X.; Ye, L. W. Atom-economic generation of gold carbenes: gold-catalyzed formal [3+2] cycloaddition between ynamides and isoxazoles. *Chem. Sci.* **2015**, *6* (2), 1265-1271.

(22) Zhou, L.; Yang, L.; Dai, S.; Gao, Y.; Fang, R.; Kirillov, A. M.; Yang, L. Insight into the reaction mechanism and chemoselectivity in the cycloaddition of ynamides and isoxazoles with H_2O. *Catal. Sci. Technol.* **2020**, *10* (1), 240-251.
(23) Gaussian 16, Rev. C.01, Frisch, M. J.; Trucks, G. W.; Schlegel, H. B.; Scuseria, G. E.; Robb, M. A.; Cheeseman, J. R.; Scalmani, G.; Barone, V.; Petersson, G. A.; Nakatsuji, H.; et al.; Gaussian, Inc., Wallingford CT, 2016.
(24) Neese, F.; Wennmohs, F.; Becker, U.; Riplinger, C. The ORCA quantum chemistry program package. *J. Chem. Phys.* **2020**, *152* (22), 224108.
(25) Zhao, Y.; Truhlar, D. G. The M06 suite of density functionals for main group thermochemistry, thermochemical kinetics, noncovalent interactions, excited states, and transition elements: two new functionals and systematic testing of four M06-class functionals and 12 other functionals. *Theor. Chem. Acc.* **2008**, *120* (1), 215-241.
(26) Hehre, W. J.; Ditchfield, R.; Pople, J. A. Self—Consistent Molecular Orbital Methods. XII. Further Extensions of Gaussian—Type Basis Sets for Use in Molecular Orbital Studies of Organic Molecules. *J. Chem. Phys.* **1972**, *56* (5), 2257-2261.
(27) Andrae, D.; Häußermann, U.; Dolg, M.; Stoll, H.; Preuß, H. Energy-adjusted ab initio pseudopotentials for the second and third row transition elements. *Theor. Chim. Acta.* **1990**, *77* (2), 123-141.
(28) Liakos, D. G.; Guo, Y.; Neese, F. Comprehensive Benchmark Results for the Domain Based Local Pair Natural Orbital Coupled Cluster Method (DLPNO-CCSD(T)) for Closed- and Open-Shell Systems. *J. Phys. Chem. A.* **2020**, *124* (1), 90-100.
(29) Weigend, F.; Ahlrichs, R. Balanced basis sets of split valence, triple zeta valence and quadruple zeta valence quality for H to Rn: Design and assessment of accuracy. *Phys. Chem. Chem. Phys.* **2005**, *7* (18), 3297-3305.
(30) (a) Cossi, M.; Barone, V.; Cammi, R.; Tomasi, J. Ab initio study of solvated molecules: a new implementation of the polarizable continuum model. *Chem. Phys. Lett.* **1996**, *255* (4), 327-335. (b) Mennucci, B.; Tomasi, J. Continuum solvation models: A new approach to the problem of solute's charge distribution and cavity boundaries. *J. Chem. Phys.* **1997**, *106* (12), 5151-5158.
(31) Reed, A. E.; Curtiss, L. A.; Weinhold, F. Intermolecular interactions from a natural bond orbital, donor-acceptor viewpoint. *Chem. Rev.* **1988**, *88* (6), 899-926.

(32) Palatinus, L.; Chapuis, G. SUPERFLIP - a computer program for the solution of crystal structures by charge flipping in arbitrary dimensions. *J. Appl. Cryst.* **2007**, *40* (4), 786-790.
(33) (a) Betteridge, P. W.; Carruthers, J. R.; Cooper, R. I.; Prout, K.; Watkin, D. J. CRYSTALS version 12: software for guided crystal structure analysis. *J. Appl. Cryst.* **2003**, *36* (6), 1487. (b) Cooper, R. I.; Thompson, A. L.; Watkin, D. J. CRYSTALS enhancements: dealing with hydrogen atoms in refinement. *J. Appl. Cryst.* **2010**, *43* (5-1), 1100-1107.
